# Differences in Pregnancy Metabolic Profiles and Their Determinants between White European and South Asian Women: Findings from the Born in Bradford Cohort

**DOI:** 10.3390/metabo9090190

**Published:** 2019-09-18

**Authors:** Kurt Taylor, Diana L. Santos Ferreira, Jane West, Tiffany Yang, Massimo Caputo, Deborah A. Lawlor

**Affiliations:** 1 Population Health Science, Bristol Medical School, Bristol BS8 2BN, UK; diana.santosferreira@bristol.ac.uk (D.L.S.F.); d.a.lawlor@bristol.ac.uk (D.A.L.); 2 MRC Integrative Epidemiology Unit at the University of Bristol, Bristol BS8 2PS, UK; 3 Bradford Institute for Health Research, Bradford Teaching Hospitals NHS Foundation Trust, Bradford BD9 6RJ, UK; jane.west@bthft.nhs.uk (J.W.); tiffany.yang@bthft.nhs.uk (T.Y.); 4 Translational Science, Bristol Medical School, Bristol BS2 8DZ, UK; M.Caputo@bristol.ac.uk; 5 Bristol NIHR Biomedical Research Center, Bristol BS1 2NT, UK

**Keywords:** pregnancy, ethnicity, serum, metabolomics, cardiometabolic profile, birth cohort, Born in Bradford

## Abstract

There is widespread metabolic disruption in women upon becoming pregnant. South Asians (SA) compared to White Europeans (WE) have more fat mass and are more insulin-resistant at a given body mass index (BMI). Whether these are reflected in other gestational metabolomic differences is unclear. Our aim was to compare gestational metabolic profiles and their determinants between WE and SA women. We used data from a United Kingdom (UK) cohort to compare metabolic profiles and associations of maternal age, education, parity, height, BMI, tricep skinfold thickness, gestational diabetes (GD), pre-eclampsia, and gestational hypertension with 156 metabolic measurements in WE (*n* = 4072) and SA (*n* = 4702) women. Metabolic profiles, measured in fasting serum taken between 26–28 weeks gestation, were quantified by nuclear magnetic resonance. Distributions of most metabolic measures differed by ethnicity. WE women had higher levels of most lipoprotein subclasses, cholesterol, glycerides and phospholipids, monosaturated fatty acids, and creatinine but lower levels of glucose, linoleic acid, omega-6 and polyunsaturated fatty acids, and most amino acids. Higher BMI and having GD were associated with higher levels of several lipoprotein subclasses, triglycerides, and other metabolites, mostly with stronger associations in WEs. We have shown differences in gestational metabolic profiles between WE and SA women and demonstrated that associations of exposures with these metabolites differ by ethnicity.

## 1. Introduction

Pregnancy is associated with widespread metabolic changes which are required to meet the demands of the developing fetus [1]. These changes are more marked in obese pregnant women, and, to some extent, they are modifiable by a lifestyle intervention in obese pregnant populations [2]. With the advent of high-throughput metabolomics, large-scale epidemiological studies identified a number of characteristics that relate to differences in metabolic profiles in non-pregnant women and men [3]. These include age [4], body mass index (BMI) [5,6], physical activity [7], alcohol consumption [8], vitamin D [9], menopausal age and stage [4], pubertal timing [10] and the use of hormonal contraception [11], statins, and proprotein convertase subtilisin/kexin type 9 (PCSK9) inhibitors [12,13]. For several of these exposures, longitudinal, genetic, and/or randomized trial evidence suggests causal effects. Less is known about pregnancy characteristics that might influence differences in pregnancy metabolic profiles. Differences in serum, plasma, or urinary metabolic profiles in relation to pre-pregnancy BMI, gestational diabetes (GD), or pre-eclampsia (PE) were reported, but studies were small (mostly with fewer than 50 cases) [14,15,16,17,18,19,20,21,22,23,24], or were in select populations, for example, overweight or obese pregnant women [25].

For a given BMI, South Asian (SA) compared with White European (WE) adults have greater fat mass and are more insulin-resistant. This led to the hypothesis that they have a thin-fat insulin-resistant phenotype, which increases their risk of type 2 diabetes and coronary heart disease [26,27,28]. Recent evidence, including from the Born in Bradford (BiB) study, suggests that this phenotype is present in childhood [29,30] and at birth [31,32,33]. These differences are also present in pregnant women, with SA women in BiB having higher gestational fasting and post-load glucose and risk of GD compared with WE women, despite their lower BMI [34]. It is plausible that there are more widespread pregnancy metabolomic differences between WE and SA women during pregnancy, but, to our knowledge, this is yet to be explored. Exploring ethnic differences in gestational metabolic profiles and the impact of exposures during pregnancy on metabolism could improve our understanding of the etiology of ethnic differences in pregnancy and offspring outcomes.

The aims of this study were to (1) compare metabolic profiles (measured on one occasion between 26–28 weeks gestation) between WE and SA pregnant women, and (2) compare the magnitude and direction of associations of maternal age, education, parity, height, BMI, tricep skinfold thickness (TST), GD, PE, and gestational hypertension (GHT) with metabolic profiles between WE and SA women.

## 2. Results

### 2.1. Participant Characteristics

The BiB cohort was used in this study and was described in detail elsewhere [35]. Of the 12,453 women (13,776 pregnancies) originally recruited to BiB, 8774 women were included in the main analyses presented here (4072 WE and 4702 SA women). Figure S3 (Supplementary Materials) illustrates the flow of women into eligible analysis groups. Table 1 shows the distributions of maternal characteristics during pregnancy for all women, stratified by ethnicity. On average, early-pregnancy BMI was greater in WE than in SA women. TST was also larger in WE than SA women; however, many women declined to have these measured, with a higher proportion of SA having missing data (Table 1 and Tables S1–S3, Supplementary Materials). The prevalence of GD in SA women was double that in WE women. The prevalence of PE was similar in the two ethnic groups, and SA women were half as likely to have GHT.

### 2.2. Ethnic Differences in Pregnancy Metabolic Profiles

**Figure 1:**
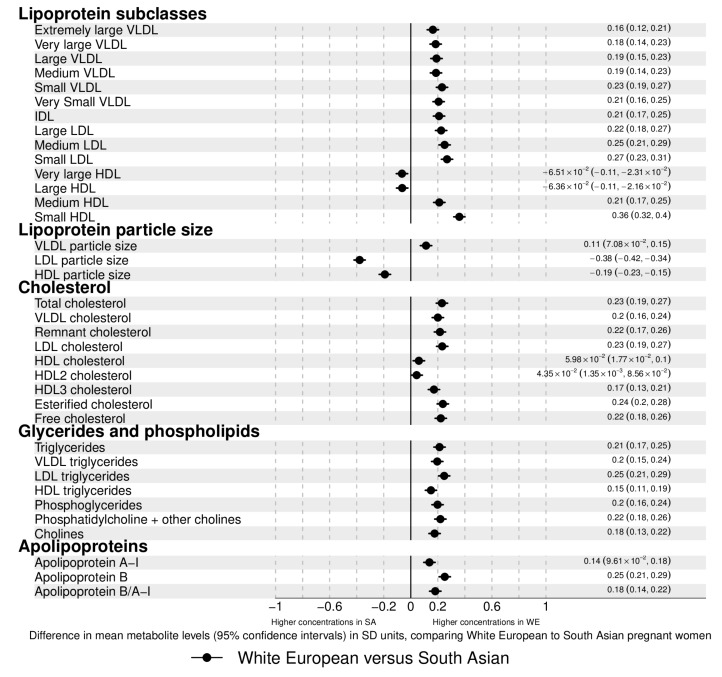

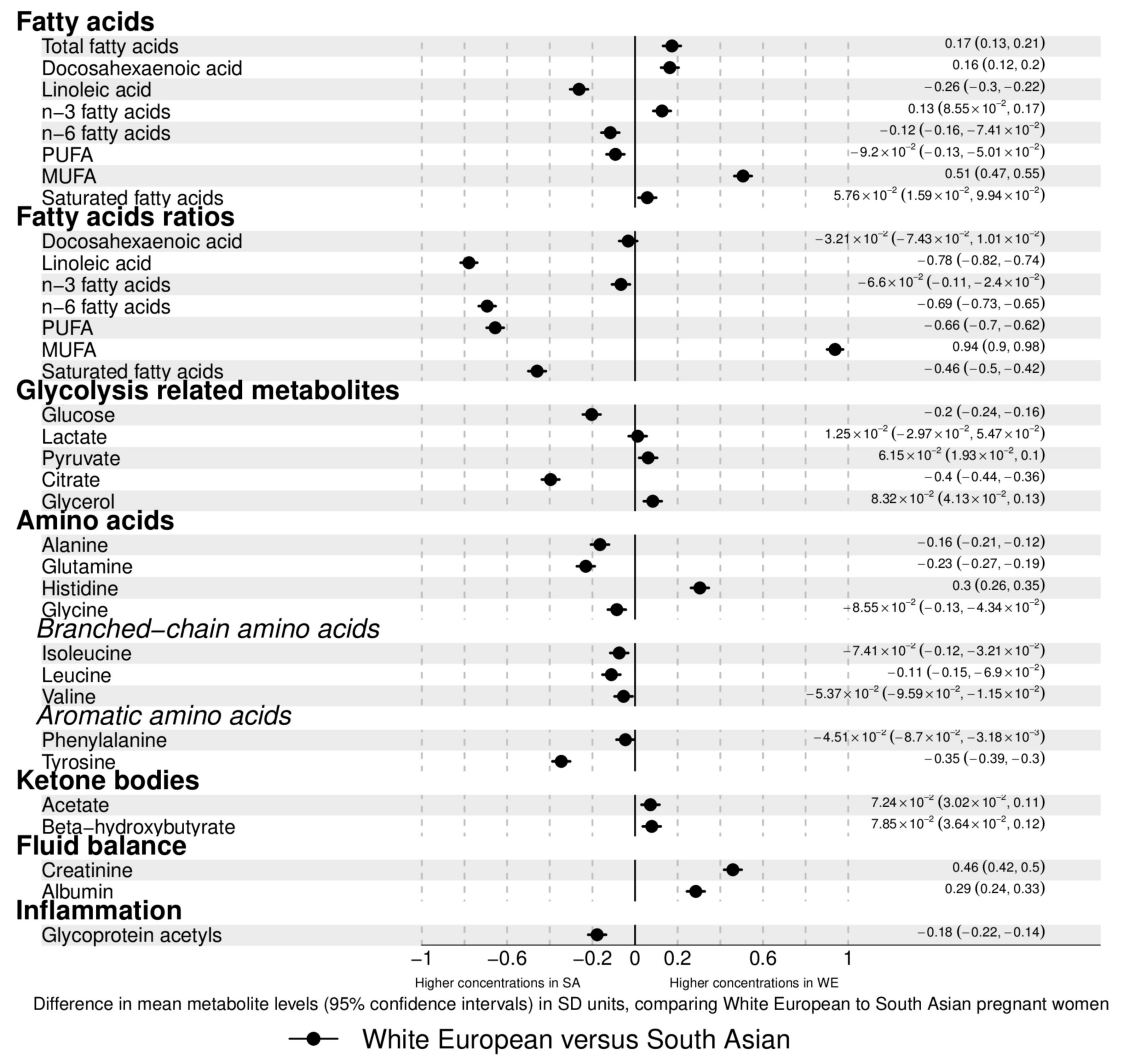
Associations between ethnicity and metabolic profiles. The associations are differences in mean metabolite concentrations/values in standard deviation (SD) units comparing White European to South Asian women. Point estimates to the left of the null are higher in South Asian (SA) with point estimates to the right higher in White European (WE) women. Error bars are 95% confidence intervals (CI). Point estimates and their corresponding 95% CIs are displayed in text to the right of each point. Differences displayed in original concentration units can be found in Table S4 (Supplementary Materials). The NMR platform quantifies all individual fatty acids within each of the 4 main classes: omega-3 polyunsaturated fatty acids (PUFAs), omega-6 PUFA, monounsaturated fatty acids (MUFAs), and saturated fatty acids (SFAs). In addition to that, two individual fatty acids are quantified: docosahexaenoic acid (DHA), an omega-3 PUFA, and linoleic acid, an omega-6 PUFA. Abbreviations: VLDL, very low-density lipoprotein; LDL, low-density lipoprotein; HDL, high-density lipoprotein; C, cholesterol.

There were ethnic differences in the distributions of most metabolites. Concentrations of most lipoprotein subclasses, cholesterols, glycerides, phospholipids, and most fatty acids (FA) were higher in WE compared to SA pregnant women. The magnitudes of difference were ~0.1–0.3 SD, although stronger (0.5 SD) for monounsaturated FAs (MUFA) (Figure 1). Very large and large high-density lipoprotein (HDL) particles, linoleic, omega-6, and polyunsaturated FA (PUFA) concentrations were higher in SA (magnitudes <0.1–0.2 SD). When examined as the percentage of total FAs, results differed. For omega-3 FAs and saturated FAs (SFAs), percentages of total FA levels were higher in SA women (whereas concentrations in original units were higher in WEs). For some of the other FAs, the magnitude of differences was larger between the ethnic groups. Glucose (0.2 SD), and citrate (0.4 SD) were higher in SA women, whereas pyruvate and glycerol concentrations were higher in WE (both <0.1 SD), and lactate concentrations were similar in the two groups. Apart from histidine, which had higher concentrations in WE, all amino-acid concentrations were higher in SA women, with differences being particularly large for tyrosine, alanine, and glutamine. Glycoprotein acetyl (GlycA) concentrations, a stable marker of cumulative inflammation, were higher in SA women.

### 2.3. Associations of Age, Education, and Parity with Gestational Metabolic Profiles in White European and South Asian Women

Age-adjusted associations of age (unadjusted), education, and parity with metabolic profiles in WE and SA women are shown in Figures S4–S6 (Supplementary Materials). These figures also show where there is statistical support for differences in association between SA and WE. Differences in original concentration units for all associations are also shown in Tables S4–S10 (Supplementary Materials). Maternal age was positively associated with most metabolites in both ethnic groups. The magnitudes of association with very low-density lipoprotein (VLDL) subclasses and citrate were greater in SA, whereas medium, large, and very large HDL subclasses, HDL-C, Apolipoprotein A1 (ApoA1), docosahexaenoic acid (DHA), omega-3 FAs, and acetate were greater in WE women (Figure S4, Supplementary Materials). In WE women, age-adjusted higher education was negatively associated with VLDL lipoproteins and cholesterol, remnant cholesterol, triglycerides, Apolipoprotein B (ApoB), total FAs, MUFAs, pyruvate, lactate, and GlycA. There were positive associations of age-adjusted education with ApoA1, HDL subclasses, all FA ratios except for MUFAs, valine, tyrosine, and creatinine. In SA women, these associations were close to the null or were weak and in the opposite direction to those seen for WE women (Figure S5, Supplementary Materials). In WE women who experienced at least one previous pregnancy, compared with those who were experiencing their first pregnancy, mean levels of VLDL subclasses and triglycerides, medium and small LDLs, MUFAs, and GlycA were higher. Mean levels of HDLs, DHA, and omega-3 FAs were lower. In SA women, associations of parity with these metabolites were close to the null (Figure S6, Supplementary Materials). In both ethnic groups, having at least one previous pregnancy was negatively associated with lactate, pyruvate, several amino acids, and albumin with stronger magnitudes of association seen in SA women.

### 2.4. Associations of Height, BMI, and Tricep Skinfold Thickness with Gestational Metabolic Profiles in White European and South Asian Women

**Figure 2:**
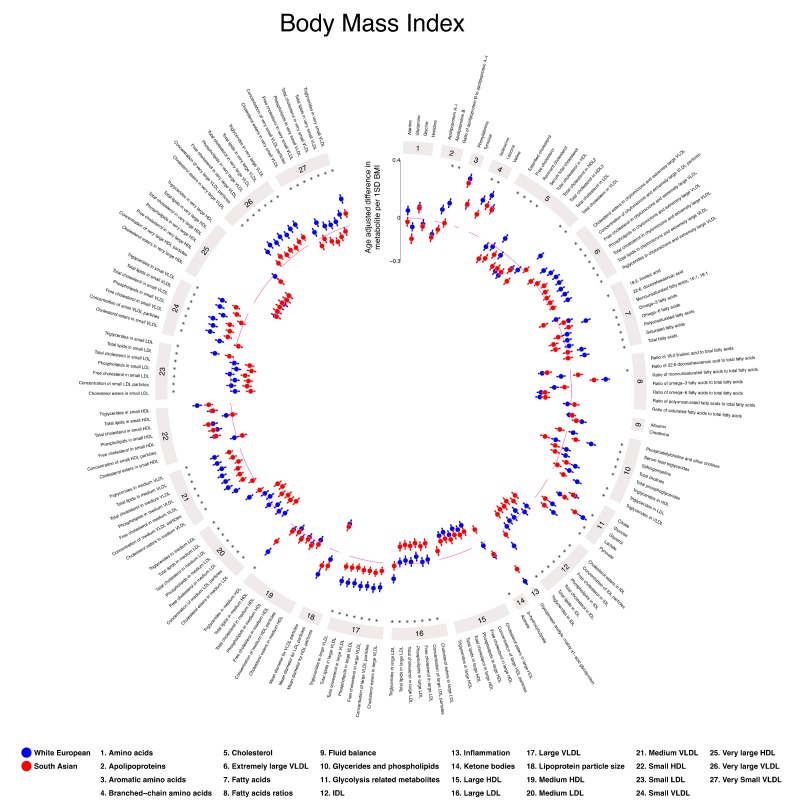
Age-adjusted associations of maternal body mass index (BMI) with pregnancy metabolic profiles stratified by ethnicity. Data points show SD differences per 1 SD higher BMI for White European (blue) and South Asian (red) women. Error bars = 95% CIs. * denotes strong statistical evidence from the interaction test (*p*_interaction_ < 0.001). Detailed figures with differences in means and 95% CIs (Figure S8) and differences in original concentration units (Table S9) can be found in Supplementary Materials. Abbreviations: VLDL, very low-density lipoprotein; LDL, low-density lipoprotein; HDL, high-density lipoprotein; C, cholesterol; MUFA, monounsaturated fatty acids; PUFA, polyunsaturated fatty acids. This plot was constructed using MR Vis (http://bristol-medical-stat.bristol.ac.uk:3838/MR-Vis/).

Overall, age-adjusted associations of height with metabolites were weak (≤0.06 SD metabolite for a 1 SD greater height) and similar in SA and WE. Results were similar after additional adjustment for education and parity (Figures S7, S13 and S16 and Table S8, Supplementary Materials). Age-adjusted BMI was positively associated with VLDL lipoprotein subclasses and triglycerides, MUFAs, all glycolysis-related metabolites, amino acids, beta-hydroxybutyrate, creatinine, and GlycA in both WE and SA women (Figure 2). The magnitude of these associations was greater in WE women than SA, except for glucose which was stronger in SA women. BMI was negatively associated with total cholesterol, with stronger associations seen in SA women. It was positively associated with VLDL cholesterol, with stronger associations in WE women. BMI was positively associated with remnant cholesterol in WE women and negatively associated with it in SA women. An opposite direction of association is also observed for the percentage of total fatty acids that was DHA, with a negative association in WEs and positive association in SAs. Results were similar when further adjusted for parity and education in addition to age (Figures S8, S13 and S16 and Table S9, Supplementary Materials).

The patterns of associations for TST were broadly similar to those seen for BMI, but with wider confidence intervals and no strong statistical support for differences between ethnic group (Figure S9 and Table S10, Supplementary Materials).

### 2.5. Associations of Gestational Diabetes, Pre-Eclampsia, and Gestational Hypertension with Gestational Metabolic Profiles in White European and South Asian Women

**Figure 3:**
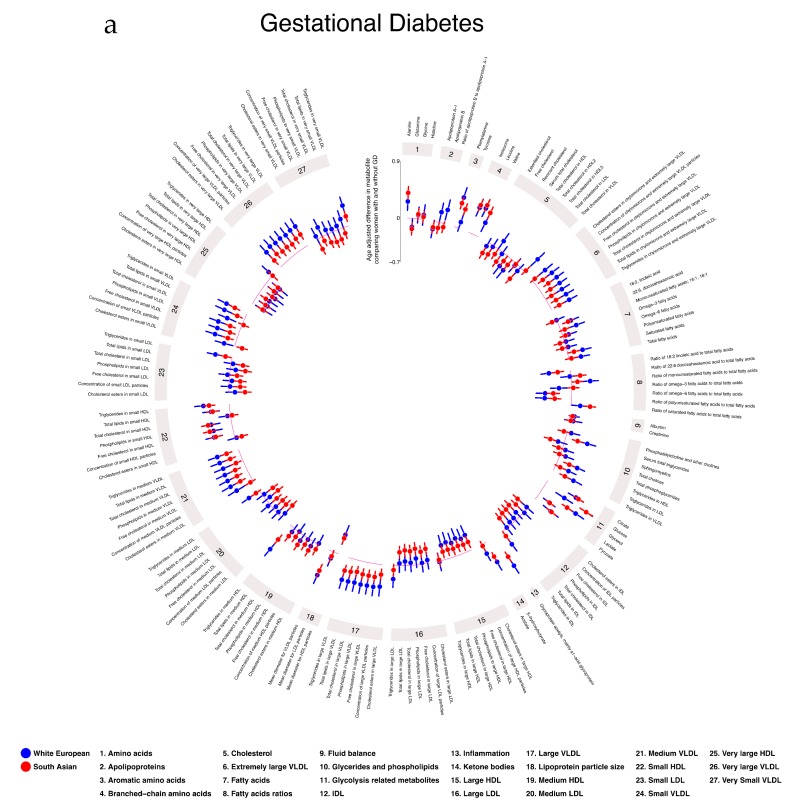

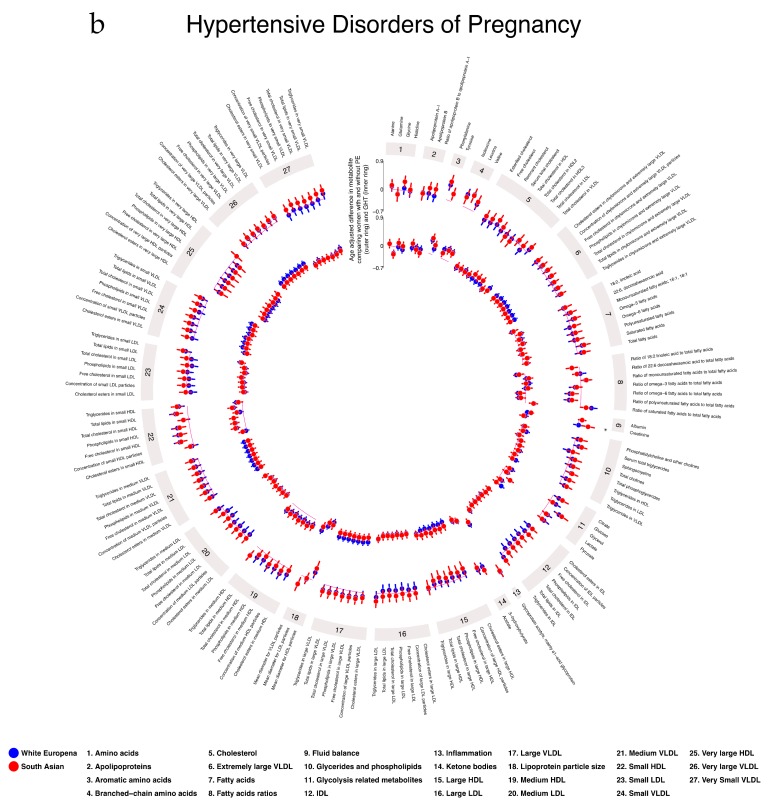
Age-adjusted associations of gestational diabetes (3**a**), pre-eclampsia (3**b**; outer ring), and gestational hypertension (3**b**; inner ring) with pregnancy metabolic profiles stratified by ethnicity. Data points show SD differences for White European (blue) and South Asian (red) women between having a pregnancy disorder or not. Error bars = 95% CIs. * denotes strong statistical evidence from the interaction test (*p*_interaction_ < 0.001). Detailed figures with differences in means and 95% CIs (Figures S10–S12) and differences in original concentration units (Tables S11–S13) can be found in Supplementary Materials. Abbreviations: VLDL, very low-density lipoprotein; LDL, low-density lipoprotein; HDL, high-density lipoprotein; C, cholesterol; MUFA, monounsaturated fatty acids; PUFA, polyunsaturated fatty acids. These plots were constructed using MR Vis (http://bristol-medical-stat.bristol.ac.uk:3838/MR-Vis/).

In age-adjusted analyses, GD was positively associated with VLDL lipoproteins, VLDL cholesterol, remnant cholesterol, most glycerides and phospholipids, all glycolysis-related metabolites, several amino acids, ketone bodies, and GlycA. GD was negatively associated with LDL and HDL subclasses, FA ratios, and glutamine in both WE and SA, with the magnitudes of association being largely consistent in the two ethnic groups (Figure 3a). There was some evidence of ethnic differences in the associations of GD with FAs measured in original units (not ratios) with positive associations in WE women and negative or null associations in SA women. As expected, GD was positively associated with glucose in both ethnic groups with a stronger association in SA. With further adjustment for education and parity, results were similar (Figures S10, S13 and S16 and Table S11, Supplementary Materials). With additional adjustment for BMI, associations of GD with all metabolites slightly attenuated toward the null in both ethnic groups (Figures S10, S15 and S18 and Table S11, Supplementary Materials).

In age-adjusted analyses, PE was positively associated with VLDL lipoprotein subclasses, glycerides and phospholipids, FAs, glycolysis-related metabolites, most amino acids, creatinine, and GlycA (Figure 3b). The magnitude of associations was greater in SA for most of these associations. PE was negatively associated with FA ratios, DHA, linoleic, omega-3, omega-6, PUFA, and SFA with these associations stronger in WE women. With further adjustment for education and parity, results were similar (Figures S11, S14 and S17 and Table S12, Supplementary Materials). With additional adjustment for BMI, associations attenuated toward the null, with the extent of attenuation being greater in WE women (Figures S11, S15 and S18 and Table S12, Supplementary Materials).

The overall pattern of associations of GHT with metabolites was similar to those seen for PE, but with some differences in the ethnic disparities (Figure 3b). For example, whereas the positive associations of PE with VLDL lipoproteins, triglycerides, and FAs were stronger in SA, the positive associations of GHT with these were stronger in WE. However, there was no strong statistical evidence of any ethnic differences in associations of GHT with metabolites. With further adjustment for education and parity, results were similar (Figures S12, S14 and S17 and Table S13, Supplementary Materials). With additional adjustment for BMI, associations for both ethnic groups attenuated toward the null (Figures S12, S15 and S18 and Table S13, Supplementary Materials).

### 2.6. Additional Analyses

We conducted two separate additional analyses. To check whether missing data (described in Table 1 and under the ‘dealing with missing data’ section of methods) influenced results, we repeated the analyses using complete case data (women who had no missing metabolite or pregnancy exposure data). We found similar associations in these results compared with our main analyses (linear fit for all models: *R^2^* ≥ 0.96; Figures S19–S28, Supplementary Materials). We repeated the analyses only including White British and Pakistani mothers, the two largest most homogeneous groups, and found very similar results to those with the broader WE and SA comparisons (linear fit for all models: *R*^2^ ≥ 0.96; Figures S29–S38, Supplementary Materials).

## 3. Discussion

We report novel differences in pregnancy NMR-derived metabolic profiles between WE and SA women. We also demonstrated how pregnancy characteristics are related to metabolic profiles and that the direction and magnitude of the relationship can differ by ethnicity. Overall, WE women had higher levels of lipoproteins and cholesterol, but lower levels of several FAs, amino acids, and glucose than SA women. After adjustment for potential confounders, we found evidence that the relationship between several pregnancy characteristics and metabolic profiles differs by ethnicity. This was particularly the case for maternal age, education, and BMI. For education and parity, many of the ethnic differences in associations were qualitative (i.e., in different directions). For example, the associations of education with remnant cholesterol and many triglycerides were negative in WE women but positive in SA women, whereas education was positively associated with glucose, valine, and tyrosine in WEs but negatively associated with these outcomes in SAs. By contrast, most associations of BMI, GD, PE, and GHT were quantitative (in the same direction in both ethnic groups but stronger in magnitude in one group compared with the other).

A previous large (*n* > 4000) study compared metabolic profiles between pregnant and non-pregnant women and noted marked differences [1], and, in a randomized controlled trial of pregnant women with obesity (*n* > 1000), a lifestyle intervention was found to effectively reduce some changes in FAs and triglycerides in these women [2]. Using that same randomized trial as a cohort, marked differences in metabolic profiles in relation to GD were demonstrated [25]. However, despite these previous studies including mixed ethnic populations, to our knowledge, none specifically analyzed ethnic differences. Several studies explored ethnic differences in standard clinical chemistry lipid measurements during pregnancy [36,37,38,39], although the ethnic groups being explored were different to our study and the sample sizes were smaller (*n* = 232–3254), thereby making direct comparisons with our results difficult. These studies consistently found that African/Afro-Caribbean women had a more favorable lipid profile in pregnancy and that this could be related to reductions in adverse pregnancy outcomes [36,40]. In the present study, we showed that WE pregnant women have higher levels of lipoproteins and cholesterols than SA women. A derangement of the maternal lipid profile during pregnancy was previously linked with multiple adverse pregnancy [41] and offspring outcomes, including congenital heart disease [42]. Whether the ethnic differences in the lipid profile presented in our study influence these outcomes requires further research. Unsurprisingly, we found SA women to have higher glucose levels than WE women, corresponding to their almost two-fold increase in GD prevalence, despite them having a lower average BMI. We also demonstrate that SA women had higher levels of FA ratios, most amino acids, and GlycA. GlycA is a common indicator of inflammation which was previously associated with an increased risk of infection, higher levels of inflammatory cytokines [43], and all-cause mortality [44].

The amino acids isoleucine, leucine, valine, tyrosine, and phenylalanine were previously associated with future risk of type 2 diabetes [45,46], and amino-acid risk scores were previously shown to predict cardiovascular disease in healthy individuals [47]. Thus, these differences in pregnant women may reflect general differences between SA and WE populations and go some way to explaining the greater risk of type 2 diabetes and cardiovascular disease in SAs [26,27,28]. Given evidence for a thin-fat insulin-resistant phenotype already being present in SA infants at birth [31,32,33], it is also possible that ethnic differences in gestational metabolites are important in the developmental origins of differences in type 2 diabetes and cardiovascular diseases between SAs and WEs.

Consistent with a previous study in obese pregnant women, we found that GD was positively associated with lipids and lipoprotein constituents in VLDL subclasses and several amino acids [25]. We extended these findings by showing that the association of GD with lipids and lipoproteins was greater in WE compared with SA women. A similar pattern was also observed for maternal BMI and TST in that magnitudes of association with most metabolites were greater in WE women, but adjustment for BMI did not notably alter ethnic differences in the associations of GD with lipids and lipoproteins. Taking these results, together with those from previous research in BiB [32,33,34], we showed that SA women have less adiposity but have higher fasting and post-load glucose and risk of GD compared with WE women. Whilst the association of BMI with gestational glucose is stronger in SA women, its association with lipids and lipoproteins are weaker. Thus, the higher cord-leptin and insulin concentrations and relative greater fatness in SA infants at birth may be mostly driven by higher maternal glucose, with possibly little influence of other metabolites. Consistent with our findings, PE was shown to be associated with lipids and amino acids and also with carnitines (which are not measured in our platform) [21]. Adjustment for BMI attenuated associations of PE with lipids in our study but had little impact on amino-acid associations. A rare complication of pregnancy is cholestasis of pregnancy, which was recently linked to non-alcoholic fatty liver disease (NAFLD) [48]. We previously showed that, in a general (non-pregnant) population of adolescents, NAFLD is associated with adverse lipid and FA profiles, as well as an inflammatory marker from the same NMR platform used here [49]. Whilst some of these associations are similar to those that we see for pregnancy complications in this paper, as pregnancy cholestasis is rare, we think it is unlikely that our results reflect pregnancy-specific hepatic problems.

Amino acids regulate key metabolic pathways; therefore, disturbances in their regulation could have profound effects on the offspring during and after pregnancy [50]. We demonstrated that maternal BMI, GD, PE, and GHT are all associated with differences in pregnancy amino-acid concentrations, and it is possible that these mediate some of the relationships of these complications with adverse pregnancy and perinatal outcomes and with later offspring health outcomes. Further research using a variety of appropriate causal approaches to test mediation is necessary to explore this further [51].

It is unclear why we find qualitative differences between WE and SA women in the associations of education and parity with multiple metabolites. These measures may reflect very different things in the two groups. For example, over 30% of the SA women were educated in Pakistan, India, or Bangladesh. Whilst we used an established method to convert educational qualifications from those countries to the equivalent of United Kingdom (UK) exams, it is possible that education is measuring something different in the two groups because of inaccuracies in the conversion of SA qualifications to English qualifications. There are also potential differences in the social and cultural meaning of education between the two populations. Similarly, as the SA women in BiB have higher parity and there are cultural differences in the desirability for large families between SA and WE, the simple binary of first pregnancy vs. any previous pregnancy used here may reflect different phenomena in the two groups. These possibilities require further exploration which is beyond the scope of this paper.

Our study has several strengths. To our knowledge, we are not aware of any study with a similar or larger sample size with detailed prospectively collected phenotypic and metabolomic measurements during pregnancy with an almost 50% ethnic split. We wrote and used a pre-specified analysis plan which can be found in the reference list [52]. In our study, all women received the same antenatal care and resided in the same UK city, meaning that the ethnic differences that we found cannot be due to geographical differences or differences in antenatal care. We undertook additional analyses to explore the potential impact of missing data on our results and whether restricting analyses to the two largest homogeneous ethnic groups changed them. Both analyses produced similar results to the main analyses, suggesting that missing data may not have biased our findings and that results may be generalizable to broadly defined SA and WE populations residing in the UK.

A key limitation of our study is not having the ability to replicate the results in an independent cohort. To the best of our knowledge, there are no cohorts with large numbers of SA and WE women with metabolomic data measured in pregnancy. However, we acknowledge that, without replication, our results should be treated with some caution. Furthermore, there are pregnancy-related changes in the levels of many metabolic traits [1]. As the patterns of change are similar by baseline (early pregnancy/pre-pregnancy), this within-person variation in levels over time would only bias our results if samples were collected across a wide range of gestational ages and there were systematic differences in the sampling time for different groups. This is not the case here, as samples were collected within a narrow gestational age range and the distribution of gestational ages of samples collected was the same in SA and WE women. Our objective was to compare SA to WE women given the evidence that SA populations, in comparison to WE populations, have a thin-fat insulin-resistant phenotype that is present at birth, and the possibility that differences between these two groups in pregnancy metabolic profiles may contribute to this phenotype. However, we acknowledge that it would also be valuable to make similar comparisons across other ethnic groups, which we were not able to do. Our results cannot be generalized to other ethnic populations. We were not able to explore associations of physical activity and diet which are likely to influence metabolites [53,54] and differ between ethnic groups. Whilst diet and physical activity cannot determine ethnicity or socioeconomic position and, therefore, could not confound their associations with metabolites, for some of the results, there may be residual confounding due to failure to adjust for these. This is likely to be the case for associations of GD, PE, and GHT with metabolites. However, some of this confounding may have been controlled for by adjusting for education and BMI, which influence diet and physical activity or are influenced by it. Next, although the NMR platform has advantages in that it is relatively stable and does not have substantial batch effects [3], it only covers a small amount of the human metabolome and, thus, does not have the scope to identify all possible differences that may arise between SA and WE women. We are unable to provide raw spectral data as these are not made available by Nightingale Health© due to protection of their intellectual property in relation to the quantification. Whilst we showed high correlations between glucose, total cholesterol, HDLc, LDLc, and triglycerides measured by this NMR platform and measured using standard clinical chemistry methods, we are not able to explore this for all of the 156 NMR measurements, as most of these are not available as standard clinical chemistry practice.

This study provides novel insight into ethnic differences in pregnancy metabolic profiles and how differences in pregnancy characteristics associate with these. Evidence shows differences between SA and WE in adiposity and cardiometabolic health that are present at birth and with pregnancy outcomes, such as small for gestational age and congenital anomalies. The mechanisms underlying these differences are unclear, but the ethnic differences in metabolic profiles observed in this study may play a mechanistic role.

## 4. Materials and Methods

### 4.1. Participants

We used data from the BiB study, a population-based prospective birth cohort including 12,453 women across 13,776 pregnancies. Full details of the study methodology were reported previously [35]. In brief, most women were recruited at their oral glucose tolerance test (OGTT) at approximately 26–28 weeks gestation, which is offered to all women booked for delivery at Bradford Royal Infirmary. Eligible women had an expected delivery between March 2007 and December 2010. Approximately half of the births are to mothers of SA origin. Ethical approval for the study was granted by the Bradford National Health Service Research Ethics Committee (ref 06/Q1202/48), and all participants gave written informed consent.

Figure S1 (Supplementary Materials) illustrates the flow of participants through the study. For our analyses, women had a fasting pregnancy serum sample which was used for NMR metabolome profiling, as well as valid pregnancy exposure data. We further restricted the cohort to women of WE and SA origin, as numbers from other ethnic backgrounds were too small to be analyzed. Following these exclusions, there were 8774 women included in the main analysis.

### 4.2. Assessment of Ethnicity

Ethnicity was self-reported by the mother at her recruitment questionnaire interview and based on the UK Office of National Statistics guidance, details of which were previously reported [55]. For women who did not have ethnicity data collected at the recruitment interview, data were abstracted from primary care medical records, which use a similar categorization. Women classified as SA included those who indicated they were Pakistani (*n* for present study = 4139, 88%), Indian (*n* = 362, 8%), or Bangladeshi (*n* = 201, 4%). Women classified as WE included those who indicated that they were White British (*n* = 3827, 94%) or other White European (*n* = 245, 6%) origin.

### 4.3. Maternal Pregnancy Measurements

For our analyses, we used the following pregnancy measures as exposures: age, height, BMI, education, parity, GD, GHT, PE, and TST. Age was obtained for all women at pregnancy booking. Height was measured at recruitment (26–28 weeks gestation) using a Leicester Height Measure (*Seca,* London, UK). Maternal BMI was calculated using the height measured at recruitment and weight measured at first antenatal clinic visit (approximately 12 weeks gestation), and it was extracted from medical records. All women were recruited from the same hospital which used *Seca* two-in-one scales (Harlow Healthcare Ltd., London, UK) to measure weight. Information on maternal education was obtained at the recruitment interview. We equivalized the mother’s highest educational qualifications (based on the qualification received and the country obtained) into one of several categories using the UK National Recognition Information Center (NARIC) (https://www.naric.org.uk/naric/): *<5 GCSE equivalent, ≥5 GCSE equivalent, “A-level” equivalent, higher than A-level equivalent, other qualifications (e.g., City and Guilds, RSA/OCR, BTEC), unknown, foreign unknown.* Unknown relates to the mother responding “do not know” during the interview. Foreign unknown relates to a qualification listed in the free text response, but no level of qualification is given, or the qualification listed cannot be equivalized to one of the above categories. For this study, maternal education was dichotomized into women that were educated to below A-level (*<5 GCSE equivalent, ≥5 GCSE equivalent, other)* and those educated to A-level or above (*A-level equivalent, higher than A-level equivalent)*. Unknown and foreign unknown were not categorized. Data on parity and all measures of blood pressure and proteinuria were abstracted from medical records. Parity was categorized as having one or more previous pregnancies (multiparous (yes)) or no previous pregnancy (nulliparous (no)). GHT was defined as new onset of elevated blood pressure after 20 weeks gestation where systolic blood pressure was 140 mmHg or greater, and/or diastolic blood pressure was 90 mmHg or greater on two or more occasions. PE was defined as GHT plus proteinuria 1+ or greater. All women booked for delivery in Bradford are offered a 75-g oral glucose tolerance test comprising fasting and 2-h post-load samples at around 26–28 weeks gestation. GD was defined according to modified World Health Organization criteria operating at the time of the study as either fasting glucose ≥ 6.1 mmol/L or 2-h post-load glucose ≥ 7.8 mmol/L.

The TST measurement was taken specifically for the BiB project at baseline (26–28 weeks). The rationale for exploring associations of TST with metabolites is because it is suggested that skinfold thickness measurements provide an estimate of pregnancy fat mass that is not influenced by fetal growth [56]. Measurements were obtained using Tanner/Whitehouse Calipers (Holtain Ltd., Crymych, Wales) by specially trained BiB study administrators according to a written protocol and always on the left side of the body. Equipment was recalibrated every 12 months. As many women refused to undress for this measurement, there are substantial (64%) amounts of missing data and we explored how select those with these measurements were likely to be by comparing characteristics between those women with a TST measurement and those without (Table S2 and S3, Supplementary Materials). SA women were less likely to have TST measures (80% missing vs. 46% in WEs). Within WE women, distributions of characteristics were similar between those with and without TST, whereas there were some differences in SA women. Specifically, those SA women with a TST measurement had a higher mean BMI, greater prevalence of GD, and were more educated, and they were more likely to have a previous pregnancy compared to those without a TST measurement (Table S3, Supplementary Materials). In those with measurements, TST was correlated with BMI (*r* = 0.66 overall; *r* = 0.68 in WE and *r* = 0.61 in SA).

### 4.4. Maternal Pregnancy Metabolic Profiling using the NMR Platform

#### 4.4.1. Sample Collection and Storage

Of the 13,776 pregnancies in the BiB cohort, 11,476 had a fasting serum sample taken at the same time (*n* = 10,574 (92% between 26–28 weeks gestation, with the remaining being within 11–39 weeks). Samples were taken by trained phlebotomists working in the antenatal clinic of the Bradford Royal Infirmary and sent immediately to the hospital laboratory. All samples were processed within 2.5 h and then placed in −80 °C freezers. There were no freeze–thaw events of the samples used for the NMR metabolic profiling. Further details on the laboratory processing procedures used for the maternal samples can be found in Text S1 (Supplementary Materials).

#### 4.4.2. NMR Protocol

Profiling of maternal circulating lipids, fatty acids, and metabolites was done by a high-throughput targeted NMR platform (Nightingale Health© (Helsinki, Finland)) at the University of Bristol, deriving quantitative molecular information on 156 metabolic traits. The NMR-based metabolite quantification is achieved through measurements of three molecular windows from each serum sample. Two of the spectra (LIPO and LMWM windows) are acquired from native serum and one spectrum from serum lipid extracts (LIPID window). The NMR spectra are measured using Bruker AVANCE III spectrometer operating at 600 MHz. Measurements of native serum samples and serum lipid extracts are conducted at 37 °C and 22 °C, respectively. The NMR platform was previously applied in various large-scale epidemiological studies [5,57,58].

#### 4.4.3. Metabolite Quantification and Quality Control

The NMR spectra were analyzed for metabolite quantification (molar concentration) in an automated fashion. For each metabolite, a ridge regression model was applied for quantification in order to overcome the problems of heavily overlapping spectral data. In the case of the lipoprotein lipid data, quantification models were calibrated using high-performance liquid chromatography methods, and individually cross-validated against NMR-independent lipid data. Low-molecular-weight metabolites, as well as lipid extract measures, were quantified as mmol/L based on regression modeling calibrated against a set of manually fitted metabolite measures. The calibration data are quantified based on iterative line-shape fitting analysis using PERCH NMR software (PERCH Solutions Ltd., Kuopio, Finland). Quantification could not be directly established for the lipid extract measures due to experimental variation in the lipid extraction protocol. Therefore, serum extract metabolites were scaled via the total cholesterol as quantified from the native serum LIPO spectrum.

#### 4.4.4. Validation of the NMR Platform

Previous work compared concentrations of standard lipids and glucose from the same samples assessed by clinical chemistry methods and the NMR platform and showed high levels of correlation for total cholesterol, LDLc, HDLc, triglycerides, and glucose in samples from adult White Europeans [3]. We performed similar analyses in BiB pregnancy samples. Figure S2 (Supplementary Materials) shows scatter plots and correlation coefficients comparing fasting glucose, total cholesterol, HDLc, LDLc, and triglycerides measured by the NMR platform to those measured using standard clinical chemistry (standard Lipid Research Clinics Protocol using enzymatic reagents and glucose oxidase method). Correlation coefficients were high for all five measures (from 0.73 for glucose to 0.93 for triglycerides). The intercepts of the scatter plots were close to zero for HDLc, LDLc, and triglycerides, but were higher for glucose (1.85 mmol/L) and total cholesterol (1.21 mmol/L), suggesting that, for these, the NMR platform systematically underestimates levels. However, the high levels of correlation mean that association analyses should be accurate.

### 4.5. Statistical Analyses

All analyses were conducted using R version 3.4.2 (R Foundation for Statistical Computing, Vienna, Austria). An analysis plan was written by K.T., D.L.S.F., and D.A.L. in February 2019 [52]. Multivariable linear regression was used to examine the associations of maternal pregnancy characteristics with pregnancy serum metabolic profiles. All models described below were run separately in WE and SA women. Robust standard errors were estimated for all associations, as some metabolite concentrations had skewed distributions. In these analyses, age, height, BMI, and TST were all analyzed in standard deviation units, with the standard deviations summarized in the whole cohort so that they had the same value in both ethnic groups and were not ethnic-specific. Ethnicity, education, parity, GD, GHT, and PE were treated as binary variables. Differences in the magnitude or direction of associations were explored by looking at the ethnic-specific point estimates and by adding an interaction term between ethnicity and the exposures to explore statistical evidence of a difference. We applied a threshold of *p*_interaction_ < 0.001 as statistical evidence of an interaction. For each multivariable regression model, potential confounders were decided a priori. We also examined age-adjusted associations of ethnicity with metabolites (whilst age could not influence ethnicity and, therefore, not confound its association with metabolites, the relation of age with metabolites means that adjusting for it might improve statistical efficiency). We explored the associations of nine exposures with metabolic profiles. Model 1 was the same for all exposures, but other models varied by exposure, as follows:

- Model 1: age-adjusted (except for association of age with metabolic profiles). Rationale—maternal age is known to influence metabolic profiles. Also, age can influence education, parity, height, BMI, TST, GD, GHT, and PE and, thus, could confound the association of any of these exposures with metabolic profiles.
- Model 2: for exposures height, BMI, TST, GD, GHT, and PE only, we adjusted for education and parity (in addition to age), as these could influence these exposures and metabolic profiles and, hence, could be confounders.
- Model 3: for GD, GHT, and PE only, we adjusted as for model 2 but additionally adjusted for BMI, as this might confound the association of these with metabolites.

#### Dealing with Missing Data

A small number of women had missing data on one or a small number of metabolites, the largest being citrate where 230 women did not have a measure (<3%). Different exposure data had different levels of missingness which varied from 0% for ethnicity, age, and GD to TST which had 64% (see Table 1 for the full breakdown of missing data). In the main analyses, for each individual model, we included the maximal number of women (i.e., in the age-adjusted models, the number of women included would be higher than where we adjusted for BMI because we included those women even if they had missing BMI). This may mean that, when we compared two models (model 1 and model 3), any differences may have been due to the adjustment or to chance differences in different subsamples. To explore this, we re-ran all the adjusted models in women that had no missing data (complete case) and compared these to the main analyses, except for TST, which we did not include as a confounding factor.

### 4.6. Additional Analyses

We conducted two separate additional analyses. Firstly, as mentioned above, we repeated our analyses including only complete case data to test whether any missing data were altering the results. For the second additional analysis, we only included White British and Pakistani women instead of WE and SA. The rationale for this was to test whether including ethnic groups where sample sizes were relatively small (Indian, Bangladeshi, other White European) influenced the results.

## Figures and Tables

**Table 1:** Distributions of maternal characteristics during pregnancy by ethnicity.

Maternal Characteristics	Category	All (*n* = 8774)	White European (*n* = 4072)	South Asian (*n* = 4702)	Diff in Means or OR (95% CI) *
**Age, years**		27.3 ± 5.6	26.7 ± 6.0	27.8 ± 5.2	1.1 (0.8, 1.3)
**Height (cm)**		161.7 ± (6.4)	164.2 ± 6.2	159.5 ± 5.8	4.7 (4.5, 5.0)
Missing (%)		172 (2.0)	57 (1.4)	115 (2.4)	-
**BMI (kg/m^2^)**		26.1 ± 5.7	26.7 (6.0)	25.6 ± 5.4	1.1 (0.9, 1.4)
Missing (%)		413 (4.7)	183 (4.5)	230 (4.9)	-
**TST (mm)**		25.4 ± 7.1	25.7 ± 7.2	24.6 (6.9)	1.1 (0.6, 1.6)
Missing (%) ^a^		5671 (64.6)	1891 (46.4)	3780 (80.4)	-
**Education**	Below A-level	5151 (58.7)	2462 (60.5)	2689 (57.2)	Ref
	A-level or above	3446 (39.3)	1523 (37.4)	1923 (40.9)	1.2 (1.1, 1.3)
Unknown/Missing (%)		177 (2.0)	87 (2.1)	90 (1.9)	-
**HDP**	Normotensive	7902 (90.1)	3533 (86.8)	4369 (92.9)	Ref
	PE	224 (2.6)	118 (2.9)	106 (2.3)	0.7 (0.6, 0.9)
	GHT	634 (7.2)	417 (10.2)	217 (4.6)	0.4 (0.3, 0.5)
Missing (%)		14 (0.2)	4 (0.1)	10 (0.2)	-
**Gestational Diabetes**	Yes	734 (8.4)	209 (5.1)	525 (11.2)	2.3 (1.9, 2.7)
**Parity**	Median (IQR)	1 (0–2)	1 (0–1)	1 (0–2)	-
	Nulliparous	3433 (39.1)	1938 (47.6)	1495 (31.8)	Ref
	Multiparous	5037 (57.4)	2000 (49.1)	3037 (64.6)	2.0 (1.8, 2.1)
Missing (%)		304 (3.5)	134 (3.3)	170 (3.6)	-
**Gest Age at Blood Sampling (weeks)**		26.3 (2.0)	26.2 (1.9)	26.3 (2.0)	0.0 (−0.1, 0.0)

Diff, difference; OR, odds ratio; CI, confidence interval; BMI, body mass index; TST, tricep skinfold thickness; HDP, hypertensive disorders of pregnancy; PE, pre-eclampsia; GHT, gestational hypertension; Gest, gestational; Ref, reference range. Data are means ± SD or *n* (%) unless stated. For characteristics with no “missing” category, data were 100% complete. ^a^ A large proportion of women had missing data for tricep skinfold thickness, and this was more marked for South Asian women. Further analyses (Tables S1–S3, Supplementary Materials) show differences in characteristics between those women who did and did not have skinfold thickness measurements. * Difference in mean calculated for continuous variables and odds ratio calculated for categorical variables; an odds ratio >1 indicates a higher exposure rate in South Asian women.

## Supplementary Materials

The following are available online at https://www.mdpi.com/2218-1989/9/9/190/s1: Text S1, Born in Bradford laboratory processing of maternal samples; Text S2; Nuclear magnetic resonance spectroscopy methods; Figure S1, Stages and methods used for NMR platform metabolic measures (adapted from Wurtz et al.); Figure S2, Comparison of lipoprotein lipids and glucose quantification using nuclear magnetic resonance (NMR) (*x*-axis) and routine clinical chemistry assays (*y*-axis) (*N* = 11,026 to 11,337). *R* = correlation coefficient; Table S1, Distributions of maternal characteristics during pregnancy by ethnicity for the sub-cohort of women who had a tricep skinfold thickness measurement; Table S2, Distributions of maternal characteristics during pregnancy for White European women stratified by whether they had a tricep skinfold measurement or not; Table S3, Distributions of maternal characteristics during pregnancy for South Asian women stratified by whether they had a tricep skinfold measurement or not; FigureS3, Study flow chart; Figure S4, Associations of maternal age with pregnancy metabolic profiles stratified by ethnicity; Figure S5, Age-adjusted associations of maternal education with pregnancy metabolic profiles stratified by ethnicity; Figure S6, Age-adjusted associations of maternal parity with pregnancy metabolic profiles stratified by ethnicity; Figure S7, Associations of maternal height with pregnancy metabolic profiles stratified by ethnicity (model 1 = age-adjusted; model 2 = age-, parity-, and education-adjusted); Figure S8, Associations of maternal body mass index with pregnancy metabolic profiles stratified by ethnicity (model 1 = age-adjusted; model 2 = age-, parity-, and education-adjusted); Figure S9, Associations of tricep skinfold thickness with pregnancy metabolic profiles stratified by ethnicity (model 1 = age-adjusted; model 2 = age-, parity-, and education-adjusted); Figure S10, Associations of maternal gestational diabetes with pregnancy metabolic profiles stratified by ethnicity (model 1 = age-adjusted; model 2 = age-, parity-, and education-adjusted, model 3 = age-, parity-, education-, and BMI-adjusted); Figure S11, Associations of maternal pre-eclampsia with pregnancy metabolic profiles stratified by ethnicity (model 1 = age-adjusted; model 2 = age-, parity-, and education-adjusted, model 3 = age-, parity-, education-, and BMI-adjusted); Figure S12, Associations of maternal gestational hypertension with pregnancy metabolic profiles stratified by ethnicity (model 1 = age-adjusted; model 2 = age-, parity-, and education-adjusted, model 3 = age-, parity-, education-, and BMI-adjusted); Figures S13–S18, Linear fit between regression models; Figures S19–S28, Sensitivity Analysis 1: Linear fit between missing data and complete case analyses; Figures S29–S38, Sensitivity Analysis 2: Linear fit between White European and White British women, and South Asian and Pakistani women; Table S4, Pregnancy mean metabolite concentrations in original units for White European and South Asian women; Table S5, Pregnancy metabolite concentration differences in original units per 1 SD higher maternal age; Table S6, Pregnancy metabolite concentration differences in original units between being educated to A-level or above vs. not. Results are-adjusted for maternal age; Table S7, Pregnancy metabolite concentration differences in original units between multiparous vs. nulliparous. Results are-adjusted for maternal age; Table S8, Pregnancy metabolite concentration differences in original units per 1 SD higher maternal height. Model 1: age-adjusted, model 2: age-, parity-, and education-adjusted; Table S9, Pregnancy metabolite concentration differences in original units per 1 SD higher maternal body mass index. Model 1: age-adjusted, model 2: age-, parity-, and education-adjusted; Table S10, Pregnancy metabolite concentration differences in original units 1 SD higher tricep skinfold measurement. Model 1: age-adjusted, model 2: age-, parity-, and education-adjusted; Table S11, Pregnancy metabolite concentration differences in original units between mothers with gestational diabetes vs. not. Model 1: age-adjusted, model 2: age-, parity-, and education-adjusted, model 3: age-, parity-, education-, and BMI-adjusted; Table S12, Pregnancy metabolite concentration differences in original units between mothers with pre-eclampsia vs. not. Model 1: age-adjusted, model 2: age-, parity-, and education-adjusted, model 3: age-, parity-, education-, and BMI-adjusted; Table S13, Pregnancy metabolite concentration differences in original units between mothers with gestational hypertension vs. not. Model 1: age-adjusted, model 2: age-, parity-, and education-adjusted, model 3: age-, parity-, education-, and BMI-adjusted.
