# Supplementary material for: Differences in Pregnancy Metabolic Profiles and Their Determinants between White European and South Asian Women: Findings from the Born in Bradford Cohort
Source: Metabolites. 2019 Sep 18;9(9):190. doi: 10.3390/metabo9090190 (PMC6780545; doi:10.3390/metabo9090190)
Supplement: Supplementary file 1 [file metabolites-09-00190-s001.zip › Supplementary File 1.pdf]

Differences in pregnancy metabolic profiles and their determinants between White European and South Asian women: Findings from the Born in Bradford Cohort

Supplementary File 1:

Contents:

Pages 1-4: Supplementary text and methods

Pages 5-7: Information in relation to the tricep skinfold measurement

Page 8: Study flowchart

Pages 9-53: Forest plots from regression models exploring associations of maternal exposures with pregnancy metabolic profiles

Pages 54-59: Correlation plots assessing agreement between regression models by linear fit (Figures S13-S18)

Pages 60-69: Sensitivity analysis 1: testing whether missing data is influencing results (Figures S19-S28)

Pages 70-79: Sensitivity analysis 2: testing whether ethnic groups with fewer women are influencing results (Figures S29-S38)

Supplementary text and methods

Text S1. Born in Bradford laboratory processing of maternal serum samples.

Isolation of serum prior to storage

Venous blood was collected in GEL tubes to obtain serum. The following processing steps were undertaken prior to storage of serum at -80OC

1) Storage racks were prepared.
2) Participant details were checked, making sure that both the BiB study ID and Hospital number on the specimen bottles matched those on the tracking forms.
3) Tubes were centrifuged at 3500 rpm for 10 minutes at room temperature.
4) A 1 ml automatic pipette was used to aliquot serum into 2 x 1.5 ml vials.
5) Vials were labelled with appropriate BiB study labels and placing the duplicate barcode label in corresponding space marked on BiB tracking form.
6) Serum aliquots (n = 3) were them placed in ranks in a -80 °C freezer.

Text S2. Nuclear Magnetic Resonance (NMR) Spectroscopy Methods

The NMR spectroscopy methodology is summarised in Figure S1 below and the metabolites that were quantified, together with the units in which they are quantified, are shown in Supplementary File 2. This NMR-based metabolite quantification is achieved through measurements of three molecular windows from each serum sample. Two of the spectra (LIPO and LMWM windows) are acquired from native serum and one spectrum from serum lipid extracts (LIPID window). The NMR spectra are measured using Bruker AVANCE III spectrometer operating at 500 or 600 MHz. Measurements of native serum samples and serum lipid extracts are conducted at 37oC and 22OC, respectively.

The LIPO window represents a standard spectrum of human serum displaying broad overlapping resonances arising from lipid molecules in various lipoprotein particles. The LIPO data are recorded using 8 transients acquired using a NOESY-presat pulse sequence with mixing time of 10ms and water peak suppression. The LMWM window includes signals from various low-molecular-weight molecules. The LMWM spectrum is recorded using a relaxation-filtered pulse sequence that suppresses most of the broad macromolecule and lipid signals to enhance detection of small solutes. Specifically, a Carr-Purcell-Meiboom-Gill (CPMG) pulse sequence with a 78ms T2-filter and fixed echo delay of 403μs is applied using 24 transients. The LIPID window of the serum extracts is acquired with a standard 1D spectrum using 32 transients.

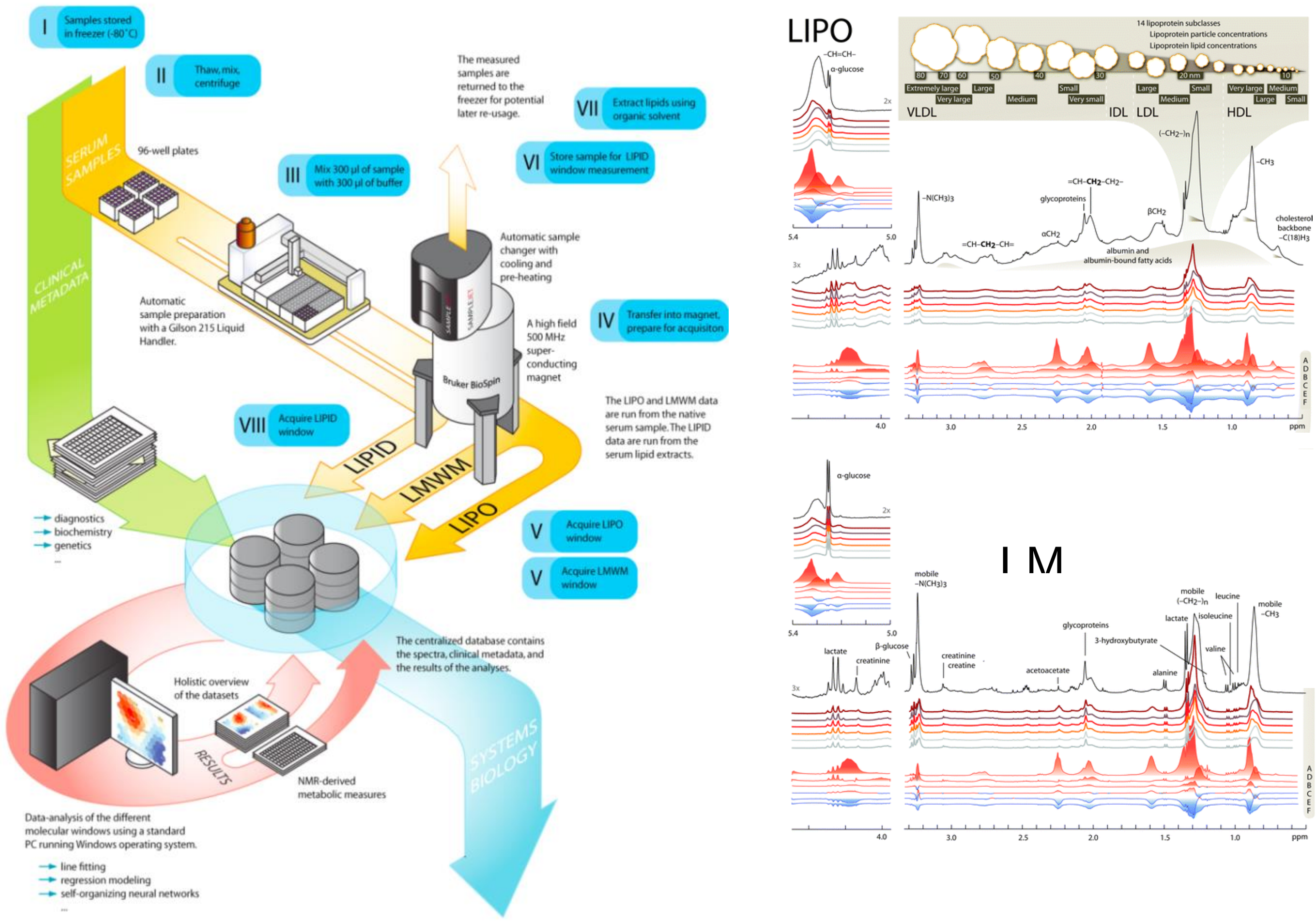

Figure S1. Stages and methods used for NMR platform metabolic measures (adapted from Wurtz et al.)

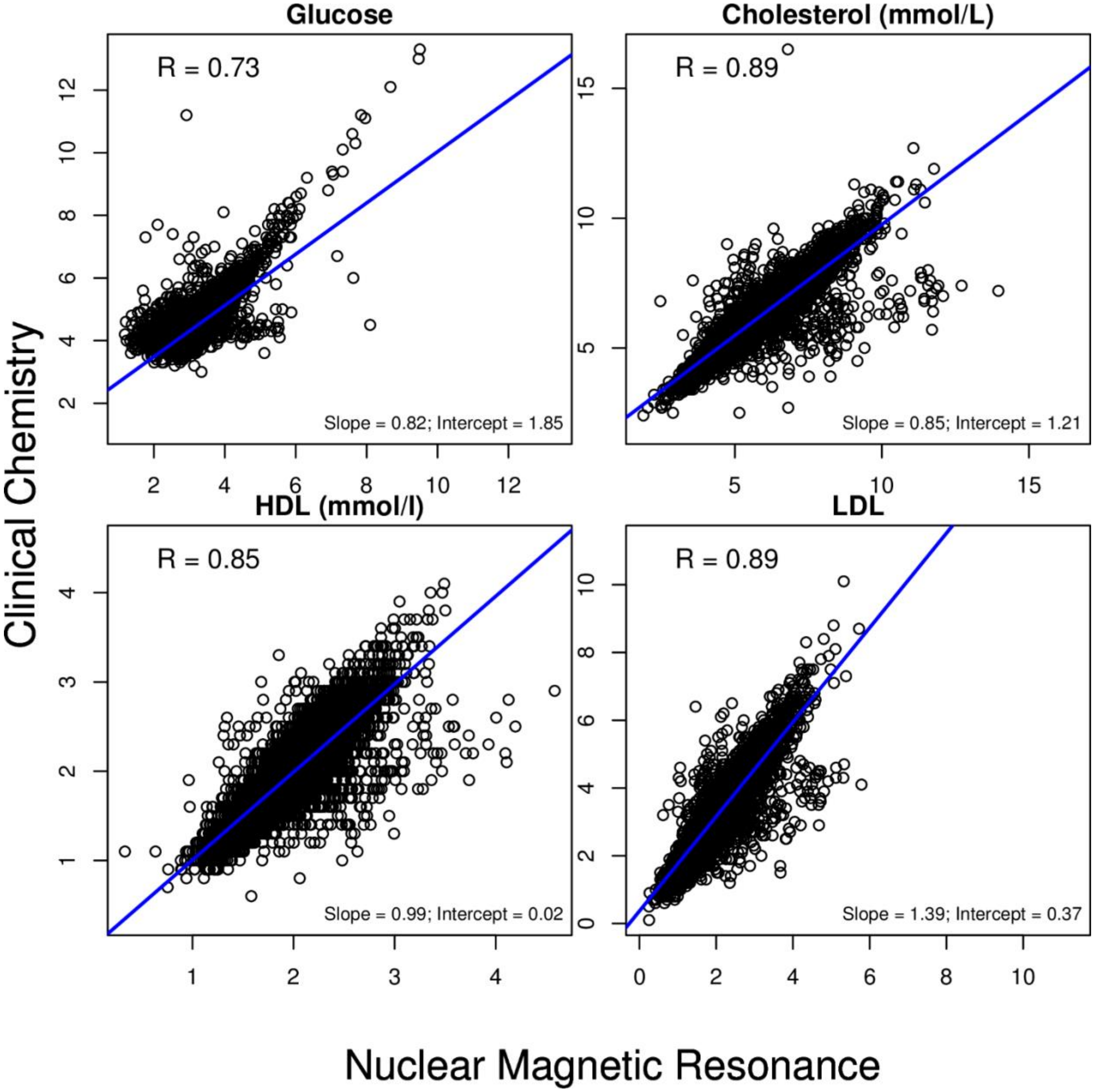
Glucose
R = 0.73
Slope = 0.82; Intercept = 1.85
Cholesterol (mmol/L)
R = 0.89
Slope = 0.85; Intercept = 1.21
HDL (mmol/l)
R = 0.85
Slope = 0.99; Intercept = 0.02
LDL
R = 0.89
Slope = 1.39; Intercept = 0.37
Clinical Chemistry
Nuclear Magnetic Resonance

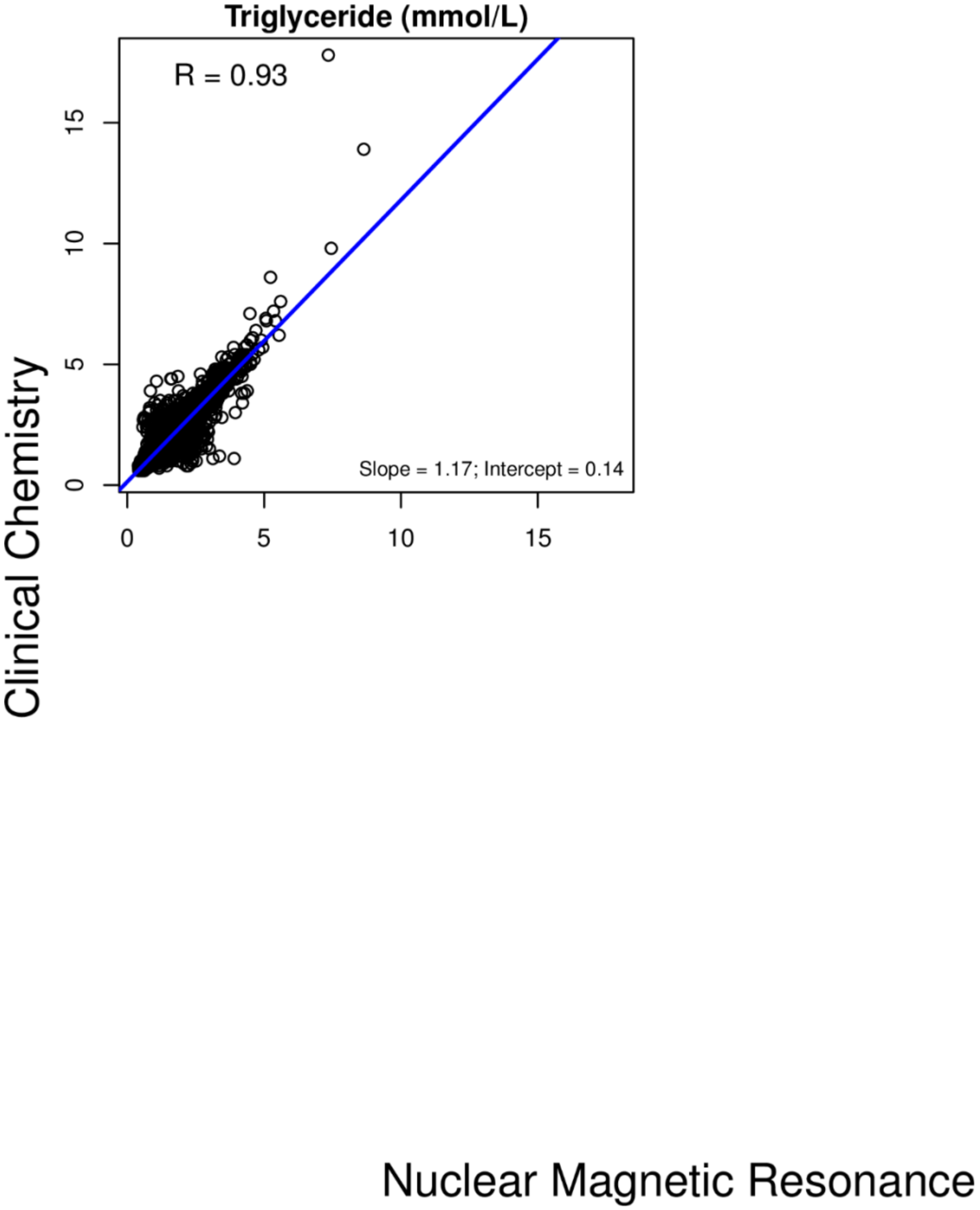

Figure S2. Comparison of lipoprotein lipids and glucose quantification using nuclear magnetic resonance (NMR) (x-axis) and routine clinical chemistry assays (y-axis) (N= 11,026 to 11,337). R= correlation coefficient.

Table S1. Distributions of maternal characteristics during pregnancy by ethnicity for the sub cohort of women who had a tricep skinfold thickness measurement

Maternal characteristics		All (n = 3,103)	White European (n = 2,181)	South Asian (n = 922)	Diff in means or OR (95% CI)*
**TST, mm**		25.4 ± 7.1	25.7 ± 7.2	24.6 ± 6.9	1.1 (0.5, 1.6)
**Age, years**		27.0 ± 5.8	26.8 ± 6.0	27.5 ± 5.0	0.7 (0.3, 1.1)
**Height, cm**		162.9 ± 6.4	164.3 ± 6.1	159.5 ± 5.7	4.8 (4.3, 5.2)
**BMI, kg/m2**		25.9 ± 5.5	26.6 ± 5.6	24.4 ± 4.7	2.2 (1.8, 2.6)
**Education**	Below A-level	1794 (58.70	1321 (61.5)	473 (52.0)	Ref
	A-level or above	1262 (41.3)	826 (38.5)	436 (48.0)	1.5 (1.3, 1.7)
**HDP**	Normotensive	2762 (89.0)	1887 (86.5)	875 (94.9)	Ref
	PE	80 (2.6)	65 (3.0)	15 (1.6)	0.5 (0.3, 0.9)
	GHT	258 (8.3)	226 (10.4)	32 (3.5)	0.3 (0.2, 0.4)
**Gestational diabetes**	Yes	169 (5.4)	98 (4.5)	71 (7.7)	1.8 (1.3, 2.4)
**Parity**	Nulliparous	1370 (46.4)	1030 (49.3)	340 (39.5)	Ref
	Multiparous	1581 (53.6)	1060 (50.7)	521 (60.5)	1.5 (1.3, 1.7)
**Gest age at blood sampling, weeks**		26.3	26.3	26.3	0

BMI, body mass index. TST, tricep skinfold thickness; Diff, difference; OR, odds ratio; Gest, gestational. Data are means ± SD or *n* (%). *Difference in mean calculated for continuous variables and odds ratio calculated for categorical variables: an odds ratio >1 indicates a higher exposure rate in South Asian women.

Table S2. Distributions of maternal characteristics during pregnancy for White European women stratified by whether they had a tricep skinfold measurement or not

Maternal characteristics		Women with TST measurement (n = 2,181)	Women without TST measurement (n = 1,891)	Diff in means or OR (95% CI)*
**Age, years**		26.8 ± 6.0	26.6 ± 6.0	0.2 (-0.2, 0.6)
**Height, cm**		164.3 ± 6.1	164.1 ± 6.3	0.2 (-0.2, 0.5)
**BMI, kg/m2**		26.6 ± 5.6	26.8 ± 6.4	0.2 (-0.2, 0.4)
**Education**	Below A-level	1321 (61.5)	1141 (62.1)	Ref
	A-level or above	826 (38.5)	697 (37.9)	1.0 (0.9, 1.1)
**HDP**	Normotensive	1887 (86.5)	1646 (87.0)	Ref
	PE	65 (3.0)	53 (2.8)	0.9 (0.6, 1.4)
	GHT	226 (10.4)	191 (10.1)	1.0 (0.8, 1.2)
**Gestational diabetes**	Yes	98 (4.5)	111 (5.9)	1.3 (1.0, 1.7)
**Parity**	Nulliparous	1030 (49.3)	908 (49.1)	Ref
	Multiparous	1060 (50.7)	940 (50.9)	1.0 (0.9, 1.1)
**Gest age at blood sampling, weeks**		26.3	26.2	0.1 (-0.7, 0.15)

BMI, body mass index. TST, tricep skinfold thickness; Diff, difference; OR, odds ratio; Gest, gestational. Data are means ± SD or *n* (%). *Difference in mean calculated for continuous variables and odds ratio calculated for categorical variables: an odds ratio >1 indicates a higher exposure rate in women without measurement.

Table S3. Distributions of maternal characteristics during pregnancy for South Asian women stratified by whether they had a tricep skinfold measurement or not

Maternal characteristics		Women with TST measurement (n = 922)	Women without TST measurement (n = 3,780)	Diff in means or OR (95% CI)*
**Age, years**		27.5 ± 5.0	27.9 ± 5.2	0.4 (0.0, 0.8)
**Height, cm**		159.5 ± 5.7	159.5 ± 5.8	0
**BMI, kg/m2**		24.4 ± 4.7	25.9 ± 5.5	1.5 (1.2, 1.9)
**Education**	Below A-level	473 (52.0)	2216 (59.8)	Ref
	A-level or above	436 (48.0)	1487 (40.2)	0.7 (0.6, 0.8)
**HDP**	Normotensive	875 (94.9)	3494 (92.4)	Ref
	PE	15 (1.6)	91 (2.4)	1.5 (0.9, 2.6)
	GHT	32 (3.5)	185 (4.9)	1.4 (0.9, 2.1)
**Gestational diabetes**	Yes	71 (7.7)	454 (12.0)	1.6 (1.3, 2.1)
**Parity**	Nulliparous	340 (39.5)	1155 (31.5)	Ref
	Multiparous	521 (60.5)	2516 (68.5)	1.4 (1.2, 1.7)
**Gest age at blood sampling, weeks**		26.3	26.3	0

BMI, body mass index. TST, tricep skinfold thickness; Diff, difference; OR, odds ratio; Gest, gestational. Data are means ± SD or *n* (%). *Difference in mean calculated for continuous variables and odds ratio calculated for categorical variables: an odds ratio >1 indicates a higher exposure rate in women without measurement.

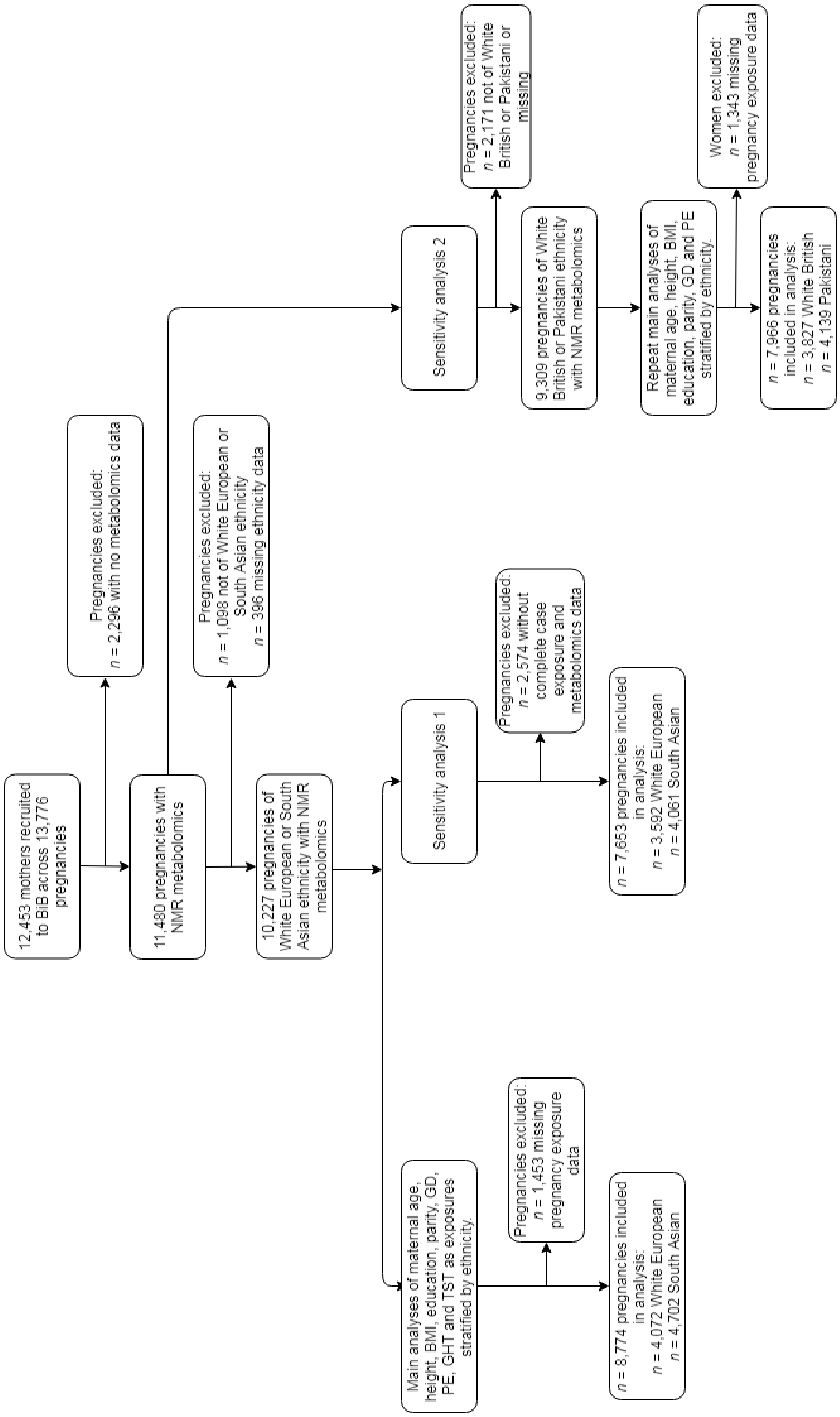

Figure S3. Study flow chart

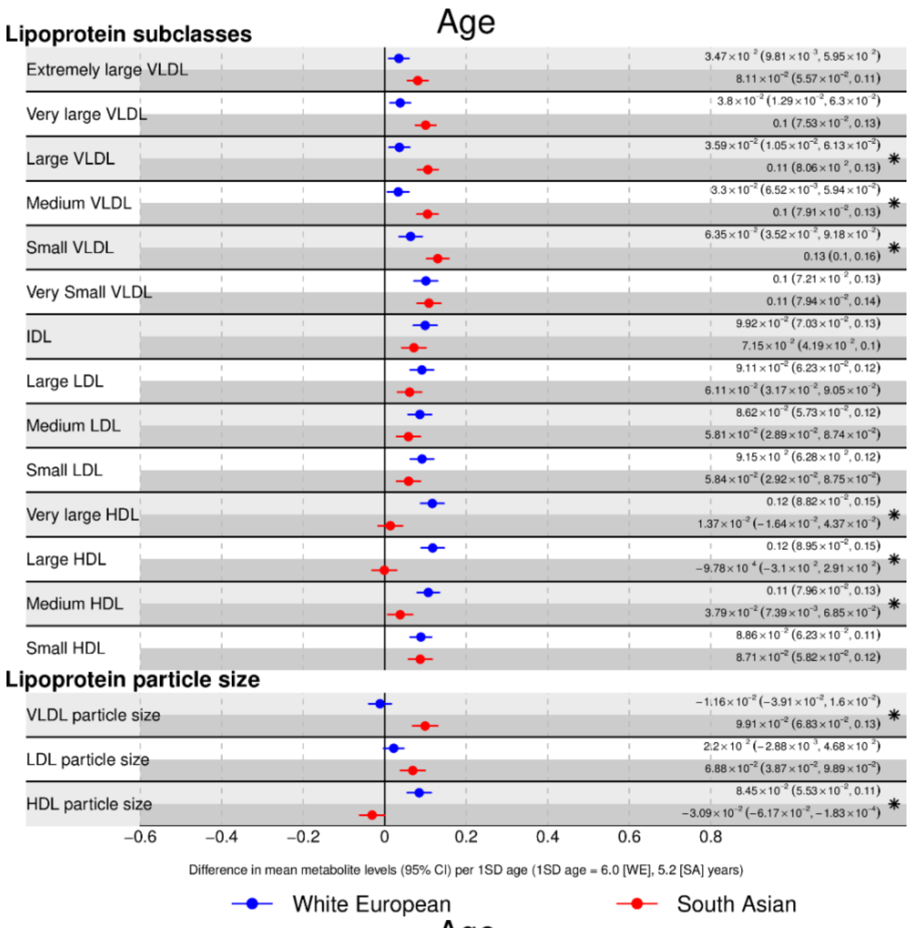
Age
Lipoprotein subclasses
Extremely large VLDL
Very large VLDL
Large VLDL
Medium VLDL
Small VLDL
Very Small VLDL
IDL
Large LDL
Medium LDL
Small LDL
Very large HDL
Large HDL
Medium HDL
Small HDL
Lipoprotein particle size
VLDL particle size
LDL particle size
HDL particle size
−0.6 −0.4 −0.2 0 0.2 0.4 0.6 0.8
Difference in mean metabolite levels (95% CI) per 1SD age (1SD age = 6.0 [WE], 5.2 [SA] years)
White European
South Asian

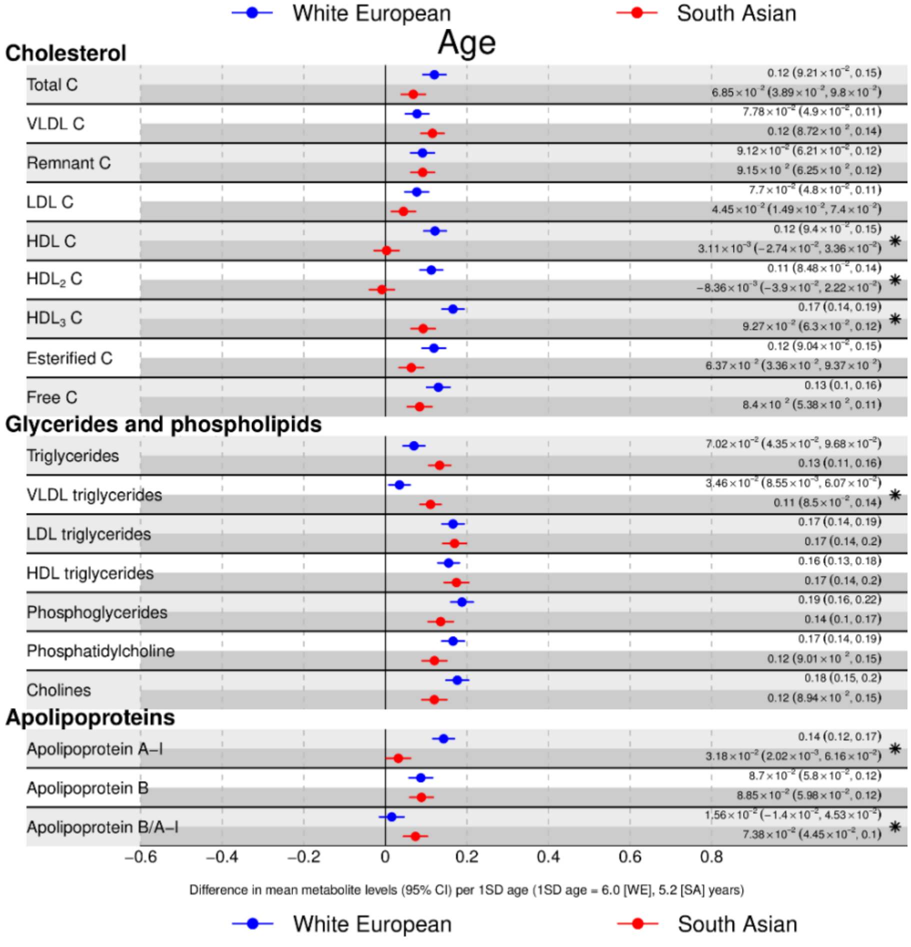
Age
Cholesterol
Total C
VLDL C
Remnant C
LDL C
HDL C
HDL2 C
HDL3 C
Esterified C
Free C
Glycerides and phospholipids
Triglycerides
VLDL triglycerides
LDL triglycerides
HDL triglycerides
Phosphoglycerides
Phosphatidylcholine
Cholines
Apolipoproteins
Apolipoprotein A–I
Apolipoprotein B
Apolipoprotein B/A–I
−0.6 −0.4 −0.2 0 0.2 0.4 0.6 0.8
Difference in mean metabolite levels (95% CI) per 1SD age (1SD age = 6.0 [WE], 5.2 [SA] years)
White European
South Asian

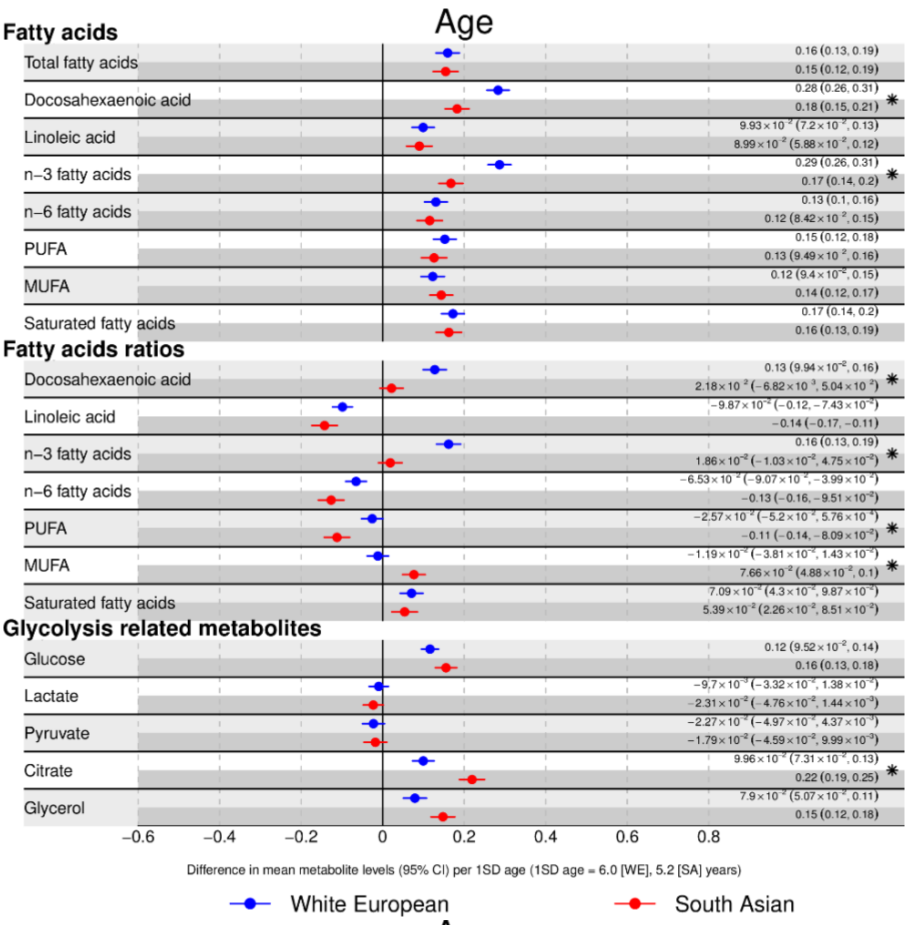
Age
Fatty acids
Total fatty acids
0.16 (0.13, 0.19)
0.15 (0.12, 0.19)
Docosahexaenoic acid
0.28 (0.26, 0.31)
0.18 (0.15, 0.21) *
Linoleic acid
9.93×10^{-2} (7.2×10^{-2}, 0.13)
8.99×10^{-2} (5.88×10^{-2}, 0.12)
n−3 fatty acids
0.29 (0.26, 0.31)
0.17 (0.14, 0.2) *
n−6 fatty acids
0.13 (0.1, 0.16)
0.12 (8.42×10^{-2}, 0.15)
PUFA
0.15 (0.12, 0.18)
0.13 (9.49×10^{-2}, 0.16)
MUFA
0.12 (9.4×10^{-2}, 0.15)
0.14 (0.12, 0.17)
Saturated fatty acids
0.17 (0.14, 0.2)
0.16 (0.13, 0.19)
Fatty acids ratios
Docosahexaenoic acid
0.13 (9.94×10^{-2}, 0.16)
2.18×10^{-2} (-6.82×10^{-3}, 5.04×10^{-2}) *
Linoleic acid
-9.87×10^{-2} (−0.12, -7.43×10^{-2})
−0.14 (−0.17, −0.11)
n−3 fatty acids
0.16 (0.13, 0.19)
1.86×10^{-2} (-1.03×10^{-2}, 4.75×10^{-2}) *
n−6 fatty acids
-6.53×10^{-2} (-9.07×10^{-2}, -3.99×10^{-2})
−0.13 (−0.16, -9.51×10^{-2})
PUFA
-2.57×10^{-2} (-5.2×10^{-2}, 5.76×10^{-4})
−0.11 (−0.14, -8.09×10^{-2}) *
MUFA
-1.19×10^{-2} (-3.81×10^{-2}, 1.43×10^{-2})
7.66×10^{-2} (4.88×10^{-2}, 0.1) *
Saturated fatty acids
7.09×10^{-2} (4.3×10^{-2}, 9.87×10^{-2})
5.39×10^{-2} (2.26×10^{-2}, 8.51×10^{-2})
Glycolysis related metabolites
Glucose
0.12 (9.52×10^{-2}, 0.14)
0.16 (0.13, 0.18)
Lactate
-9.7×10^{-3} (-3.32×10^{-2}, 1.38×10^{-2})
-2.31×10^{-2} (-4.76×10^{-2}, 1.44×10^{-3})
Pyruvate
-2.27×10^{-2} (-4.97×10^{-2}, 4.37×10^{-3})
-1.79×10^{-2} (-4.59×10^{-2}, 9.99×10^{-3})
Citrate
9.96×10^{-2} (7.31×10^{-2}, 0.13)
0.22 (0.19, 0.25) *
Glycerol
7.9×10^{-2} (5.07×10^{-2}, 0.11)
0.15 (0.12, 0.18)
−0.6 −0.4 −0.2 0 0.2 0.4 0.6 0.8
Difference in mean metabolite levels (95% CI) per 1SD age (1SD age = 6.0 [WE], 5.2 [SA] years)
White European
South Asian

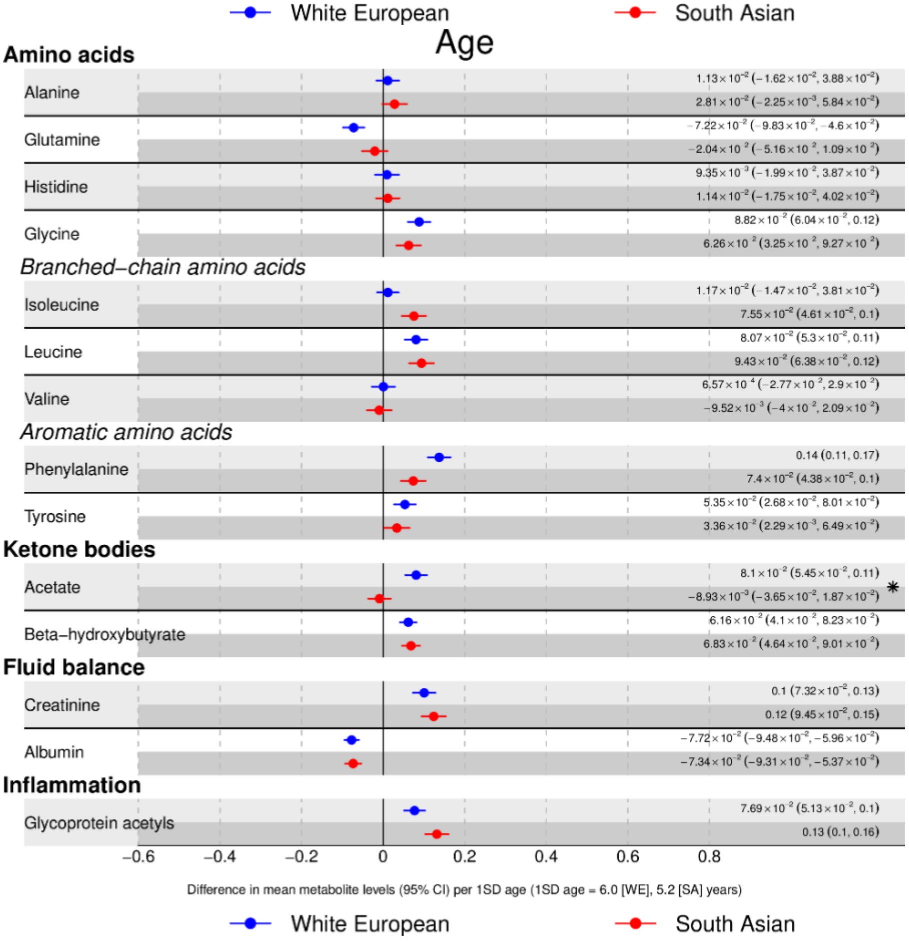
Age
Amino acids
Alanine
1.13×10^{-2} (-1.62×10^{-2}, 3.88×10^{-2})
2.81×10^{-2} (-2.25×10^{-3}, 5.84×10^{-2})
Glutamine
-7.22×10^{-2} (-9.83×10^{-2}, -4.6×10^{-2})
-2.04×10^{-2} (-5.16×10^{-2}, 1.09×10^{-2})
Histidine
9.35×10^{-3} (-1.99×10^{-2}, 3.87×10^{-2})
1.14×10^{-2} (-1.75×10^{-2}, 4.02×10^{-2})
Glycine
8.82×10^{-2} (6.04×10^{-2}, 0.12)
6.26×10^{-2} (3.25×10^{-2}, 9.27×10^{-2})
Branched−chain amino acids
Isoleucine
1.17×10^{-2} (-1.47×10^{-2}, 3.81×10^{-2})
7.55×10^{-2} (4.61×10^{-2}, 0.1)
Leucine
8.07×10^{-2} (5.3×10^{-2}, 0.11)
9.43×10^{-2} (6.38×10^{-2}, 0.12)
Valine
6.57×10^{-4} (-2.77×10^{-2}, 2.9×10^{-2})
-9.52×10^{-3} (-4×10^{-2}, 2.09×10^{-2})
Aromatic amino acids
Phenylalanine
0.14 (0.11, 0.17)
7.4×10^{-2} (4.38×10^{-2}, 0.1)
Tyrosine
5.35×10^{-2} (2.68×10^{-2}, 8.01×10^{-2})
3.36×10^{-2} (2.29×10^{-3}, 6.49×10^{-2})
Ketone bodies
Acetate
8.1×10^{-2} (5.45×10^{-2}, 0.11)
-8.93×10^{-3} (-3.65×10^{-2}, 1.87×10^{-2}) *
Beta−hydroxybutyrate
6.16×10^{-2} (4.1×10^{-2}, 8.23×10^{-2})
6.83×10^{-2} (4.64×10^{-2}, 9.01×10^{-2})
Fluid balance
Creatinine
0.1 (7.32×10^{-2}, 0.13)
0.12 (9.45×10^{-2}, 0.15)
Albumin
-7.72×10^{-2} (-9.48×10^{-2}, -5.96×10^{-2})
-7.34×10^{-2} (-9.31×10^{-2}, -5.37×10^{-2})
Inflammation
Glycoprotein acetyls
7.69×10^{-2} (5.13×10^{-2}, 0.1)
0.13 (0.1, 0.16)
−0.6 −0.4 −0.2 0 0.2 0.4 0.6 0.8
Difference in mean metabolite levels (95% CI) per 1SD age (1SD age = 6.0 [WE], 5.2 [SA] years)
White European
South Asian

Figure S4. Associations of maternal age with pregnancy metabolic profiles stratified by ethnicity. Data points show SD differences for White European (blue) and South Asian (red) women. Error bars = 95% confidence intervals. Exact point estimates and their corresponding 95% CIs are displayed in text to the right of each point. * denotes strong statistical evidence from the interaction test (Pinteraction <0.001). Differences in quantified units (mostly mmol/l) are listed in Supplementary File 2. Abbreviations: VLDL, very low-density lipoprotein; LDL, low-density lipoprotein; HDL, high-density lipoprotein; C, cholesterol; MUFA, monounsaturated fatty acids; PUFA, polyunsaturated fatty acids

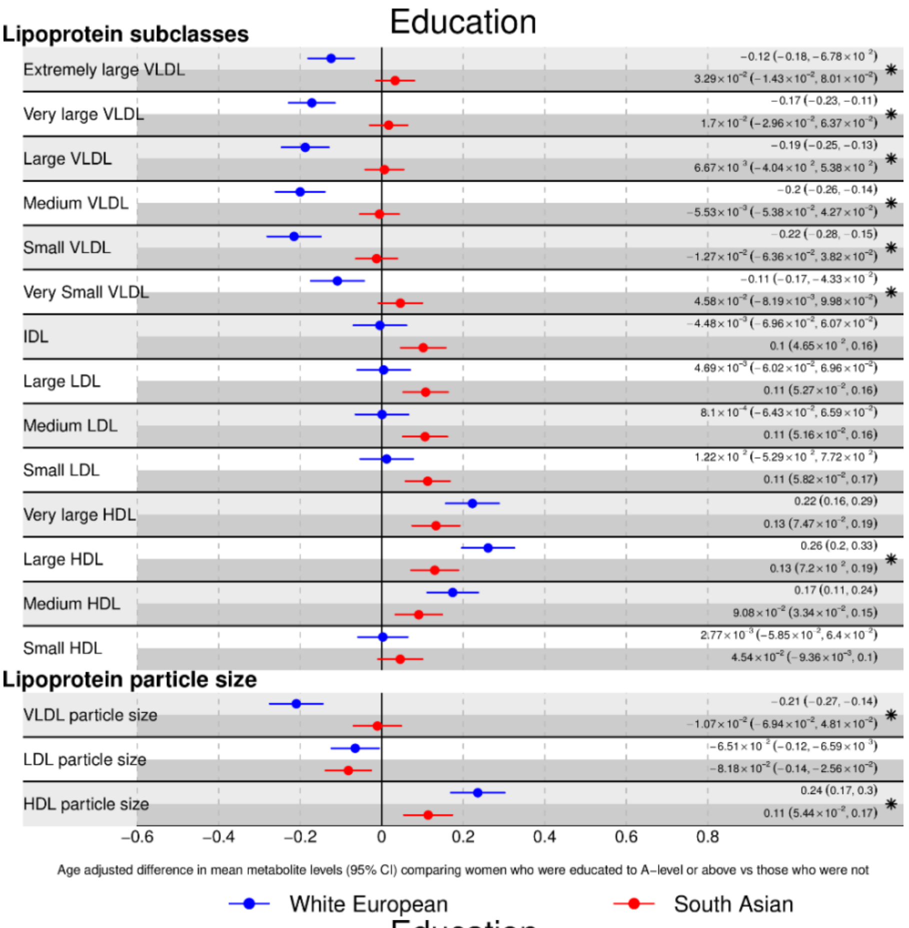
Education
Lipoprotein subclasses
Extremely large VLDL
−0.12 (−0.18, −6.78×10⁻²)
3.29×10⁻² (−1.43×10⁻², 8.01×10⁻²)
Very large VLDL
−0.17 (−0.23, −0.11)
1.7×10⁻² (−2.96×10⁻², 6.37×10⁻²)
Large VLDL
−0.19 (−0.25, −0.13)
6.67×10⁻³ (−4.04×10⁻², 5.38×10⁻²)
Medium VLDL
−0.2 (−0.26, −0.14)
−5.53×10⁻³ (−5.38×10⁻², 4.27×10⁻²)
Small VLDL
−0.22 (−0.28, −0.15)
−1.27×10⁻² (−6.36×10⁻², 3.82×10⁻²)
Very Small VLDL
−0.11 (−0.17, −4.33×10⁻²)
4.58×10⁻² (−8.19×10⁻³, 9.98×10⁻²)
IDL
−4.48×10⁻³ (−6.96×10⁻², 6.07×10⁻²)
0.1 (4.65×10⁻², 0.16)
Large LDL
4.69×10⁻³ (−6.02×10⁻², 6.96×10⁻²)
0.11 (5.27×10⁻², 0.16)
Medium LDL
8.1×10⁻⁴ (−6.43×10⁻², 6.59×10⁻²)
0.11 (5.16×10⁻², 0.16)
Small LDL
1.22×10⁻² (−5.29×10⁻², 7.72×10⁻²)
0.11 (5.82×10⁻², 0.17)
Very large HDL
0.22 (0.16, 0.29)
0.13 (7.47×10⁻², 0.19)
Large HDL
0.26 (0.2, 0.33)
0.13 (7.2×10⁻², 0.19)
Medium HDL
0.17 (0.11, 0.24)
9.08×10⁻² (3.34×10⁻², 0.15)
Small HDL
2.77×10⁻³ (−5.85×10⁻², 6.4×10⁻²)
4.54×10⁻² (−9.36×10⁻³, 0.1)
Lipoprotein particle size
VLDL particle size
−0.21 (−0.27, −0.14)
−1.07×10⁻² (−6.94×10⁻², 4.81×10⁻²)
LDL particle size
−6.51×10⁻² (−0.12, −6.59×10⁻³)
−8.18×10⁻² (−0.14, −2.56×10⁻²)
HDL particle size
0.24 (0.17, 0.3)
0.11 (5.44×10⁻², 0.17)
−0.6 −0.4 −0.2 0 0.2 0.4 0.6 0.8
Age adjusted difference in mean metabolite levels (95% CI) comparing women who were educated to A−level or above vs those who were not
White European
South Asian

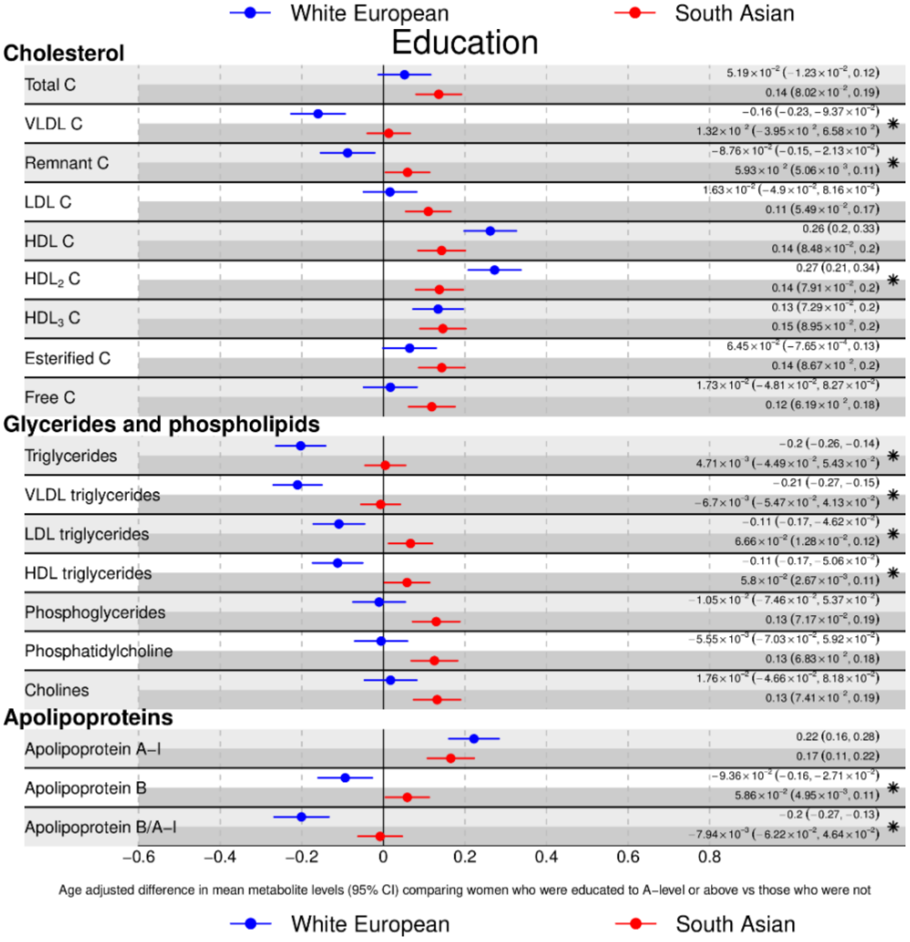
Education
Cholesterol
Total C
5.19×10⁻² (−1.23×10⁻², 0.12)
0.14 (8.02×10⁻², 0.19)
VLDL C
−0.16 (−0.23, −9.37×10⁻²)
1.32×10⁻² (−3.95×10⁻², 6.58×10⁻²)
Remnant C
−8.76×10⁻² (−0.15, −2.13×10⁻²)
5.93×10⁻² (5.06×10⁻³, 0.11)
LDL C
1.63×10⁻² (−4.9×10⁻², 8.16×10⁻²)
0.11 (5.49×10⁻², 0.17)
HDL C
0.26 (0.2, 0.33)
0.14 (8.48×10⁻², 0.2)
HDL2 C
0.27 (0.21, 0.34)
0.14 (7.91×10⁻², 0.2)
HDL3 C
0.13 (7.29×10⁻², 0.2)
0.15 (8.95×10⁻², 0.2)
Esterified C
6.45×10⁻² (−7.65×10⁻⁴, 0.13)
0.14 (8.67×10⁻², 0.2)
Free C
1.73×10⁻² (−4.81×10⁻², 8.27×10⁻²)
0.12 (6.19×10⁻², 0.18)
Glycerides and phospholipids
Triglycerides
−0.2 (−0.26, −0.14)
4.71×10⁻³ (−4.49×10⁻², 5.43×10⁻²)
VLDL triglycerides
−0.21 (−0.27, −0.15)
−6.7×10⁻³ (−5.47×10⁻², 4.13×10⁻²)
LDL triglycerides
−0.11 (−0.17, −4.62×10⁻²)
6.66×10⁻² (1.28×10⁻², 0.12)
HDL triglycerides
−0.11 (−0.17, −5.06×10⁻²)
5.8×10⁻² (2.67×10⁻³, 0.11)
Phosphoglycerides
−1.05×10⁻³ (−7.46×10⁻², 5.37×10⁻²)
0.13 (7.17×10⁻², 0.19)
Phosphatidylcholine
−5.55×10⁻³ (−7.03×10⁻², 5.92×10⁻²)
0.13 (6.83×10⁻², 0.18)
Cholines
1.76×10⁻² (−4.66×10⁻², 8.18×10⁻²)
0.13 (7.41×10⁻², 0.19)
Apolipoproteins
Apolipoprotein A−I
0.22 (0.16, 0.28)
0.17 (0.11, 0.22)
Apolipoprotein B
−9.36×10⁻² (−0.16, −2.71×10⁻²)
5.86×10⁻² (4.95×10⁻³, 0.11)
Apolipoprotein B/A−I
−0.2 (−0.27, −0.13)
−7.94×10⁻³ (−6.22×10⁻², 4.64×10⁻²)
−0.6 −0.4 −0.2 0 0.2 0.4 0.6 0.8
Age adjusted difference in mean metabolite levels (95% CI) comparing women who were educated to A−level or above vs those who were not
White European
South Asian

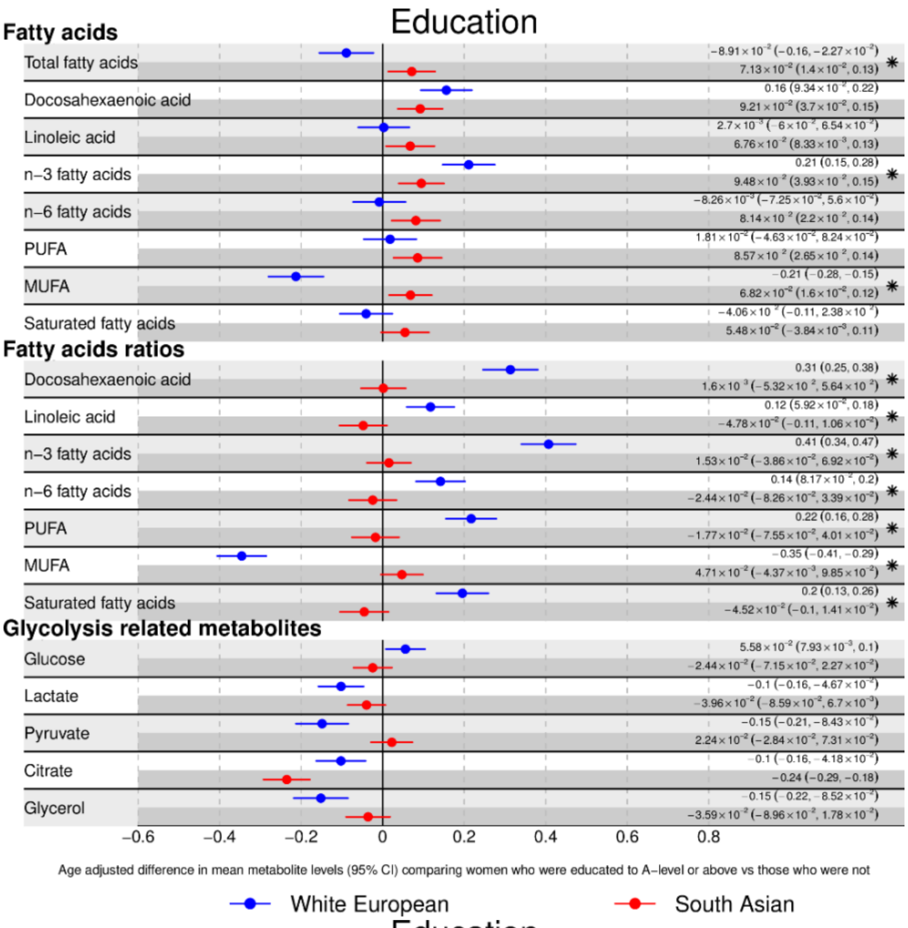

Education
Fatty acids
Total fatty acids
-8.91×10^{-2} (-0.16, -2.27×10^{-2})
7.13×10^{-2} (1.4×10^{-2}, 0.13) *
Docosahexaenoic acid
0.16 (9.34×10^{-2}, 0.22)
9.21×10^{-2} (3.7×10^{-2}, 0.15)
Linoleic acid
2.7×10^{-3} (-6×10^{-2}, 6.54×10^{-2})
6.76×10^{-2} (8.33×10^{-3}, 0.13)
n−3 fatty acids
0.21 (0.15, 0.28)
9.48×10^{-2} (3.93×10^{-2}, 0.15) *
n−6 fatty acids
-8.26×10^{-3} (-7.25×10^{-2}, 5.6×10^{-2})
8.14×10^{-2} (2.2×10^{-2}, 0.14)
PUFA
1.81×10^{-2} (-4.63×10^{-2}, 8.24×10^{-2})
8.57×10^{-2} (2.65×10^{-2}, 0.14)
MUFA
-0.21 (-0.28, -0.15)
6.82×10^{-2} (1.6×10^{-2}, 0.12) *
Saturated fatty acids
-4.06×10^{-2} (-0.11, 2.38×10^{-2})
5.48×10^{-2} (-3.84×10^{-3}, 0.11)
Fatty acids ratios
Docosahexaenoic acid
0.31 (0.25, 0.38)
1.6×10^{-3} (-5.32×10^{-2}, 5.64×10^{-2}) *
Linoleic acid
0.12 (5.92×10^{-2}, 0.18)
-4.78×10^{-2} (-0.11, 1.06×10^{-2}) *
n−3 fatty acids
0.41 (0.34, 0.47)
1.53×10^{-2} (-3.86×10^{-2}, 6.92×10^{-2}) *
n−6 fatty acids
0.14 (8.17×10^{-2}, 0.2)
-2.44×10^{-2} (-8.26×10^{-2}, 3.39×10^{-2}) *
PUFA
0.22 (0.16, 0.28)
-1.77×10^{-2} (-7.55×10^{-2}, 4.01×10^{-2}) *
MUFA
-0.35 (-0.41, -0.29)
4.71×10^{-2} (-4.37×10^{-3}, 9.85×10^{-2}) *
Saturated fatty acids
0.2 (0.13, 0.26)
-4.52×10^{-2} (-0.1, 1.41×10^{-2}) *
Glycolysis related metabolites
Glucose
5.58×10^{-2} (7.93×10^{-3}, 0.1)
-2.44×10^{-2} (-7.15×10^{-2}, 2.27×10^{-2})
Lactate
-0.1 (-0.16, -4.67×10^{-2})
-3.96×10^{-2} (-8.59×10^{-2}, 6.7×10^{-3})
Pyruvate
-0.15 (-0.21, -8.43×10^{-2})
2.24×10^{-2} (-2.84×10^{-2}, 7.31×10^{-2})
Citrate
-0.1 (-0.16, -4.18×10^{-2})
-0.24 (-0.29, -0.18)
Glycerol
-0.15 (-0.22, -8.52×10^{-2})
-3.59×10^{-2} (-8.96×10^{-2}, 1.78×10^{-2})
−0.6 −0.4 −0.2 0 0.2 0.4 0.6 0.8
Age adjusted difference in mean metabolite levels (95% CI) comparing women who were educated to A−level or above vs those who were not
White European
South Asian

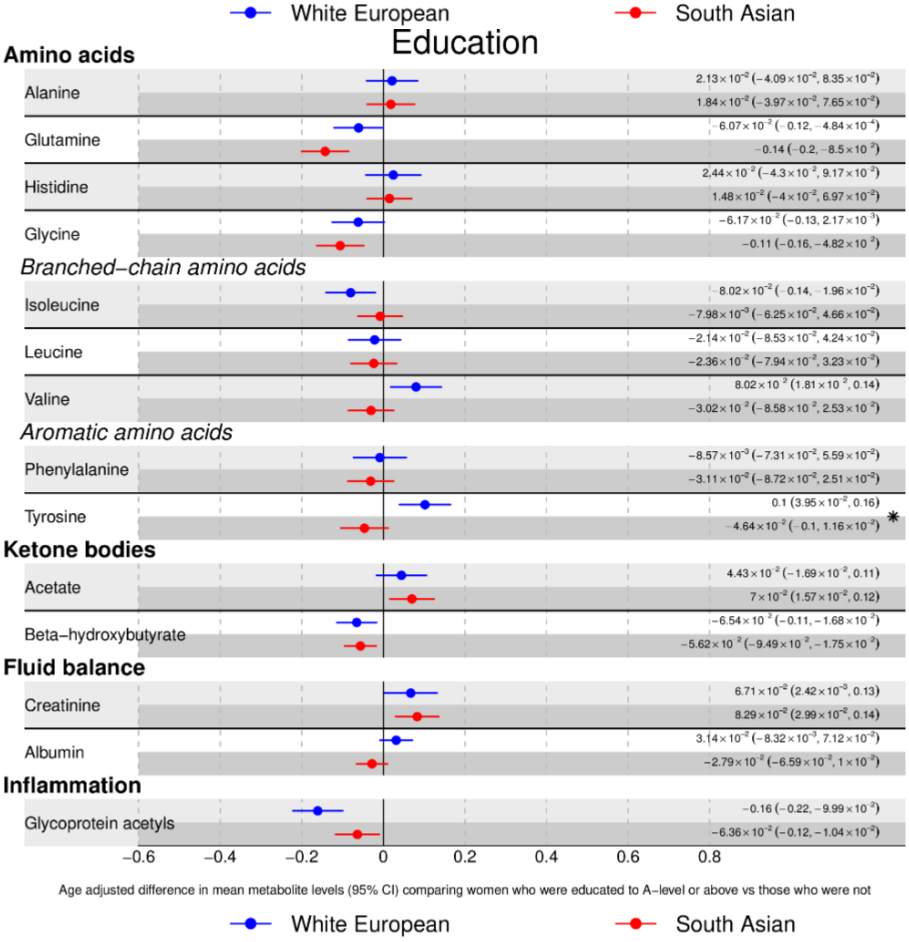

Education
Amino acids
Alanine
2.13×10^{-2} (-4.09×10^{-2}, 8.35×10^{-2})
1.84×10^{-2} (-3.97×10^{-2}, 7.65×10^{-2})
Glutamine
-6.07×10^{-2} (-0.12, -4.84×10^{-4})
-0.14 (-0.2, -8.5×10^{-2})
Histidine
2.44×10^{-2} (-4.3×10^{-2}, 9.17×10^{-2})
1.48×10^{-2} (-4×10^{-2}, 6.97×10^{-2})
Glycine
-6.17×10^{-2} (-0.13, 2.17×10^{-3})
-0.11 (-0.16, -4.82×10^{-2})
Branched−chain amino acids
Isoleucine
-8.02×10^{-2} (-0.14, -1.96×10^{-2})
-7.98×10^{-3} (-6.25×10^{-2}, 4.66×10^{-2})
Leucine
-2.14×10^{-2} (-8.53×10^{-2}, 4.24×10^{-2})
-2.36×10^{-2} (-7.94×10^{-2}, 3.23×10^{-2})
Valine
8.02×10^{-2} (1.81×10^{-2}, 0.14)
-3.02×10^{-2} (-8.58×10^{-2}, 2.53×10^{-2})
Aromatic amino acids
Phenylalanine
-8.57×10^{-3} (-7.31×10^{-2}, 5.59×10^{-2})
-3.11×10^{-2} (-8.72×10^{-2}, 2.51×10^{-2})
Tyrosine
0.1 (3.95×10^{-2}, 0.16)
-4.64×10^{-2} (-0.1, 1.16×10^{-2}) *
Ketone bodies
Acetate
4.43×10^{-2} (-1.69×10^{-2}, 0.11)
7×10^{-2} (1.57×10^{-2}, 0.12)
Beta−hydroxybutyrate
-6.54×10^{-2} (-0.11, -1.68×10^{-2})
-5.62×10^{-2} (-9.49×10^{-2}, -1.75×10^{-2})
Fluid balance
Creatinine
6.71×10^{-2} (2.42×10^{-3}, 0.13)
8.29×10^{-2} (2.99×10^{-2}, 0.14)
Albumin
3.14×10^{-2} (-8.32×10^{-3}, 7.12×10^{-2})
-2.79×10^{-2} (-6.59×10^{-2}, 1×10^{-2})
Inflammation
Glycoprotein acetyls
-0.16 (-0.22, -9.99×10^{-2})
-6.36×10^{-2} (-0.12, -1.04×10^{-2})
−0.6 −0.4 −0.2 0 0.2 0.4 0.6 0.8
Age adjusted difference in mean metabolite levels (95% CI) comparing women who were educated to A−level or above vs those who were not
White European
South Asian

Figure S5. Age-adjusted associations of maternal education with pregnancy metabolic profiles stratified by ethnicity. Data points show SD differences for White European (blue) and South Asian (red) women. Error bars = 95% confidence intervals. Exact point estimates and their corresponding 95% CIs are displayed in text to the right of each point. * denotes strong statistical evidence from the interaction test (Pinteraction <0.001). Differences in quantified units (mostly mmol/l) are listed in Supplementary File 2. Abbreviations: VLDL, very low-density lipoprotein; LDL, low-density lipoprotein; HDL, high-density lipoprotein; C, cholesterol; MUFA, monounsaturated fatty acids; PUFA, polyunsaturated fatty acids

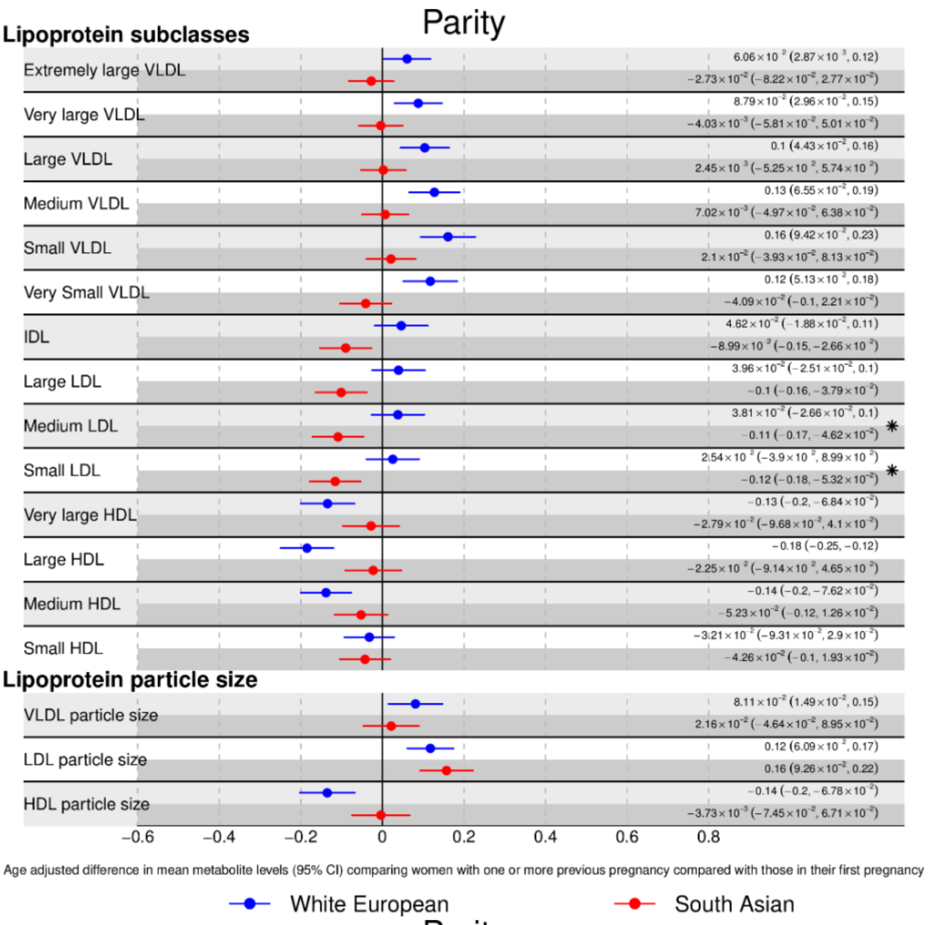
Parity
Lipoprotein subclasses
Extremely large VLDL
Very large VLDL
Large VLDL
Medium VLDL
Small VLDL
Very Small VLDL
IDL
Large LDL
Medium LDL
Small LDL
Very large HDL
Large HDL
Medium HDL
Small HDL
Lipoprotein particle size
VLDL particle size
LDL particle size
HDL particle size
−0.6 −0.4 −0.2 0 0.2 0.4 0.6 0.8
Age adjusted difference in mean metabolite levels (95% CI) comparing women with one or more previous pregnancy compared with those in their first pregnancy
White European
South Asian

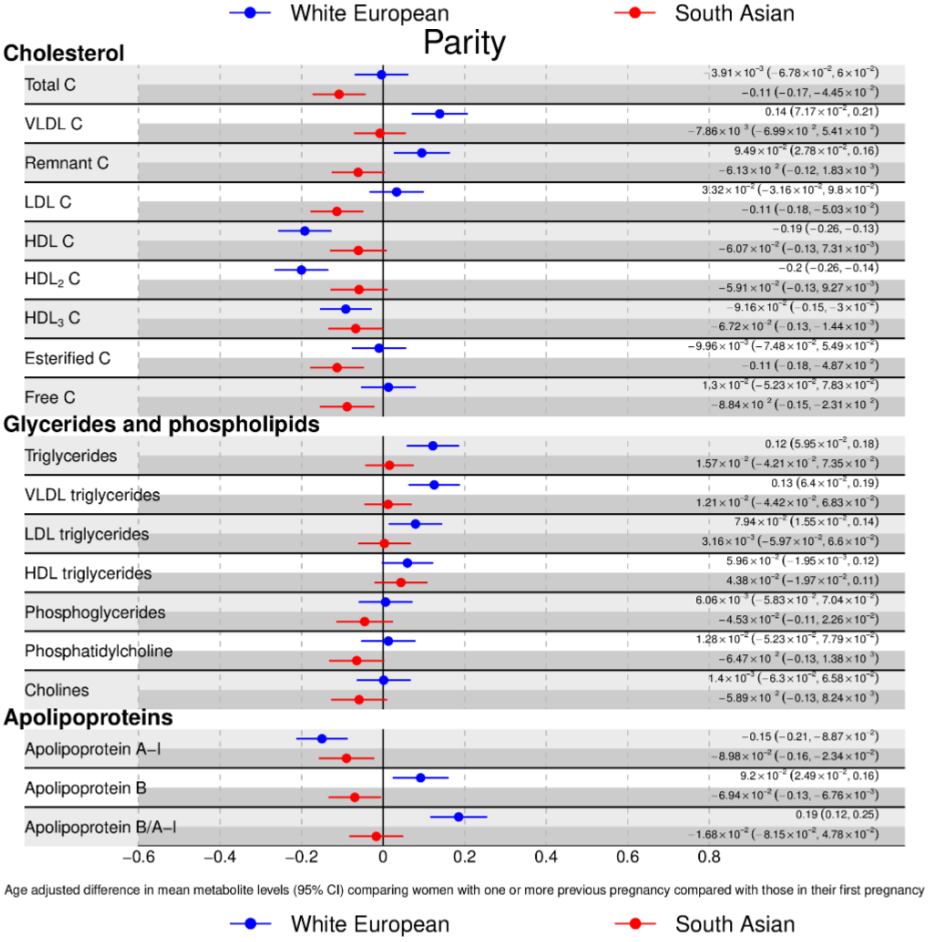
Parity
Cholesterol
Total C
VLDL C
Remnant C
LDL C
HDL C
HDL2 C
HDL3 C
Esterified C
Free C
Glycerides and phospholipids
Triglycerides
VLDL triglycerides
LDL triglycerides
HDL triglycerides
Phosphoglycerides
Phosphatidylcholine
Cholines
Apolipoproteins
Apolipoprotein A–I
Apolipoprotein B
Apolipoprotein B/A–I
−0.6 −0.4 −0.2 0 0.2 0.4 0.6 0.8
Age adjusted difference in mean metabolite levels (95% CI) comparing women with one or more previous pregnancy compared with those in their first pregnancy
White European
South Asian

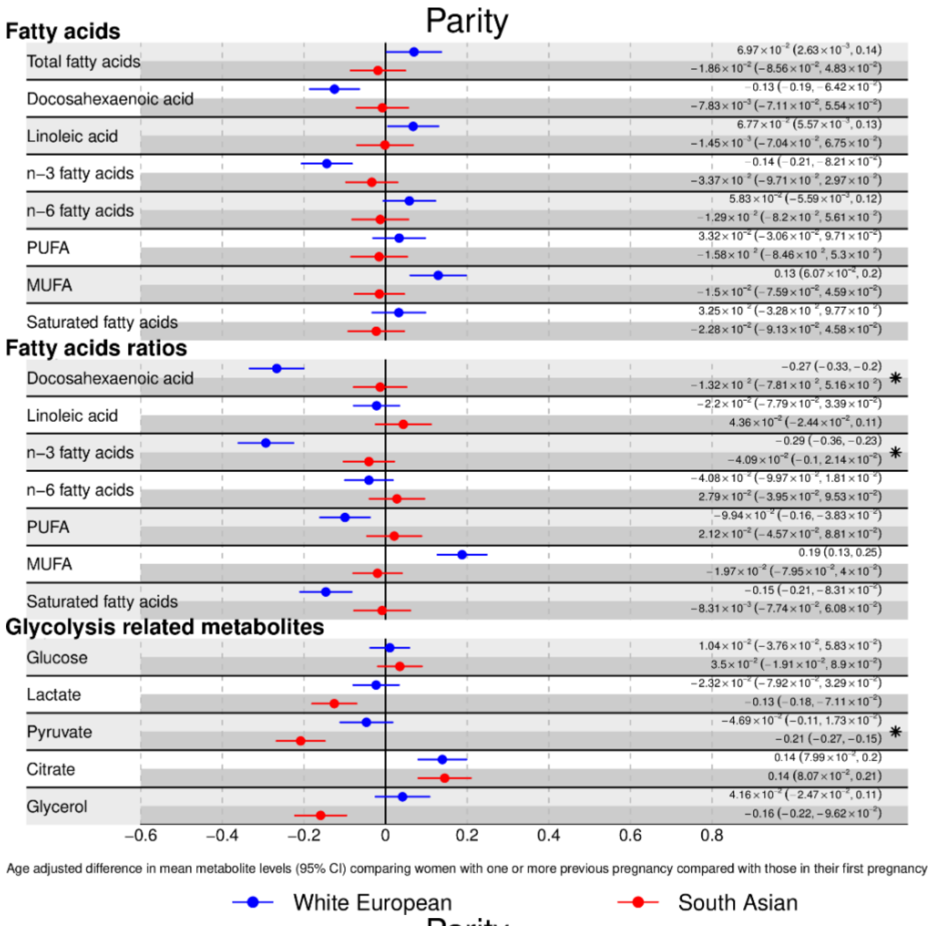
Parity
Fatty acids
Total fatty acids
Docosahexaenoic acid
Linoleic acid
n−3 fatty acids
n−6 fatty acids
PUFA
MUFA
Saturated fatty acids
Fatty acids ratios
Docosahexaenoic acid
Linoleic acid
n−3 fatty acids
n−6 fatty acids
PUFA
MUFA
Saturated fatty acids
Glycolysis related metabolites
Glucose
Lactate
Pyruvate
Citrate
Glycerol
−0.6 −0.4 −0.2 0 0.2 0.4 0.6 0.8
Age adjusted difference in mean metabolite levels (95% CI) comparing women with one or more previous pregnancy compared with those in their first pregnancy
White European
South Asian

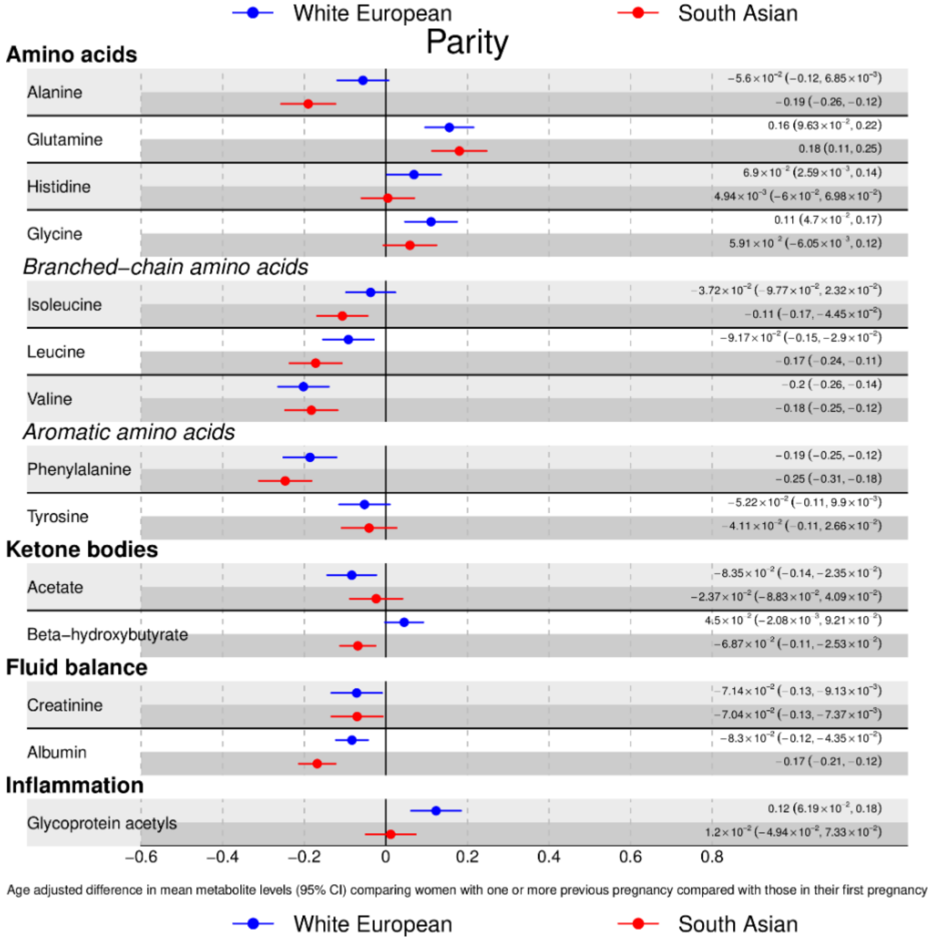
Parity
Amino acids
Alanine
Glutamine
Histidine
Glycine
Branched−chain amino acids
Isoleucine
Leucine
Valine
Aromatic amino acids
Phenylalanine
Tyrosine
Ketone bodies
Acetate
Beta−hydroxybutyrate
Fluid balance
Creatinine
Albumin
Inflammation
Glycoprotein acetyls
−0.6 −0.4 −0.2 0 0.2 0.4 0.6 0.8
Age adjusted difference in mean metabolite levels (95% CI) comparing women with one or more previous pregnancy compared with those in their first pregnancy
White European
South Asian

Figure S6. Age-adjusted associations of maternal parity with pregnancy metabolic profiles stratified by ethnicity. Data points show SD differences for White European (blue) and South Asian (red) women. Error bars = 95% confidence intervals. Exact point estimates and their corresponding 95% CIs are displayed in text to the right of each point. * denotes strong statistical evidence from the interaction test (Pinteraction <0.001). Differences in quantified units (mostly mmol/l) are listed in Supplementary File 2. Abbreviations: VLDL, very low-density lipoprotein; LDL, low-density lipoprotein; HDL, high-density lipoprotein; C, cholesterol; MUFA, monounsaturated fatty acids; PUFA, polyunsaturated fatty acids

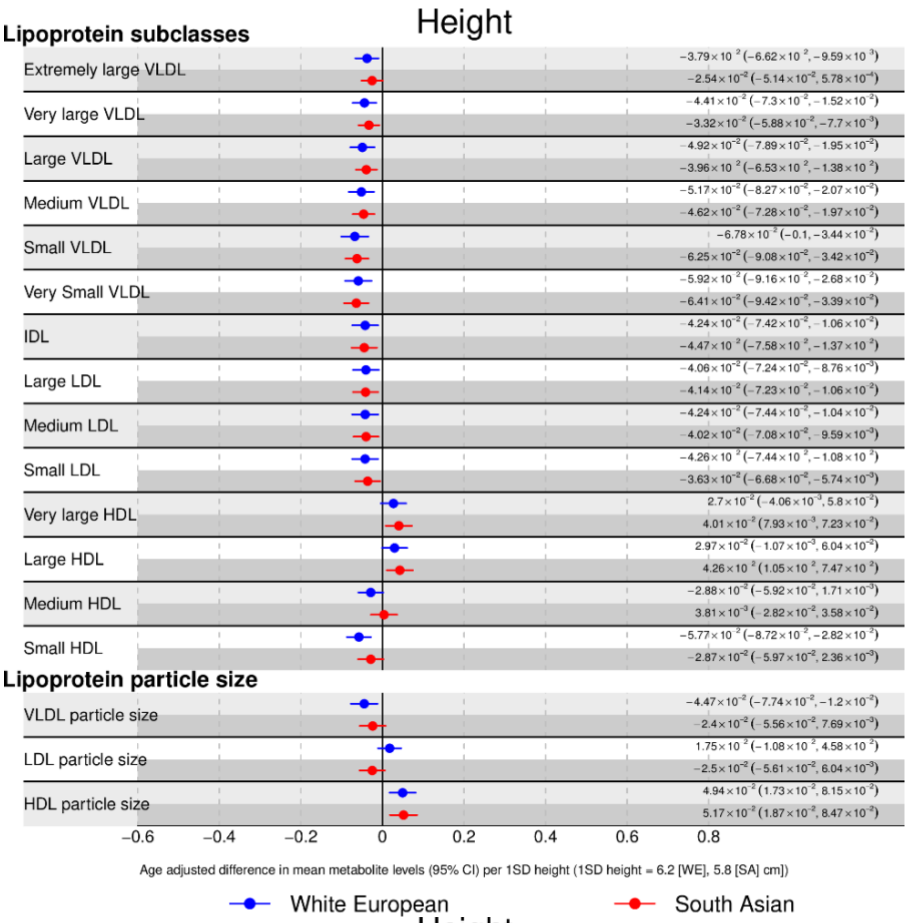
Height
Lipoprotein subclasses
Extremely large VLDL
-3.79×10^{-2} (-6.62×10^{-2}, -9.59×10^{-3})
-2.54×10^{-2} (-5.14×10^{-2}, 5.78×10^{-4})
Very large VLDL
-4.41×10^{-2} (-7.3×10^{-2}, -1.52×10^{-2})
-3.32×10^{-2} (-5.88×10^{-2}, -7.7×10^{-3})
Large VLDL
-4.92×10^{-2} (-7.89×10^{-2}, -1.95×10^{-2})
-3.96×10^{-2} (-6.53×10^{-2}, -1.38×10^{-2})
Medium VLDL
-5.17×10^{-2} (-8.27×10^{-2}, -2.07×10^{-2})
-4.62×10^{-2} (-7.28×10^{-2}, -1.97×10^{-2})
Small VLDL
-6.78×10^{-2} (-0.1, -3.44×10^{-2})
-6.25×10^{-2} (-9.08×10^{-2}, -3.42×10^{-2})
Very Small VLDL
-5.92×10^{-2} (-9.16×10^{-2}, -2.68×10^{-2})
-6.41×10^{-2} (-9.42×10^{-2}, -3.39×10^{-2})
IDL
-4.24×10^{-2} (-7.42×10^{-2}, -1.06×10^{-2})
-4.47×10^{-2} (-7.58×10^{-2}, -1.37×10^{-2})
Large LDL
-4.06×10^{-2} (-7.24×10^{-2}, -8.76×10^{-3})
-4.14×10^{-2} (-7.23×10^{-2}, -1.06×10^{-2})
Medium LDL
-4.24×10^{-2} (-7.44×10^{-2}, -1.04×10^{-2})
-4.02×10^{-2} (-7.08×10^{-2}, -9.59×10^{-3})
Small LDL
-4.26×10^{-2} (-7.44×10^{-2}, -1.08×10^{-2})
-3.63×10^{-2} (-6.68×10^{-2}, -5.74×10^{-3})
Very large HDL
2.7×10^{-2} (-4.06×10^{-3}, 5.8×10^{-2})
4.01×10^{-2} (7.93×10^{-3}, 7.23×10^{-2})
Large HDL
2.97×10^{-2} (-1.07×10^{-3}, 6.04×10^{-2})
4.26×10^{-2} (1.05×10^{-2}, 7.47×10^{-2})
Medium HDL
-2.88×10^{-2} (-5.92×10^{-2}, 1.71×10^{-3})
3.81×10^{-3} (-2.82×10^{-2}, 3.58×10^{-2})
Small HDL
-5.77×10^{-2} (-8.72×10^{-2}, -2.82×10^{-2})
-2.87×10^{-2} (-5.97×10^{-2}, 2.36×10^{-3})
Lipoprotein particle size
VLDL particle size
-4.47×10^{-2} (-7.74×10^{-2}, -1.2×10^{-2})
-2.4×10^{-2} (-5.56×10^{-2}, 7.69×10^{-3})
LDL particle size
1.75×10^{-2} (-1.08×10^{-2}, 4.58×10^{-2})
-2.5×10^{-2} (-5.61×10^{-2}, 6.04×10^{-3})
HDL particle size
4.94×10^{-2} (1.73×10^{-2}, 8.15×10^{-2})
5.17×10^{-2} (1.87×10^{-2}, 8.47×10^{-2})
−0.6
−0.4
−0.2
0
0.2
0.4
0.6
0.8
Age adjusted difference in mean metabolite levels (95% CI) per 1SD height (1SD height = 6.2 [WE], 5.8 [SA] cm])
White European
South Asian

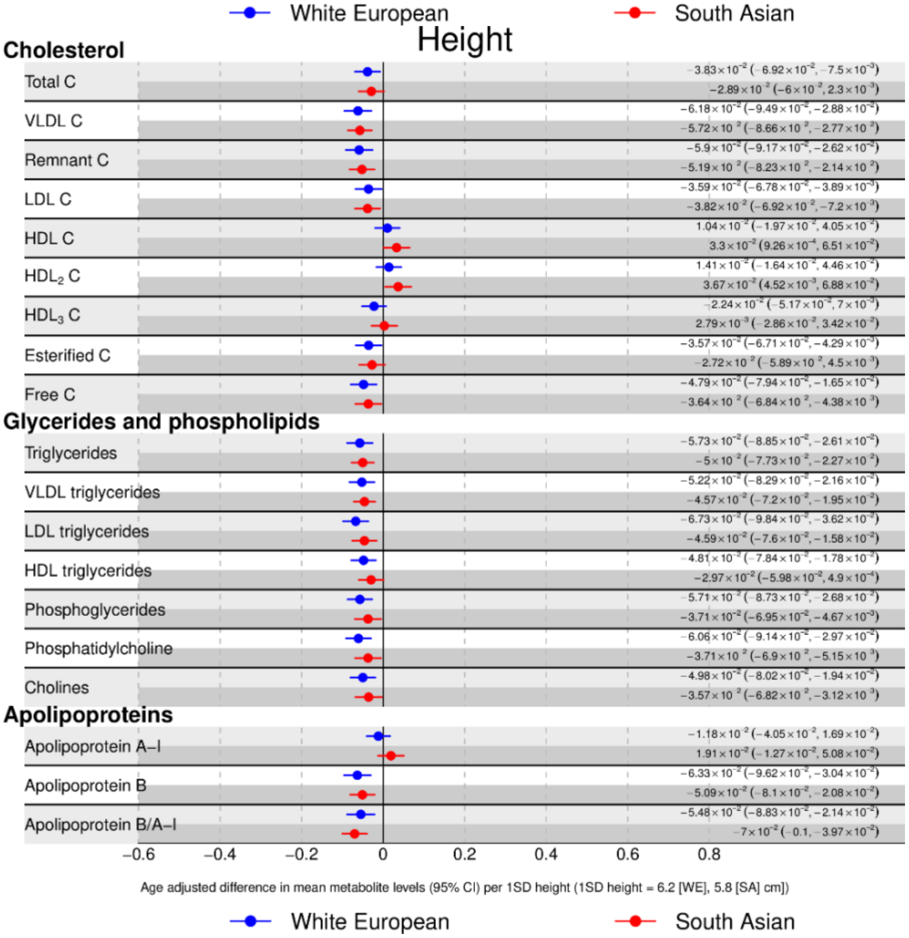
Height
Cholesterol
Total C
-3.83×10^{-2} (-6.92×10^{-2}, -7.5×10^{-3})
-2.89×10^{-2} (-6×10^{-2}, 2.3×10^{-3})
VLDL C
-6.18×10^{-2} (-9.49×10^{-2}, -2.88×10^{-2})
-5.72×10^{-2} (-8.66×10^{-2}, -2.77×10^{-2})
Remnant C
-5.9×10^{-2} (-9.17×10^{-2}, -2.62×10^{-2})
-5.19×10^{-2} (-8.23×10^{-2}, -2.14×10^{-2})
LDL C
-3.59×10^{-2} (-6.78×10^{-2}, -3.89×10^{-3})
-3.82×10^{-2} (-6.92×10^{-2}, -7.2×10^{-3})
HDL C
1.04×10^{-2} (-1.97×10^{-2}, 4.05×10^{-2})
3.3×10^{-2} (9.26×10^{-4}, 6.51×10^{-2})
HDL_2 C
1.41×10^{-2} (-1.64×10^{-2}, 4.46×10^{-2})
3.67×10^{-2} (4.52×10^{-3}, 6.88×10^{-2})
HDL_3 C
-2.24×10^{-2} (-5.17×10^{-2}, 7×10^{-3})
2.79×10^{-3} (-2.86×10^{-2}, 3.42×10^{-2})
Esterified C
-3.57×10^{-2} (-6.71×10^{-2}, -4.29×10^{-3})
-2.72×10^{-2} (-5.89×10^{-2}, 4.5×10^{-3})
Free C
-4.79×10^{-2} (-7.94×10^{-2}, -1.65×10^{-2})
-3.64×10^{-2} (-6.84×10^{-2}, -4.38×10^{-3})
Glycerides and phospholipids
Triglycerides
-5.73×10^{-2} (-8.85×10^{-2}, -2.61×10^{-2})
-5×10^{-2} (-7.73×10^{-2}, -2.27×10^{-2})
VLDL triglycerides
-5.22×10^{-2} (-8.29×10^{-2}, -2.16×10^{-2})
-4.57×10^{-2} (-7.2×10^{-2}, -1.95×10^{-2})
LDL triglycerides
-6.73×10^{-2} (-9.84×10^{-2}, -3.62×10^{-2})
-4.59×10^{-2} (-7.6×10^{-2}, -1.58×10^{-2})
HDL triglycerides
-4.81×10^{-2} (-7.84×10^{-2}, -1.78×10^{-2})
-2.97×10^{-2} (-5.98×10^{-2}, 4.9×10^{-4})
Phosphoglycerides
-5.71×10^{-2} (-8.73×10^{-2}, -2.68×10^{-2})
-3.71×10^{-2} (-6.95×10^{-2}, -4.67×10^{-3})
Phosphatidylcholine
-6.06×10^{-2} (-9.14×10^{-2}, -2.97×10^{-2})
-3.71×10^{-2} (-6.9×10^{-2}, -5.15×10^{-3})
Cholines
-4.98×10^{-2} (-8.02×10^{-2}, -1.94×10^{-2})
-3.57×10^{-2} (-6.82×10^{-2}, -3.12×10^{-3})
Apolipoproteins
Apolipoprotein A–I
-1.18×10^{-2} (-4.05×10^{-2}, 1.69×10^{-2})
1.91×10^{-2} (-1.27×10^{-2}, 5.08×10^{-2})
Apolipoprotein B
-6.33×10^{-2} (-9.62×10^{-2}, -3.04×10^{-2})
-5.09×10^{-2} (-8.1×10^{-2}, -2.08×10^{-2})
Apolipoprotein B/A–I
-5.48×10^{-2} (-8.83×10^{-2}, -2.14×10^{-2})
-7×10^{-2} (-0.1, -3.97×10^{-2})
−0.6
−0.4
−0.2
0
0.2
0.4
0.6
0.8
Age adjusted difference in mean metabolite levels (95% CI) per 1SD height (1SD height = 6.2 [WE], 5.8 [SA] cm])
White European
South Asian

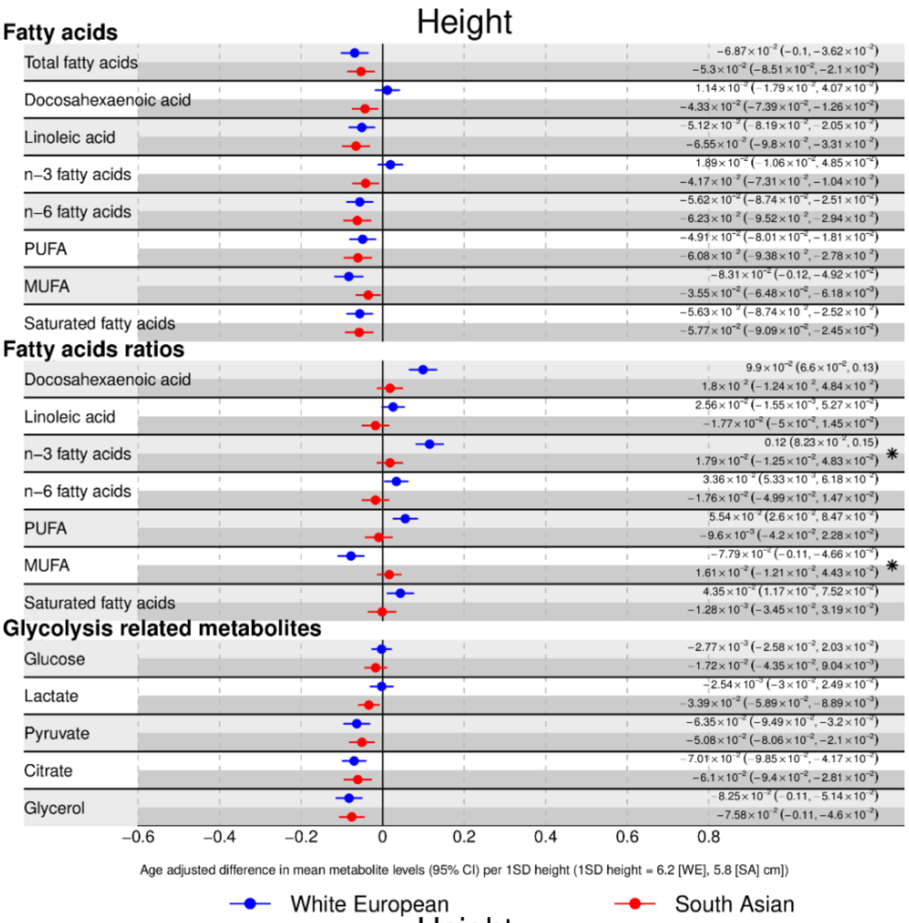
Height
Fatty acids
Total fatty acids
Docosahexaenoic acid
Linoleic acid
n−3 fatty acids
n−6 fatty acids
PUFA
MUFA
Saturated fatty acids
Fatty acids ratios
Docosahexaenoic acid
Linoleic acid
n−3 fatty acids
n−6 fatty acids
PUFA
MUFA
Saturated fatty acids
Glycolysis related metabolites
Glucose
Lactate
Pyruvate
Citrate
Glycerol
−0.6 −0.4 −0.2 0 0.2 0.4 0.6 0.8
Age adjusted difference in mean metabolite levels (95% CI) per 1SD height (1SD height = 6.2 [WE], 5.8 [SA] cm])
White European
South Asian

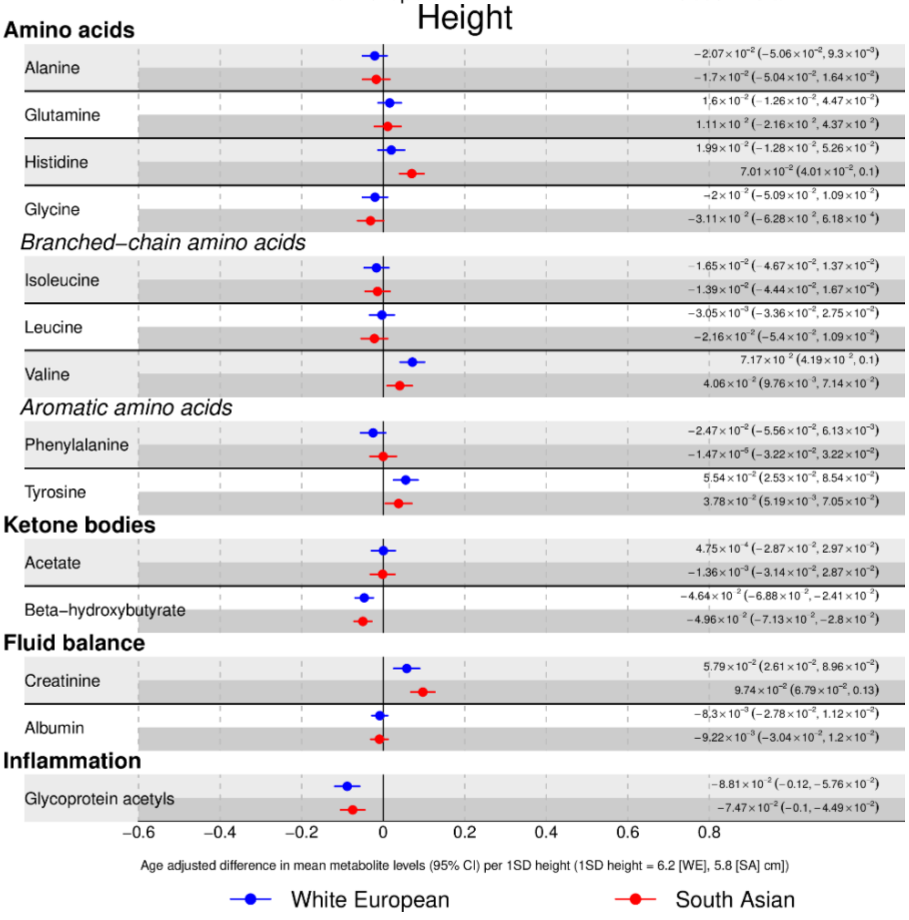
Height
Amino acids
Alanine
Glutamine
Histidine
Glycine
Branched−chain amino acids
Isoleucine
Leucine
Valine
Aromatic amino acids
Phenylalanine
Tyrosine
Ketone bodies
Acetate
Beta−hydroxybutyrate
Fluid balance
Creatinine
Albumin
Inflammation
Glycoprotein acetyls
−0.6 −0.4 −0.2 0 0.2 0.4 0.6 0.8
Age adjusted difference in mean metabolite levels (95% CI) per 1SD height (1SD height = 6.2 [WE], 5.8 [SA] cm])
White European
South Asian

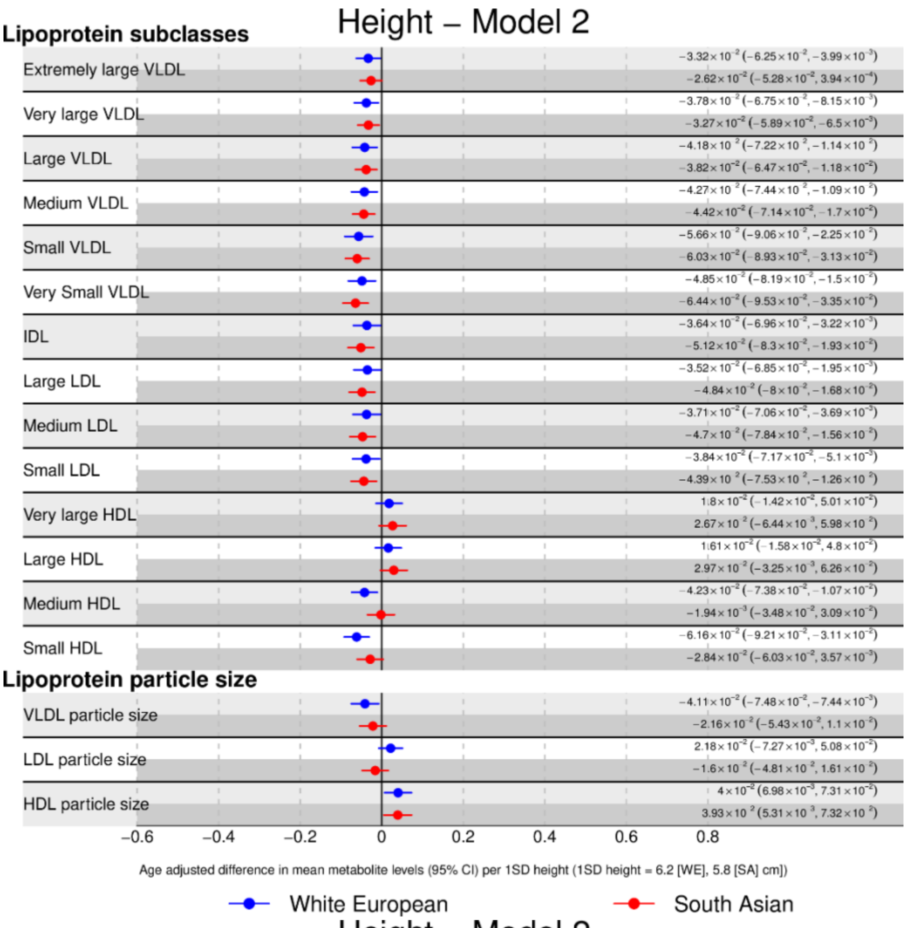
Height – Model 2
Lipoprotein subclasses
Extremely large VLDL
Very large VLDL
Large VLDL
Medium VLDL
Small VLDL
Very Small VLDL
IDL
Large LDL
Medium LDL
Small LDL
Very large HDL
Large HDL
Medium HDL
Small HDL
Lipoprotein particle size
VLDL particle size
LDL particle size
HDL particle size
−0.6 −0.4 −0.2 0 0.2 0.4 0.6 0.8
Age adjusted difference in mean metabolite levels (95% CI) per 1SD height (1SD height = 6.2 [WE], 5.8 [SA] cm])
White European
South Asian

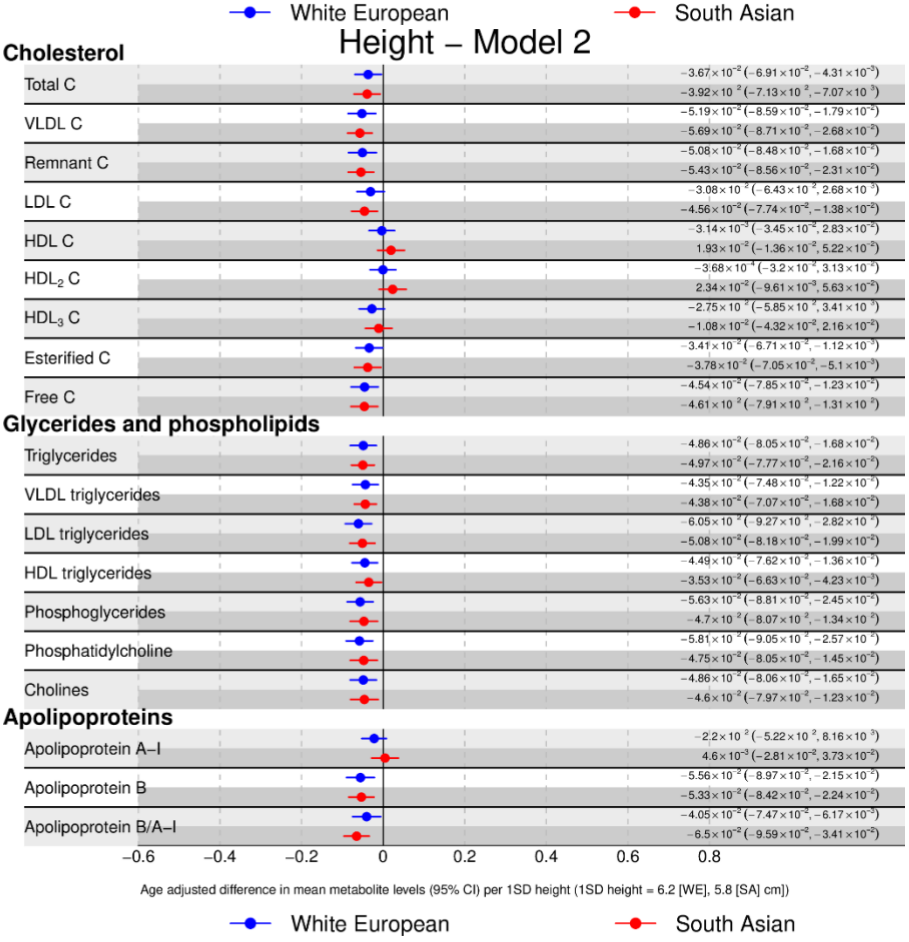
Height – Model 2
Cholesterol
Total C
VLDL C
Remnant C
LDL C
HDL C
HDL2 C
HDL3 C
Esterified C
Free C
Glycerides and phospholipids
Triglycerides
VLDL triglycerides
LDL triglycerides
HDL triglycerides
Phosphoglycerides
Phosphatidylcholine
Cholines
Apolipoproteins
Apolipoprotein A–I
Apolipoprotein B
Apolipoprotein B/A–I
−0.6 −0.4 −0.2 0 0.2 0.4 0.6 0.8
Age adjusted difference in mean metabolite levels (95% CI) per 1SD height (1SD height = 6.2 [WE], 5.8 [SA] cm])
White European
South Asian

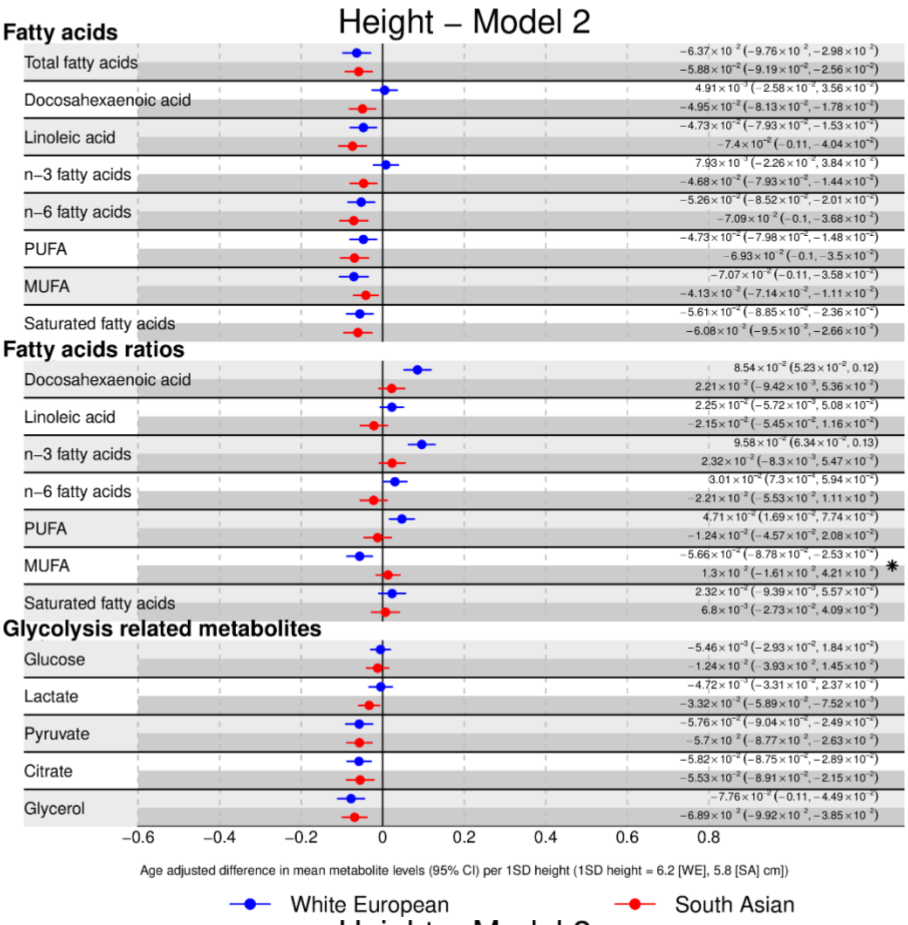

Height – Model 2
Fatty acids
Total fatty acids
Docosahexaenoic acid
Linoleic acid
n−3 fatty acids
n−6 fatty acids
PUFA
MUFA
Saturated fatty acids
Fatty acids ratios
Docosahexaenoic acid
Linoleic acid
n−3 fatty acids
n−6 fatty acids
PUFA
MUFA
Saturated fatty acids
Glycolysis related metabolites
Glucose
Lactate
Pyruvate
Citrate
Glycerol
−0.6 −0.4 −0.2 0 0.2 0.4 0.6 0.8
Age adjusted difference in mean metabolite levels (95% CI) per 1SD height (1SD height = 6.2 [WE], 5.8 [SA] cm])
White European
South Asian

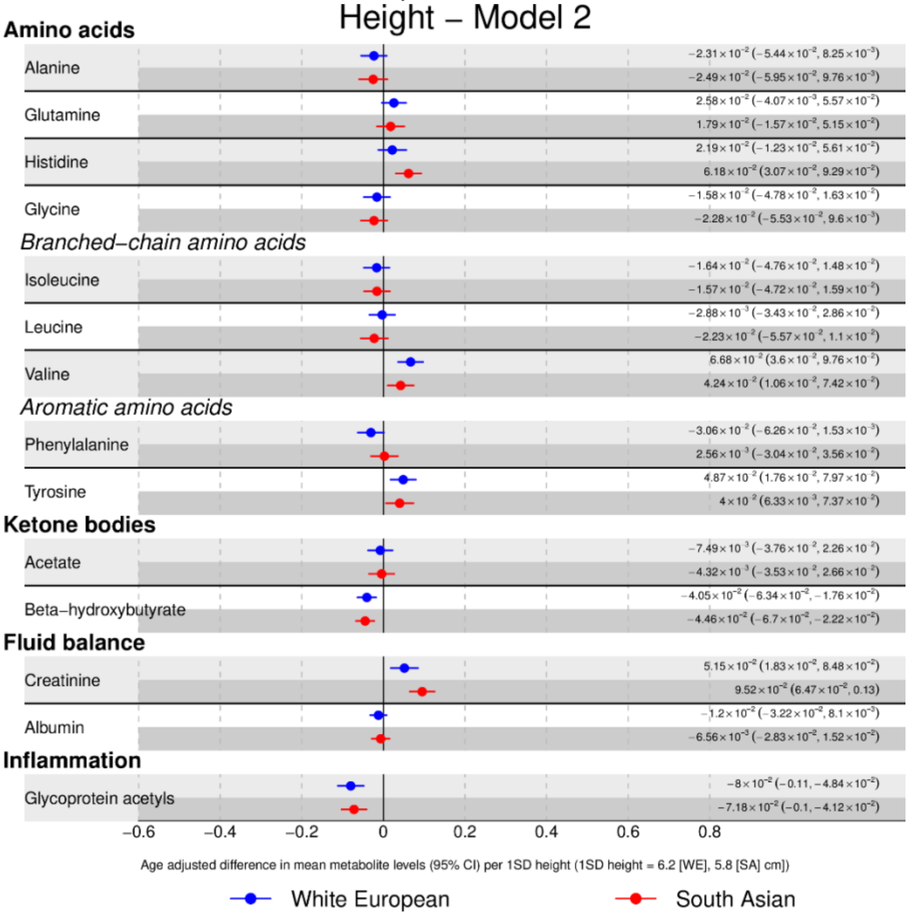

Height – Model 2
Amino acids
Alanine
Glutamine
Histidine
Glycine
Branched−chain amino acids
Isoleucine
Leucine
Valine
Aromatic amino acids
Phenylalanine
Tyrosine
Ketone bodies
Acetate
Beta−hydroxybutyrate
Fluid balance
Creatinine
Albumin
Inflammation
Glycoprotein acetyls
−0.6 −0.4 −0.2 0 0.2 0.4 0.6 0.8
Age adjusted difference in mean metabolite levels (95% CI) per 1SD height (1SD height = 6.2 [WE], 5.8 [SA] cm])
White European
South Asian

Figure S7. Associations of maternal height with pregnancy metabolic profiles stratified by ethnicity (model 1 = age-adjusted; model 2 = age-, parity- and education-adjusted). Data points show SD differences for White European (blue) and South Asian (red) women. Error bars = 95% confidence intervals. Exact point estimates and their corresponding 95% CIs are displayed in text to the right of each point. * denotes strong statistical evidence from the interaction test (Pinteraction <0.001). Differences in quantified units (mostly mmol/l) are listed in Supplementary File 2. Abbreviations: VLDL, very low-density lipoprotein; LDL, low-density lipoprotein; HDL, high-density lipoprotein; C, cholesterol; MUFA, monounsaturated fatty acids; PUFA, polyunsaturated fatty acids

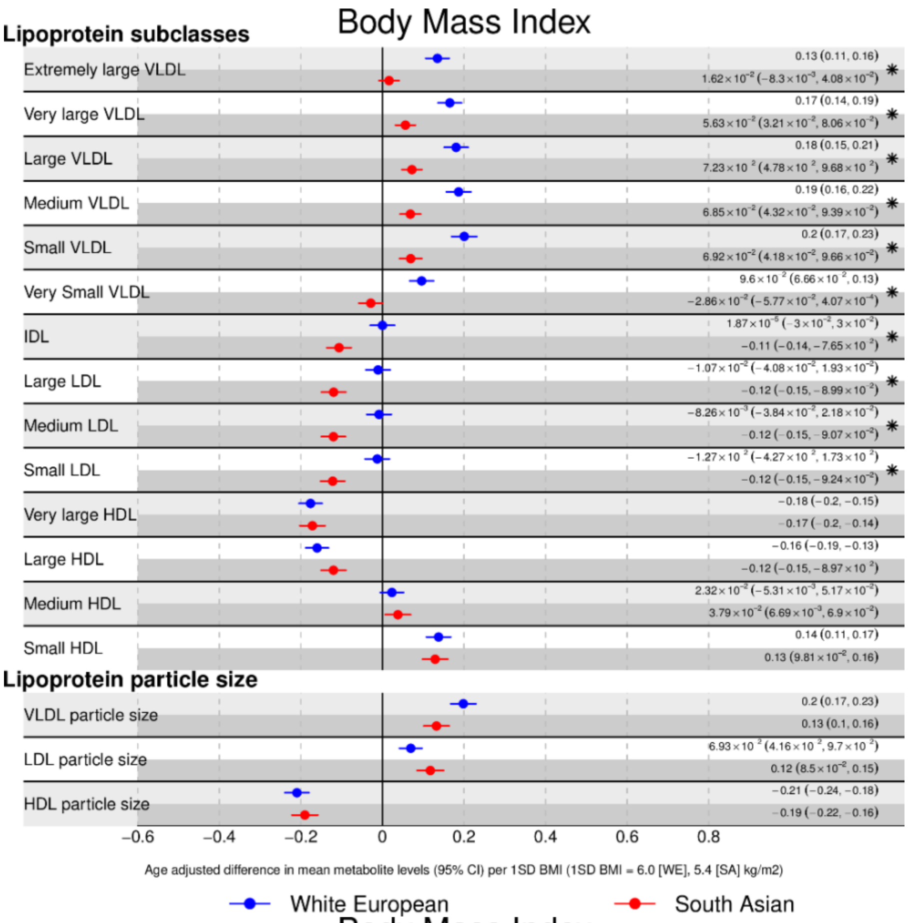
Body Mass Index
Lipoprotein subclasses
Extremely large VLDL
Very large VLDL
Large VLDL
Medium VLDL
Small VLDL
Very Small VLDL
IDL
Large LDL
Medium LDL
Small LDL
Very large HDL
Large HDL
Medium HDL
Small HDL
Lipoprotein particle size
VLDL particle size
LDL particle size
HDL particle size
−0.6 −0.4 −0.2 0 0.2 0.4 0.6 0.8
Age adjusted difference in mean metabolite levels (95% CI) per 1SD BMI (1SD BMI = 6.0 [WE], 5.4 [SA] kg/m2)
White European
South Asian

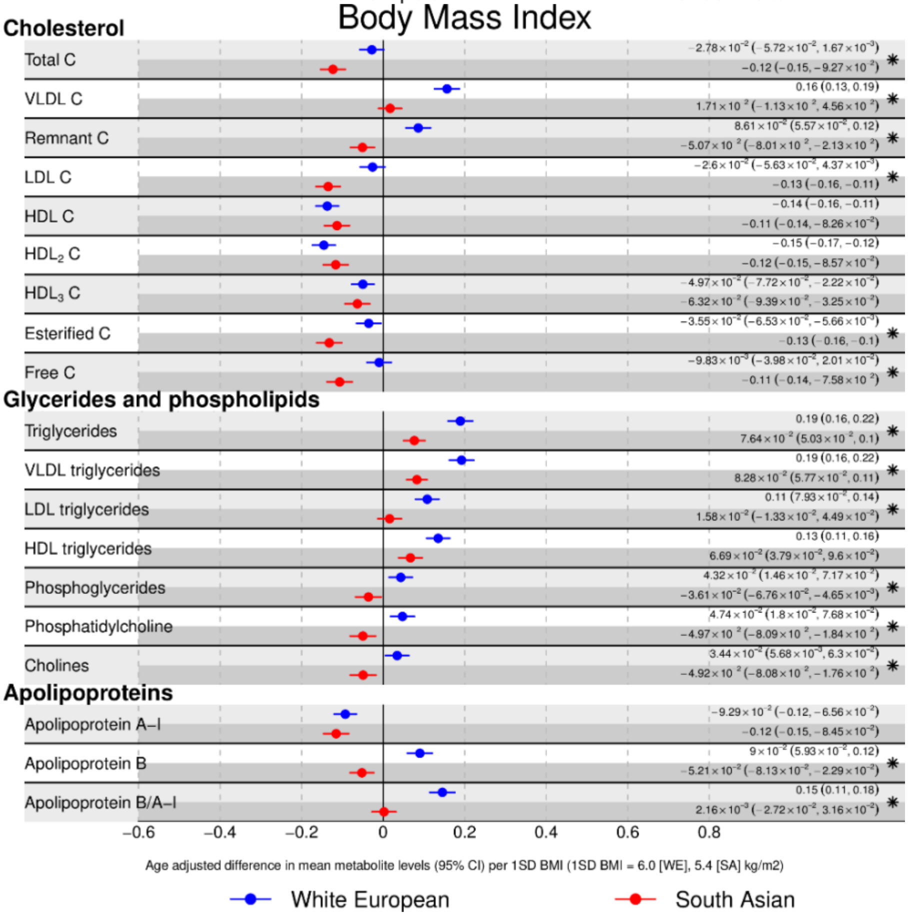
Body Mass Index
Cholesterol
Total C
VLDL C
Remnant C
LDL C
HDL C
HDL2 C
HDL3 C
Esterified C
Free C
Glycerides and phospholipids
Triglycerides
VLDL triglycerides
LDL triglycerides
HDL triglycerides
Phosphoglycerides
Phosphatidylcholine
Cholines
Apolipoproteins
Apolipoprotein A−I
Apolipoprotein B
Apolipoprotein B/A−I
−0.6 −0.4 −0.2 0 0.2 0.4 0.6 0.8
Age adjusted difference in mean metabolite levels (95% CI) per 1SD BMI (1SD BMI = 6.0 [WE], 5.4 [SA] kg/m2)
White European
South Asian

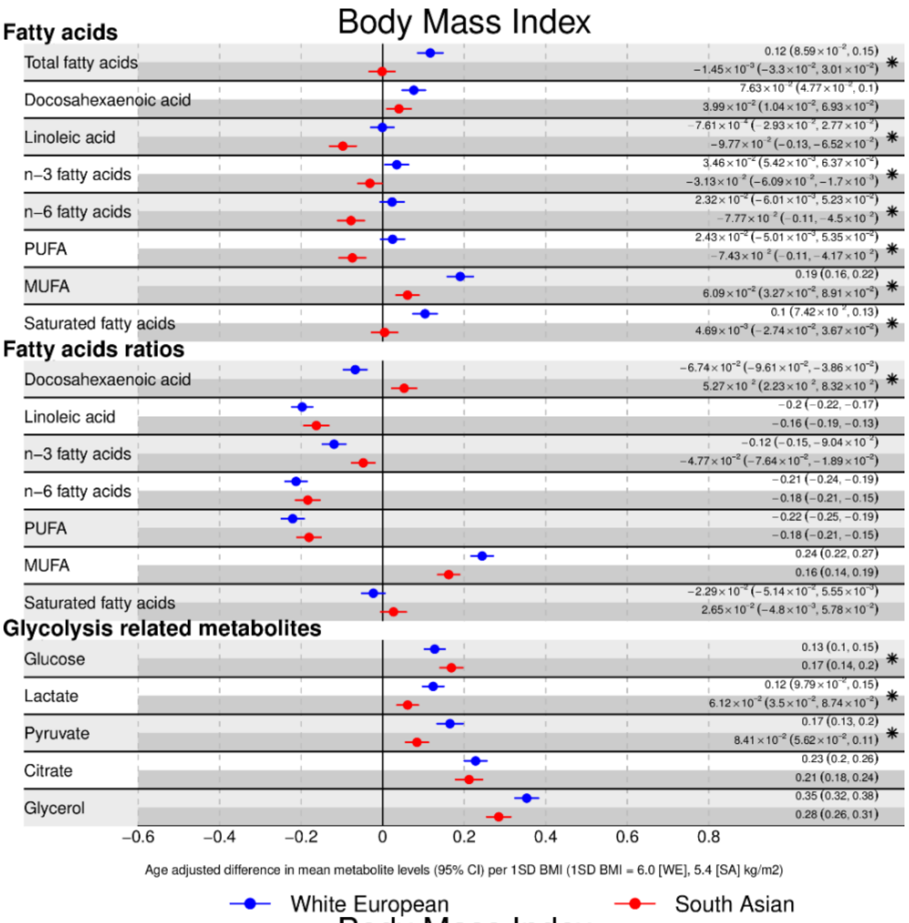

Body Mass Index
Fatty acids
Total fatty acids
0.12 (8.59×10^{-2}, 0.15)
-1.45×10^{-3} (-3.3×10^{-2}, 3.01×10^{-2}) *
Docosahexaenoic acid
7.63×10^{-2} (4.77×10^{-2}, 0.1)
3.99×10^{-2} (1.04×10^{-2}, 6.93×10^{-2})
Linoleic acid
-7.61×10^{-4} (-2.93×10^{-2}, 2.77×10^{-2})
-9.77×10^{-2} (-0.13, -6.52×10^{-2}) *
n−3 fatty acids
3.46×10^{-2} (5.42×10^{-3}, 6.37×10^{-2})
-3.13×10^{-2} (-6.09×10^{-2}, -1.7×10^{-3}) *
n−6 fatty acids
2.32×10^{-2} (-6.01×10^{-3}, 5.23×10^{-2})
-7.77×10^{-2} (-0.11, -4.5×10^{-2}) *
PUFA
2.43×10^{-2} (-5.01×10^{-3}, 5.35×10^{-2})
-7.43×10^{-2} (-0.11, -4.17×10^{-2}) *
MUFA
0.19 (0.16, 0.22)
6.09×10^{-2} (3.27×10^{-2}, 8.91×10^{-2}) *
Saturated fatty acids
0.1 (7.42×10^{-2}, 0.13)
4.69×10^{-3} (-2.74×10^{-2}, 3.67×10^{-2}) *
Fatty acids ratios
Docosahexaenoic acid
-6.74×10^{-2} (-9.61×10^{-2}, -3.86×10^{-2})
5.27×10^{-2} (2.23×10^{-2}, 8.32×10^{-2}) *
Linoleic acid
-0.2 (-0.22, -0.17)
-0.16 (-0.19, -0.13)
n−3 fatty acids
-0.12 (-0.15, -9.04×10^{-2})
-4.77×10^{-2} (-7.64×10^{-2}, -1.89×10^{-2})
n−6 fatty acids
-0.21 (-0.24, -0.19)
-0.18 (-0.21, -0.15)
PUFA
-0.22 (-0.25, -0.19)
-0.18 (-0.21, -0.15)
MUFA
0.24 (0.22, 0.27)
0.16 (0.14, 0.19)
Saturated fatty acids
-2.29×10^{-2} (-5.14×10^{-2}, 5.55×10^{-3})
2.65×10^{-2} (-4.8×10^{-3}, 5.78×10^{-2})
Glycolysis related metabolites
Glucose
0.13 (0.1, 0.15)
0.17 (0.14, 0.2) *
Lactate
0.12 (9.79×10^{-2}, 0.15)
6.12×10^{-2} (3.5×10^{-2}, 8.74×10^{-2}) *
Pyruvate
0.17 (0.13, 0.2)
8.41×10^{-2} (5.62×10^{-2}, 0.11) *
Citrate
0.23 (0.2, 0.26)
0.21 (0.18, 0.24)
Glycerol
0.35 (0.32, 0.38)
0.28 (0.26, 0.31)
−0.6 −0.4 −0.2 0 0.2 0.4 0.6 0.8
Age adjusted difference in mean metabolite levels (95% CI) per 1SD BMI (1SD BMI = 6.0 [WE], 5.4 [SA] kg/m2)
White European
South Asian

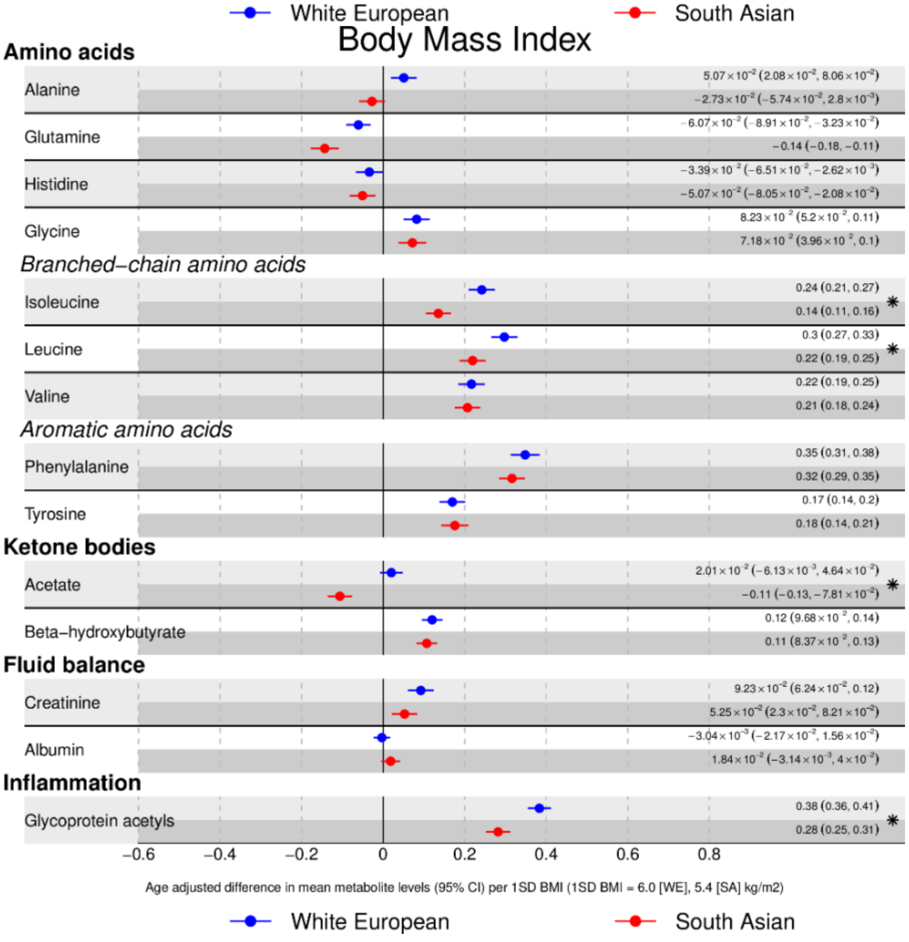

Body Mass Index
Amino acids
Alanine
5.07×10^{-2} (2.08×10^{-2}, 8.06×10^{-2})
-2.73×10^{-2} (-5.74×10^{-2}, 2.8×10^{-3})
Glutamine
-6.07×10^{-2} (-8.91×10^{-2}, -3.23×10^{-2})
-0.14 (-0.18, -0.11)
Histidine
-3.39×10^{-2} (-6.51×10^{-2}, -2.62×10^{-3})
-5.07×10^{-2} (-8.05×10^{-2}, -2.08×10^{-2})
Glycine
8.23×10^{-2} (5.2×10^{-2}, 0.11)
7.18×10^{-2} (3.96×10^{-2}, 0.1)
Branched−chain amino acids
Isoleucine
0.24 (0.21, 0.27)
0.14 (0.11, 0.16) *
Leucine
0.3 (0.27, 0.33)
0.22 (0.19, 0.25) *
Valine
0.22 (0.19, 0.25)
0.21 (0.18, 0.24)
Aromatic amino acids
Phenylalanine
0.35 (0.31, 0.38)
0.32 (0.29, 0.35)
Tyrosine
0.17 (0.14, 0.2)
0.18 (0.14, 0.21)
Ketone bodies
Acetate
2.01×10^{-2} (-6.13×10^{-3}, 4.64×10^{-2})
-0.11 (-0.13, -7.81×10^{-2}) *
Beta−hydroxybutyrate
0.12 (9.68×10^{-2}, 0.14)
0.11 (8.37×10^{-2}, 0.13)
Fluid balance
Creatinine
9.23×10^{-2} (6.24×10^{-2}, 0.12)
5.25×10^{-2} (2.3×10^{-2}, 8.21×10^{-2})
Albumin
-3.04×10^{-3} (-2.17×10^{-2}, 1.56×10^{-2})
1.84×10^{-2} (-3.14×10^{-3}, 4×10^{-2})
Inflammation
Glycoprotein acetyls
0.38 (0.36, 0.41)
0.28 (0.25, 0.31) *
−0.6 −0.4 −0.2 0 0.2 0.4 0.6 0.8
Age adjusted difference in mean metabolite levels (95% CI) per 1SD BMI (1SD BMI = 6.0 [WE], 5.4 [SA] kg/m2)
White European
South Asian

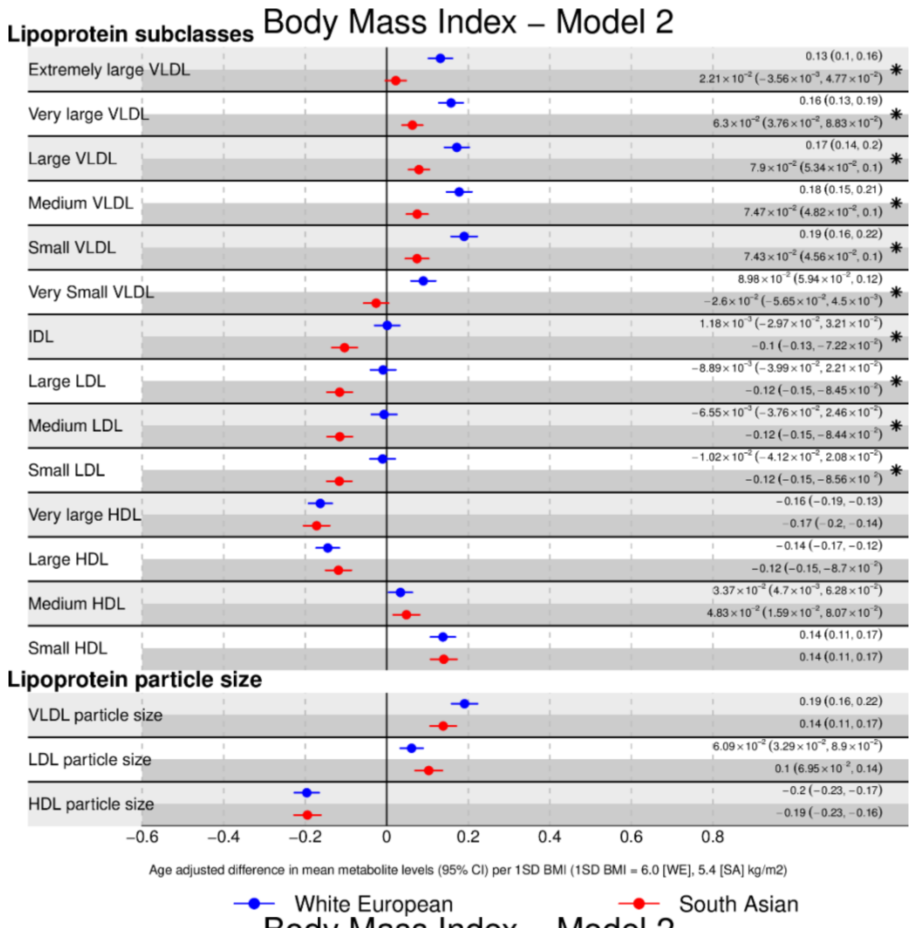

Body Mass Index – Model 2
Lipoprotein subclasses
Extremely large VLDL
Very large VLDL
Large VLDL
Medium VLDL
Small VLDL
Very Small VLDL
IDL
Large LDL
Medium LDL
Small LDL
Very large HDL
Large HDL
Medium HDL
Small HDL
Lipoprotein particle size
VLDL particle size
LDL particle size
HDL particle size
−0.6
−0.4
−0.2
0
0.2
0.4
0.6
0.8
Age adjusted difference in mean metabolite levels (95% CI) per 1SD BMI (1SD BMI = 6.0 [WE], 5.4 [SA] kg/m2)
White European
South Asian

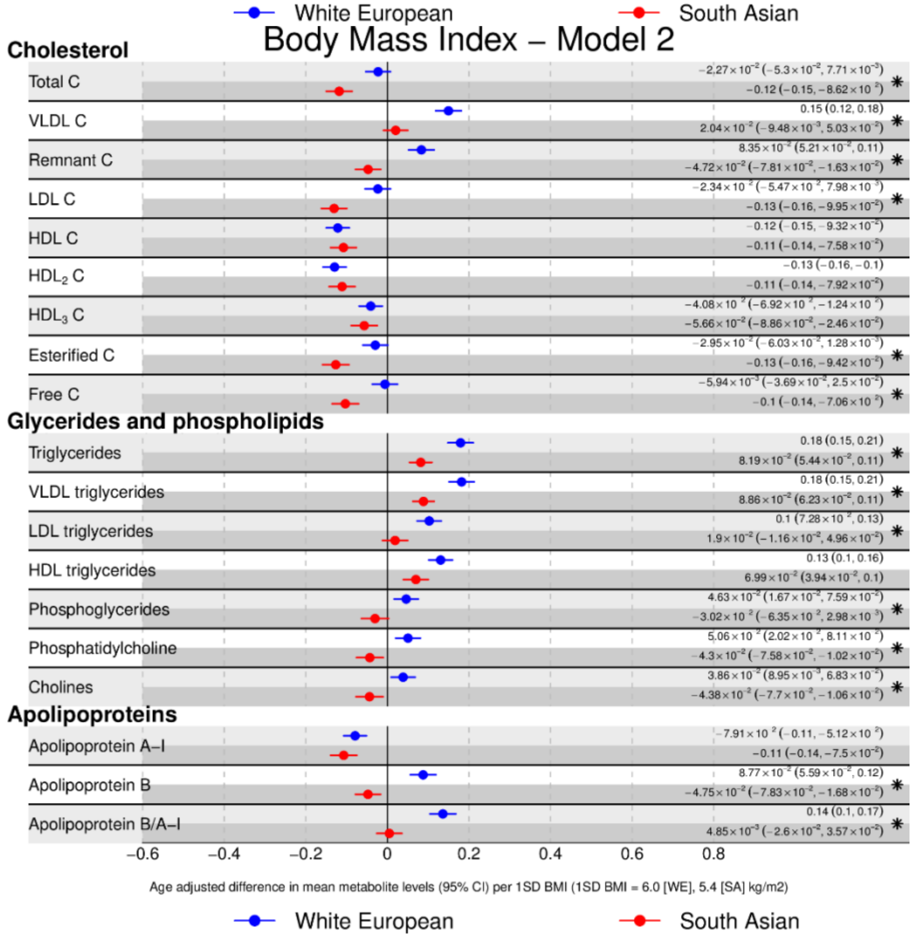

Body Mass Index – Model 2
Cholesterol
Total C
VLDL C
Remnant C
LDL C
HDL C
HDL2 C
HDL3 C
Esterified C
Free C
Glycerides and phospholipids
Triglycerides
VLDL triglycerides
LDL triglycerides
HDL triglycerides
Phosphoglycerides
Phosphatidylcholine
Cholines
Apolipoproteins
Apolipoprotein A–I
Apolipoprotein B
Apolipoprotein B/A–I
−0.6
−0.4
−0.2
0
0.2
0.4
0.6
0.8
Age adjusted difference in mean metabolite levels (95% CI) per 1SD BMI (1SD BMI = 6.0 [WE], 5.4 [SA] kg/m2)
White European
South Asian

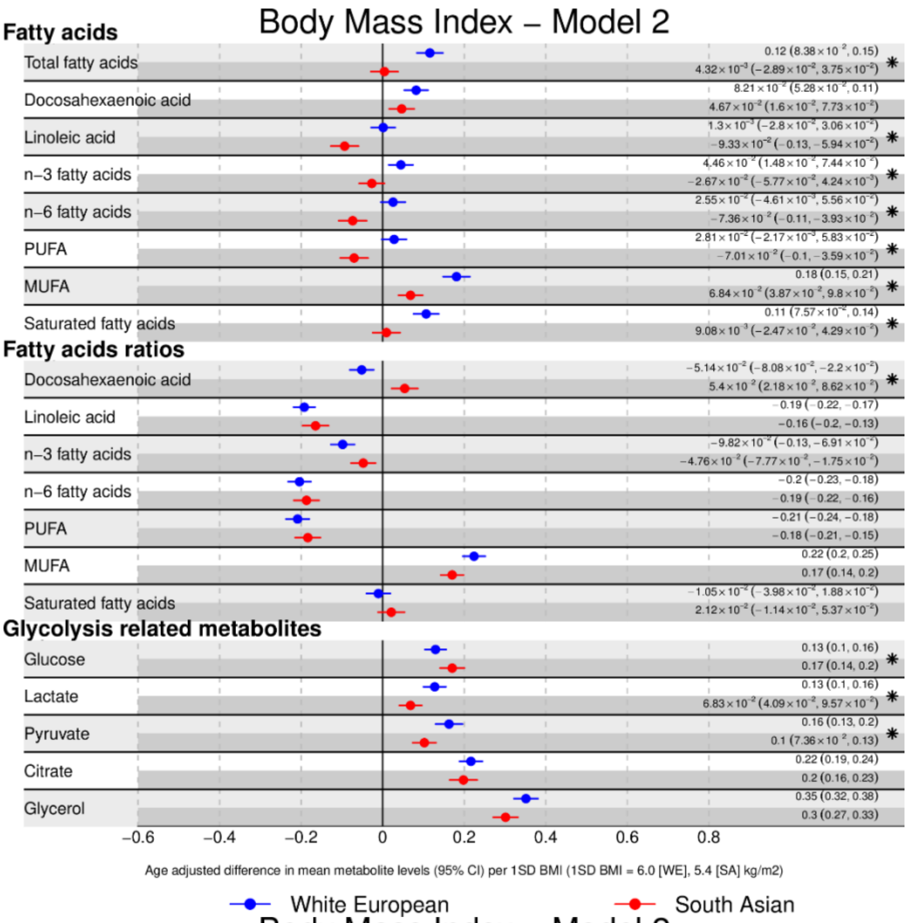

Body Mass Index – Model 2
Fatty acids
Total fatty acids
0.12 (8.38×10^-2, 0.15)
4.32×10^-3 (−2.89×10^-2, 3.75×10^-2) *
Docosahexaenoic acid
8.21×10^-2 (5.28×10^-2, 0.11)
4.67×10^-2 (1.6×10^-2, 7.73×10^-2)
Linoleic acid
1.3×10^-3 (−2.8×10^-2, 3.06×10^-2)
−9.33×10^-2 (−0.13, −5.94×10^-2) *
n−3 fatty acids
4.46×10^-2 (1.48×10^-2, 7.44×10^-2)
−2.67×10^-2 (−5.77×10^-2, 4.24×10^-3) *
n−6 fatty acids
2.55×10^-2 (−4.61×10^-3, 5.56×10^-2)
−7.36×10^-2 (−0.11, −3.93×10^-2) *
PUFA
2.81×10^-2 (−2.17×10^-3, 5.83×10^-2)
−7.01×10^-2 (−0.1, −3.59×10^-2) *
MUFA
0.18 (0.15, 0.21)
6.84×10^-2 (3.87×10^-2, 9.8×10^-2) *
Saturated fatty acids
0.11 (7.57×10^-2, 0.14)
9.08×10^-3 (−2.47×10^-2, 4.29×10^-2) *
Fatty acids ratios
Docosahexaenoic acid
−5.14×10^-2 (−8.08×10^-2, −2.2×10^-2)
5.4×10^-2 (2.18×10^-2, 8.62×10^-2) *
Linoleic acid
−0.19 (−0.22, −0.17)
−0.16 (−0.2, −0.13)
n−3 fatty acids
−9.82×10^-2 (−0.13, −6.91×10^-2)
−4.76×10^-2 (−7.77×10^-2, −1.75×10^-2)
n−6 fatty acids
−0.2 (−0.23, −0.18)
−0.19 (−0.22, −0.16)
PUFA
−0.21 (−0.24, −0.18)
−0.18 (−0.21, −0.15)
MUFA
0.22 (0.2, 0.25)
0.17 (0.14, 0.2)
Saturated fatty acids
−1.05×10^-2 (−3.98×10^-2, 1.88×10^-2)
2.12×10^-2 (−1.14×10^-2, 5.37×10^-2)
Glycolysis related metabolites
Glucose
0.13 (0.1, 0.16)
0.17 (0.14, 0.2) *
Lactate
0.13 (0.1, 0.16)
6.83×10^-2 (4.09×10^-2, 9.57×10^-2) *
Pyruvate
0.16 (0.13, 0.2)
0.1 (7.36×10^-2, 0.13) *
Citrate
0.22 (0.19, 0.24)
0.2 (0.16, 0.23)
Glycerol
0.35 (0.32, 0.38)
0.3 (0.27, 0.33)
−0.6 −0.4 −0.2 0 0.2 0.4 0.6 0.8
Age adjusted difference in mean metabolite levels (95% CI) per 1SD BMI (1SD BMI = 6.0 [WE], 5.4 [SA] kg/m2)
White European
South Asian

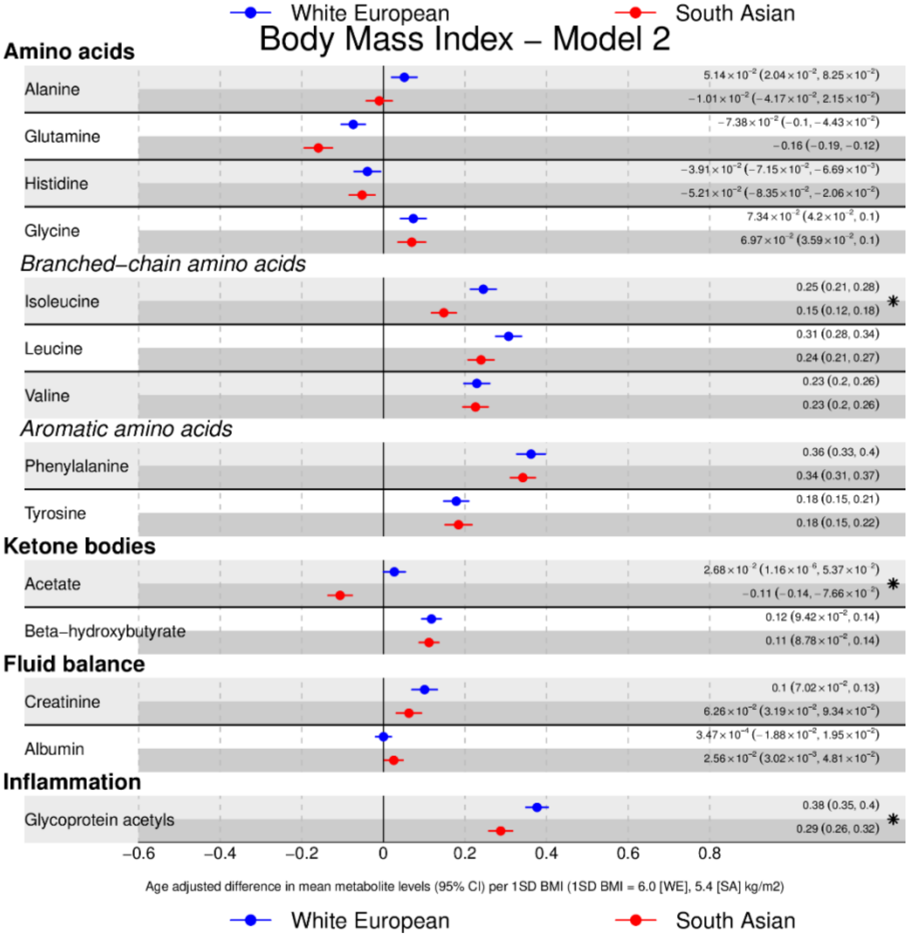

Body Mass Index – Model 2
Amino acids
Alanine
5.14×10^-2 (2.04×10^-2, 8.25×10^-2)
−1.01×10^-2 (−4.17×10^-2, 2.15×10^-2)
Glutamine
−7.38×10^-2 (−0.1, −4.43×10^-2)
−0.16 (−0.19, −0.12)
Histidine
−3.91×10^-2 (−7.15×10^-2, −6.69×10^-3)
−5.21×10^-2 (−8.35×10^-2, −2.06×10^-2)
Glycine
7.34×10^-2 (4.2×10^-2, 0.1)
6.97×10^-2 (3.59×10^-2, 0.1)
Branched−chain amino acids
Isoleucine
0.25 (0.21, 0.28)
0.15 (0.12, 0.18) *
Leucine
0.31 (0.28, 0.34)
0.24 (0.21, 0.27)
Valine
0.23 (0.2, 0.26)
0.23 (0.2, 0.26)
Aromatic amino acids
Phenylalanine
0.36 (0.33, 0.4)
0.34 (0.31, 0.37)
Tyrosine
0.18 (0.15, 0.21)
0.18 (0.15, 0.22)
Ketone bodies
Acetate
2.68×10^-2 (1.16×10^-6, 5.37×10^-2)
−0.11 (−0.14, −7.66×10^-2) *
Beta−hydroxybutyrate
0.12 (9.42×10^-2, 0.14)
0.11 (8.78×10^-2, 0.14)
Fluid balance
Creatinine
0.1 (7.02×10^-2, 0.13)
6.26×10^-2 (3.19×10^-2, 9.34×10^-2)
Albumin
3.47×10^-4 (−1.88×10^-2, 1.95×10^-2)
2.56×10^-2 (3.02×10^-3, 4.81×10^-2)
Inflammation
Glycoprotein acetyls
0.38 (0.35, 0.4)
0.29 (0.26, 0.32) *
−0.6 −0.4 −0.2 0 0.2 0.4 0.6 0.8
Age adjusted difference in mean metabolite levels (95% CI) per 1SD BMI (1SD BMI = 6.0 [WE], 5.4 [SA] kg/m2)
White European
South Asian

Figure S8. Associations of maternal body mass index with pregnancy metabolic profiles stratified by ethnicity (model 1 = age-adjusted; model 2 = age-, parity- and education-adjusted). Data points show SD differences for White European (blue) and South Asian (red) women. Error bars = 95% confidence intervals. Exact point estimates and their corresponding 95% CIs are displayed in text to the right of each point. * denotes strong statistical evidence from the interaction test (Pinteraction <0.001). Differences in quantified units (mostly mmol/l) are listed in Supplementary File 2. Abbreviations: VLDL, very low-density lipoprotein; LDL, low-density lipoprotein; HDL, high-density lipoprotein; C, cholesterol; MUFA, monounsaturated fatty acids; PUFA, polyunsaturated fatty acids

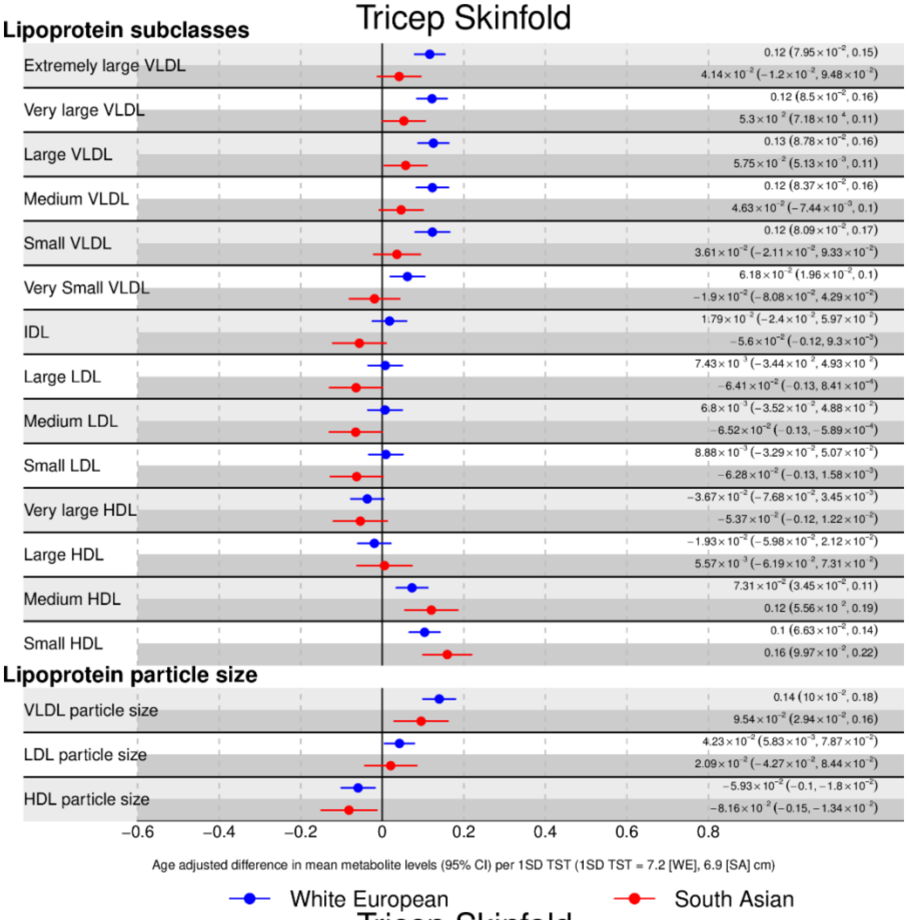
Tricep Skinfold
Lipoprotein subclasses
Extremely large VLDL
0.12 (7.95×10^-2, 0.15)
4.14×10^-2 (−1.2×10^-2, 9.48×10^-2)
Very large VLDL
0.12 (8.5×10^-2, 0.16)
5.3×10^-2 (7.18×10^-4, 0.11)
Large VLDL
0.13 (8.78×10^-2, 0.16)
5.75×10^-2 (5.13×10^-3, 0.11)
Medium VLDL
0.12 (8.37×10^-2, 0.16)
4.63×10^-2 (−7.44×10^-3, 0.1)
Small VLDL
0.12 (8.09×10^-2, 0.17)
3.61×10^-2 (−2.11×10^-2, 9.33×10^-2)
Very Small VLDL
6.18×10^-2 (1.96×10^-2, 0.1)
−1.9×10^-2 (−8.08×10^-2, 4.29×10^-2)
IDL
1.79×10^-2 (−2.4×10^-2, 5.97×10^-2)
−5.6×10^-2 (−0.12, 9.3×10^-3)
Large LDL
7.43×10^-3 (−3.44×10^-2, 4.93×10^-2)
−6.41×10^-2 (−0.13, 8.41×10^-4)
Medium LDL
6.8×10^-3 (−3.52×10^-2, 4.88×10^-2)
−6.52×10^-2 (−0.13, −5.89×10^-4)
Small LDL
8.88×10^-3 (−3.29×10^-2, 5.07×10^-2)
−6.28×10^-2 (−0.13, 1.58×10^-3)
Very large HDL
−3.67×10^-2 (−7.68×10^-2, 3.45×10^-3)
−5.37×10^-2 (−0.12, 1.22×10^-2)
Large HDL
−1.93×10^-2 (−5.98×10^-2, 2.12×10^-2)
5.57×10^-3 (−6.19×10^-2, 7.31×10^-2)
Medium HDL
7.31×10^-2 (3.45×10^-2, 0.11)
0.12 (5.56×10^-2, 0.19)
Small HDL
0.1 (6.63×10^-2, 0.14)
0.16 (9.97×10^-2, 0.22)
Lipoprotein particle size
VLDL particle size
0.14 (10×10^-2, 0.18)
9.54×10^-2 (2.94×10^-2, 0.16)
LDL particle size
4.23×10^-2 (5.83×10^-3, 7.87×10^-2)
2.09×10^-2 (−4.27×10^-2, 8.44×10^-2)
HDL particle size
−5.93×10^-2 (−0.1, −1.8×10^-2)
−8.16×10^-2 (−0.15, −1.34×10^-2)
−0.6 −0.4 −0.2 0 0.2 0.4 0.6 0.8
Age adjusted difference in mean metabolite levels (95% CI) per 1SD TST (1SD TST = 7.2 [WE], 6.9 [SA] cm)
White European
South Asian

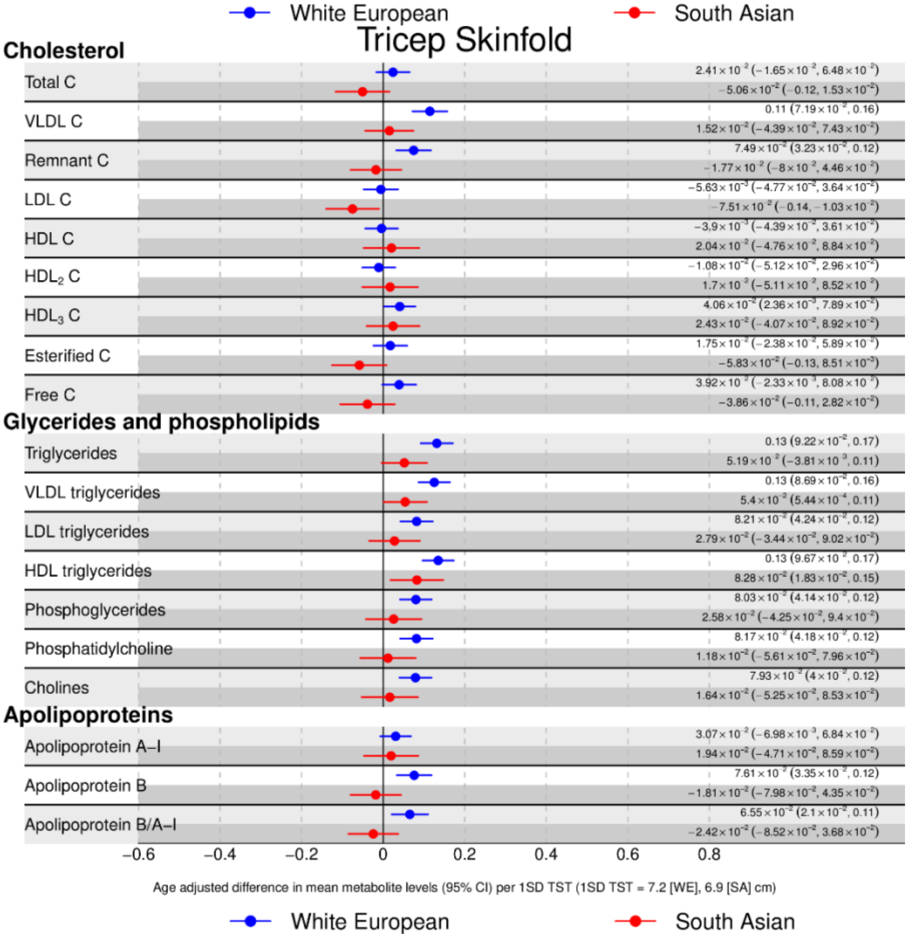
Tricep Skinfold
Cholesterol
Total C
2.41×10^-2 (−1.65×10^-2, 6.48×10^-2)
−5.06×10^-2 (−0.12, 1.53×10^-2)
VLDL C
0.11 (7.19×10^-2, 0.16)
1.52×10^-2 (−4.39×10^-2, 7.43×10^-2)
Remnant C
7.49×10^-2 (3.23×10^-2, 0.12)
−1.77×10^-2 (−8×10^-2, 4.46×10^-2)
LDL C
−5.63×10^-3 (−4.77×10^-2, 3.64×10^-2)
−7.51×10^-2 (−0.14, −1.03×10^-2)
HDL C
−3.9×10^-3 (−4.39×10^-2, 3.61×10^-2)
2.04×10^-2 (−4.76×10^-2, 8.84×10^-2)
HDL2 C
−1.08×10^-2 (−5.12×10^-2, 2.96×10^-2)
1.7×10^-2 (−5.11×10^-2, 8.52×10^-2)
HDL3 C
4.06×10^-2 (2.36×10^-3, 7.89×10^-2)
2.43×10^-2 (−4.07×10^-2, 8.92×10^-2)
Esterified C
1.75×10^-2 (−2.38×10^-2, 5.89×10^-2)
−5.83×10^-2 (−0.13, 8.51×10^-3)
Free C
3.92×10^-2 (−2.33×10^-3, 8.08×10^-2)
−3.86×10^-2 (−0.11, 2.82×10^-2)
Glycerides and phospholipids
Triglycerides
0.13 (9.22×10^-2, 0.17)
5.19×10^-2 (−3.81×10^-3, 0.11)
VLDL triglycerides
0.13 (8.69×10^-2, 0.16)
5.4×10^-2 (5.44×10^-4, 0.11)
LDL triglycerides
8.21×10^-2 (4.24×10^-2, 0.12)
2.79×10^-2 (−3.44×10^-2, 9.02×10^-2)
HDL triglycerides
0.13 (9.67×10^-2, 0.17)
8.28×10^-2 (1.83×10^-2, 0.15)
Phosphoglycerides
8.03×10^-2 (4.14×10^-2, 0.12)
2.58×10^-2 (−4.25×10^-2, 9.4×10^-2)
Phosphatidylcholine
8.17×10^-2 (4.18×10^-2, 0.12)
1.18×10^-2 (−5.61×10^-2, 7.96×10^-2)
Cholines
7.93×10^-2 (4×10^-2, 0.12)
1.64×10^-2 (−5.25×10^-2, 8.53×10^-2)
Apolipoproteins
Apolipoprotein A–I
3.07×10^-2 (−6.98×10^-3, 6.84×10^-2)
1.94×10^-2 (−4.71×10^-2, 8.59×10^-2)
Apolipoprotein B
7.61×10^-2 (3.35×10^-2, 0.12)
−1.81×10^-2 (−7.98×10^-2, 4.35×10^-2)
Apolipoprotein B/A–I
6.55×10^-2 (2.1×10^-2, 0.11)
−2.42×10^-2 (−8.52×10^-2, 3.68×10^-2)
−0.6 −0.4 −0.2 0 0.2 0.4 0.6 0.8
Age adjusted difference in mean metabolite levels (95% CI) per 1SD TST (1SD TST = 7.2 [WE], 6.9 [SA] cm)
White European
South Asian

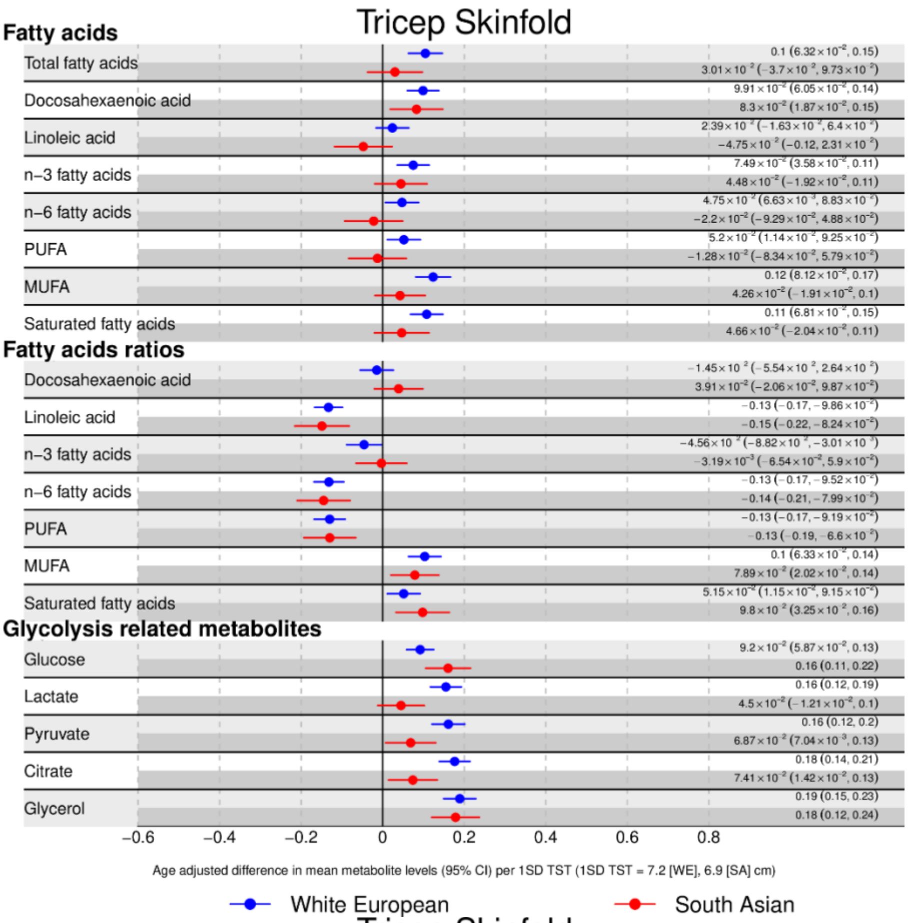
Tricep Skinfold
Fatty acids
Total fatty acids
Docosahexaenoic acid
Linoleic acid
n−3 fatty acids
n−6 fatty acids
PUFA
MUFA
Saturated fatty acids
Fatty acids ratios
Docosahexaenoic acid
Linoleic acid
n−3 fatty acids
n−6 fatty acids
PUFA
MUFA
Saturated fatty acids
Glycolysis related metabolites
Glucose
Lactate
Pyruvate
Citrate
Glycerol
−0.6
−0.4
−0.2
0
0.2
0.4
0.6
0.8
Age adjusted difference in mean metabolite levels (95% CI) per 1SD TST (1SD TST = 7.2 [WE], 6.9 [SA] cm)
White European
South Asian

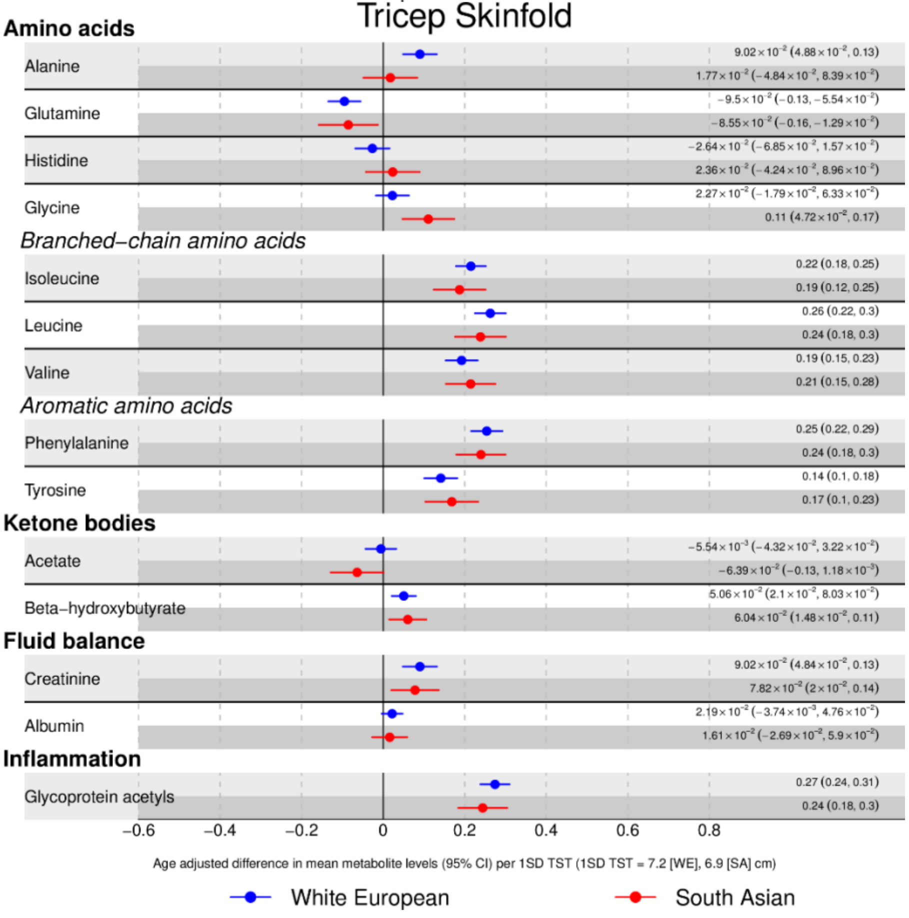
Tricep Skinfold
Amino acids
Alanine
Glutamine
Histidine
Glycine
Branched−chain amino acids
Isoleucine
Leucine
Valine
Aromatic amino acids
Phenylalanine
Tyrosine
Ketone bodies
Acetate
Beta−hydroxybutyrate
Fluid balance
Creatinine
Albumin
Inflammation
Glycoprotein acetyls
−0.6
−0.4
−0.2
0
0.2
0.4
0.6
0.8
Age adjusted difference in mean metabolite levels (95% CI) per 1SD TST (1SD TST = 7.2 [WE], 6.9 [SA] cm)
White European
South Asian

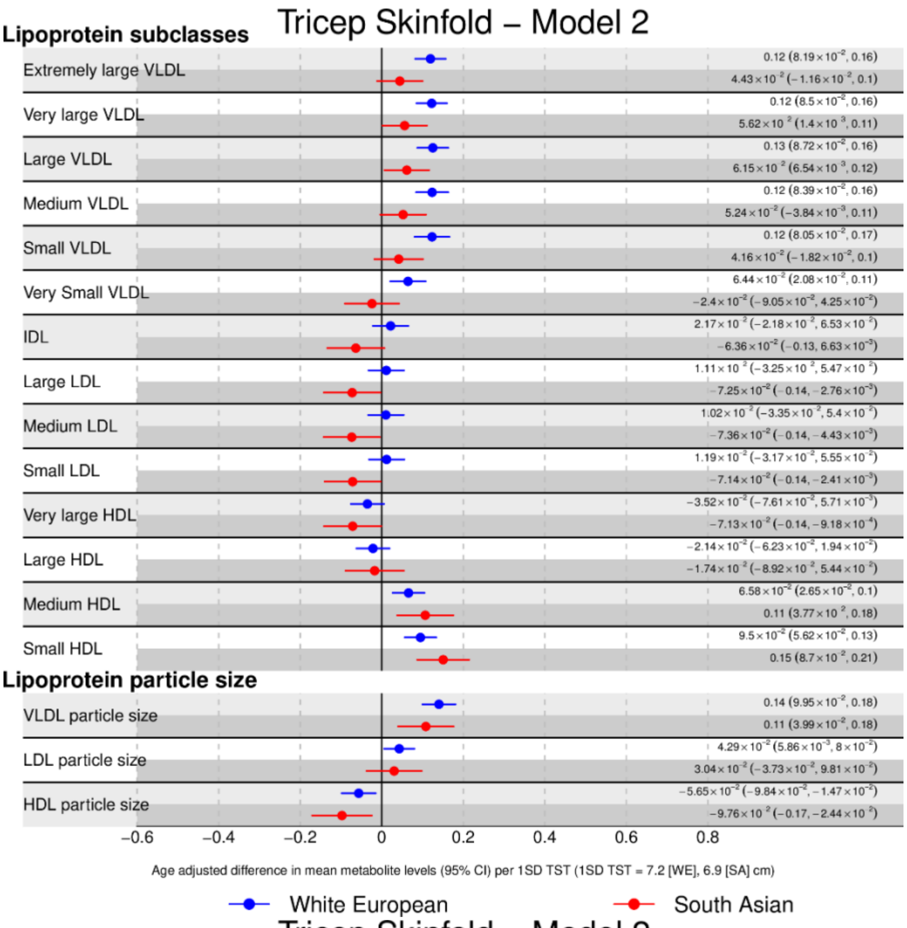

Tricep Skinfold – Model 2
Lipoprotein subclasses
Extremely large VLDL
Very large VLDL
Large VLDL
Medium VLDL
Small VLDL
Very Small VLDL
IDL
Large LDL
Medium LDL
Small LDL
Very large HDL
Large HDL
Medium HDL
Small HDL
Lipoprotein particle size
VLDL particle size
LDL particle size
HDL particle size
−0.6 −0.4 −0.2 0 0.2 0.4 0.6 0.8
Age adjusted difference in mean metabolite levels (95% CI) per 1SD TST (1SD TST = 7.2 [WE], 6.9 [SA] cm)
White European
South Asian

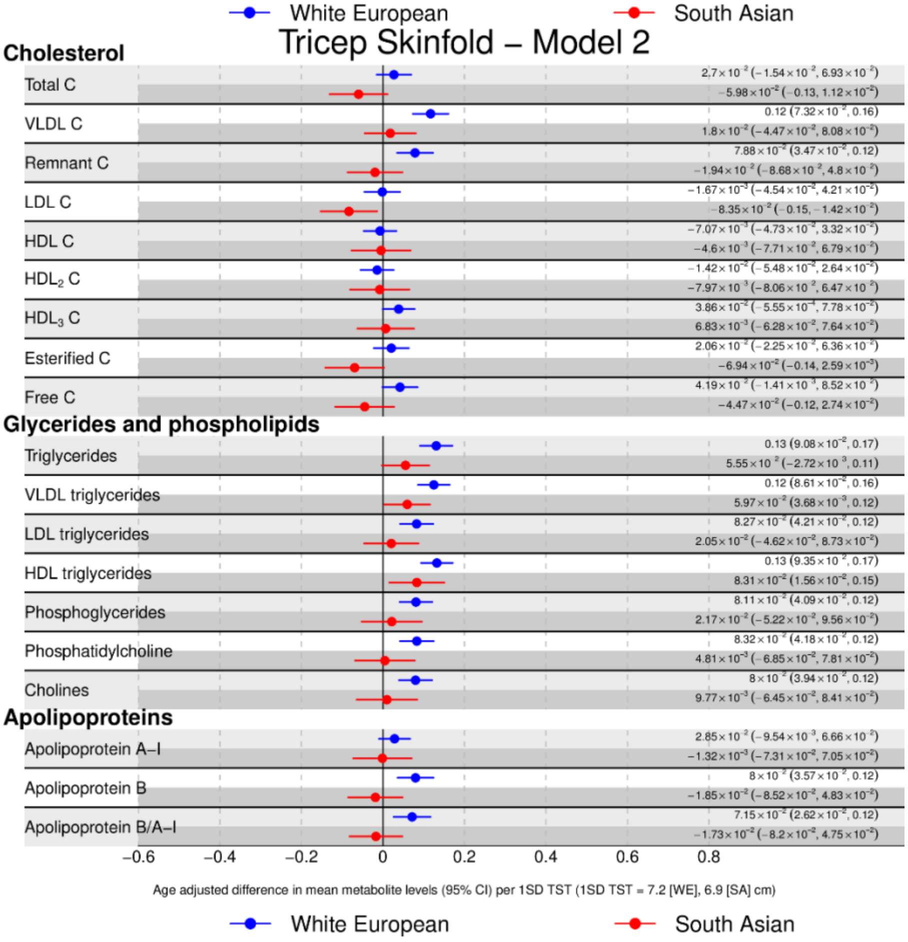

Tricep Skinfold – Model 2
Cholesterol
Total C
VLDL C
Remnant C
LDL C
HDL C
HDL2 C
HDL3 C
Esterified C
Free C
Glycerides and phospholipids
Triglycerides
VLDL triglycerides
LDL triglycerides
HDL triglycerides
Phosphoglycerides
Phosphatidylcholine
Cholines
Apolipoproteins
Apolipoprotein A–I
Apolipoprotein B
Apolipoprotein B/A–I
−0.6 −0.4 −0.2 0 0.2 0.4 0.6 0.8
Age adjusted difference in mean metabolite levels (95% CI) per 1SD TST (1SD TST = 7.2 [WE], 6.9 [SA] cm)
White European
South Asian

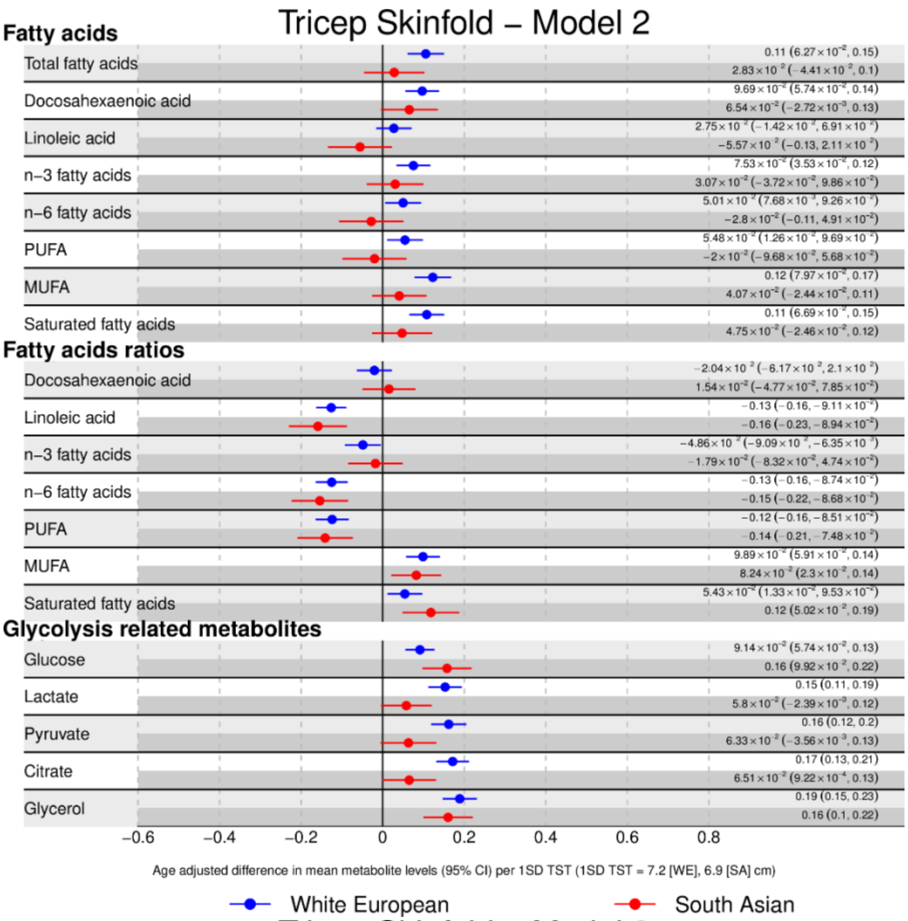

Tricep Skinfold – Model 2
Fatty acids
Total fatty acids
0.11 (6.27×10⁻², 0.15)
2.83×10⁻² (−4.41×10⁻², 0.1)
Docosahexaenoic acid
9.69×10⁻² (5.74×10⁻², 0.14)
6.54×10⁻² (−2.72×10⁻³, 0.13)
Linoleic acid
2.75×10⁻² (−1.42×10⁻², 6.91×10⁻²)
−5.57×10⁻² (−0.13, 2.11×10⁻²)
n−3 fatty acids
7.53×10⁻² (3.53×10⁻², 0.12)
3.07×10⁻² (−3.72×10⁻², 9.86×10⁻²)
n−6 fatty acids
5.01×10⁻² (7.68×10⁻³, 9.26×10⁻²)
−2.8×10⁻² (−0.11, 4.91×10⁻²)
PUFA
5.48×10⁻² (1.26×10⁻², 9.69×10⁻²)
−2×10⁻² (−9.68×10⁻², 5.68×10⁻²)
MUFA
0.12 (7.97×10⁻², 0.17)
4.07×10⁻² (−2.44×10⁻², 0.11)
Saturated fatty acids
0.11 (6.69×10⁻², 0.15)
4.75×10⁻² (−2.46×10⁻², 0.12)
Fatty acids ratios
Docosahexaenoic acid
−2.04×10⁻² (−6.17×10⁻², 2.1×10⁻²)
1.54×10⁻² (−4.77×10⁻², 7.85×10⁻²)
Linoleic acid
−0.13 (−0.16, −9.11×10⁻²)
−0.16 (−0.23, −8.94×10⁻²)
n−3 fatty acids
−4.86×10⁻² (−9.09×10⁻², −6.35×10⁻³)
−1.79×10⁻² (−8.32×10⁻², 4.74×10⁻²)
n−6 fatty acids
−0.13 (−0.16, −8.74×10⁻²)
−0.15 (−0.22, −8.68×10⁻²)
PUFA
−0.12 (−0.16, −8.51×10⁻²)
−0.14 (−0.21, −7.48×10⁻²)
MUFA
9.89×10⁻² (5.91×10⁻², 0.14)
8.24×10⁻² (2.3×10⁻², 0.14)
Saturated fatty acids
5.43×10⁻² (1.33×10⁻², 9.53×10⁻²)
0.12 (5.02×10⁻², 0.19)
Glycolysis related metabolites
Glucose
9.14×10⁻² (5.74×10⁻², 0.13)
0.16 (9.92×10⁻², 0.22)
Lactate
0.15 (0.11, 0.19)
5.8×10⁻² (−2.39×10⁻³, 0.12)
Pyruvate
0.16 (0.12, 0.2)
6.33×10⁻² (−3.56×10⁻³, 0.13)
Citrate
0.17 (0.13, 0.21)
6.51×10⁻² (9.22×10⁻⁴, 0.13)
Glycerol
0.19 (0.15, 0.23)
0.16 (0.1, 0.22)
−0.6
−0.4
−0.2
0
0.2
0.4
0.6
0.8
Age adjusted difference in mean metabolite levels (95% CI) per 1SD TST (1SD TST = 7.2 [WE], 6.9 [SA] cm)
White European
South Asian

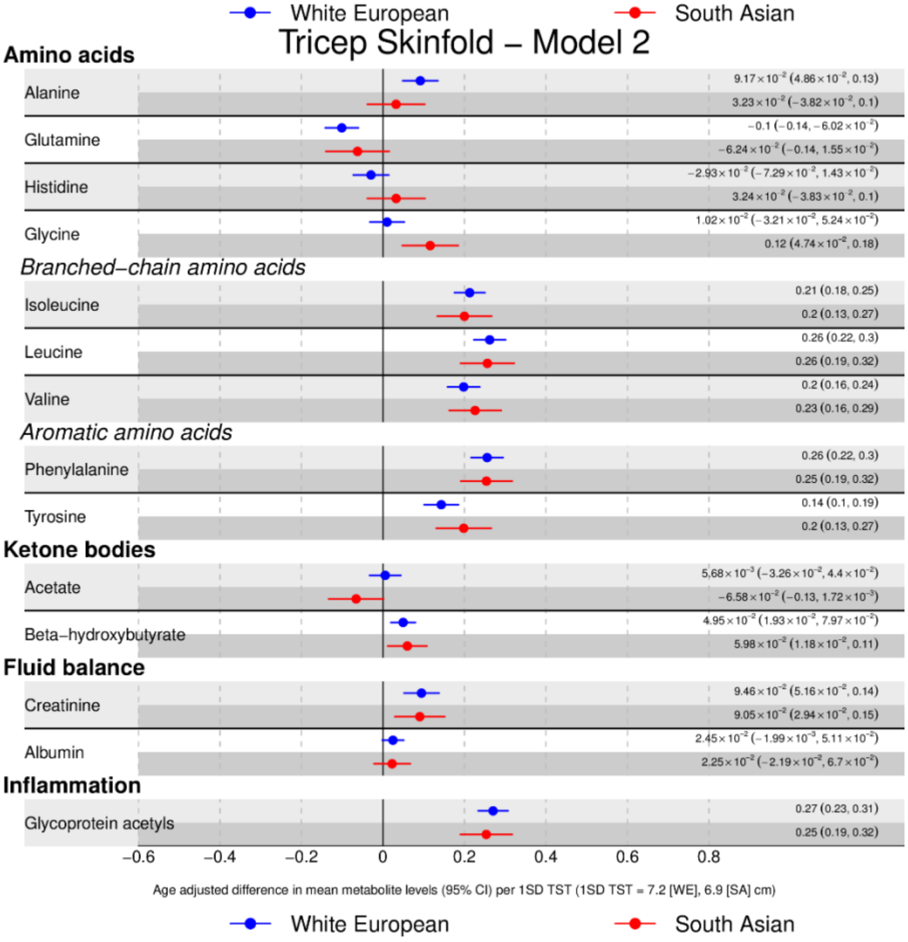

Tricep Skinfold – Model 2
Amino acids
Alanine
9.17×10⁻² (4.86×10⁻², 0.13)
3.23×10⁻² (−3.82×10⁻², 0.1)
Glutamine
−0.1 (−0.14, −6.02×10⁻²)
−6.24×10⁻² (−0.14, 1.55×10⁻²)
Histidine
−2.93×10⁻² (−7.29×10⁻², 1.43×10⁻²)
3.24×10⁻² (−3.83×10⁻², 0.1)
Glycine
1.02×10⁻² (−3.21×10⁻², 5.24×10⁻²)
0.12 (4.74×10⁻², 0.18)
Branched−chain amino acids
Isoleucine
0.21 (0.18, 0.25)
0.2 (0.13, 0.27)
Leucine
0.26 (0.22, 0.3)
0.26 (0.19, 0.32)
Valine
0.2 (0.16, 0.24)
0.23 (0.16, 0.29)
Aromatic amino acids
Phenylalanine
0.26 (0.22, 0.3)
0.25 (0.19, 0.32)
Tyrosine
0.14 (0.1, 0.19)
0.2 (0.13, 0.27)
Ketone bodies
Acetate
5.68×10⁻³ (−3.26×10⁻², 4.4×10⁻²)
−6.58×10⁻² (−0.13, 1.72×10⁻³)
Beta−hydroxybutyrate
4.95×10⁻² (1.93×10⁻², 7.97×10⁻²)
5.98×10⁻² (1.18×10⁻², 0.11)
Fluid balance
Creatinine
9.46×10⁻² (5.16×10⁻², 0.14)
9.05×10⁻² (2.94×10⁻², 0.15)
Albumin
2.45×10⁻² (−1.99×10⁻³, 5.11×10⁻²)
2.25×10⁻² (−2.19×10⁻², 6.7×10⁻²)
Inflammation
Glycoprotein acetyls
0.27 (0.23, 0.31)
0.25 (0.19, 0.32)
−0.6
−0.4
−0.2
0
0.2
0.4
0.6
0.8
Age adjusted difference in mean metabolite levels (95% CI) per 1SD TST (1SD TST = 7.2 [WE], 6.9 [SA] cm)
White European
South Asian

Figure S9. Associations of tricep skinfold thickness with pregnancy metabolic profiles stratified by ethnicity (model 1 = age-adjusted; model 2 = age-, parity- and education-adjusted). Data points show SD differences for White European (blue) and South Asian (red) women. Error bars = 95% confidence intervals. Exact point estimates and their corresponding 95% CIs are displayed in text to the right of each point. * denotes strong statistical evidence from the interaction test (Pinteraction <0.001). Differences in quantified units (mostly mmol/l) are listed in Supplementary File 2. Abbreviations: VLDL, very low-density lipoprotein; LDL, low-density lipoprotein; HDL, high-density lipoprotein; C, cholesterol; MUFA, monounsaturated fatty acids; PUFA, polyunsaturated fatty acids

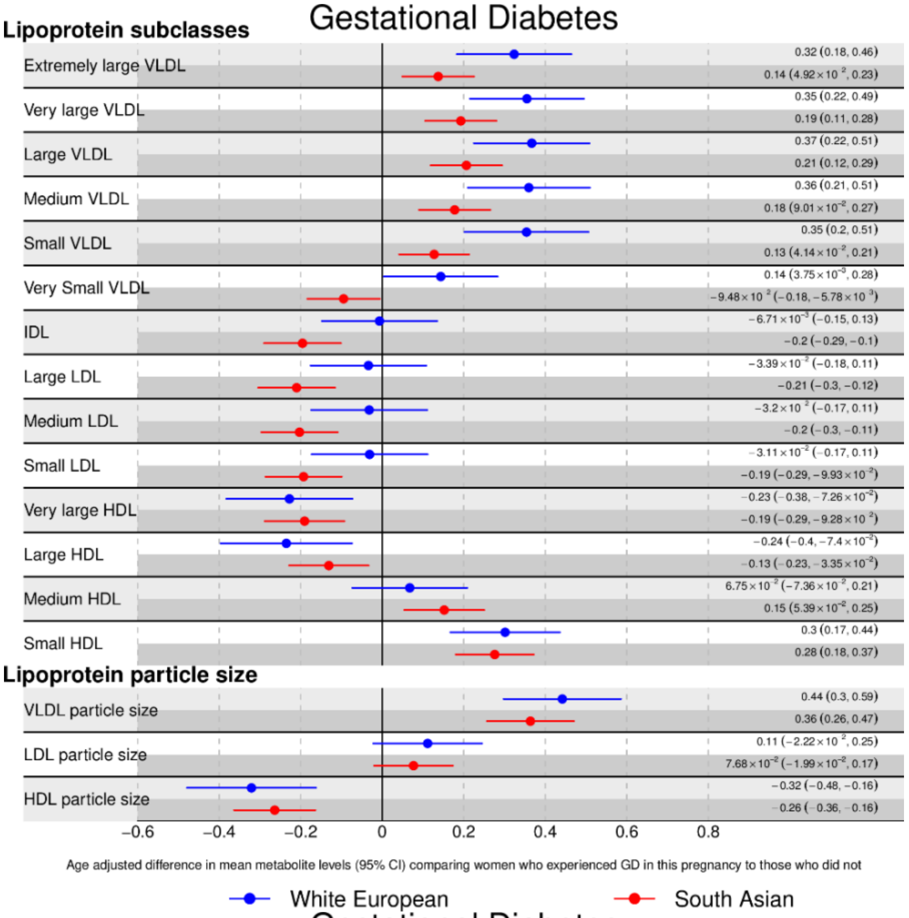
Gestational Diabetes
Lipoprotein subclasses
Extremely large VLDL
Very large VLDL
Large VLDL
Medium VLDL
Small VLDL
Very Small VLDL
IDL
Large LDL
Medium LDL
Small LDL
Very large HDL
Large HDL
Medium HDL
Small HDL
Lipoprotein particle size
VLDL particle size
LDL particle size
HDL particle size
−0.6 −0.4 −0.2 0 0.2 0.4 0.6 0.8
Age adjusted difference in mean metabolite levels (95% CI) comparing women who experienced GD in this pregnancy to those who did not
White European
South Asian

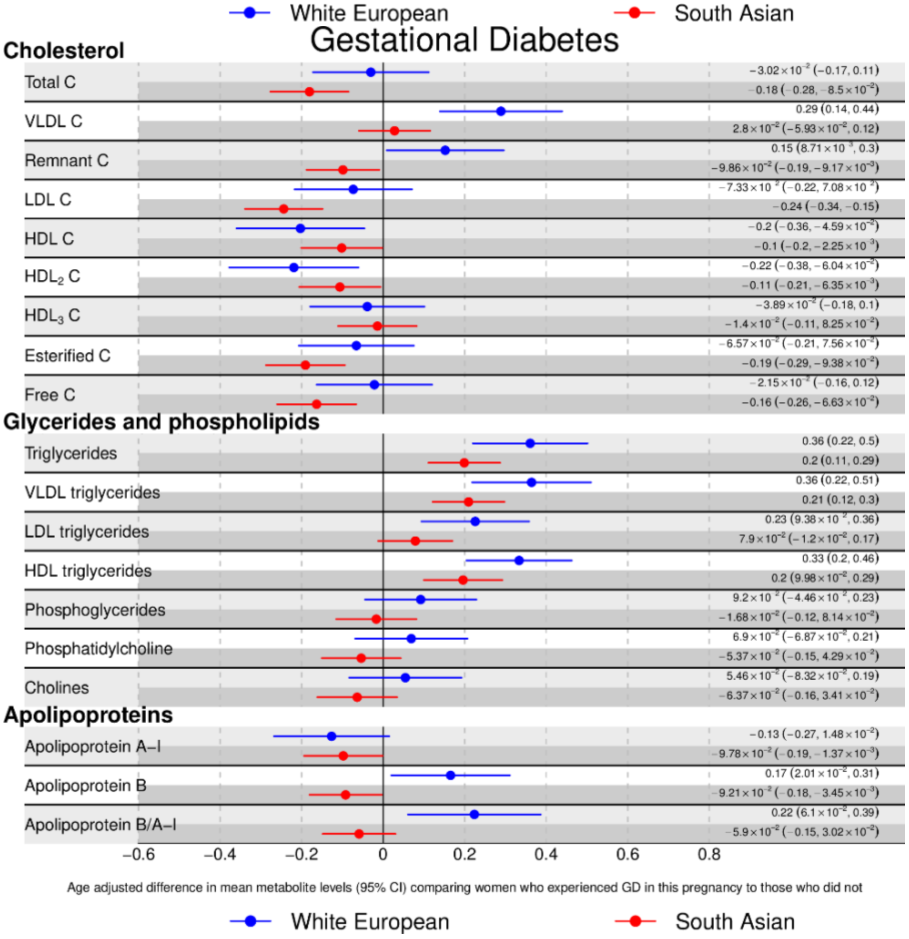
Gestational Diabetes
Cholesterol
Total C
VLDL C
Remnant C
LDL C
HDL C
HDL2 C
HDL3 C
Esterified C
Free C
Glycerides and phospholipids
Triglycerides
VLDL triglycerides
LDL triglycerides
HDL triglycerides
Phosphoglycerides
Phosphatidylcholine
Cholines
Apolipoproteins
Apolipoprotein A–I
Apolipoprotein B
Apolipoprotein B/A–I
−0.6 −0.4 −0.2 0 0.2 0.4 0.6 0.8
Age adjusted difference in mean metabolite levels (95% CI) comparing women who experienced GD in this pregnancy to those who did not
White European
South Asian

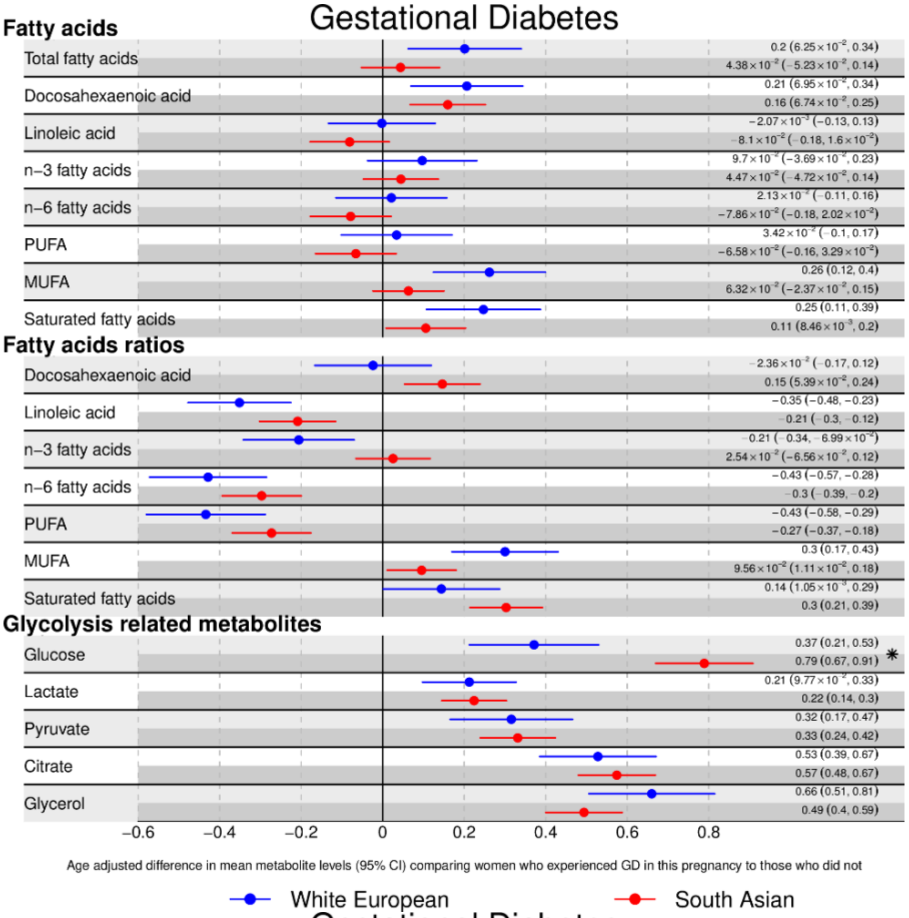
Gestational Diabetes
Fatty acids
Total fatty acids
Docosahexaenoic acid
Linoleic acid
n−3 fatty acids
n−6 fatty acids
PUFA
MUFA
Saturated fatty acids
Fatty acids ratios
Docosahexaenoic acid
Linoleic acid
n−3 fatty acids
n−6 fatty acids
PUFA
MUFA
Saturated fatty acids
Glycolysis related metabolites
Glucose
Lactate
Pyruvate
Citrate
Glycerol
−0.6 −0.4 −0.2 0 0.2 0.4 0.6 0.8
Age adjusted difference in mean metabolite levels (95% CI) comparing women who experienced GD in this pregnancy to those who did not
White European
South Asian

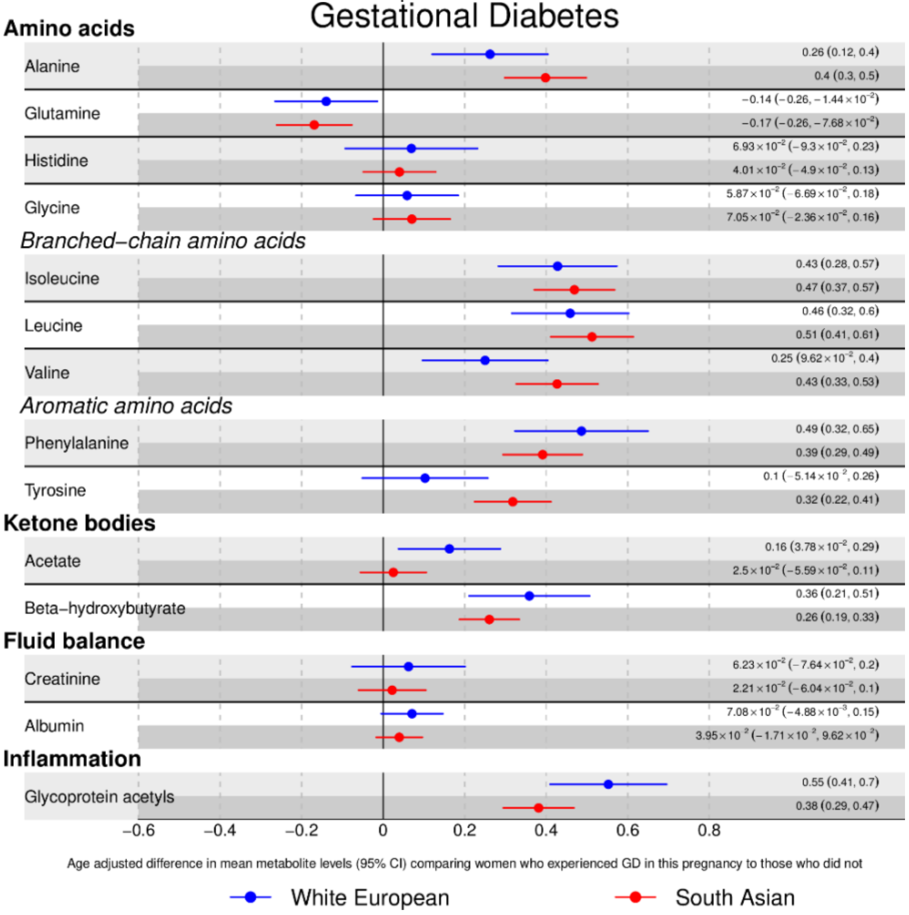
Gestational Diabetes
Amino acids
Alanine
Glutamine
Histidine
Glycine
Branched−chain amino acids
Isoleucine
Leucine
Valine
Aromatic amino acids
Phenylalanine
Tyrosine
Ketone bodies
Acetate
Beta−hydroxybutyrate
Fluid balance
Creatinine
Albumin
Inflammation
Glycoprotein acetyls
−0.6 −0.4 −0.2 0 0.2 0.4 0.6 0.8
Age adjusted difference in mean metabolite levels (95% CI) comparing women who experienced GD in this pregnancy to those who did not
White European
South Asian

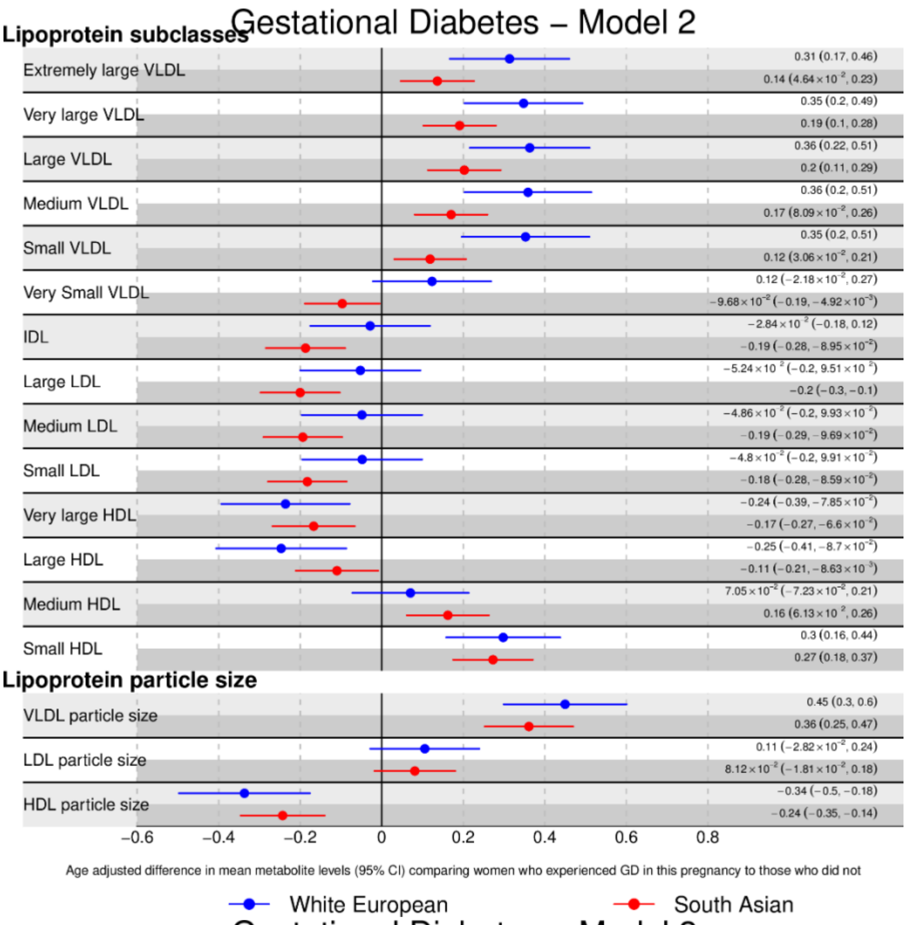

Gestational Diabetes – Model 2
Lipoprotein subclasses
Extremely large VLDL
Very large VLDL
Large VLDL
Medium VLDL
Small VLDL
Very Small VLDL
IDL
Large LDL
Medium LDL
Small LDL
Very large HDL
Large HDL
Medium HDL
Small HDL
Lipoprotein particle size
VLDL particle size
LDL particle size
HDL particle size
Age adjusted difference in mean metabolite levels (95% CI) comparing women who experienced GD in this pregnancy to those who did not
White European
South Asian

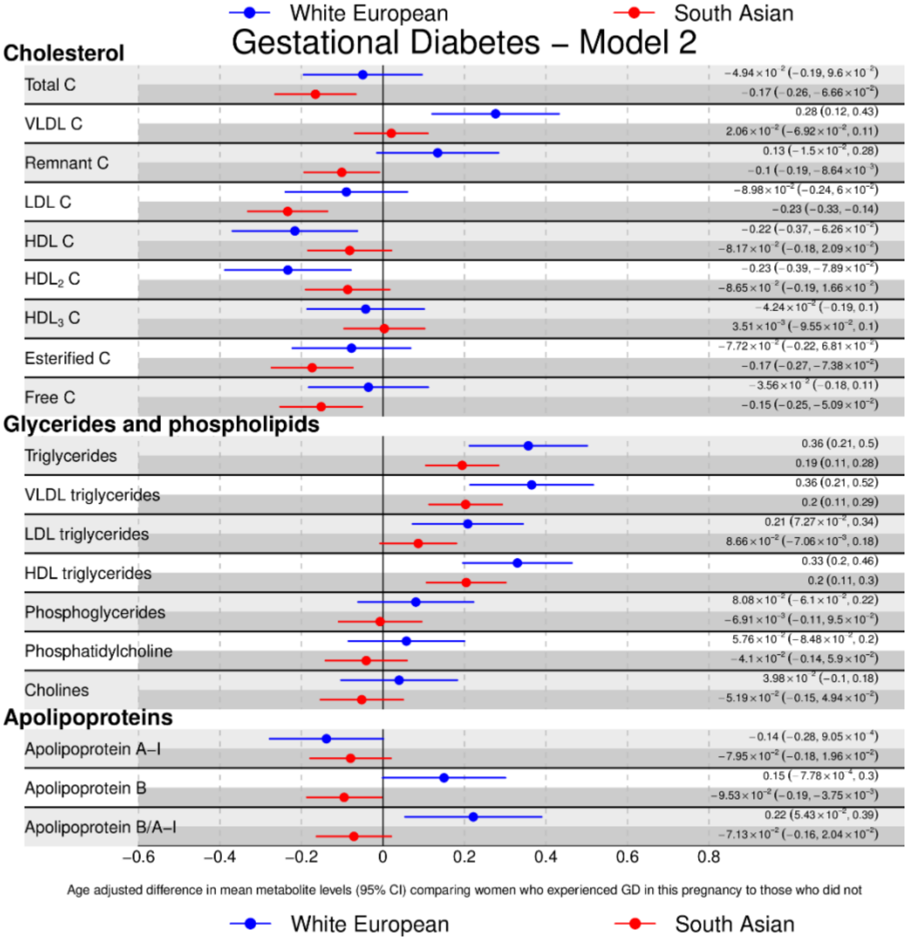

Gestational Diabetes – Model 2
Cholesterol
Total C
VLDL C
Remnant C
LDL C
HDL C
HDL2 C
HDL3 C
Esterified C
Free C
Glycerides and phospholipids
Triglycerides
VLDL triglycerides
LDL triglycerides
HDL triglycerides
Phosphoglycerides
Phosphatidylcholine
Cholines
Apolipoproteins
Apolipoprotein A–I
Apolipoprotein B
Apolipoprotein B/A–I
Age adjusted difference in mean metabolite levels (95% CI) comparing women who experienced GD in this pregnancy to those who did not
White European
South Asian

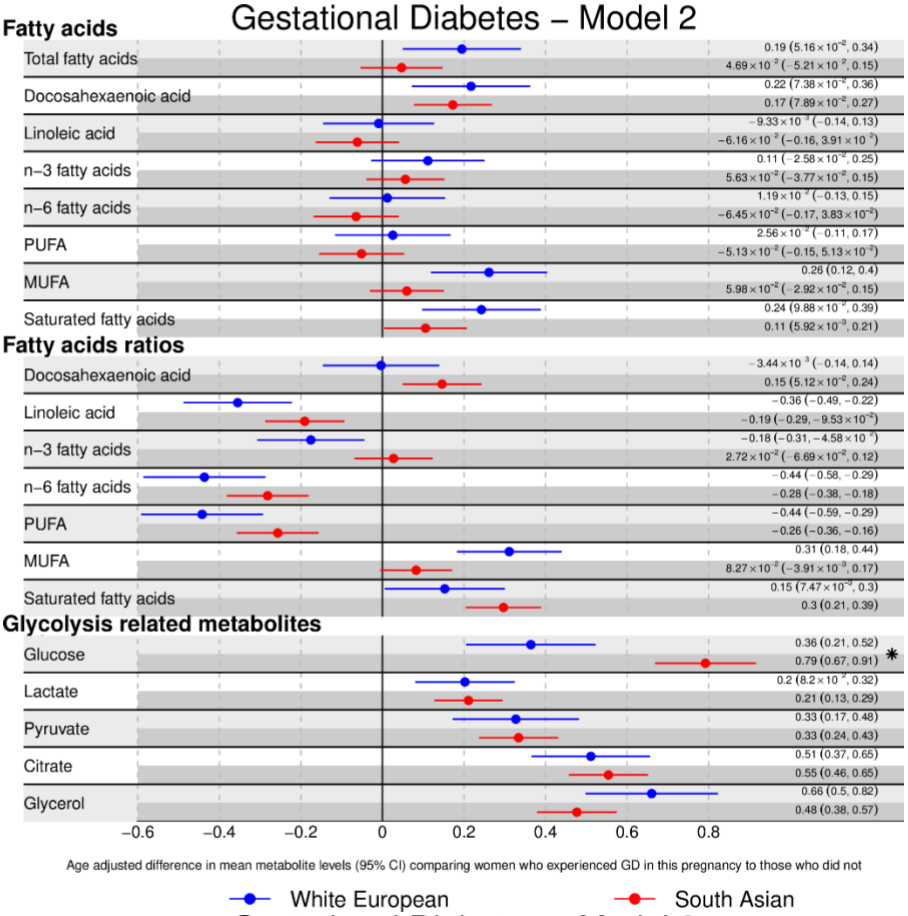
Gestational Diabetes – Model 2
Fatty acids
Total fatty acids
0.19 (5.16×10^{-2}, 0.34)
4.69×10^{-2} (-5.21×10^{-2}, 0.15)
Docosahexaenoic acid
0.22 (7.38×10^{-2}, 0.36)
0.17 (7.89×10^{-2}, 0.27)
Linoleic acid
-9.33×10^{-3} (−0.14, 0.13)
-6.16×10^{-2} (−0.16, 3.91×10^{-2})
n−3 fatty acids
0.11 (-2.58×10^{-2}, 0.25)
5.63×10^{-2} (-3.77×10^{-2}, 0.15)
n−6 fatty acids
1.19×10^{-2} (−0.13, 0.15)
-6.45×10^{-2} (−0.17, 3.83×10^{-2})
PUFA
2.56×10^{-2} (−0.11, 0.17)
-5.13×10^{-2} (−0.15, 5.13×10^{-2})
MUFA
0.26 (0.12, 0.4)
5.98×10^{-2} (-2.92×10^{-2}, 0.15)
Saturated fatty acids
0.24 (9.88×10^{-2}, 0.39)
0.11 (5.92×10^{-3}, 0.21)
Fatty acids ratios
Docosahexaenoic acid
-3.44×10^{-3} (−0.14, 0.14)
0.15 (5.12×10^{-2}, 0.24)
Linoleic acid
−0.36 (−0.49, −0.22)
−0.19 (−0.29, -9.53×10^{-2})
n−3 fatty acids
−0.18 (−0.31, -4.58×10^{-2})
2.72×10^{-2} (-6.69×10^{-2}, 0.12)
n−6 fatty acids
−0.44 (−0.58, −0.29)
−0.28 (−0.38, −0.18)
PUFA
−0.44 (−0.59, −0.29)
−0.26 (−0.36, −0.16)
MUFA
0.31 (0.18, 0.44)
8.27×10^{-2} (-3.91×10^{-3}, 0.17)
Saturated fatty acids
0.15 (7.47×10^{-3}, 0.3)
0.3 (0.21, 0.39)
Glycolysis related metabolites
Glucose
0.36 (0.21, 0.52)
0.79 (0.67, 0.91) *
Lactate
0.2 (8.2×10^{-2}, 0.32)
0.21 (0.13, 0.29)
Pyruvate
0.33 (0.17, 0.48)
0.33 (0.24, 0.43)
Citrate
0.51 (0.37, 0.65)
0.55 (0.46, 0.65)
Glycerol
0.66 (0.5, 0.82)
0.48 (0.38, 0.57)
−0.6 −0.4 −0.2 0 0.2 0.4 0.6 0.8
Age adjusted difference in mean metabolite levels (95% CI) comparing women who experienced GD in this pregnancy to those who did not
White European
South Asian

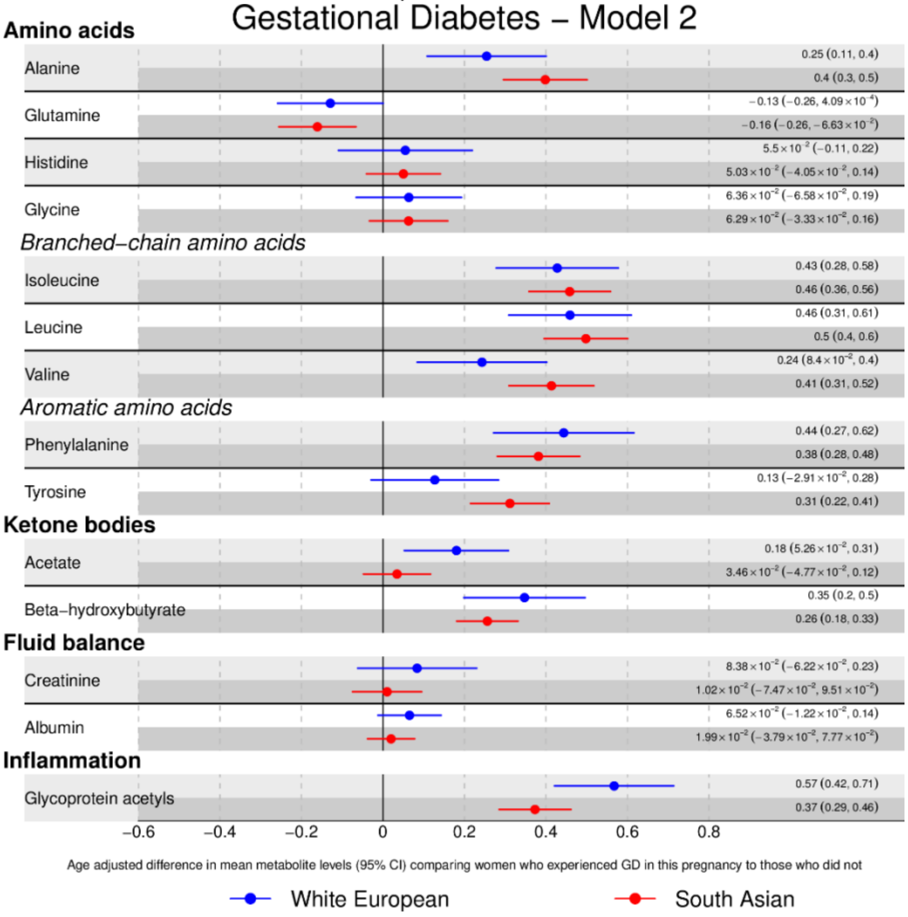
Gestational Diabetes – Model 2
Amino acids
Alanine
0.25 (0.11, 0.4)
0.4 (0.3, 0.5)
Glutamine
−0.13 (−0.26, 4.09×10^{-4})
−0.16 (−0.26, -6.63×10^{-2})
Histidine
5.5×10^{-2} (−0.11, 0.22)
5.03×10^{-2} (-4.05×10^{-2}, 0.14)
Glycine
6.36×10^{-2} (-6.58×10^{-2}, 0.19)
6.29×10^{-2} (-3.33×10^{-2}, 0.16)
Branched−chain amino acids
Isoleucine
0.43 (0.28, 0.58)
0.46 (0.36, 0.56)
Leucine
0.46 (0.31, 0.61)
0.5 (0.4, 0.6)
Valine
0.24 (8.4×10^{-2}, 0.4)
0.41 (0.31, 0.52)
Aromatic amino acids
Phenylalanine
0.44 (0.27, 0.62)
0.38 (0.28, 0.48)
Tyrosine
0.13 (-2.91×10^{-2}, 0.28)
0.31 (0.22, 0.41)
Ketone bodies
Acetate
0.18 (5.26×10^{-2}, 0.31)
3.46×10^{-2} (-4.77×10^{-2}, 0.12)
Beta−hydroxybutyrate
0.35 (0.2, 0.5)
0.26 (0.18, 0.33)
Fluid balance
Creatinine
8.38×10^{-2} (-6.22×10^{-2}, 0.23)
1.02×10^{-2} (-7.47×10^{-2}, 9.51×10^{-2})
Albumin
6.52×10^{-2} (-1.22×10^{-2}, 0.14)
1.99×10^{-2} (-3.79×10^{-2}, 7.77×10^{-2})
Inflammation
Glycoprotein acetyls
0.57 (0.42, 0.71)
0.37 (0.29, 0.46)
−0.6 −0.4 −0.2 0 0.2 0.4 0.6 0.8
Age adjusted difference in mean metabolite levels (95% CI) comparing women who experienced GD in this pregnancy to those who did not
White European
South Asian

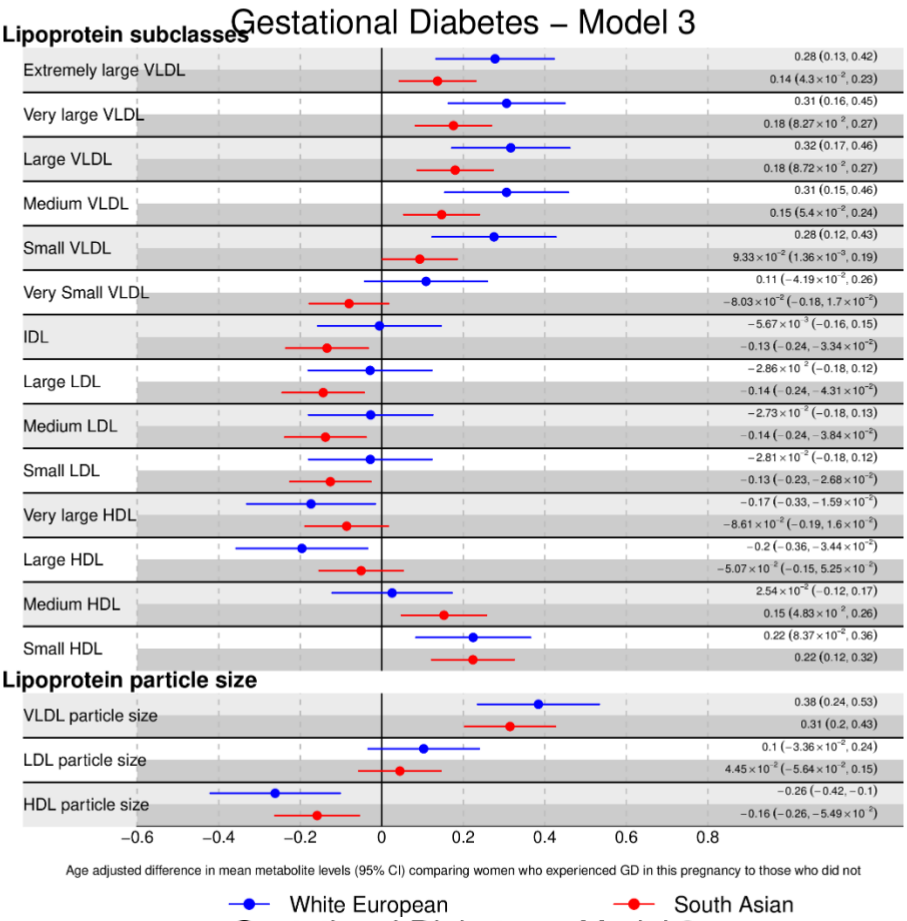
Gestational Diabetes – Model 3
Lipoprotein subclasses
Extremely large VLDL
0.28 (0.13, 0.42)
0.14 (4.3×10^-2, 0.23)
Very large VLDL
0.31 (0.16, 0.45)
0.18 (8.27×10^-2, 0.27)
Large VLDL
0.32 (0.17, 0.46)
0.18 (8.72×10^-2, 0.27)
Medium VLDL
0.31 (0.15, 0.46)
0.15 (5.4×10^-2, 0.24)
Small VLDL
0.28 (0.12, 0.43)
9.33×10^-2 (1.36×10^-3, 0.19)
Very Small VLDL
0.11 (−4.19×10^-2, 0.26)
−8.03×10^-2 (−0.18, 1.7×10^-2)
IDL
−5.67×10^-3 (−0.16, 0.15)
−0.13 (−0.24, −3.34×10^-2)
Large LDL
−2.86×10^-2 (−0.18, 0.12)
−0.14 (−0.24, −4.31×10^-2)
Medium LDL
−2.73×10^-2 (−0.18, 0.13)
−0.14 (−0.24, −3.84×10^-2)
Small LDL
−2.81×10^-2 (−0.18, 0.12)
−0.13 (−0.23, −2.68×10^-2)
Very large HDL
−0.17 (−0.33, −1.59×10^-2)
−8.61×10^-2 (−0.19, 1.6×10^-2)
Large HDL
−0.2 (−0.36, −3.44×10^-2)
−5.07×10^-2 (−0.15, 5.25×10^-2)
Medium HDL
2.54×10^-2 (−0.12, 0.17)
0.15 (4.83×10^-2, 0.26)
Small HDL
0.22 (8.37×10^-2, 0.36)
0.22 (0.12, 0.32)
Lipoprotein particle size
VLDL particle size
0.38 (0.24, 0.53)
0.31 (0.2, 0.43)
LDL particle size
0.1 (−3.36×10^-2, 0.24)
4.45×10^-2 (−5.64×10^-2, 0.15)
HDL particle size
−0.26 (−0.42, −0.1)
−0.16 (−0.26, −5.49×10^-2)
−0.6 −0.4 −0.2 0 0.2 0.4 0.6 0.8
Age adjusted difference in mean metabolite levels (95% CI) comparing women who experienced GD in this pregnancy to those who did not
White European
South Asian

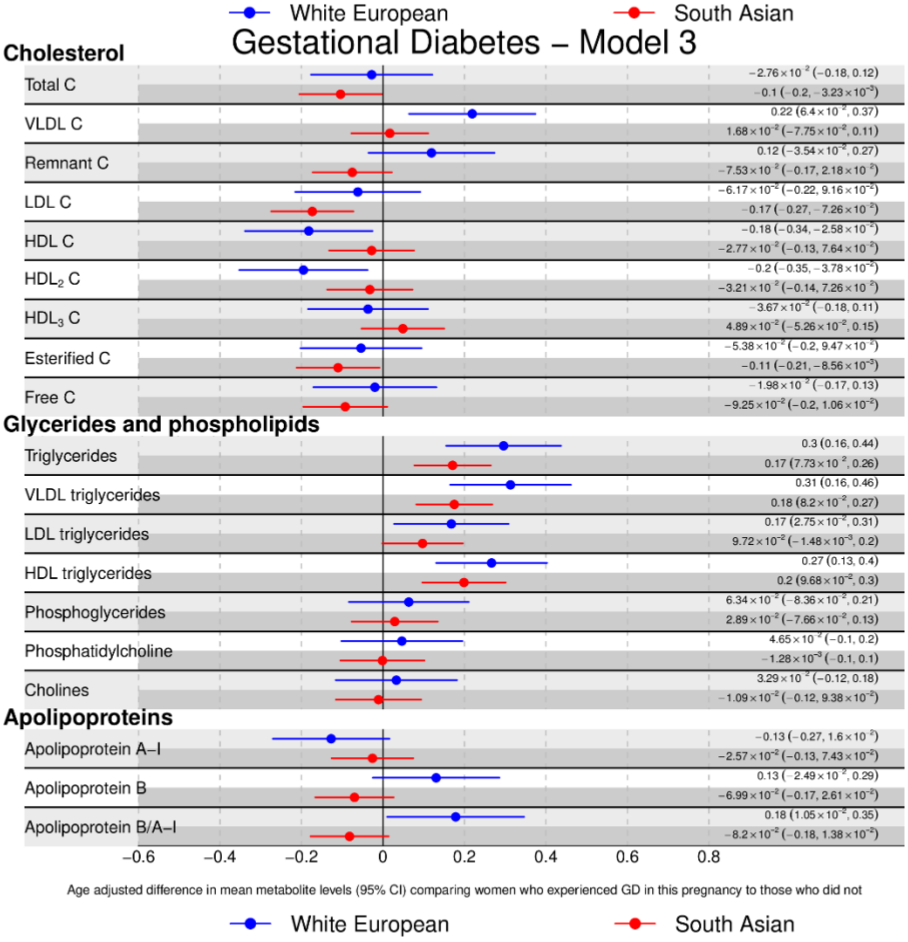
Gestational Diabetes – Model 3
Cholesterol
Total C
−2.76×10^-2 (−0.18, 0.12)
−0.1 (−0.2, −3.23×10^-3)
VLDL C
0.22 (6.4×10^-2, 0.37)
1.68×10^-2 (−7.75×10^-2, 0.11)
Remnant C
0.12 (−3.54×10^-2, 0.27)
−7.53×10^-2 (−0.17, 2.18×10^-2)
LDL C
−6.17×10^-2 (−0.22, 9.16×10^-2)
−0.17 (−0.27, −7.26×10^-2)
HDL C
−0.18 (−0.34, −2.58×10^-2)
−2.77×10^-2 (−0.13, 7.64×10^-2)
HDL2 C
−0.2 (−0.35, −3.78×10^-2)
−3.21×10^-2 (−0.14, 7.26×10^-2)
HDL3 C
−3.67×10^-2 (−0.18, 0.11)
4.89×10^-2 (−5.26×10^-2, 0.15)
Esterified C
−5.38×10^-2 (−0.2, 9.47×10^-2)
−0.11 (−0.21, −8.56×10^-3)
Free C
−1.98×10^-2 (−0.17, 0.13)
−9.25×10^-2 (−0.2, 1.06×10^-2)
Glycerides and phospholipids
Triglycerides
0.3 (0.16, 0.44)
0.17 (7.73×10^-2, 0.26)
VLDL triglycerides
0.31 (0.16, 0.46)
0.18 (8.2×10^-2, 0.27)
LDL triglycerides
0.17 (2.75×10^-2, 0.31)
9.72×10^-2 (−1.48×10^-3, 0.2)
HDL triglycerides
0.27 (0.13, 0.4)
0.2 (9.68×10^-2, 0.3)
Phosphoglycerides
6.34×10^-2 (−8.36×10^-2, 0.21)
2.89×10^-2 (−7.66×10^-2, 0.13)
Phosphatidylcholine
4.65×10^-2 (−0.1, 0.2)
−1.28×10^-3 (−0.1, 0.1)
Cholines
3.29×10^-2 (−0.12, 0.18)
−1.09×10^-2 (−0.12, 9.38×10^-2)
Apolipoproteins
Apolipoprotein A–I
−0.13 (−0.27, 1.6×10^-2)
−2.57×10^-2 (−0.13, 7.43×10^-2)
Apolipoprotein B
0.13 (−2.49×10^-2, 0.29)
−6.99×10^-2 (−0.17, 2.61×10^-2)
Apolipoprotein B/A–I
0.18 (1.05×10^-2, 0.35)
−8.2×10^-2 (−0.18, 1.38×10^-2)
−0.6 −0.4 −0.2 0 0.2 0.4 0.6 0.8
Age adjusted difference in mean metabolite levels (95% CI) comparing women who experienced GD in this pregnancy to those who did not
White European
South Asian

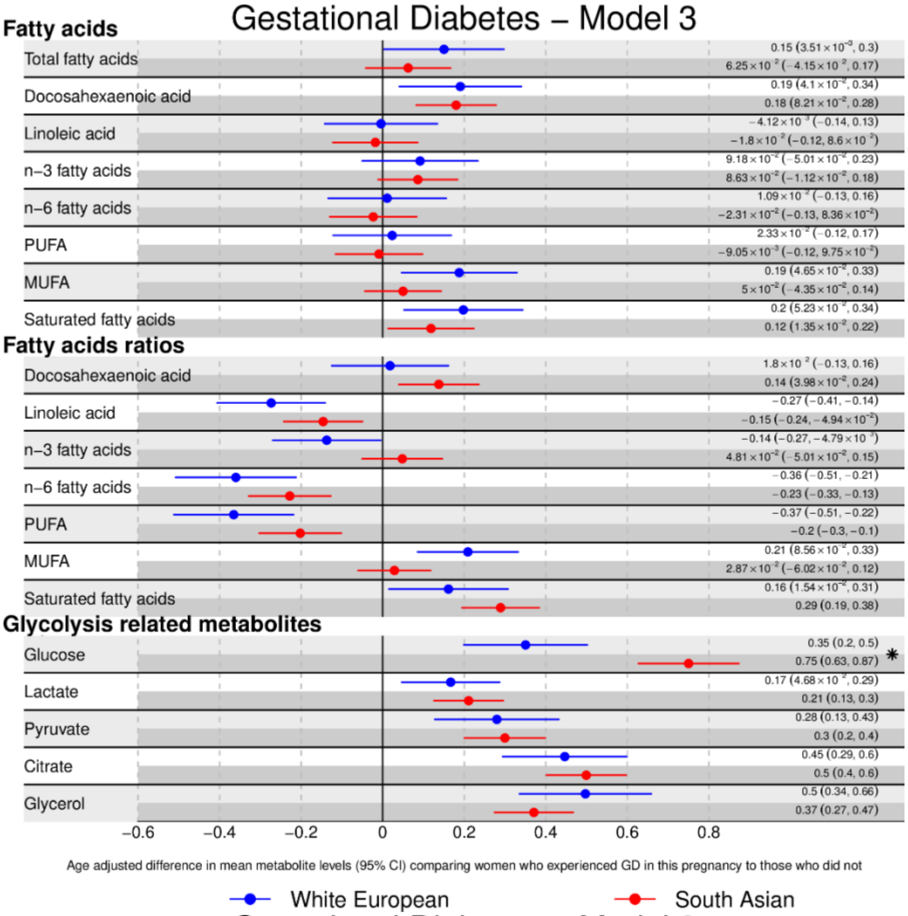
Gestational Diabetes – Model 3
Fatty acids
Total fatty acids
Docosahexaenoic acid
Linoleic acid
n−3 fatty acids
n−6 fatty acids
PUFA
MUFA
Saturated fatty acids
Fatty acids ratios
Docosahexaenoic acid
Linoleic acid
n−3 fatty acids
n−6 fatty acids
PUFA
MUFA
Saturated fatty acids
Glycolysis related metabolites
Glucose
Lactate
Pyruvate
Citrate
Glycerol
0.15 (3.51 × 10^-3, 0.3)
6.25 × 10^-2 (−4.15 × 10^-2, 0.17)
0.19 (4.1 × 10^-2, 0.34)
0.18 (8.21 × 10^-2, 0.28)
−4.12 × 10^-3 (−0.14, 0.13)
−1.8 × 10^-2 (−0.12, 8.6 × 10^-2)
9.18 × 10^-2 (−5.01 × 10^-2, 0.23)
8.63 × 10^-2 (−1.12 × 10^-2, 0.18)
1.09 × 10^-2 (−0.13, 0.16)
−2.31 × 10^-2 (−0.13, 8.36 × 10^-2)
2.33 × 10^-2 (−0.12, 0.17)
−9.05 × 10^-3 (−0.12, 9.75 × 10^-2)
0.19 (4.65 × 10^-2, 0.33)
5 × 10^-2 (−4.35 × 10^-2, 0.14)
0.2 (5.23 × 10^-2, 0.34)
0.12 (1.35 × 10^-2, 0.22)
1.8 × 10^-2 (−0.13, 0.16)
0.14 (3.98 × 10^-2, 0.24)
−0.27 (−0.41, −0.14)
−0.15 (−0.24, −4.94 × 10^-2)
−0.14 (−0.27, −4.79 × 10^-3)
4.81 × 10^-2 (−5.01 × 10^-2, 0.15)
−0.36 (−0.51, −0.21)
−0.23 (−0.33, −0.13)
−0.37 (−0.51, −0.22)
−0.2 (−0.3, −0.1)
0.21 (8.56 × 10^-2, 0.33)
2.87 × 10^-2 (−6.02 × 10^-2, 0.12)
0.16 (1.54 × 10^-2, 0.31)
0.29 (0.19, 0.38)
0.35 (0.2, 0.5)
0.75 (0.63, 0.87)
*
0.17 (4.68 × 10^-2, 0.29)
0.21 (0.13, 0.3)
0.28 (0.13, 0.43)
0.3 (0.2, 0.4)
0.45 (0.29, 0.6)
0.5 (0.4, 0.6)
0.5 (0.34, 0.66)
0.37 (0.27, 0.47)
−0.6 −0.4 −0.2 0 0.2 0.4 0.6 0.8
Age adjusted difference in mean metabolite levels (95% CI) comparing women who experienced GD in this pregnancy to those who did not
White European
South Asian

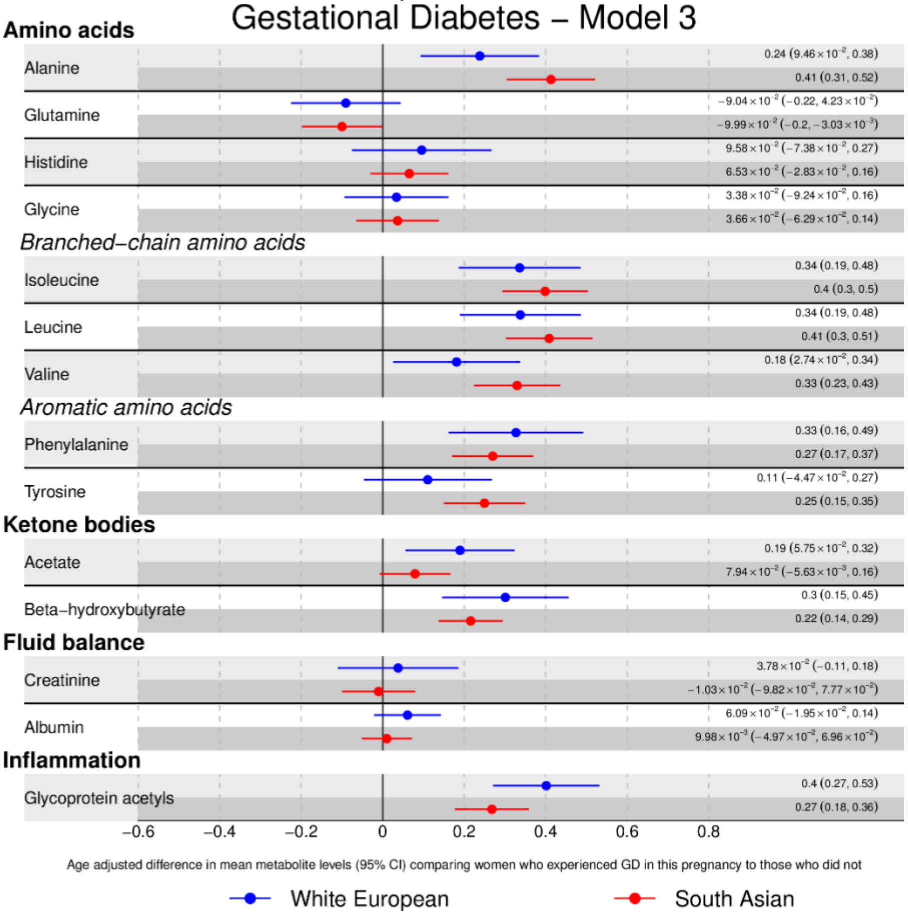
Gestational Diabetes – Model 3
Amino acids
Alanine
Glutamine
Histidine
Glycine
Branched−chain amino acids
Isoleucine
Leucine
Valine
Aromatic amino acids
Phenylalanine
Tyrosine
Ketone bodies
Acetate
Beta−hydroxybutyrate
Fluid balance
Creatinine
Albumin
Inflammation
Glycoprotein acetyls
0.24 (9.46 × 10^-2, 0.38)
0.41 (0.31, 0.52)
−9.04 × 10^-2 (−0.22, 4.23 × 10^-2)
−9.99 × 10^-2 (−0.2, −3.03 × 10^-3)
9.58 × 10^-2 (−7.38 × 10^-2, 0.27)
6.53 × 10^-2 (−2.83 × 10^-2, 0.16)
3.38 × 10^-2 (−9.24 × 10^-2, 0.16)
3.66 × 10^-2 (−6.29 × 10^-2, 0.14)
0.34 (0.19, 0.48)
0.4 (0.3, 0.5)
0.34 (0.19, 0.48)
0.41 (0.3, 0.51)
0.18 (2.74 × 10^-2, 0.34)
0.33 (0.23, 0.43)
0.33 (0.16, 0.49)
0.27 (0.17, 0.37)
0.11 (−4.47 × 10^-2, 0.27)
0.25 (0.15, 0.35)
0.19 (5.75 × 10^-2, 0.32)
7.94 × 10^-2 (−5.63 × 10^-3, 0.16)
0.3 (0.15, 0.45)
0.22 (0.14, 0.29)
3.78 × 10^-2 (−0.11, 0.18)
−1.03 × 10^-2 (−9.82 × 10^-2, 7.77 × 10^-2)
6.09 × 10^-2 (−1.95 × 10^-2, 0.14)
9.98 × 10^-3 (−4.97 × 10^-2, 6.96 × 10^-2)
0.4 (0.27, 0.53)
0.27 (0.18, 0.36)
−0.6 −0.4 −0.2 0 0.2 0.4 0.6 0.8
Age adjusted difference in mean metabolite levels (95% CI) comparing women who experienced GD in this pregnancy to those who did not
White European
South Asian

Figure S10. Associations of maternal gestational diabetes with pregnancy metabolic profiles stratified by ethnicity (model 1 = age-adjusted; model 2 = age-, parity- and education-adjusted, model 3 = age-, parity-, education- and BMI-adjusted). Data points show SD differences for White European (blue) and South Asian (red) women. Error bars = 95% confidence intervals. Exact point estimates and their corresponding 95% CIs are displayed in text to the right of each point. * denotes strong statistical evidence from the interaction test (Pinteraction <0.001). Differences in quantified units (mostly mmol/l) are listed in Supplementary File 2. Abbreviations: VLDL, very low-density lipoprotein; LDL, low-density lipoprotein; HDL, high-density lipoprotein; C, cholesterol; MUFA, monounsaturated fatty acids; PUFA, polyunsaturated fatty acids

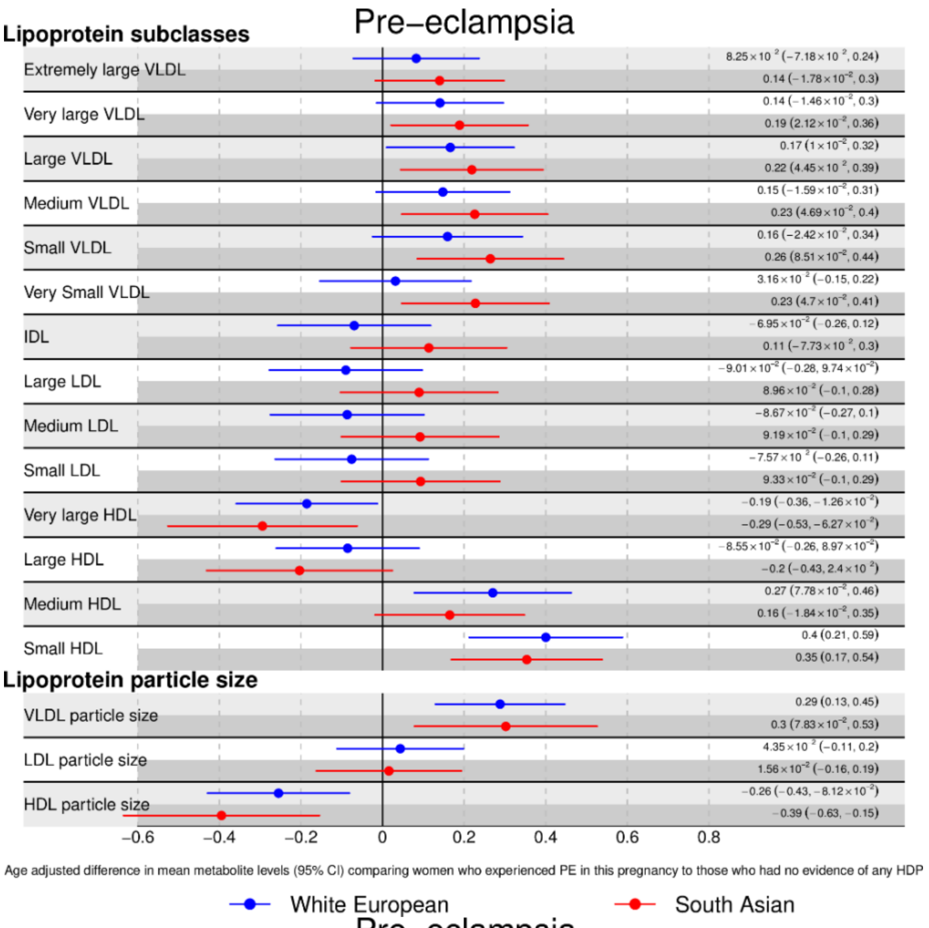
Pre−eclampsia
Lipoprotein subclasses
Extremely large VLDL
8.25×10^{-2} (-7.18×10^{-2}, 0.24)
0.14 (-1.78×10^{-2}, 0.3)
Very large VLDL
0.14 (-1.46×10^{-2}, 0.3)
0.19 (2.12×10^{-2}, 0.36)
Large VLDL
0.17 (1×10^{-2}, 0.32)
0.22 (4.45×10^{-2}, 0.39)
Medium VLDL
0.15 (-1.59×10^{-2}, 0.31)
0.23 (4.69×10^{-2}, 0.4)
Small VLDL
0.16 (-2.42×10^{-2}, 0.34)
0.26 (8.51×10^{-2}, 0.44)
Very Small VLDL
3.16×10^{-2} (−0.15, 0.22)
0.23 (4.7×10^{-2}, 0.41)
IDL
-6.95×10^{-2} (−0.26, 0.12)
0.11 (-7.73×10^{-2}, 0.3)
Large LDL
-9.01×10^{-2} (−0.28, 9.74×10^{-2})
8.96×10^{-2} (−0.1, 0.28)
Medium LDL
-8.67×10^{-2} (−0.27, 0.1)
9.19×10^{-2} (−0.1, 0.29)
Small LDL
-7.57×10^{-2} (−0.26, 0.11)
9.33×10^{-2} (−0.1, 0.29)
Very large HDL
−0.19 (−0.36, -1.26×10^{-2})
−0.29 (−0.53, -6.27×10^{-2})
Large HDL
-8.55×10^{-2} (−0.26, 8.97×10^{-2})
−0.2 (−0.43, 2.4×10^{-2})
Medium HDL
0.27 (7.78×10^{-2}, 0.46)
0.16 (-1.84×10^{-2}, 0.35)
Small HDL
0.4 (0.21, 0.59)
0.35 (0.17, 0.54)
Lipoprotein particle size
VLDL particle size
0.29 (0.13, 0.45)
0.3 (7.83×10^{-2}, 0.53)
LDL particle size
4.35×10^{-2} (−0.11, 0.2)
1.56×10^{-2} (−0.16, 0.19)
HDL particle size
−0.26 (−0.43, -8.12×10^{-2})
−0.39 (−0.63, −0.15)
−0.6
−0.4
−0.2
0
0.2
0.4
0.6
0.8
Age adjusted difference in mean metabolite levels (95% CI) comparing women who experienced PE in this pregnancy to those who had no evidence of any HDP
White European
South Asian

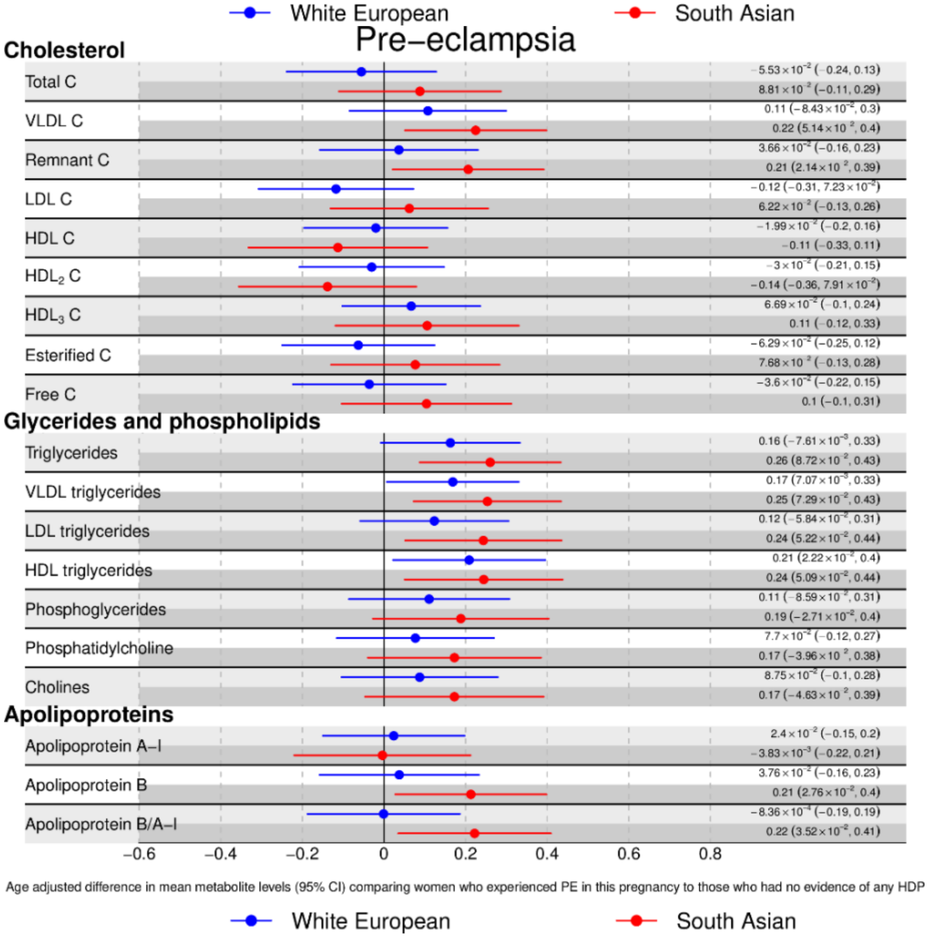
Pre−eclampsia
Cholesterol
Total C
-5.53×10^{-2} (−0.24, 0.13)
8.81×10^{-2} (−0.11, 0.29)
VLDL C
0.11 (-8.43×10^{-2}, 0.3)
0.22 (5.14×10^{-2}, 0.4)
Remnant C
3.66×10^{-2} (−0.16, 0.23)
0.21 (2.14×10^{-2}, 0.39)
LDL C
−0.12 (−0.31, 7.23×10^{-2})
6.22×10^{-2} (−0.13, 0.26)
HDL C
-1.99×10^{-2} (−0.2, 0.16)
−0.11 (−0.33, 0.11)
HDL_2 C
-3×10^{-2} (−0.21, 0.15)
−0.14 (−0.36, 7.91×10^{-2})
HDL_3 C
6.69×10^{-2} (−0.1, 0.24)
0.11 (−0.12, 0.33)
Esterified C
-6.29×10^{-2} (−0.25, 0.12)
7.68×10^{-2} (−0.13, 0.28)
Free C
-3.6×10^{-2} (−0.22, 0.15)
0.1 (−0.1, 0.31)
Glycerides and phospholipids
Triglycerides
0.16 (-7.61×10^{-3}, 0.33)
0.26 (8.72×10^{-2}, 0.43)
VLDL triglycerides
0.17 (7.07×10^{-3}, 0.33)
0.25 (7.29×10^{-2}, 0.43)
LDL triglycerides
0.12 (-5.84×10^{-2}, 0.31)
0.24 (5.22×10^{-2}, 0.44)
HDL triglycerides
0.21 (2.22×10^{-2}, 0.4)
0.24 (5.09×10^{-2}, 0.44)
Phosphoglycerides
0.11 (-8.59×10^{-2}, 0.31)
0.19 (-2.71×10^{-2}, 0.4)
Phosphatidylcholine
7.7×10^{-2} (−0.12, 0.27)
0.17 (-3.96×10^{-2}, 0.38)
Cholines
8.75×10^{-2} (−0.1, 0.28)
0.17 (-4.63×10^{-2}, 0.39)
Apolipoproteins
Apolipoprotein A−I
2.4×10^{-2} (−0.15, 0.2)
-3.83×10^{-3} (−0.22, 0.21)
Apolipoprotein B
3.76×10^{-2} (−0.16, 0.23)
0.21 (2.76×10^{-2}, 0.4)
Apolipoprotein B/A−I
-8.36×10^{-4} (−0.19, 0.19)
0.22 (3.52×10^{-2}, 0.41)
−0.6
−0.4
−0.2
0
0.2
0.4
0.6
0.8
Age adjusted difference in mean metabolite levels (95% CI) comparing women who experienced PE in this pregnancy to those who had no evidence of any HDP
White European
South Asian

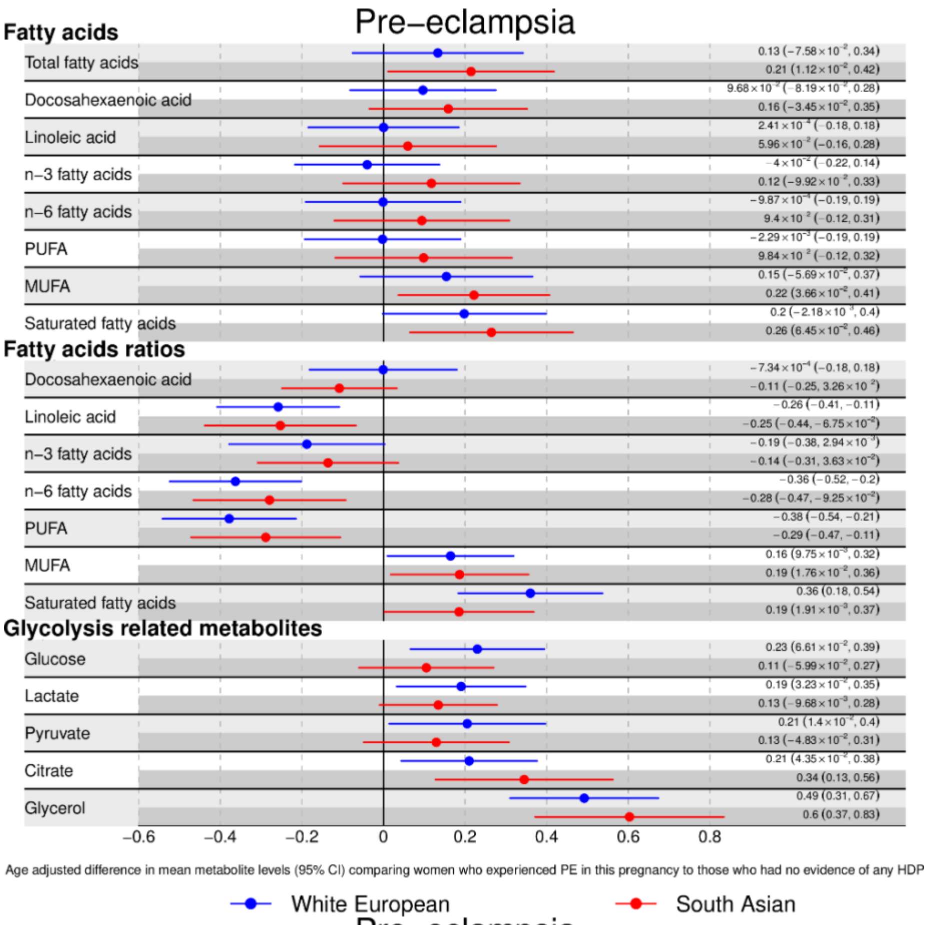

Pre−eclampsia
Fatty acids
Total fatty acids
Docosahexaenoic acid
Linoleic acid
n−3 fatty acids
n−6 fatty acids
PUFA
MUFA
Saturated fatty acids
Fatty acids ratios
Docosahexaenoic acid
Linoleic acid
n−3 fatty acids
n−6 fatty acids
PUFA
MUFA
Saturated fatty acids
Glycolysis related metabolites
Glucose
Lactate
Pyruvate
Citrate
Glycerol
−0.6 −0.4 −0.2 0 0.2 0.4 0.6 0.8
Age adjusted difference in mean metabolite levels (95% CI) comparing women who experienced PE in this pregnancy to those who had no evidence of any HDP
White European
South Asian

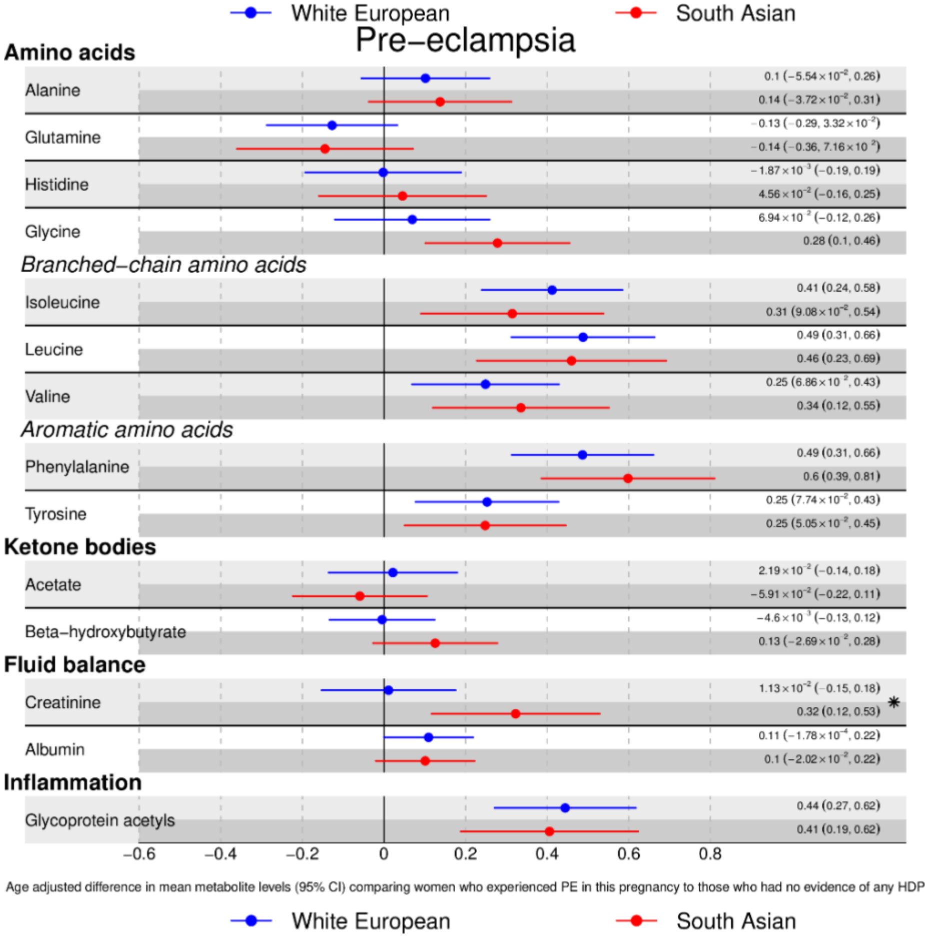

Pre−eclampsia
Amino acids
Alanine
Glutamine
Histidine
Glycine
Branched−chain amino acids
Isoleucine
Leucine
Valine
Aromatic amino acids
Phenylalanine
Tyrosine
Ketone bodies
Acetate
Beta−hydroxybutyrate
Fluid balance
Creatinine
Albumin
Inflammation
Glycoprotein acetyls
−0.6 −0.4 −0.2 0 0.2 0.4 0.6 0.8
Age adjusted difference in mean metabolite levels (95% CI) comparing women who experienced PE in this pregnancy to those who had no evidence of any HDP
White European
South Asian

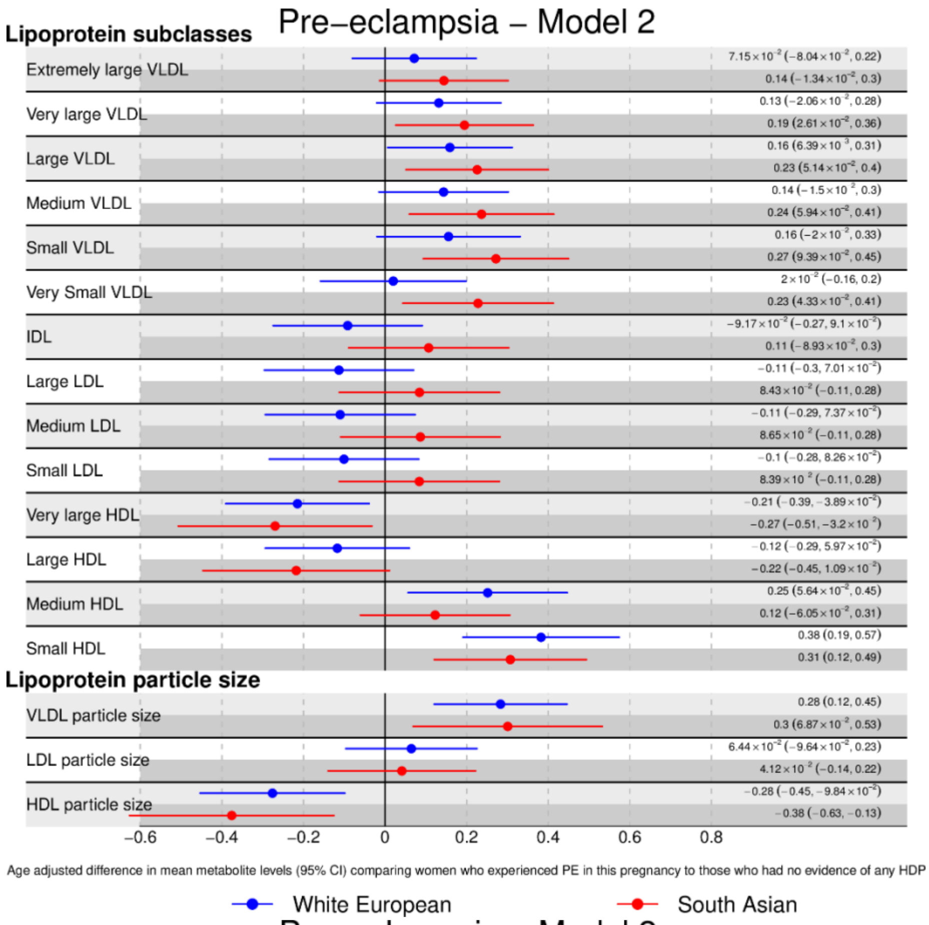
Pre–eclampsia – Model 2
Lipoprotein subclasses
Extremely large VLDL
Very large VLDL
Large VLDL
Medium VLDL
Small VLDL
Very Small VLDL
IDL
Large LDL
Medium LDL
Small LDL
Very large HDL
Large HDL
Medium HDL
Small HDL
Lipoprotein particle size
VLDL particle size
LDL particle size
HDL particle size
7.15×10⁻² (−8.04×10⁻², 0.22)
0.14 (−1.34×10⁻², 0.3)
0.13 (−2.06×10⁻², 0.28)
0.19 (2.61×10⁻², 0.36)
0.16 (6.39×10⁻³, 0.31)
0.23 (5.14×10⁻², 0.4)
0.14 (−1.5×10⁻², 0.3)
0.24 (5.94×10⁻², 0.41)
0.16 (−2×10⁻², 0.33)
0.27 (9.39×10⁻², 0.45)
2×10⁻² (−0.16, 0.2)
0.23 (4.33×10⁻², 0.41)
−9.17×10⁻² (−0.27, 9.1×10⁻²)
0.11 (−8.93×10⁻², 0.3)
−0.11 (−0.3, 7.01×10⁻²)
8.43×10⁻² (−0.11, 0.28)
−0.11 (−0.29, 7.37×10⁻²)
8.65×10⁻² (−0.11, 0.28)
−0.1 (−0.28, 8.26×10⁻²)
8.39×10⁻² (−0.11, 0.28)
−0.21 (−0.39, −3.89×10⁻²)
−0.27 (−0.51, −3.2×10⁻²)
−0.12 (−0.29, 5.97×10⁻²)
−0.22 (−0.45, 1.09×10⁻²)
0.25 (5.64×10⁻², 0.45)
0.12 (−6.05×10⁻², 0.31)
0.38 (0.19, 0.57)
0.31 (0.12, 0.49)
0.28 (0.12, 0.45)
0.3 (6.87×10⁻², 0.53)
6.44×10⁻² (−9.64×10⁻², 0.23)
4.12×10⁻² (−0.14, 0.22)
−0.28 (−0.45, −9.84×10⁻²)
−0.38 (−0.63, −0.13)
−0.6
−0.4
−0.2
0
0.2
0.4
0.6
0.8
Age adjusted difference in mean metabolite levels (95% CI) comparing women who experienced PE in this pregnancy to those who had no evidence of any HDP
White European
South Asian

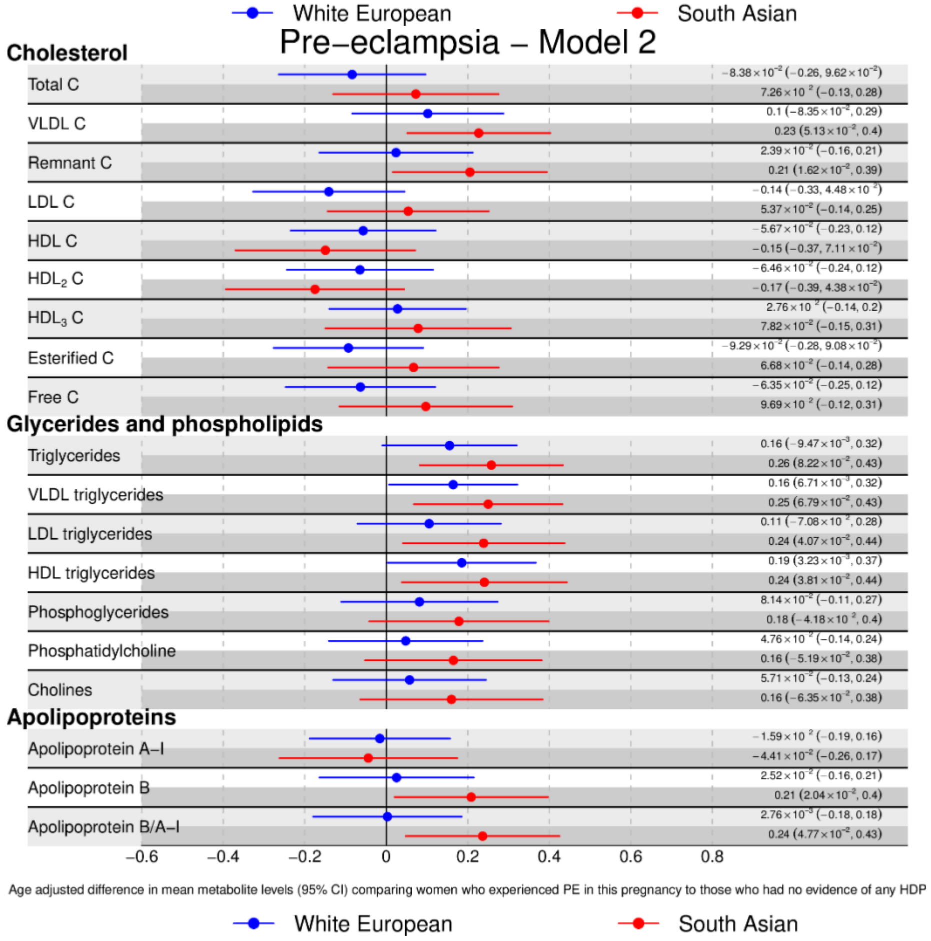
Pre–eclampsia – Model 2
Cholesterol
Total C
VLDL C
Remnant C
LDL C
HDL C
HDL2 C
HDL3 C
Esterified C
Free C
Glycerides and phospholipids
Triglycerides
VLDL triglycerides
LDL triglycerides
HDL triglycerides
Phosphoglycerides
Phosphatidylcholine
Cholines
Apolipoproteins
Apolipoprotein A–I
Apolipoprotein B
Apolipoprotein B/A–I
−8.38×10⁻² (−0.26, 9.62×10⁻²)
7.26×10⁻² (−0.13, 0.28)
0.1 (−8.35×10⁻², 0.29)
0.23 (5.13×10⁻², 0.4)
2.39×10⁻² (−0.16, 0.21)
0.21 (1.62×10⁻², 0.39)
−0.14 (−0.33, 4.48×10⁻²)
5.37×10⁻² (−0.14, 0.25)
−5.67×10⁻² (−0.23, 0.12)
−0.15 (−0.37, 7.11×10⁻²)
−6.46×10⁻² (−0.24, 0.12)
−0.17 (−0.39, 4.38×10⁻²)
2.76×10⁻² (−0.14, 0.2)
7.82×10⁻² (−0.15, 0.31)
−9.29×10⁻² (−0.28, 9.08×10⁻²)
6.68×10⁻² (−0.14, 0.28)
−6.35×10⁻² (−0.25, 0.12)
9.69×10⁻² (−0.12, 0.31)
0.16 (−9.47×10⁻³, 0.32)
0.26 (8.22×10⁻², 0.43)
0.16 (6.71×10⁻³, 0.32)
0.25 (6.79×10⁻², 0.43)
0.11 (−7.08×10⁻², 0.28)
0.24 (4.07×10⁻², 0.44)
0.19 (3.23×10⁻³, 0.37)
0.24 (3.81×10⁻², 0.44)
8.14×10⁻² (−0.11, 0.27)
0.18 (−4.18×10⁻³, 0.4)
4.76×10⁻² (−0.14, 0.24)
0.16 (−5.19×10⁻², 0.38)
5.71×10⁻² (−0.13, 0.24)
0.16 (−6.35×10⁻², 0.38)
−1.59×10⁻² (−0.19, 0.16)
−4.41×10⁻² (−0.26, 0.17)
2.52×10⁻² (−0.16, 0.21)
0.21 (2.04×10⁻², 0.4)
2.76×10⁻³ (−0.18, 0.18)
0.24 (4.77×10⁻², 0.43)
−0.6
−0.4
−0.2
0
0.2
0.4
0.6
0.8
Age adjusted difference in mean metabolite levels (95% CI) comparing women who experienced PE in this pregnancy to those who had no evidence of any HDP
White European
South Asian

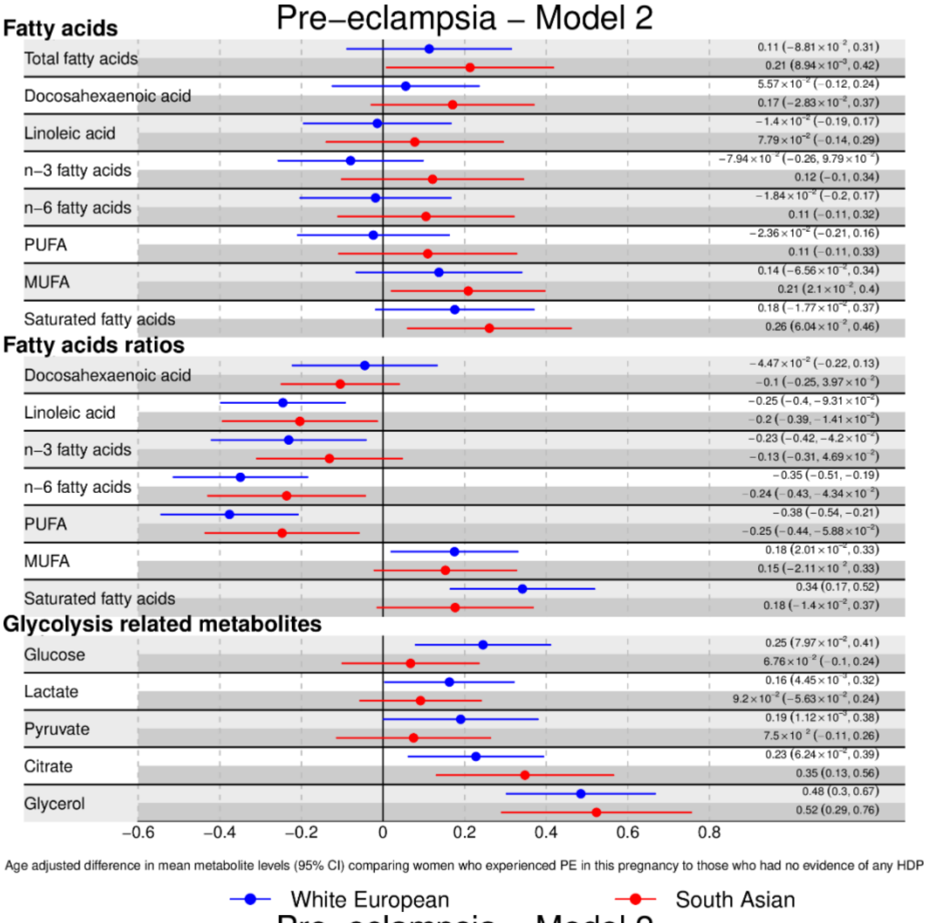

Pre−eclampsia − Model 2
Fatty acids
Total fatty acids
0.11 (−8.81×10⁻², 0.31)
0.21 (8.94×10⁻³, 0.42)
Docosahexaenoic acid
5.57×10⁻² (−0.12, 0.24)
0.17 (−2.83×10⁻², 0.37)
Linoleic acid
−1.4×10⁻² (−0.19, 0.17)
7.79×10⁻² (−0.14, 0.29)
n−3 fatty acids
−7.94×10⁻² (−0.26, 9.79×10⁻²)
0.12 (−0.1, 0.34)
n−6 fatty acids
−1.84×10⁻² (−0.2, 0.17)
0.11 (−0.11, 0.32)
PUFA
−2.36×10⁻² (−0.21, 0.16)
0.11 (−0.11, 0.33)
MUFA
0.14 (−6.56×10⁻², 0.34)
0.21 (2.1×10⁻², 0.4)
Saturated fatty acids
0.18 (−1.77×10⁻², 0.37)
0.26 (6.04×10⁻², 0.46)
Fatty acids ratios
Docosahexaenoic acid
−4.47×10⁻² (−0.22, 0.13)
−0.1 (−0.25, 3.97×10⁻²)
Linoleic acid
−0.25 (−0.4, −9.31×10⁻²)
−0.2 (−0.39, −1.41×10⁻²)
n−3 fatty acids
−0.23 (−0.42, −4.2×10⁻²)
−0.13 (−0.31, 4.69×10⁻²)
n−6 fatty acids
−0.35 (−0.51, −0.19)
−0.24 (−0.43, −4.34×10⁻²)
PUFA
−0.38 (−0.54, −0.21)
−0.25 (−0.44, −5.88×10⁻²)
MUFA
0.18 (2.01×10⁻², 0.33)
0.15 (−2.11×10⁻², 0.33)
Saturated fatty acids
0.34 (0.17, 0.52)
0.18 (−1.4×10⁻², 0.37)
Glycolysis related metabolites
Glucose
0.25 (7.97×10⁻², 0.41)
6.76×10⁻² (−0.1, 0.24)
Lactate
0.16 (4.45×10⁻³, 0.32)
9.2×10⁻² (−5.63×10⁻², 0.24)
Pyruvate
0.19 (1.12×10⁻³, 0.38)
7.5×10⁻² (−0.11, 0.26)
Citrate
0.23 (6.24×10⁻², 0.39)
0.35 (0.13, 0.56)
Glycerol
0.48 (0.3, 0.67)
0.52 (0.29, 0.76)
−0.6
−0.4
−0.2
0
0.2
0.4
0.6
0.8
Age adjusted difference in mean metabolite levels (95% CI) comparing women who experienced PE in this pregnancy to those who had no evidence of any HDP
White European
South Asian

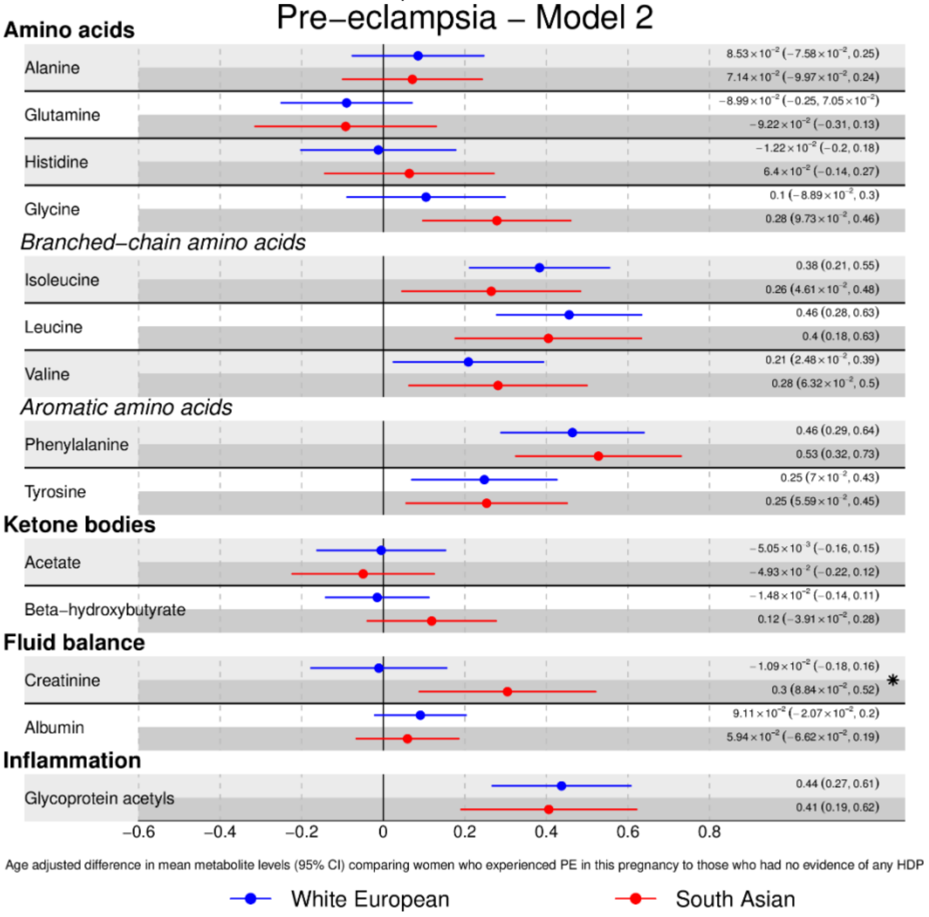

Pre−eclampsia − Model 2
Amino acids
Alanine
8.53×10⁻² (−7.58×10⁻², 0.25)
7.14×10⁻² (−9.97×10⁻², 0.24)
Glutamine
−8.99×10⁻² (−0.25, 7.05×10⁻²)
−9.22×10⁻² (−0.31, 0.13)
Histidine
−1.22×10⁻² (−0.2, 0.18)
6.4×10⁻² (−0.14, 0.27)
Glycine
0.1 (−8.89×10⁻², 0.3)
0.28 (9.73×10⁻², 0.46)
Branched−chain amino acids
Isoleucine
0.38 (0.21, 0.55)
0.26 (4.61×10⁻², 0.48)
Leucine
0.46 (0.28, 0.63)
0.4 (0.18, 0.63)
Valine
0.21 (2.48×10⁻², 0.39)
0.28 (6.32×10⁻², 0.5)
Aromatic amino acids
Phenylalanine
0.46 (0.29, 0.64)
0.53 (0.32, 0.73)
Tyrosine
0.25 (7×10⁻², 0.43)
0.25 (5.59×10⁻², 0.45)
Ketone bodies
Acetate
−5.05×10⁻³ (−0.16, 0.15)
−4.93×10⁻² (−0.22, 0.12)
Beta−hydroxybutyrate
−1.48×10⁻² (−0.14, 0.11)
0.12 (−3.91×10⁻², 0.28)
Fluid balance
Creatinine
−1.09×10⁻² (−0.18, 0.16)
0.3 (8.84×10⁻², 0.52)
*
Albumin
9.11×10⁻² (−2.07×10⁻², 0.2)
5.94×10⁻² (−6.62×10⁻², 0.19)
Inflammation
Glycoprotein acetyls
0.44 (0.27, 0.61)
0.41 (0.19, 0.62)
−0.6
−0.4
−0.2
0
0.2
0.4
0.6
0.8
Age adjusted difference in mean metabolite levels (95% CI) comparing women who experienced PE in this pregnancy to those who had no evidence of any HDP
White European
South Asian

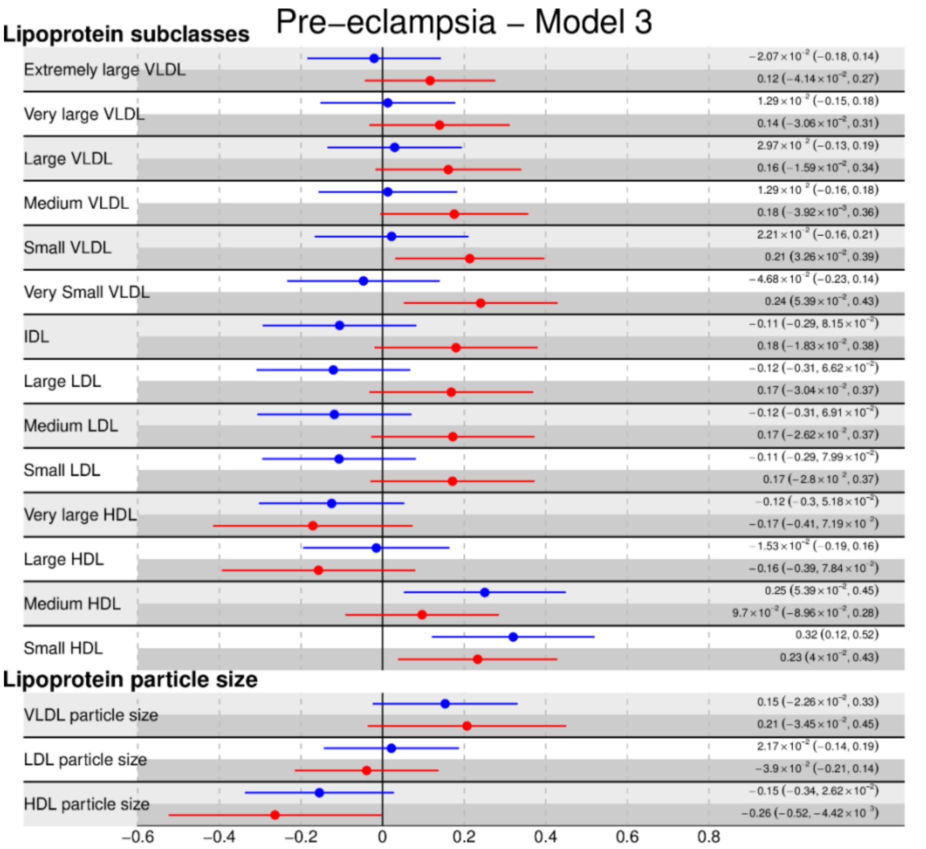

Age adjusted difference in mean metabolite levels (95% CI) comparing women who experienced PE in this pregnancy to those who had no evidence of any HDP

White European South Asian

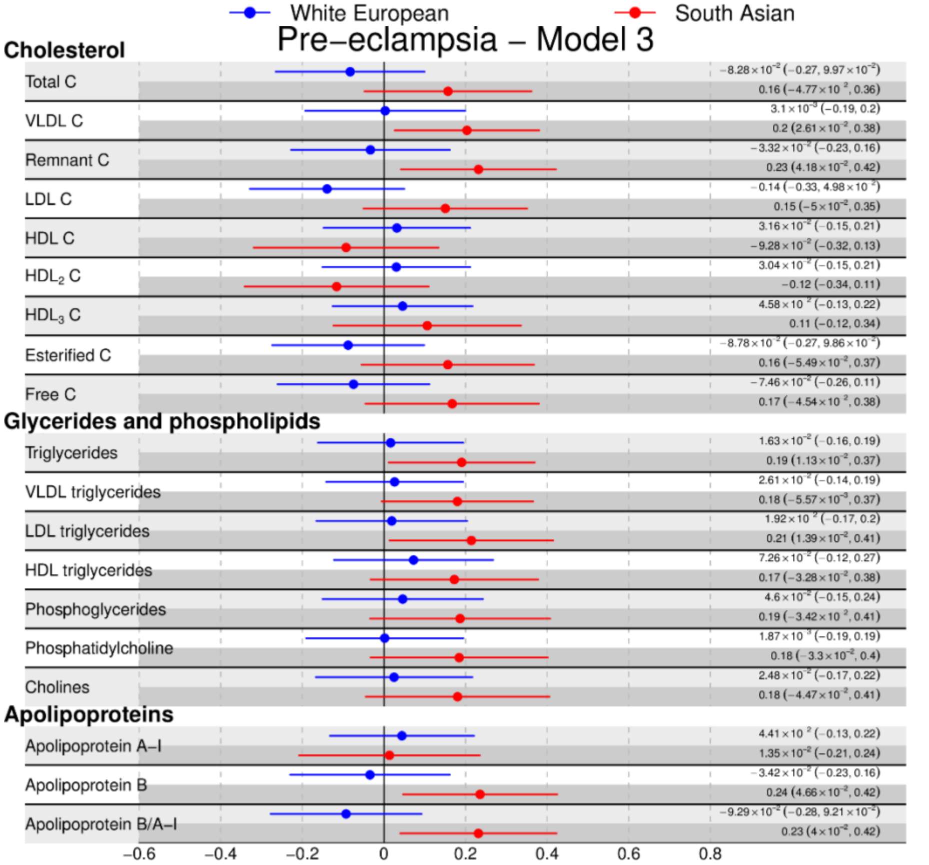

Age adjusted difference in mean metabolite levels (95% CI) comparing women who experienced PE in this pregnancy to those who had no evidence of any HDP

White European South Asian

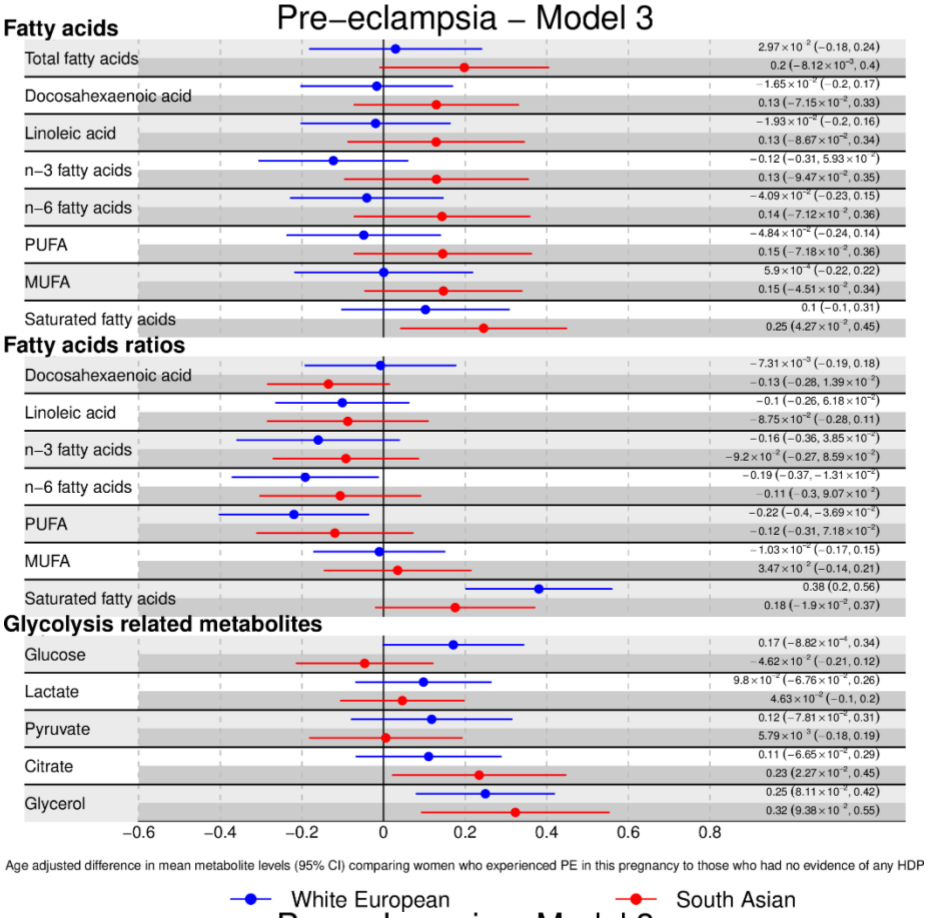
Pre−eclampsia − Model 3
Fatty acids
Total fatty acids
Docosahexaenoic acid
Linoleic acid
n−3 fatty acids
n−6 fatty acids
PUFA
MUFA
Saturated fatty acids
Fatty acids ratios
Docosahexaenoic acid
Linoleic acid
n−3 fatty acids
n−6 fatty acids
PUFA
MUFA
Saturated fatty acids
Glycolysis related metabolites
Glucose
Lactate
Pyruvate
Citrate
Glycerol
−0.6 −0.4 −0.2 0 0.2 0.4 0.6 0.8
Age adjusted difference in mean metabolite levels (95% CI) comparing women who experienced PE in this pregnancy to those who had no evidence of any HDP
White European
South Asian

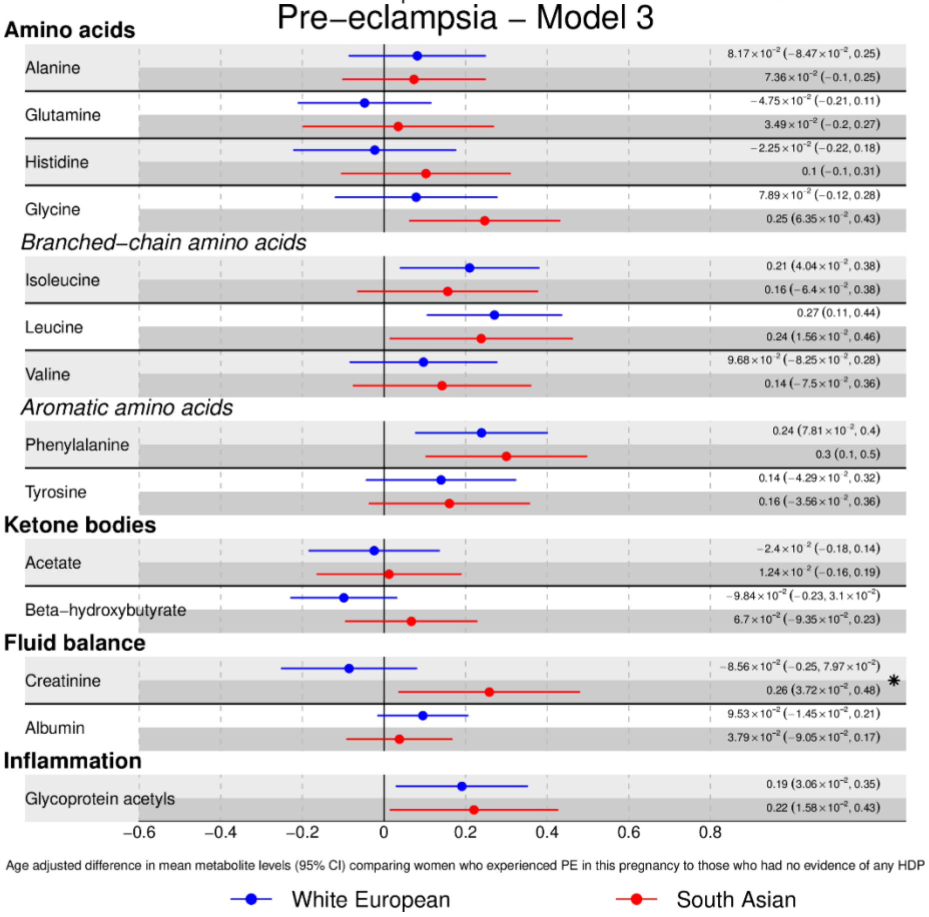
Pre−eclampsia − Model 3
Amino acids
Alanine
Glutamine
Histidine
Glycine
Branched−chain amino acids
Isoleucine
Leucine
Valine
Aromatic amino acids
Phenylalanine
Tyrosine
Ketone bodies
Acetate
Beta−hydroxybutyrate
Fluid balance
Creatinine
Albumin
Inflammation
Glycoprotein acetyls
−0.6 −0.4 −0.2 0 0.2 0.4 0.6 0.8
Age adjusted difference in mean metabolite levels (95% CI) comparing women who experienced PE in this pregnancy to those who had no evidence of any HDP
White European
South Asian

Figure S11. Associations of maternal pre-eclampsia with pregnancy metabolic profiles stratified by ethnicity (model 1 = age-adjusted; model 2 = age-, parity- and education-adjusted, model 3 = age-, parity-, education- and BMI-adjusted). Data points show SD differences for White European (blue) and South Asian (red) women. Error bars = 95% confidence intervals. Exact point estimates and their corresponding 95% CIs are displayed in text to the right of each point. * denotes strong statistical evidence from the interaction test (Pinteraction <0.001). Differences in quantified units (mostly mmol/l) are listed in Supplementary File 2. Abbreviations: VLDL, very low-density lipoprotein; LDL, low-density lipoprotein; HDL, high-density lipoprotein; C, cholesterol; MUFA, monounsaturated fatty acids; PUFA, polyunsaturated fatty acids

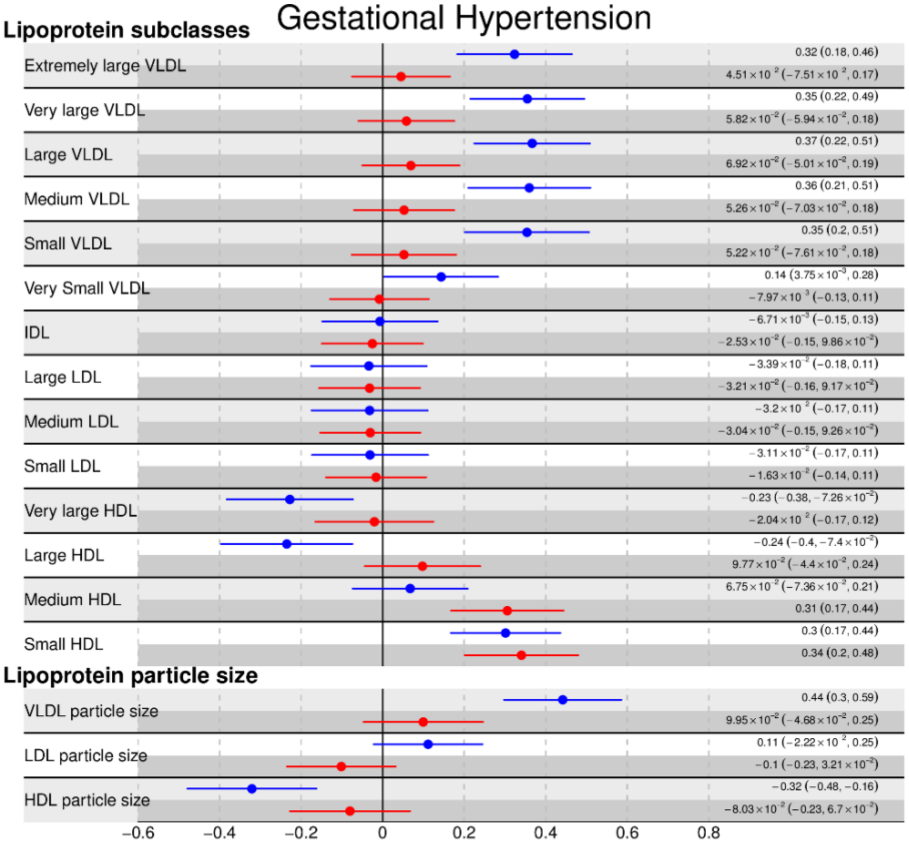

Age adjusted difference in mean metabolite levels (95% CI) comparing women who experienced GHT in this pregnancy to those who had no evidence of any HDP

White European South Asian

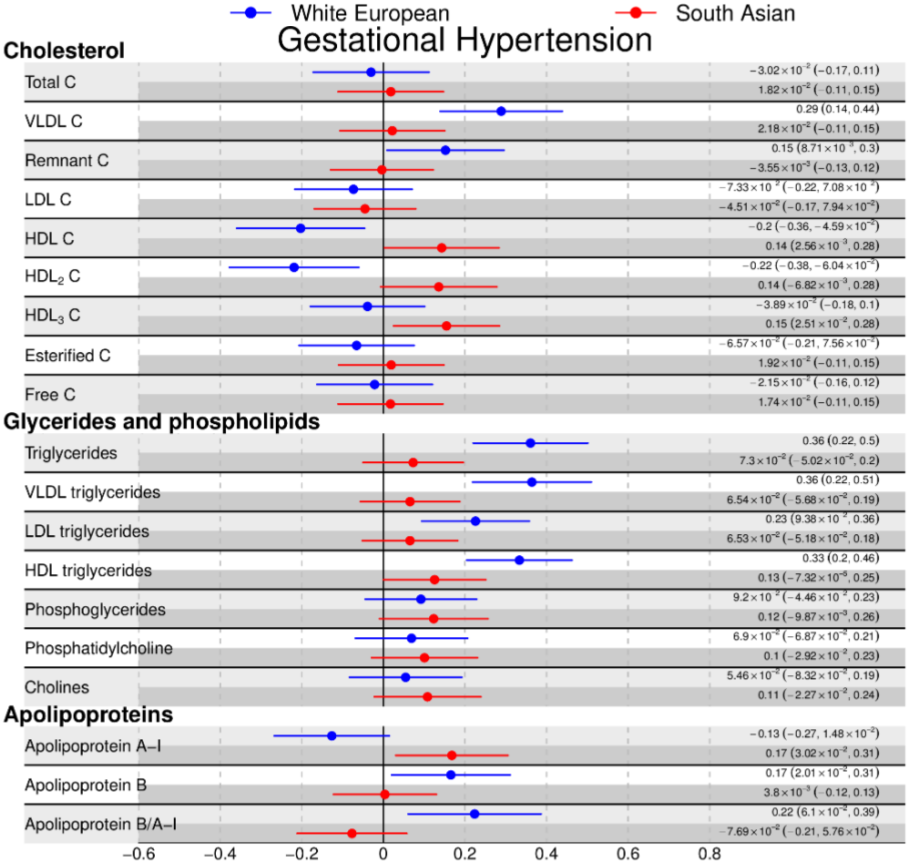

Age adjusted difference in mean metabolite levels (95% CI) comparing women who experienced GHT in this pregnancy to those who had no evidence of any HDP

White European South Asian

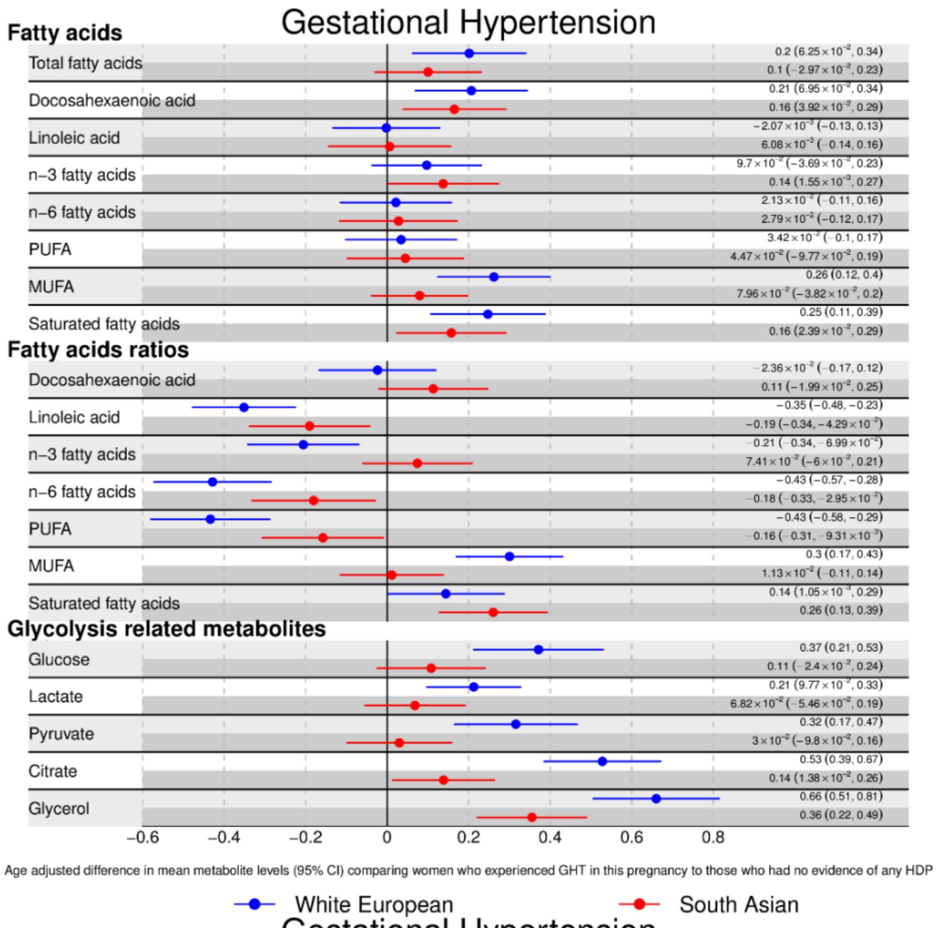
Gestational Hypertension
Fatty acids
Total fatty acids
Docosahexaenoic acid
Linoleic acid
n−3 fatty acids
n−6 fatty acids
PUFA
MUFA
Saturated fatty acids
Fatty acids ratios
Docosahexaenoic acid
Linoleic acid
n−3 fatty acids
n−6 fatty acids
PUFA
MUFA
Saturated fatty acids
Glycolysis related metabolites
Glucose
Lactate
Pyruvate
Citrate
Glycerol
Age adjusted difference in mean metabolite levels (95% CI) comparing women who experienced GHT in this pregnancy to those who had no evidence of any HDP
White European
South Asian

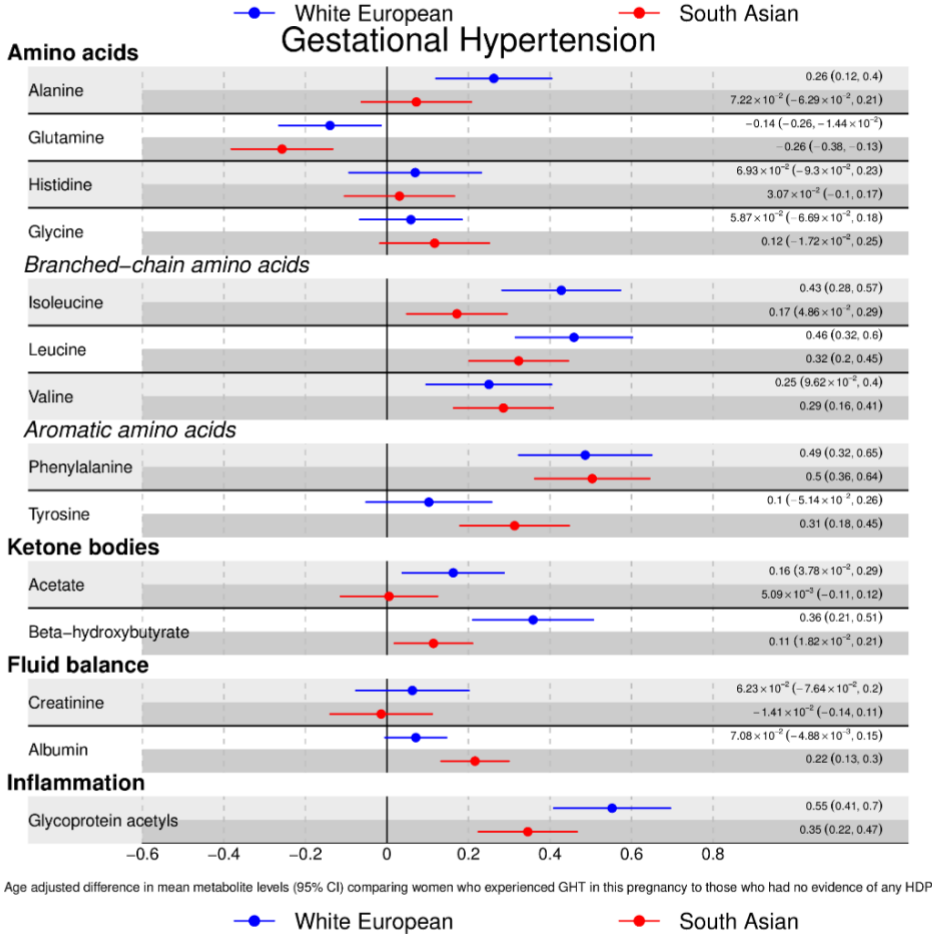
Gestational Hypertension
Amino acids
Alanine
Glutamine
Histidine
Glycine
Branched−chain amino acids
Isoleucine
Leucine
Valine
Aromatic amino acids
Phenylalanine
Tyrosine
Ketone bodies
Acetate
Beta−hydroxybutyrate
Fluid balance
Creatinine
Albumin
Inflammation
Glycoprotein acetyls
Age adjusted difference in mean metabolite levels (95% CI) comparing women who experienced GHT in this pregnancy to those who had no evidence of any HDP
White European
South Asian

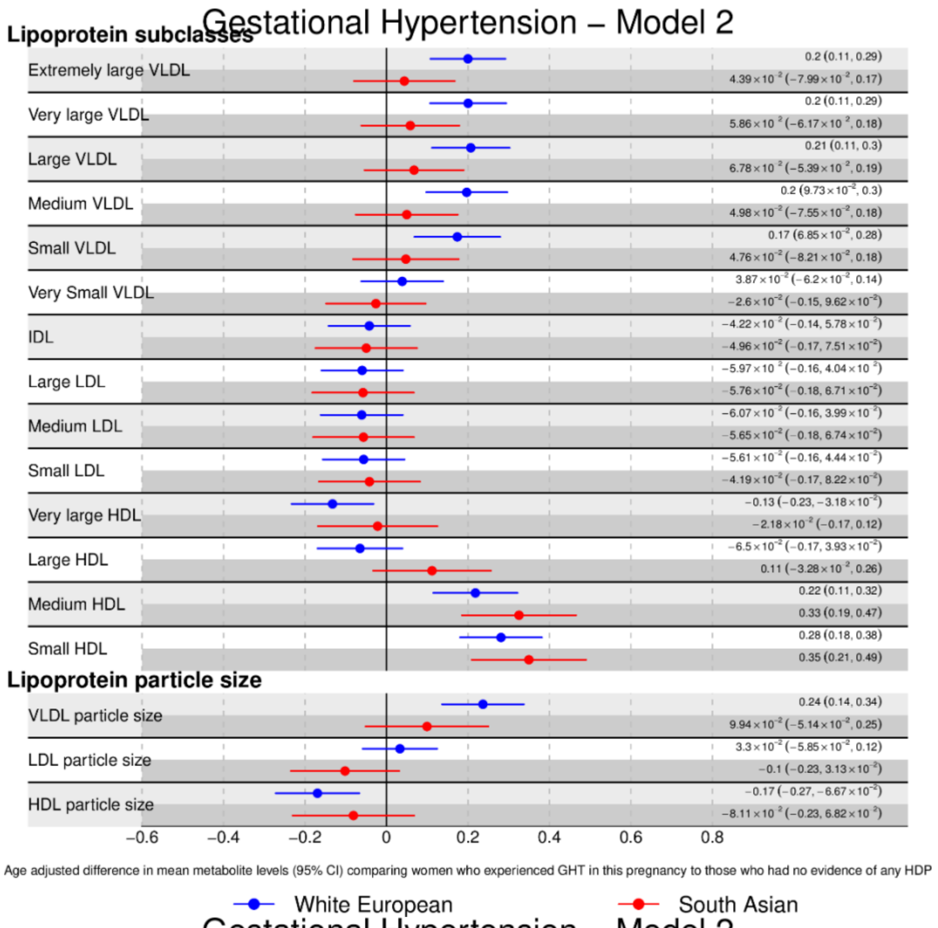

Gestational Hypertension – Model 2
Lipoprotein subclasses
Extremely large VLDL
Very large VLDL
Large VLDL
Medium VLDL
Small VLDL
Very Small VLDL
IDL
Large LDL
Medium LDL
Small LDL
Very large HDL
Large HDL
Medium HDL
Small HDL
Lipoprotein particle size
VLDL particle size
LDL particle size
HDL particle size
0.2 (0.11, 0.29)
0.2 (0.11, 0.29)
0.21 (0.11, 0.3)
0.17 (6.85 × 10⁻², 0.28)
0.22 (0.11, 0.32)
0.33 (0.19, 0.47)
0.28 (0.18, 0.38)
0.35 (0.21, 0.49)
0.24 (0.14, 0.34)
−0.6 −0.4 −0.2 0 0.2 0.4 0.6 0.8
Age adjusted difference in mean metabolite levels (95% CI) comparing women who experienced GHT in this pregnancy to those who had no evidence of any HDP
White European
South Asian

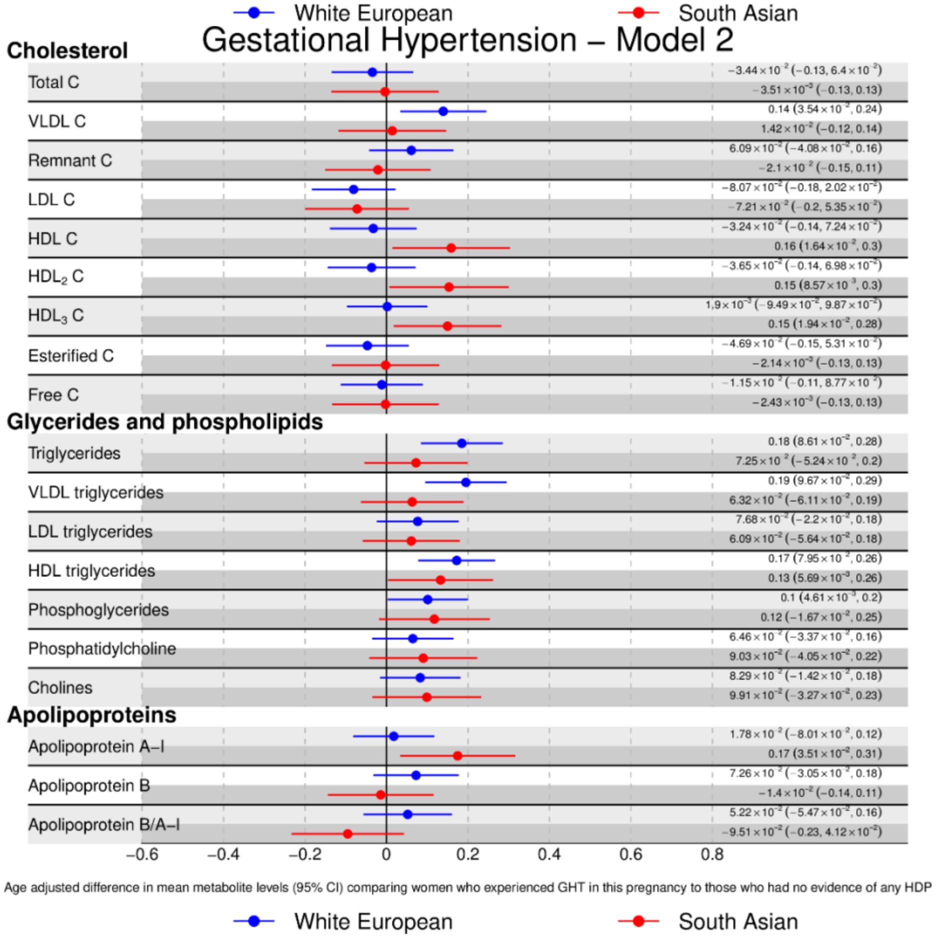

Gestational Hypertension – Model 2
Cholesterol
Total C
VLDL C
Remnant C
LDL C
HDL C
HDL2 C
HDL3 C
Esterified C
Free C
Glycerides and phospholipids
Triglycerides
VLDL triglycerides
LDL triglycerides
HDL triglycerides
Phosphoglycerides
Phosphatidylcholine
Cholines
Apolipoproteins
Apolipoprotein A–I
Apolipoprotein B
Apolipoprotein B/A–I
−0.6 −0.4 −0.2 0 0.2 0.4 0.6 0.8
Age adjusted difference in mean metabolite levels (95% CI) comparing women who experienced GHT in this pregnancy to those who had no evidence of any HDP
White European
South Asian

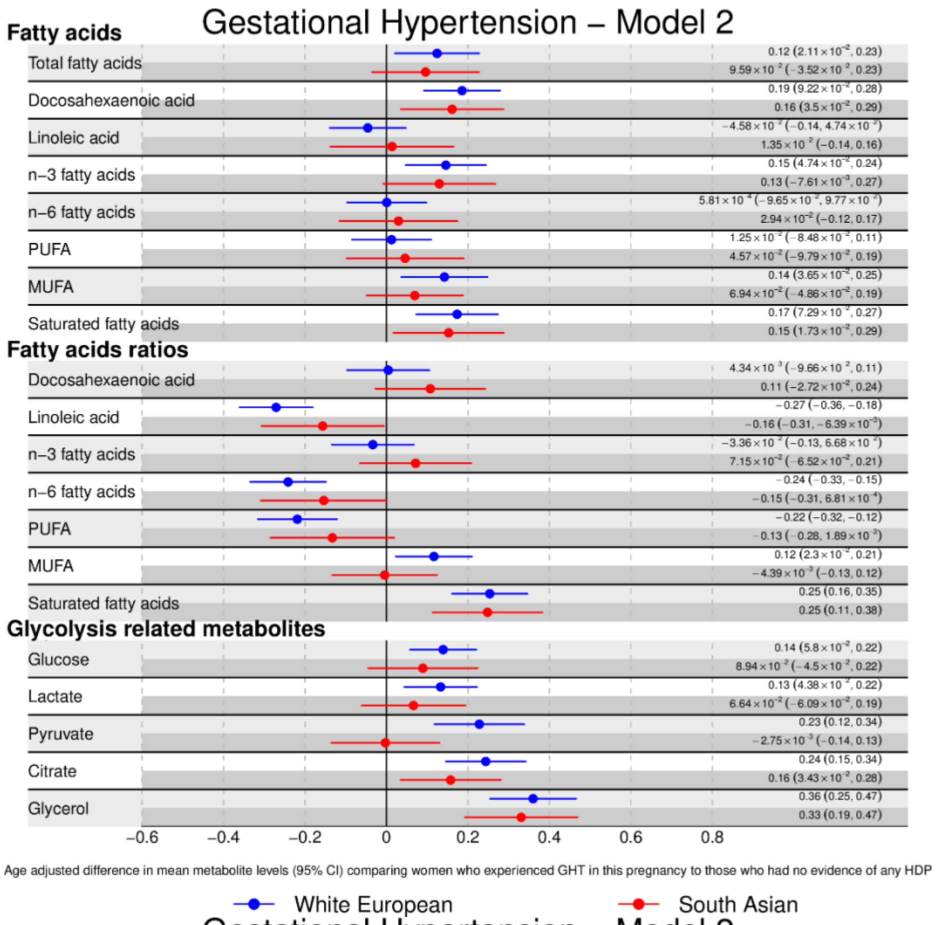
Gestational Hypertension – Model 2
Fatty acids
Total fatty acids
0.12 (2.11×10⁻², 0.23)
9.59×10⁻² (−3.52×10⁻², 0.23)
Docosahexaenoic acid
0.19 (9.22×10⁻², 0.28)
0.16 (3.5×10⁻², 0.29)
Linoleic acid
−4.58×10⁻² (−0.14, 4.74×10⁻²)
1.35×10⁻² (−0.14, 0.16)
n−3 fatty acids
0.15 (4.74×10⁻², 0.24)
0.13 (−7.61×10⁻³, 0.27)
n−6 fatty acids
5.81×10⁻⁴ (−9.65×10⁻², 9.77×10⁻²)
2.94×10⁻² (−0.12, 0.17)
PUFA
1.25×10⁻² (−8.48×10⁻², 0.11)
4.57×10⁻² (−9.79×10⁻², 0.19)
MUFA
0.14 (3.65×10⁻², 0.25)
6.94×10⁻² (−4.86×10⁻², 0.19)
Saturated fatty acids
0.17 (7.29×10⁻², 0.27)
0.15 (1.73×10⁻², 0.29)
Fatty acids ratios
Docosahexaenoic acid
4.34×10⁻³ (−9.66×10⁻², 0.11)
0.11 (−2.72×10⁻², 0.24)
Linoleic acid
−0.27 (−0.36, −0.18)
−0.16 (−0.31, −6.39×10⁻³)
n−3 fatty acids
−3.36×10⁻² (−0.13, 6.68×10⁻²)
7.15×10⁻² (−6.52×10⁻², 0.21)
n−6 fatty acids
−0.24 (−0.33, −0.15)
−0.15 (−0.31, 6.81×10⁻⁴)
PUFA
−0.22 (−0.32, −0.12)
−0.13 (−0.28, 1.89×10⁻²)
MUFA
0.12 (2.3×10⁻², 0.21)
−4.39×10⁻³ (−0.13, 0.12)
Saturated fatty acids
0.25 (0.16, 0.35)
0.25 (0.11, 0.38)
Glycolysis related metabolites
Glucose
0.14 (5.8×10⁻², 0.22)
8.94×10⁻² (−4.5×10⁻², 0.22)
Lactate
0.13 (4.38×10⁻², 0.22)
6.64×10⁻² (−6.09×10⁻², 0.19)
Pyruvate
0.23 (0.12, 0.34)
−2.75×10⁻³ (−0.14, 0.13)
Citrate
0.24 (0.15, 0.34)
0.16 (3.43×10⁻², 0.28)
Glycerol
0.36 (0.25, 0.47)
0.33 (0.19, 0.47)
−0.6
−0.4
−0.2
0
0.2
0.4
0.6
0.8
Age adjusted difference in mean metabolite levels (95% CI) comparing women who experienced GHT in this pregnancy to those who had no evidence of any HDP
White European
South Asian

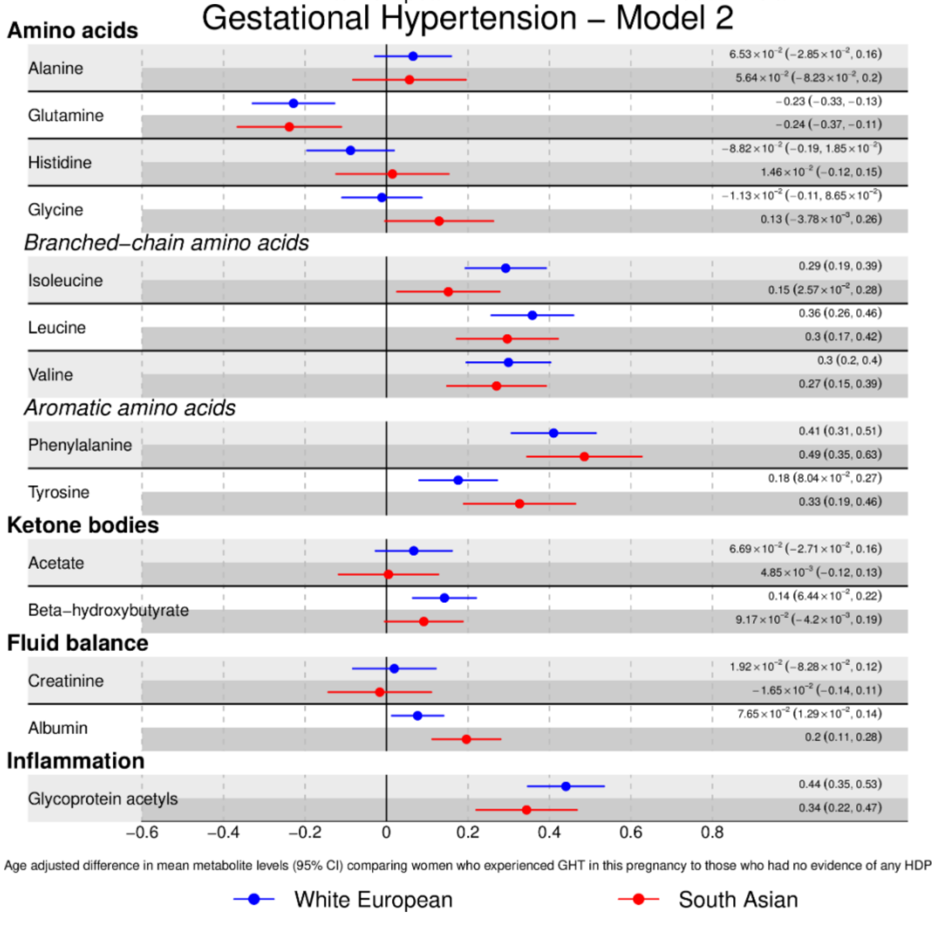
Gestational Hypertension – Model 2
Amino acids
Alanine
6.53×10⁻² (−2.85×10⁻², 0.16)
5.64×10⁻² (−8.23×10⁻², 0.2)
Glutamine
−0.23 (−0.33, −0.13)
−0.24 (−0.37, −0.11)
Histidine
−8.82×10⁻² (−0.19, 1.85×10⁻²)
1.46×10⁻² (−0.12, 0.15)
Glycine
−1.13×10⁻² (−0.11, 8.65×10⁻²)
0.13 (−3.78×10⁻³, 0.26)
Branched−chain amino acids
Isoleucine
0.29 (0.19, 0.39)
0.15 (2.57×10⁻², 0.28)
Leucine
0.36 (0.26, 0.46)
0.3 (0.17, 0.42)
Valine
0.3 (0.2, 0.4)
0.27 (0.15, 0.39)
Aromatic amino acids
Phenylalanine
0.41 (0.31, 0.51)
0.49 (0.35, 0.63)
Tyrosine
0.18 (8.04×10⁻², 0.27)
0.33 (0.19, 0.46)
Ketone bodies
Acetate
6.69×10⁻² (−2.71×10⁻², 0.16)
4.85×10⁻³ (−0.12, 0.13)
Beta−hydroxybutyrate
0.14 (6.44×10⁻², 0.22)
9.17×10⁻² (−4.2×10⁻³, 0.19)
Fluid balance
Creatinine
1.92×10⁻² (−8.28×10⁻², 0.12)
−1.65×10⁻² (−0.14, 0.11)
Albumin
7.65×10⁻² (1.29×10⁻², 0.14)
0.2 (0.11, 0.28)
Inflammation
Glycoprotein acetyls
0.44 (0.35, 0.53)
0.34 (0.22, 0.47)
−0.6
−0.4
−0.2
0
0.2
0.4
0.6
0.8
Age adjusted difference in mean metabolite levels (95% CI) comparing women who experienced GHT in this pregnancy to those who had no evidence of any HDP
White European
South Asian

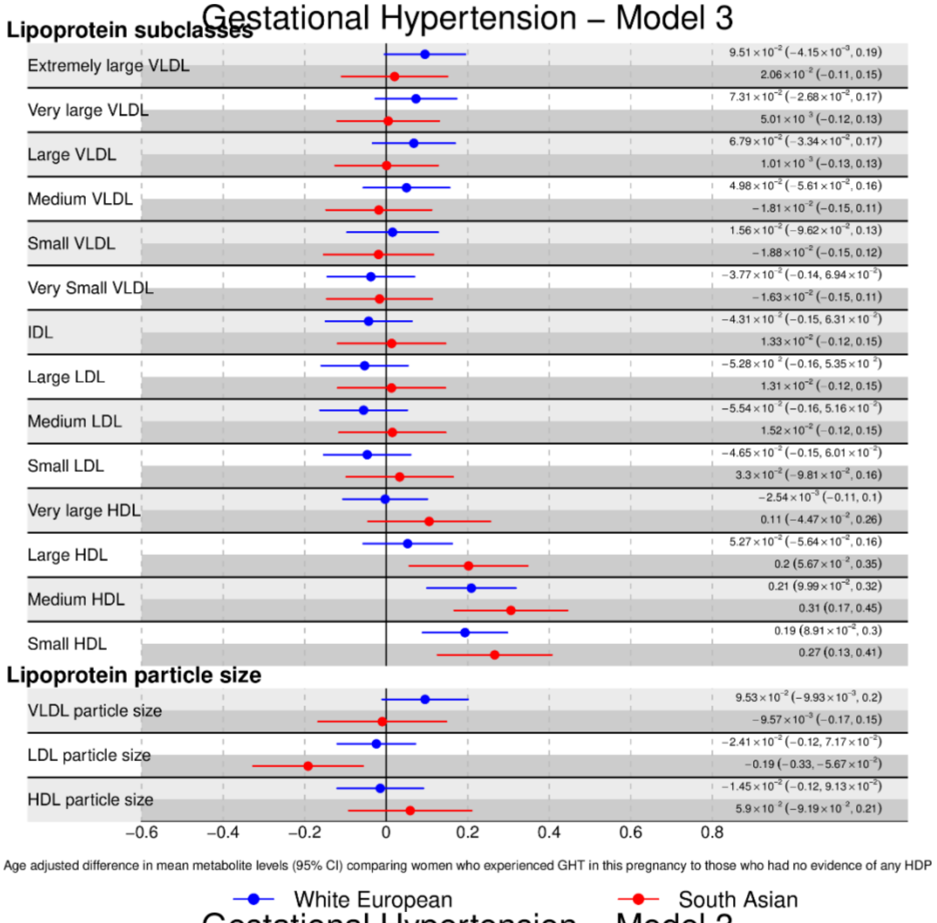
Gestational Hypertension – Model 3
Lipoprotein subclasses
Extremely large VLDL
$9.51\times10^{-2}\ (-4.15\times10^{-3}, 0.19)$
$2.06\times10^{-2}\ (-0.11, 0.15)$
Very large VLDL
$7.31\times10^{-2}\ (-2.68\times10^{-2}, 0.17)$
$5.01\times10^{-3}\ (-0.12, 0.13)$
Large VLDL
$6.79\times10^{-2}\ (-3.34\times10^{-2}, 0.17)$
$1.01\times10^{-3}\ (-0.13, 0.13)$
Medium VLDL
$4.98\times10^{-2}\ (-5.61\times10^{-2}, 0.16)$
$-1.81\times10^{-2}\ (-0.15, 0.11)$
Small VLDL
$1.56\times10^{-2}\ (-9.62\times10^{-2}, 0.13)$
$-1.88\times10^{-2}\ (-0.15, 0.12)$
Very Small VLDL
$-3.77\times10^{-2}\ (-0.14, 6.94\times10^{-2})$
$-1.63\times10^{-2}\ (-0.15, 0.11)$
IDL
$-4.31\times10^{-2}\ (-0.15, 6.31\times10^{-2})$
$1.33\times10^{-2}\ (-0.12, 0.15)$
Large LDL
$-5.28\times10^{-2}\ (-0.16, 5.35\times10^{-2})$
$1.31\times10^{-2}\ (-0.12, 0.15)$
Medium LDL
$-5.54\times10^{-2}\ (-0.16, 5.16\times10^{-2})$
$1.52\times10^{-2}\ (-0.12, 0.15)$
Small LDL
$-4.65\times10^{-2}\ (-0.15, 6.01\times10^{-2})$
$3.3\times10^{-2}\ (-9.81\times10^{-2}, 0.16)$
Very large HDL
$-2.54\times10^{-3}\ (-0.11, 0.1)$
$0.11\ (-4.47\times10^{-2}, 0.26)$
Large HDL
$5.27\times10^{-2}\ (-5.64\times10^{-2}, 0.16)$
$0.2\ (5.67\times10^{-2}, 0.35)$
Medium HDL
$0.21\ (9.99\times10^{-2}, 0.32)$
$0.31\ (0.17, 0.45)$
Small HDL
$0.19\ (8.91\times10^{-2}, 0.3)$
$0.27\ (0.13, 0.41)$
Lipoprotein particle size
VLDL particle size
$9.53\times10^{-2}\ (-9.93\times10^{-3}, 0.2)$
$-9.57\times10^{-3}\ (-0.17, 0.15)$
LDL particle size
$-2.41\times10^{-2}\ (-0.12, 7.17\times10^{-2})$
$-0.19\ (-0.33, -5.67\times10^{-2})$
HDL particle size
$-1.45\times10^{-2}\ (-0.12, 9.13\times10^{-2})$
$5.9\times10^{-2}\ (-9.19\times10^{-2}, 0.21)$
−0.6 −0.4 −0.2 0 0.2 0.4 0.6 0.8
Age adjusted difference in mean metabolite levels (95% CI) comparing women who experienced GHT in this pregnancy to those who had no evidence of any HDP
White European
South Asian

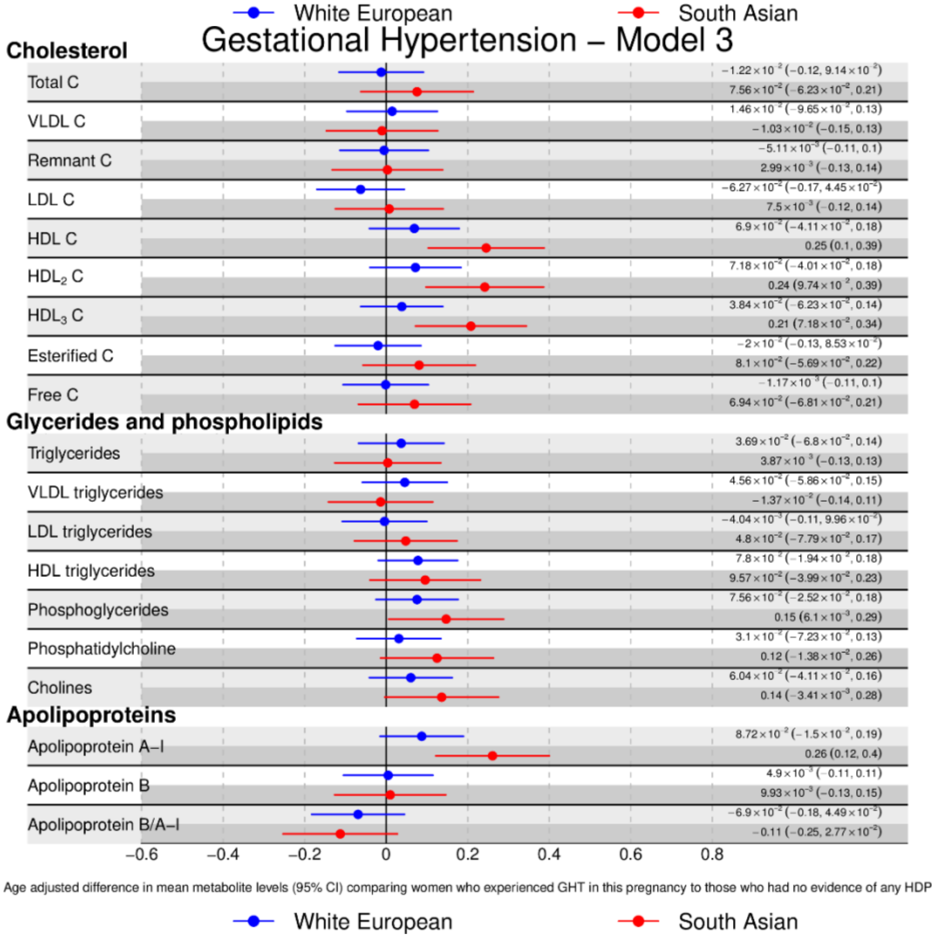
Gestational Hypertension – Model 3
Cholesterol
Total C
$-1.22\times10^{-2}\ (-0.12, 9.14\times10^{-2})$
$7.56\times10^{-2}\ (-6.23\times10^{-2}, 0.21)$
VLDL C
$1.46\times10^{-2}\ (-9.65\times10^{-2}, 0.13)$
$-1.03\times10^{-2}\ (-0.15, 0.13)$
Remnant C
$-5.11\times10^{-3}\ (-0.11, 0.1)$
$2.99\times10^{-3}\ (-0.13, 0.14)$
LDL C
$-6.27\times10^{-2}\ (-0.17, 4.45\times10^{-2})$
$7.5\times10^{-3}\ (-0.12, 0.14)$
HDL C
$6.9\times10^{-2}\ (-4.11\times10^{-2}, 0.18)$
$0.25\ (0.1, 0.39)$
HDL_2 C
$7.18\times10^{-2}\ (-4.01\times10^{-2}, 0.18)$
$0.24\ (9.74\times10^{-2}, 0.39)$
HDL_3 C
$3.84\times10^{-2}\ (-6.23\times10^{-2}, 0.14)$
$0.21\ (7.18\times10^{-2}, 0.34)$
Esterified C
$-2\times10^{-2}\ (-0.13, 8.53\times10^{-2})$
$8.1\times10^{-2}\ (-5.69\times10^{-2}, 0.22)$
Free C
$-1.17\times10^{-3}\ (-0.11, 0.1)$
$6.94\times10^{-2}\ (-6.81\times10^{-2}, 0.21)$
Glycerides and phospholipids
Triglycerides
$3.69\times10^{-2}\ (-6.8\times10^{-2}, 0.14)$
$3.87\times10^{-3}\ (-0.13, 0.13)$
VLDL triglycerides
$4.56\times10^{-2}\ (-5.86\times10^{-2}, 0.15)$
$-1.37\times10^{-2}\ (-0.14, 0.11)$
LDL triglycerides
$-4.04\times10^{-3}\ (-0.11, 9.96\times10^{-2})$
$4.8\times10^{-2}\ (-7.79\times10^{-2}, 0.17)$
HDL triglycerides
$7.8\times10^{-2}\ (-1.94\times10^{-2}, 0.18)$
$9.57\times10^{-2}\ (-3.99\times10^{-2}, 0.23)$
Phosphoglycerides
$7.56\times10^{-2}\ (-2.52\times10^{-2}, 0.18)$
$0.15\ (6.1\times10^{-3}, 0.29)$
Phosphatidylcholine
$3.1\times10^{-2}\ (-7.23\times10^{-2}, 0.13)$
$0.12\ (-1.38\times10^{-2}, 0.26)$
Cholines
$6.04\times10^{-2}\ (-4.11\times10^{-2}, 0.16)$
$0.14\ (-3.41\times10^{-3}, 0.28)$
Apolipoproteins
Apolipoprotein A–I
$8.72\times10^{-2}\ (-1.5\times10^{-2}, 0.19)$
$0.26\ (0.12, 0.4)$
Apolipoprotein B
$4.9\times10^{-3}\ (-0.11, 0.11)$
$9.93\times10^{-3}\ (-0.13, 0.15)$
Apolipoprotein B/A–I
$-6.9\times10^{-2}\ (-0.18, 4.49\times10^{-2})$
$-0.11\ (-0.25, 2.77\times10^{-2})$
−0.6 −0.4 −0.2 0 0.2 0.4 0.6 0.8
Age adjusted difference in mean metabolite levels (95% CI) comparing women who experienced GHT in this pregnancy to those who had no evidence of any HDP
White European
South Asian

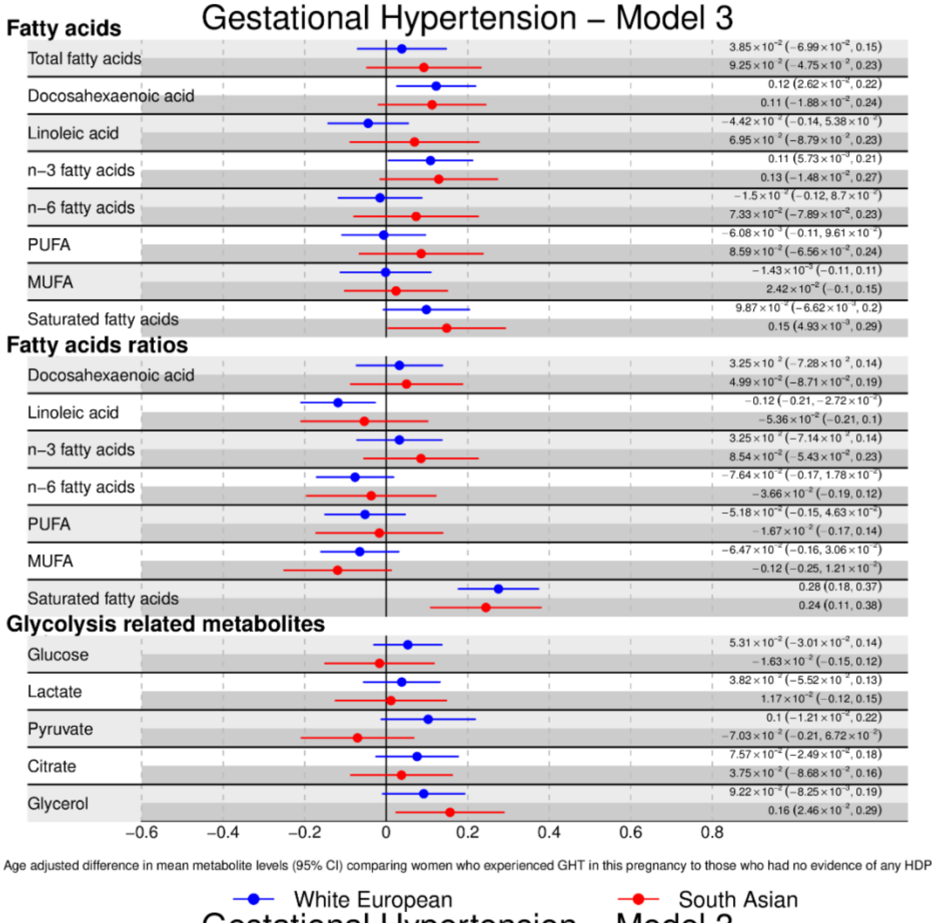

Gestational Hypertension – Model 3
Fatty acids
Total fatty acids
Docosahexaenoic acid
Linoleic acid
n−3 fatty acids
n−6 fatty acids
PUFA
MUFA
Saturated fatty acids
Fatty acids ratios
Docosahexaenoic acid
Linoleic acid
n−3 fatty acids
n−6 fatty acids
PUFA
MUFA
Saturated fatty acids
Glycolysis related metabolites
Glucose
Lactate
Pyruvate
Citrate
Glycerol
3.85×10^{-2} (-6.99×10^{-2}, 0.15)
9.25×10^{-2} (-4.75×10^{-2}, 0.23)
0.12 (2.62×10^{-2}, 0.22)
0.11 (-1.88×10^{-2}, 0.24)
-4.42×10^{-2} (−0.14, 5.38×10^{-2})
6.95×10^{-2} (-8.79×10^{-2}, 0.23)
0.11 (5.73×10^{-3}, 0.21)
0.13 (-1.48×10^{-2}, 0.27)
-1.5×10^{-2} (−0.12, 8.7×10^{-2})
7.33×10^{-2} (-7.89×10^{-2}, 0.23)
-6.08×10^{-3} (−0.11, 9.61×10^{-2})
8.59×10^{-2} (-6.56×10^{-2}, 0.24)
-1.43×10^{-3} (−0.11, 0.11)
2.42×10^{-2} (−0.1, 0.15)
9.87×10^{-2} (-6.62×10^{-3}, 0.2)
0.15 (4.93×10^{-3}, 0.29)
3.25×10^{-2} (-7.28×10^{-2}, 0.14)
4.99×10^{-2} (-8.71×10^{-2}, 0.19)
−0.12 (−0.21, -2.72×10^{-2})
-5.36×10^{-2} (−0.21, 0.1)
3.25×10^{-2} (-7.14×10^{-2}, 0.14)
8.54×10^{-2} (-5.43×10^{-2}, 0.23)
-7.64×10^{-2} (−0.17, 1.78×10^{-2})
-3.66×10^{-2} (−0.19, 0.12)
-5.18×10^{-2} (−0.15, 4.63×10^{-2})
-1.67×10^{-2} (−0.17, 0.14)
-6.47×10^{-2} (−0.16, 3.06×10^{-2})
−0.12 (−0.25, 1.21×10^{-2})
0.28 (0.18, 0.37)
0.24 (0.11, 0.38)
5.31×10^{-2} (-3.01×10^{-2}, 0.14)
-1.63×10^{-2} (−0.15, 0.12)
3.82×10^{-2} (-5.52×10^{-2}, 0.13)
1.17×10^{-2} (−0.12, 0.15)
0.1 (-1.21×10^{-2}, 0.22)
-7.03×10^{-2} (−0.21, 6.72×10^{-2})
7.57×10^{-2} (-2.49×10^{-2}, 0.18)
3.75×10^{-2} (-8.68×10^{-2}, 0.16)
9.22×10^{-2} (-8.25×10^{-3}, 0.19)
0.16 (2.46×10^{-2}, 0.29)
−0.6
−0.4
−0.2
0
0.2
0.4
0.6
0.8
Age adjusted difference in mean metabolite levels (95% CI) comparing women who experienced GHT in this pregnancy to those who had no evidence of any HDP
White European
South Asian

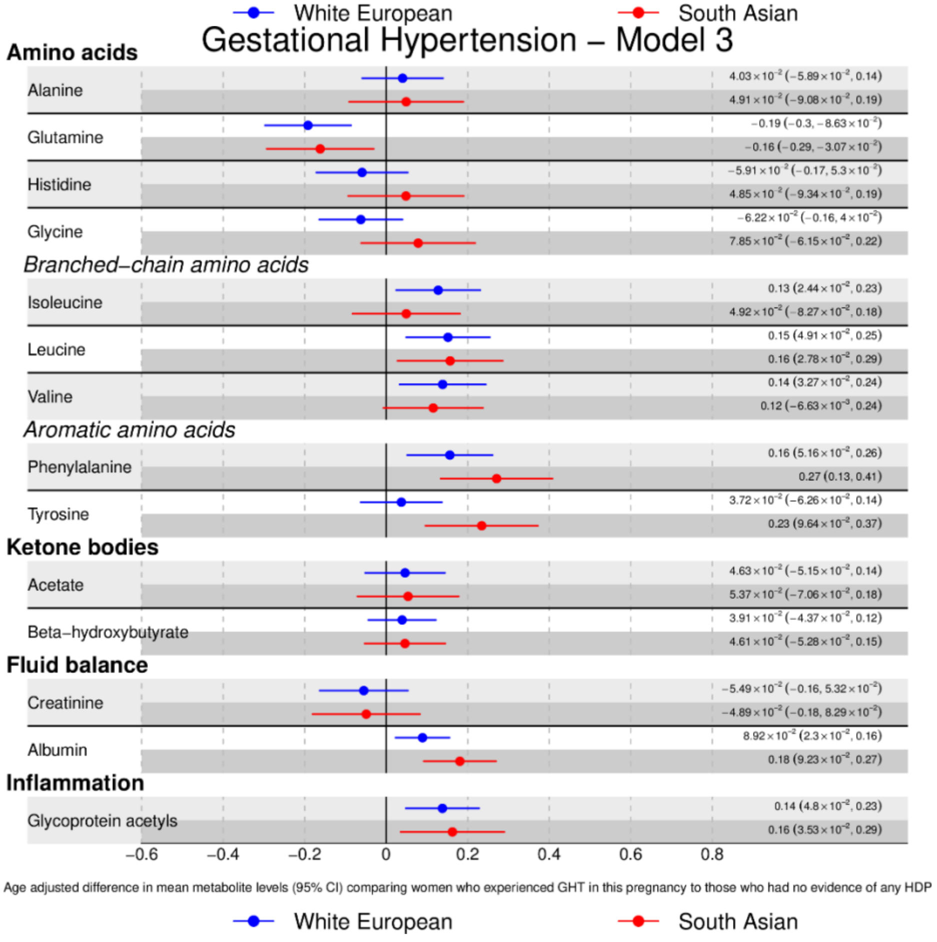

Gestational Hypertension – Model 3
Amino acids
Alanine
Glutamine
Histidine
Glycine
Branched−chain amino acids
Isoleucine
Leucine
Valine
Aromatic amino acids
Phenylalanine
Tyrosine
Ketone bodies
Acetate
Beta−hydroxybutyrate
Fluid balance
Creatinine
Albumin
Inflammation
Glycoprotein acetyls
4.03×10^{-2} (-5.89×10^{-2}, 0.14)
4.91×10^{-2} (-9.08×10^{-2}, 0.19)
−0.19 (−0.3, -8.63×10^{-2})
−0.16 (−0.29, -3.07×10^{-2})
-5.91×10^{-2} (−0.17, 5.3×10^{-2})
4.85×10^{-2} (-9.34×10^{-2}, 0.19)
-6.22×10^{-2} (−0.16, 4×10^{-2})
7.85×10^{-2} (-6.15×10^{-2}, 0.22)
0.13 (2.44×10^{-2}, 0.23)
4.92×10^{-2} (-8.27×10^{-2}, 0.18)
0.15 (4.91×10^{-2}, 0.25)
0.16 (2.78×10^{-2}, 0.29)
0.14 (3.27×10^{-2}, 0.24)
0.12 (-6.63×10^{-3}, 0.24)
0.16 (5.16×10^{-2}, 0.26)
0.27 (0.13, 0.41)
3.72×10^{-2} (-6.26×10^{-2}, 0.14)
0.23 (9.64×10^{-2}, 0.37)
4.63×10^{-2} (-5.15×10^{-2}, 0.14)
5.37×10^{-2} (-7.06×10^{-2}, 0.18)
3.91×10^{-2} (-4.37×10^{-2}, 0.12)
4.61×10^{-2} (-5.28×10^{-2}, 0.15)
-5.49×10^{-2} (−0.16, 5.32×10^{-2})
-4.89×10^{-2} (−0.18, 8.29×10^{-2})
8.92×10^{-2} (2.3×10^{-2}, 0.16)
0.18 (9.23×10^{-2}, 0.27)
0.14 (4.8×10^{-2}, 0.23)
0.16 (3.53×10^{-2}, 0.29)
−0.6
−0.4
−0.2
0
0.2
0.4
0.6
0.8
Age adjusted difference in mean metabolite levels (95% CI) comparing women who experienced GHT in this pregnancy to those who had no evidence of any HDP
White European
South Asian

Figure S12. Associations of maternal gestational hypertension with pregnancy metabolic profiles stratified by ethnicity (model 1 = age-adjusted; model 2 = age-, parity- and education-adjusted, model 3 = age-, parity-, education- and BMI-adjusted). Data points show SD differences for White European (blue) and South Asian (red) women. Error bars = 95% confidence intervals. Exact point estimates and their corresponding 95% CIs are displayed in text to the right of each point. * denotes strong statistical evidence from the interaction test (Pinteraction <0.001). Differences in quantified units (mostly mmol/l) are listed in Supplementary File 2. Abbreviations: VLDL, very low-density lipoprotein; LDL, low-density lipoprotein; HDL, high-density lipoprotein; C, cholesterol; MUFA, monounsaturated fatty acids; PUFA, polyunsaturated fatty acids

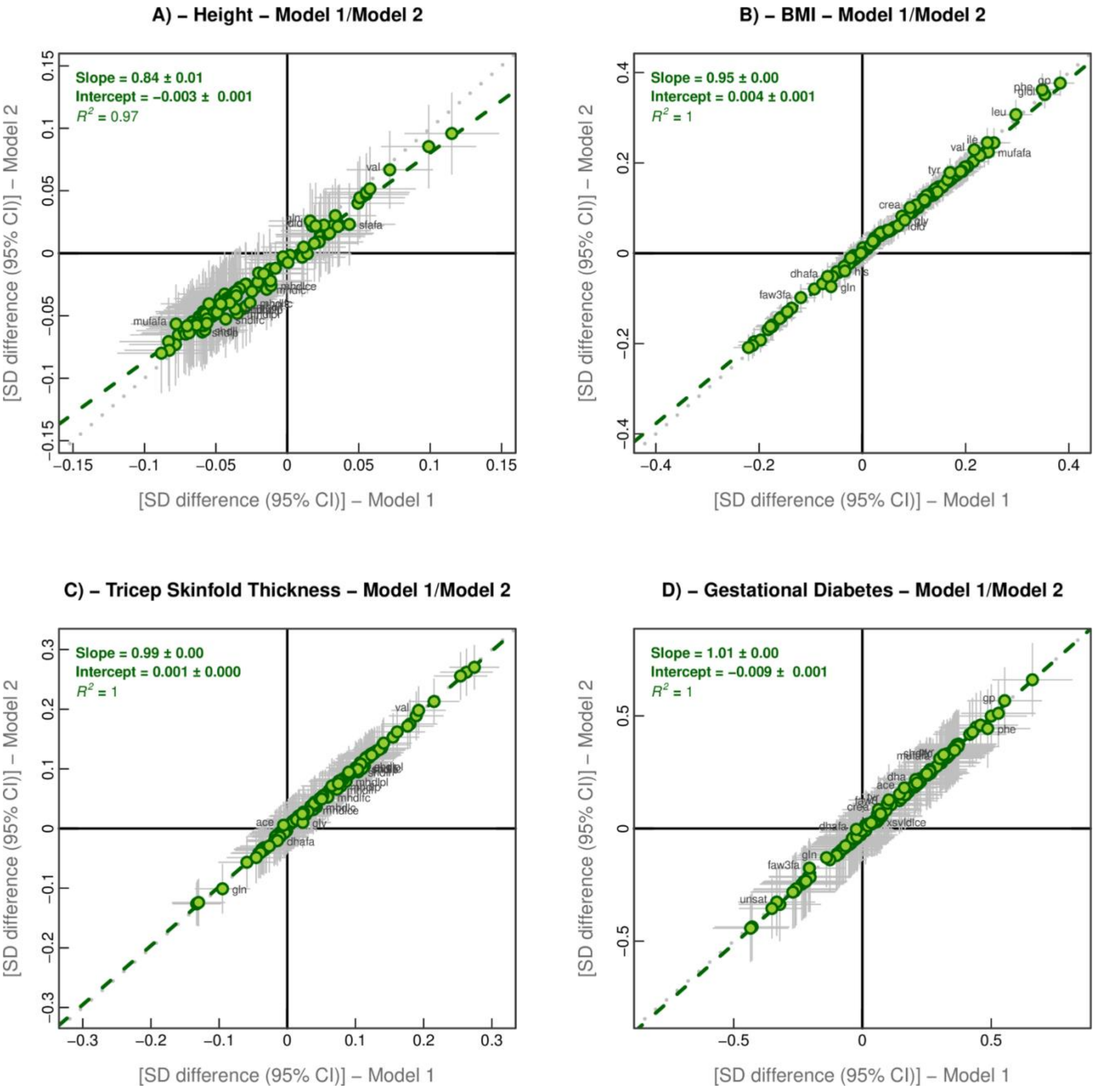

Figure S13. Linear fit between regression model 1 (age adjusted) and regression model 2 (age, parity, education adjusted) represented by the green dashed line for maternal: height (a), BMI (b), tricep skinfold thickness (c), and gestational diabetes (d) in *White European* women. Each green dot represents a metabolic trait. The positions of the dots are determined by difference in mean maternal metabolite (in SD units) for each increase of 1-SD maternal exposure in model 1 analyses (x-axis) and difference in mean maternal metabolite (in SD units) for each increase in 1-SD maternal exposure in model 2 analyses (y-axis). For binary exposures, the differences are in SD units between the exposures and non-exposed. The horizontal grey lines on each dot denote the confidence intervals (CI) for model 1 associations and the vertical grey lines indicate the CI for model 2 estimates. A linear fit of the overall concordance (the similarity in magnitude between model 1 and model 2 associations) is shown by the green dashed line. A slope of 1 with an intercept of 0 (dashed grey line), with all green dots sitting on that line, would indicate that model 1 and model 2 results had the same magnitude and direction. Note for some exposures (e.g. with Tricep skin folds and gestational diabetes) the concordance is so high that the dotted grey line is difficult to see as the linear fit lie from the data (green dashed) covers it. The proportion of variance in model 2 associations that is explained by model 1 associations (R^2) in these analyses provides a measure of goodness of linear fit, with higher values (closer to 1) indicating higher consistency between models.

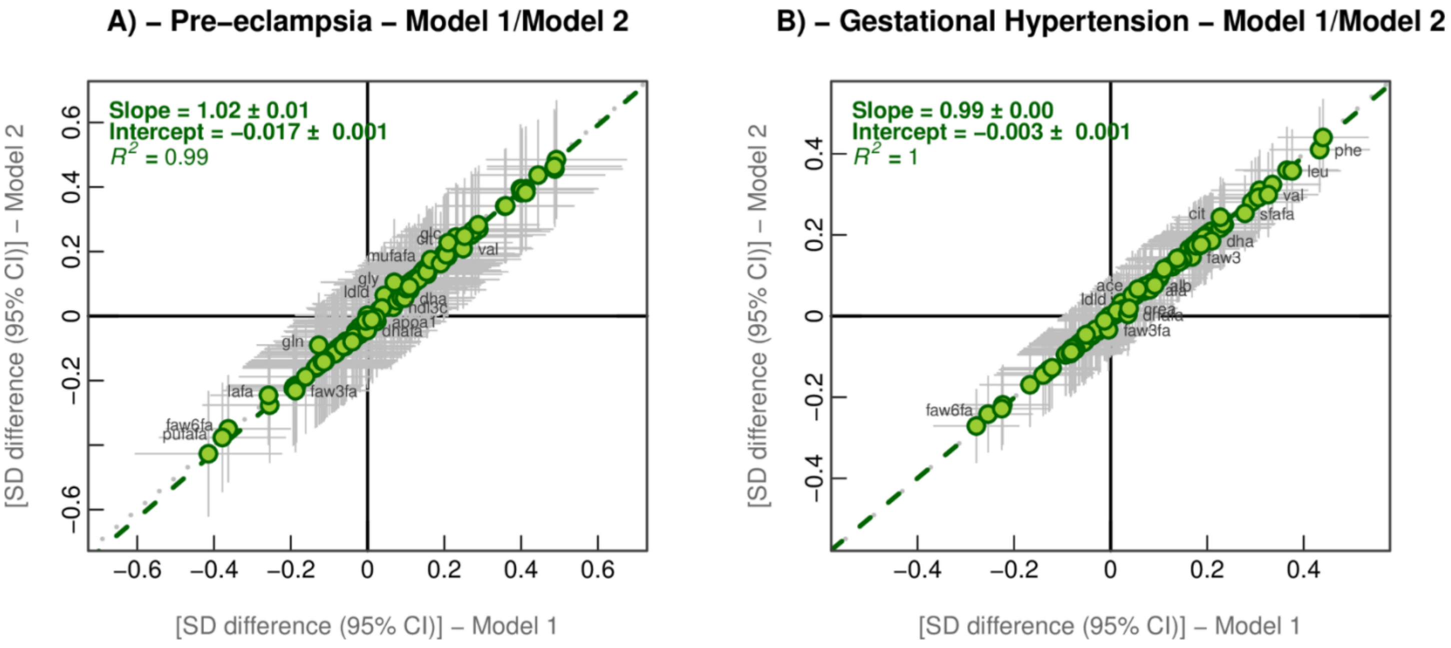

Figure S14. Linear fit between regression model 1 (age adjusted) and regression model 2 (age, parity, education adjusted) represented by the green dashed line for maternal: pre-eclampsia (a) and gestational hypertension (b) in *White European* women. Each green dot represents a metabolic trait. The positions of the dots are determined by difference in mean maternal metabolite (in SD units) for each increase of 1-SD maternal exposure in model 1 analyses (x-axis) and difference in mean maternal metabolite (in SD units) for each increase in 1-SD maternal exposure in model 2 analyses (y-axis). For binary exposures, the differences are in SD units between the exposures and non-exposed. The horizontal grey lines on each dot denote the confidence intervals (CI) for model 1 associations and the vertical grey lines indicate the CI for model 2 estimates. A linear fit of the overall concordance (the similarity in magnitude between model 1 and model 2 associations) is shown by the green dashed line. A slope of 1 with an intercept of 0 (dashed grey line), with all green dots sitting on that line, would indicate that model 1 and model 2 results had the same magnitude and direction. Note for some exposures (e.g. with Tricep skin folds and gestational diabetes) the concordance is so high that the dotted grey line is difficult to see as the linear fit lie from the data (green dashed) covers it. The proportion of variance in model 2 associations that is explained by model 1 associations (R2) in these analyses provides a measure of goodness of linear fit, with higher values (closer to 1) indicating higher consistency between models.

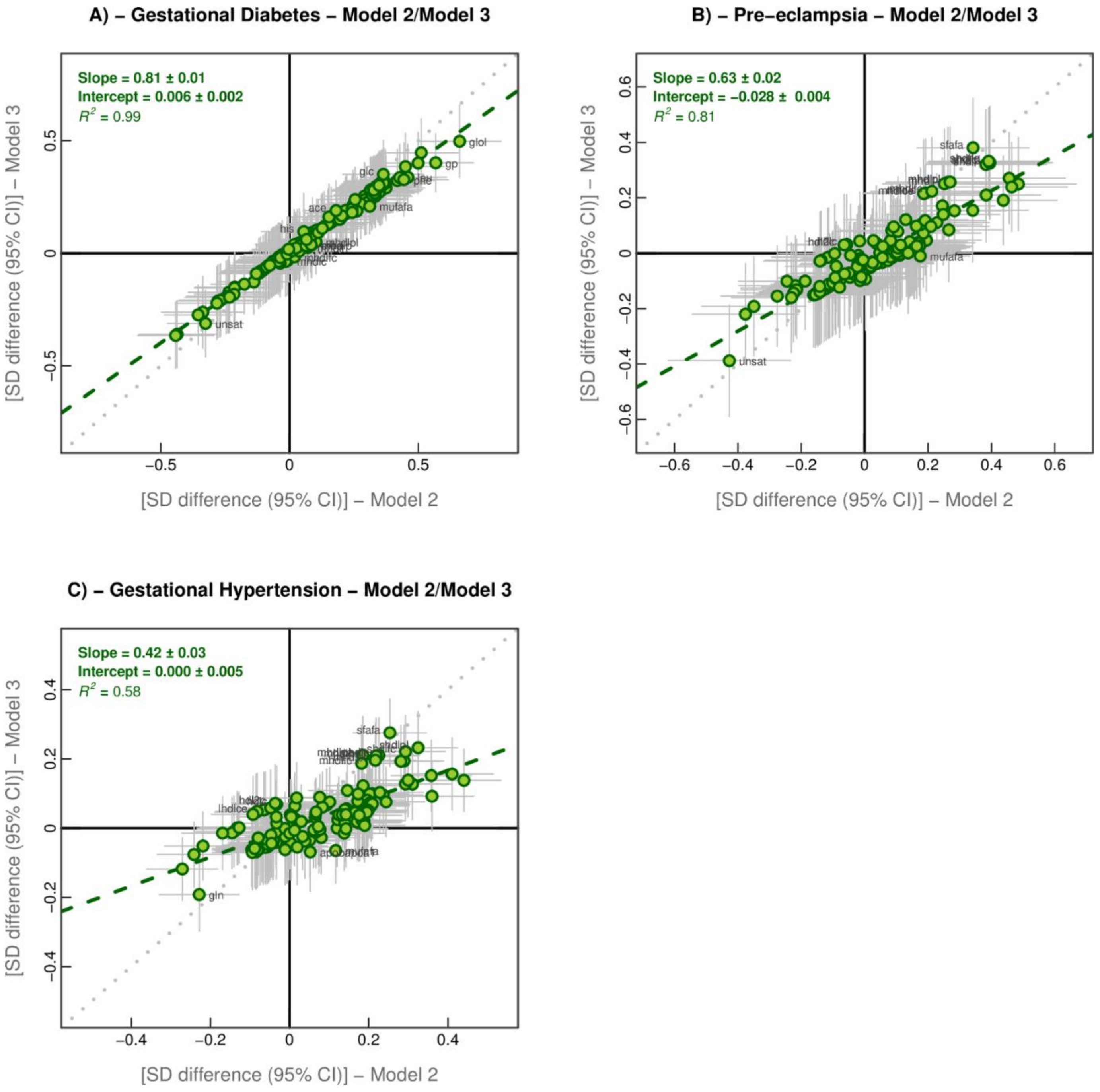

Figure S15. Linear fit between regression model 2 (age, parity, education adjusted) and regression model 3 (age, parity, education, BMI adjusted) represented by the green dashed line for maternal: gestational diabetes (a), pre-eclampsia (b) and gestational hypertension (c) in *White European* women. Each green dot represents a metabolic trait. The positions of the dots are determined by difference in mean maternal metabolite (in SD units) for each increase of 1-SD maternal exposure in model 2 analyses (x-axis) and difference in mean maternal metabolite (in SD units) for each increase in 1-SD maternal exposure in model 3 analyses (y-axis). For binary exposures, the differences are in SD units between the exposures and non-exposed. The horizontal grey lines on each dot denote the confidence intervals (CI) for model 1 associations and the vertical grey lines indicate the CI for model 2 estimates. A linear fit of the overall concordance (the similarity in magnitude between model 1 and model 2 associations) is shown by the green dashed line. A slope of 1 with an intercept of 0 (dashed grey line), with all green dots sitting on that line, would indicate that model 1 and model 2 results had the same magnitude and direction. The proportion of variance in model 2 associations that is explained by model 1 associations (R2) in these analyses provides a measure of goodness of linear fit, with higher values (closer to 1) indicating higher consistency between models.

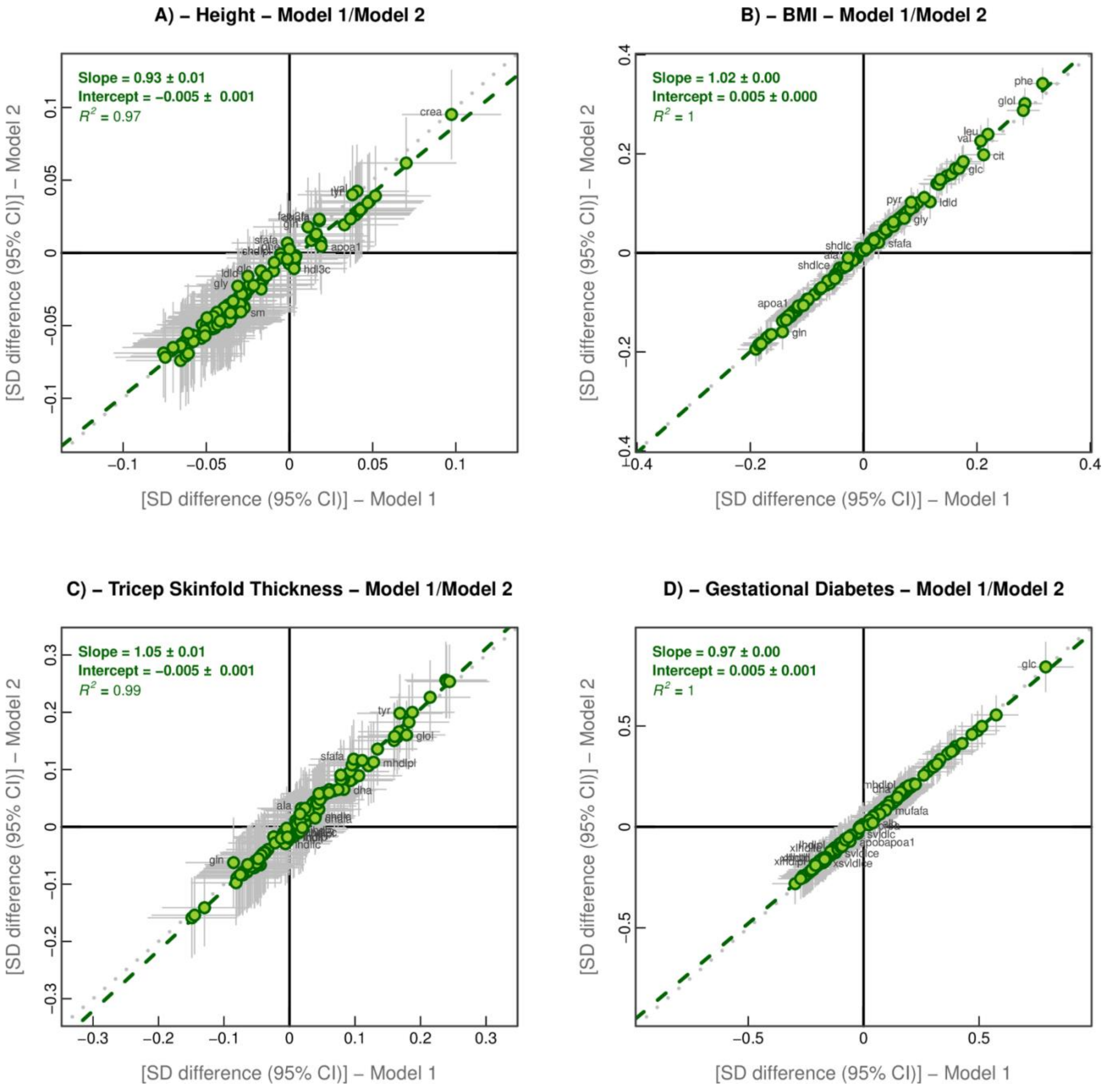

Figure S16. Linear fit between regression model 1 (age adjusted) and regression model 2 (age, parity, education adjusted) represented by the green dashed line for maternal: height (a), BMI (b), tricep skinfold thickness (c), and gestational diabetes (d) in *South Asian* women. Each green dot represents a metabolic trait. The positions of the dots are determined by difference in mean maternal metabolite (in SD units) for each increase of 1-SD maternal exposure in model 1 analyses (x-axis) and difference in mean maternal metabolite (in SD units) for each increase in 1-SD maternal exposure in model 2 analyses (y-axis). For binary exposures, the differences are in SD units between the exposures and non-exposed. The horizontal grey lines on each dot denote the confidence intervals (CI) for model 1 associations and the vertical grey lines indicate the CI for model 2 estimates. A linear fit of the overall concordance (the similarity in magnitude between model 1 and model 2 associations) is shown by the green dashed line. A slope of 1 with an intercept of 0 (dashed grey line), with all green dots sitting on that line, would indicate that model 1 and model 2 results had the same magnitude and direction. The proportion of variance in model 2 associations that is explained by model 1 associations (R^2) in these analyses provides a measure of goodness of linear fit, with higher values (closer to 1) indicating higher consistency between models.

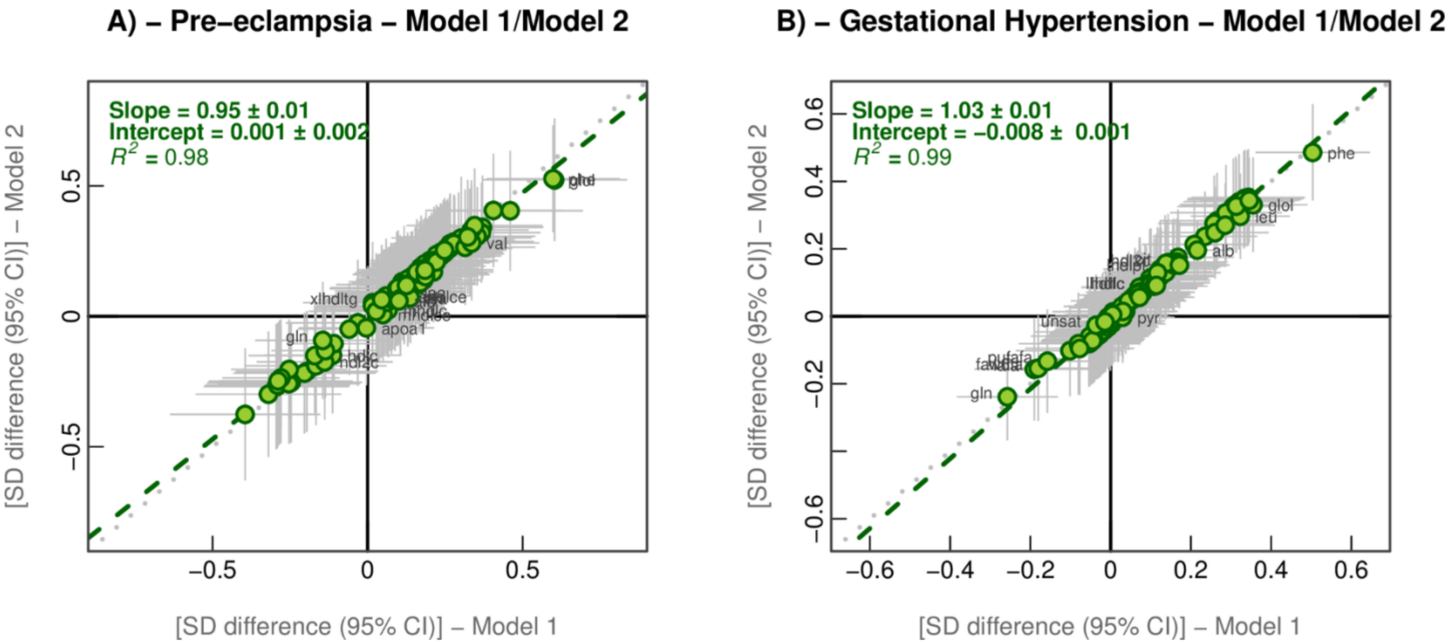

Figure S17. Linear fit between regression model 1 (age adjusted) and regression model 2 (age, parity, education adjusted) represented by the green dashed line for maternal: pre-eclampsia (a) and gestational hypertension(b) in *South Asian* women. Each green dot represents a metabolic trait. The positions of the dots are determined by difference in mean maternal metabolite (in SD units) for each increase of 1-SD maternal exposure in model 1 analyses (x-axis) and difference in mean maternal metabolite (in SD units) for each increase in 1-SD maternal exposure in model 2 analyses (y-axis). For binary exposures, the differences are in SD units between the exposures and non-exposed. The horizontal grey lines on each dot denote the confidence intervals (CI) for model 1 associations and the vertical grey lines indicate the CI for model 2 estimates. A linear fit of the overall concordance (the similarity in magnitude between model 1 and model 2 associations) is shown by the green dashed line. A slope of 1 with an intercept of 0 (dashed grey line), with all green dots sitting on that line, would indicate that model 1 and model 2 results had the same magnitude and direction. The proportion of variance in model 2 associations that is explained by model 1 associations (R^2) in these analyses provides a measure of goodness of linear fit, with higher values (closer to 1) indicating higher consistency between models.

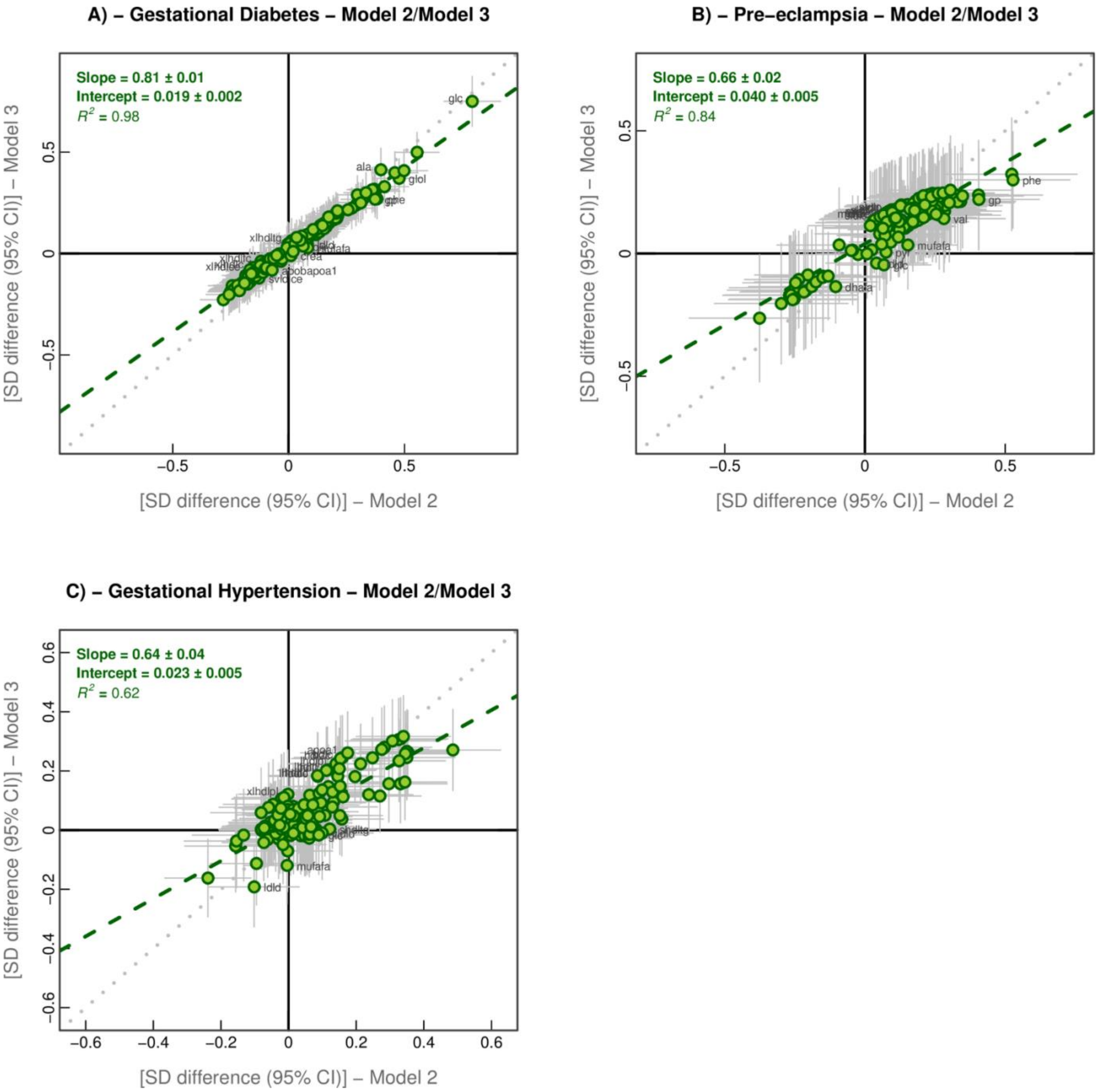

Figure S18. Linear fit between regression model 2 (age, parity, education adjusted) and regression model 3 (age, parity, education, BMI adjusted) represented by the green dashed line for maternal: gestational diabetes (a), pre-eclampsia (b) and gestational hypertension (c) in *South Asian* women. Each green dot represents a metabolic trait. The positions of the dots are determined by difference in mean maternal metabolite (in SD units) for each increase of 1-SD maternal exposure in model 2 analyses (x-axis) and difference in mean maternal metabolite (in SD units) for each increase in 1-SD maternal exposure in model 3 analyses (y-axis). For binary exposures, the differences are in SD units between the exposures and non-exposed. The horizontal grey lines on each dot denote the confidence intervals (CI) for model 1 associations and the vertical grey lines indicate the CI for model 2 estimates. A linear fit of the overall concordance (the similarity in magnitude between model 1 and model 2 associations) is shown by the green dashed line. A slope of 1 with an intercept of 0 (dashed grey line), with all green dots sitting on that line, would indicate that model 1 and model 2 results had the same magnitude and direction. The proportion of variance in model 2 associations that is explained by model 1 associations (R2) in these analyses provides a measure of goodness of linear fit, with higher values (closer to 1) indicating higher consistency between models.

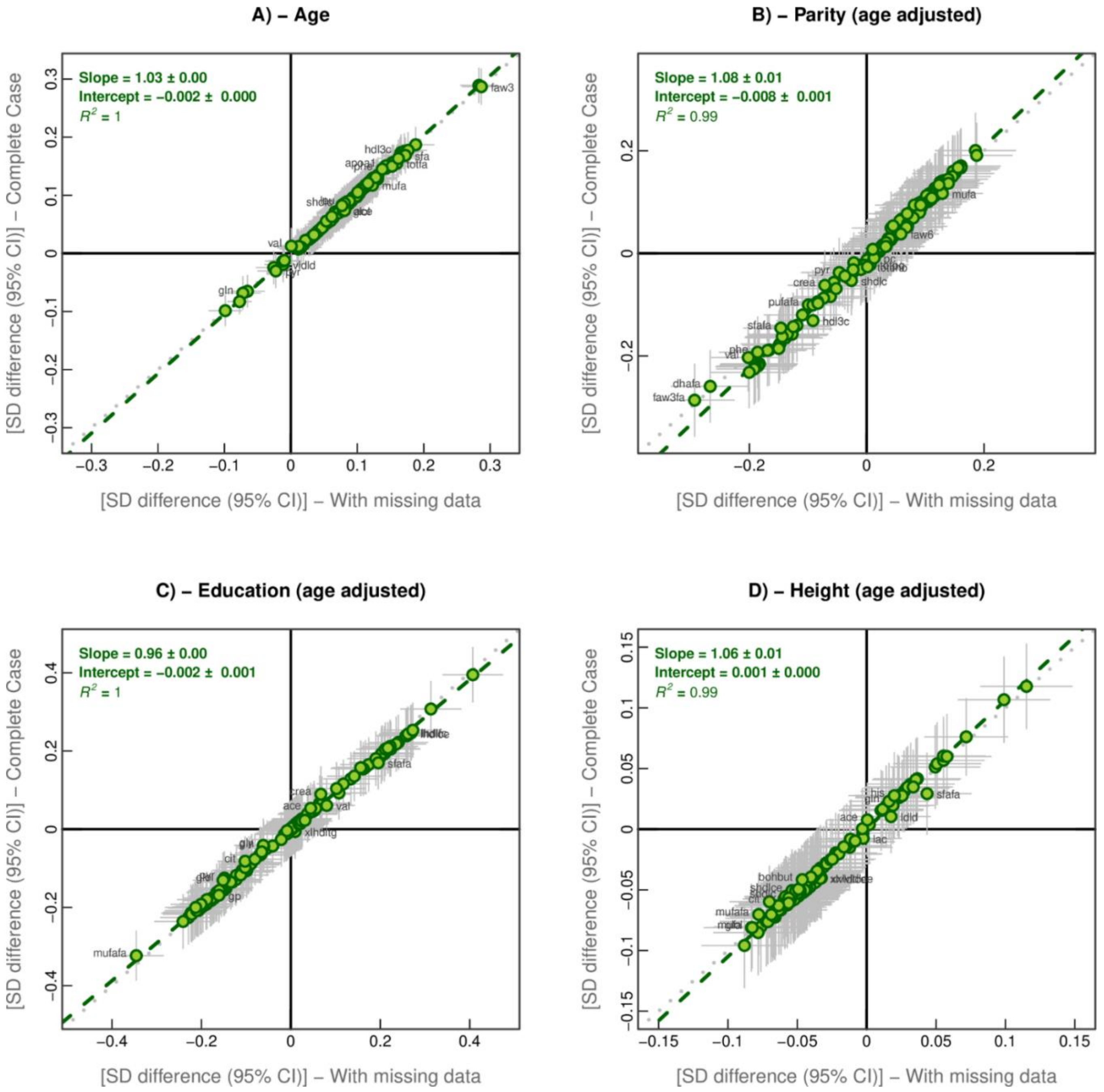

Figure S19. Sensitivity Analysis 1: Linear fit between missing data and complete case analyses for age-adjusted models (green dashed line): maternal age (a), parity (b), education (c), and height (d) in *White European* women. Each green dot represents a metabolic trait. The positions of the dots are determined by difference in mean maternal metabolite (in SD units) for each increase of 1-SD maternal exposure in the main analyses whereby data were not complete (x-axis) and difference in mean maternal metabolite (in SD units) for each increase in 1-SD maternal exposure in complete-case analyses (y-axis). For dichotomous exposures, the differences are in SD units between the exposures and non-exposed. Missing data is described in table 1 of the main text. The horizontal grey lines on each dot denote the confidence intervals (CI) for main analyses associations and the vertical grey lines indicate the CI for complete-case estimates. A linear fit of the overall concordance (the similarity in magnitude between complete case and non complete case associations) is shown by the green dashed line. A slope of 1 with an intercept of 0 (dashed grey line), with all green dots sitting on that line, would indicate that results had the same magnitude and direction. The proportion of variance in non complete case associations that is explained by complete case associations (R2) in these analyses provides a measure of goodness of linear fit, with higher values (closer to 1) indicating higher consistency between complete case.

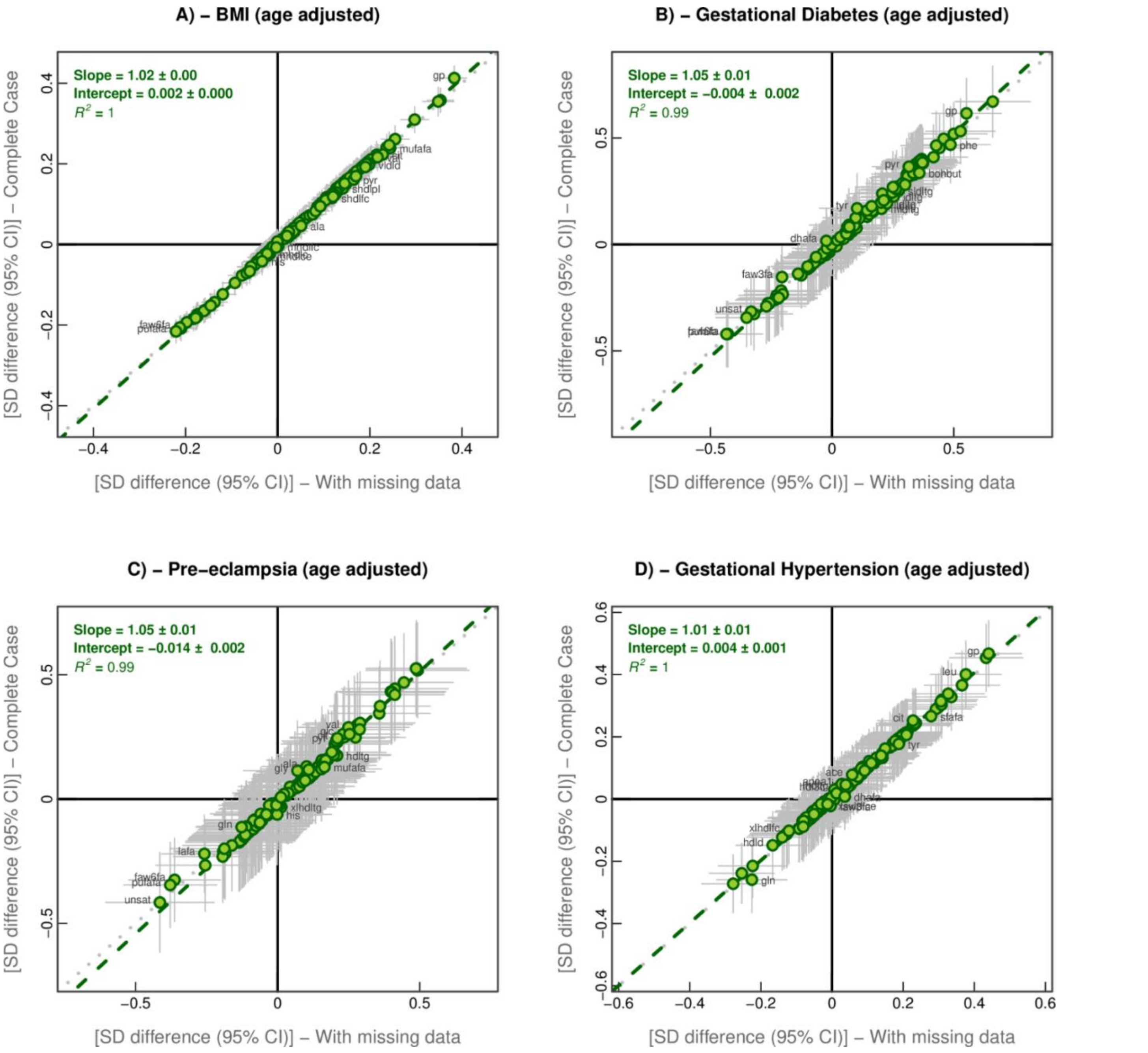

Figure S20. Sensitivity Analysis 1: Linear fit between missing data and complete case analyses for age-adjusted models (green dashed line): maternal BMI (a), gestational diabetes (b), pre-eclampsia (c) and gestational hypertension (d) in *White European* women. Each green dot represents a metabolic trait. The positions of the dots are determined by difference in mean maternal metabolite (in SD units) for each increase of 1-SD maternal exposure in the main analyses whereby data were not complete (x-axis) and difference in mean maternal metabolite (in SD units) for each increase in 1-SD maternal exposure in complete-case analyses (y-axis). For dichotomous exposures, the differences are in SD units between the exposures and non-exposed. Missing data is described in table 1 of the main text. The horizontal grey lines on each dot denote the confidence intervals (CI) for main analyses associations and the vertical grey lines indicate the CI for complete-case estimates. A linear fit of the overall concordance (the similarity in magnitude between complete case and non complete case associations) is shown by the green dashed line. A slope of 1 with an intercept of 0 (dashed grey line), with all green dots sitting on that line, would indicate that results had the same magnitude and direction. The proportion of variance in non complete case associations that is explained by complete case associations (R2) in these analyses provides a measure of goodness of linear fit, with higher values (closer to 1) indicating higher consistency between complete case.

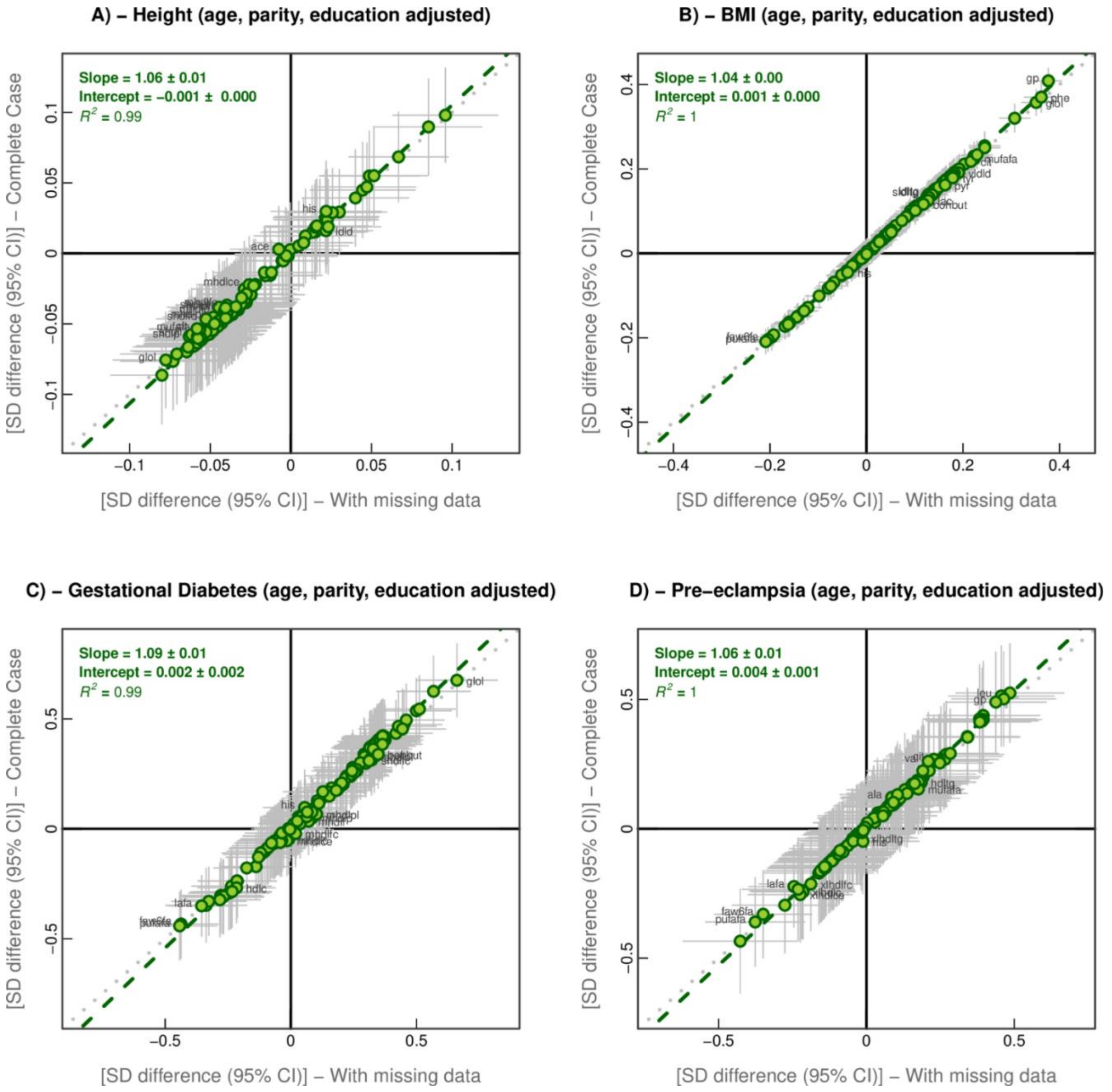

Figure S21. Sensitivity Analysis 1: Linear fit between missing data and complete case analyses for age, parity and education-adjusted models (green dashed line): maternal height (a), BMI (b) gestational diabetes (c) and pre-eclampsia (d) in *White European* women. Each green dot represents a metabolic trait. The positions of the dots are determined by difference in mean maternal metabolite (in SD units) for each increase of 1-SD maternal exposure in the main analyses whereby data were not complete (x-axis) and difference in mean maternal metabolite (in SD units) for each increase in 1-SD maternal exposure in complete-case analyses (y-axis). For dichotomous exposures, the differences are in SD units between the exposures and non-exposed. Missing data is described in table 1 of the main text. The horizontal grey lines on each dot denote the confidence intervals (CI) for main analyses associations and the vertical grey lines indicate the CI for complete-case estimates. A linear fit of the overall concordance (the similarity in magnitude between complete case and non complete case associations) is shown by the green dashed line. A slope of 1 with an intercept of 0 (dashed grey line), with all green dots sitting on that line, would indicate that results had the same magnitude and direction. The proportion of variance in non complete case associations that is explained by complete case associations (R2) in these analyses provides a measure of goodness of linear fit, with higher values (closer to 1) indicating higher consistency between complete case.

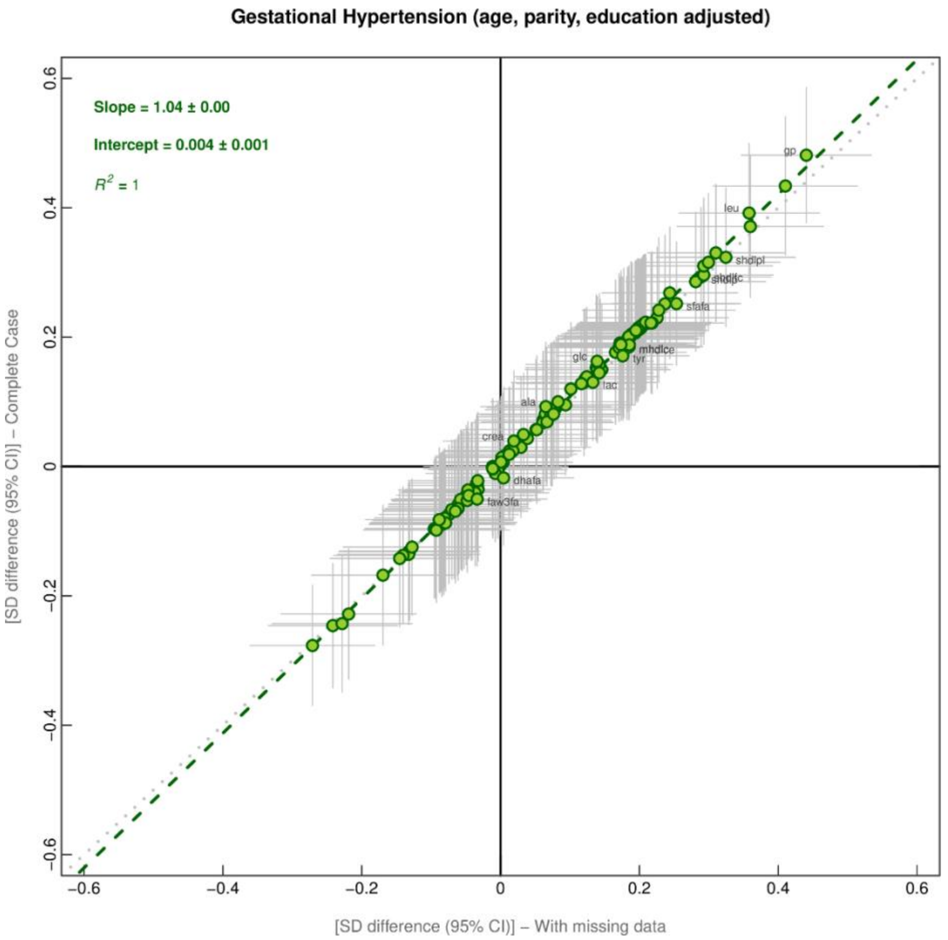

Figure S22. Sensitivity Analysis 1: Linear fit between missing data and complete case analyses for age, parity, education and BMI-adjusted models (green dashed line) for maternal gestational hypertension in *White European* women. Each green dot represents a metabolic trait. The positions of the dots are determined by difference in mean maternal metabolite (in SD units) for each increase of 1-SD maternal exposure in the main analyses whereby data were not complete (x-axis) and difference in mean maternal metabolite (in SD units) for each increase in 1-SD maternal exposure in complete-case analyses (y-axis). For dichotomous exposures, the differences are in SD units between the exposures and non-exposed. Missing data is described in table 1 of the main text. The horizontal grey lines on each dot denote the confidence intervals (CI) for main analyses associations and the vertical grey lines indicate the CI for complete-case estimates. A linear fit of the overall concordance (the similarity in magnitude between complete case and non complete case associations) is shown by the green dashed line. A slope of 1 with an intercept of 0 (dashed grey line), with all green dots sitting on that line, would indicate that results had the same magnitude and direction. The proportion of variance in non complete case associations that is explained by complete case associations (R2) in these analyses provides a measure of goodness of linear fit, with higher values (closer to 1) indicating higher consistency between complete case.

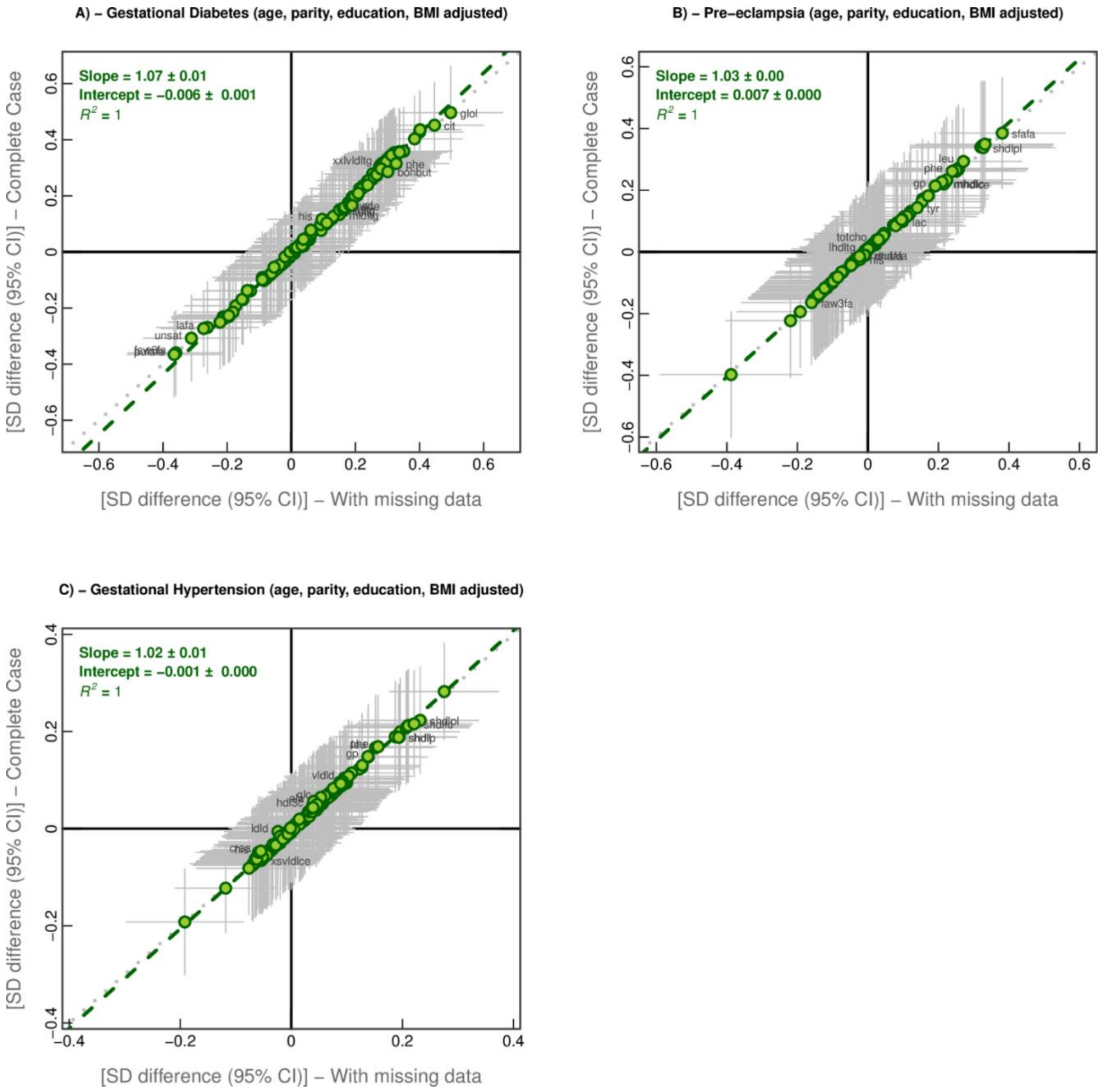

Figure S23. Sensitivity Analysis 1: Linear fit between missing data and complete case analyses for age, parity, education and BMI-adjusted models (green dashed line): gestational diabetes (a), pre-eclampsia (b) and gestational hypertension (c) in *White European* women. Each green dot represents a metabolic trait. The positions of the dots are determined by difference in mean maternal metabolite (in SD units) for each increase of 1-SD maternal exposure in the main analyses whereby data were not complete (x-axis) and difference in mean maternal metabolite (in SD units) for each increase in 1-SD maternal exposure in complete-case analyses (y-axis). For dichotomous exposures, the differences are in SD units between the exposures and non-exposed. Missing data is described in table 1 of the main text. The horizontal grey lines on each dot denote the confidence intervals (CI) for main analyses associations and the vertical grey lines indicate the CI for complete-case estimates. A linear fit of the overall concordance (the similarity in magnitude between complete case and non complete case associations) is shown by the green dashed line. A slope of 1 with an intercept of 0 (dashed grey line), with all green dots sitting on that line, would indicate that results had the same magnitude and direction. The proportion of variance in non complete case associations that is explained by complete case associations (R2) in these analyses provides a measure of goodness of linear fit, with higher values (closer to 1) indicating higher consistency between complete case.

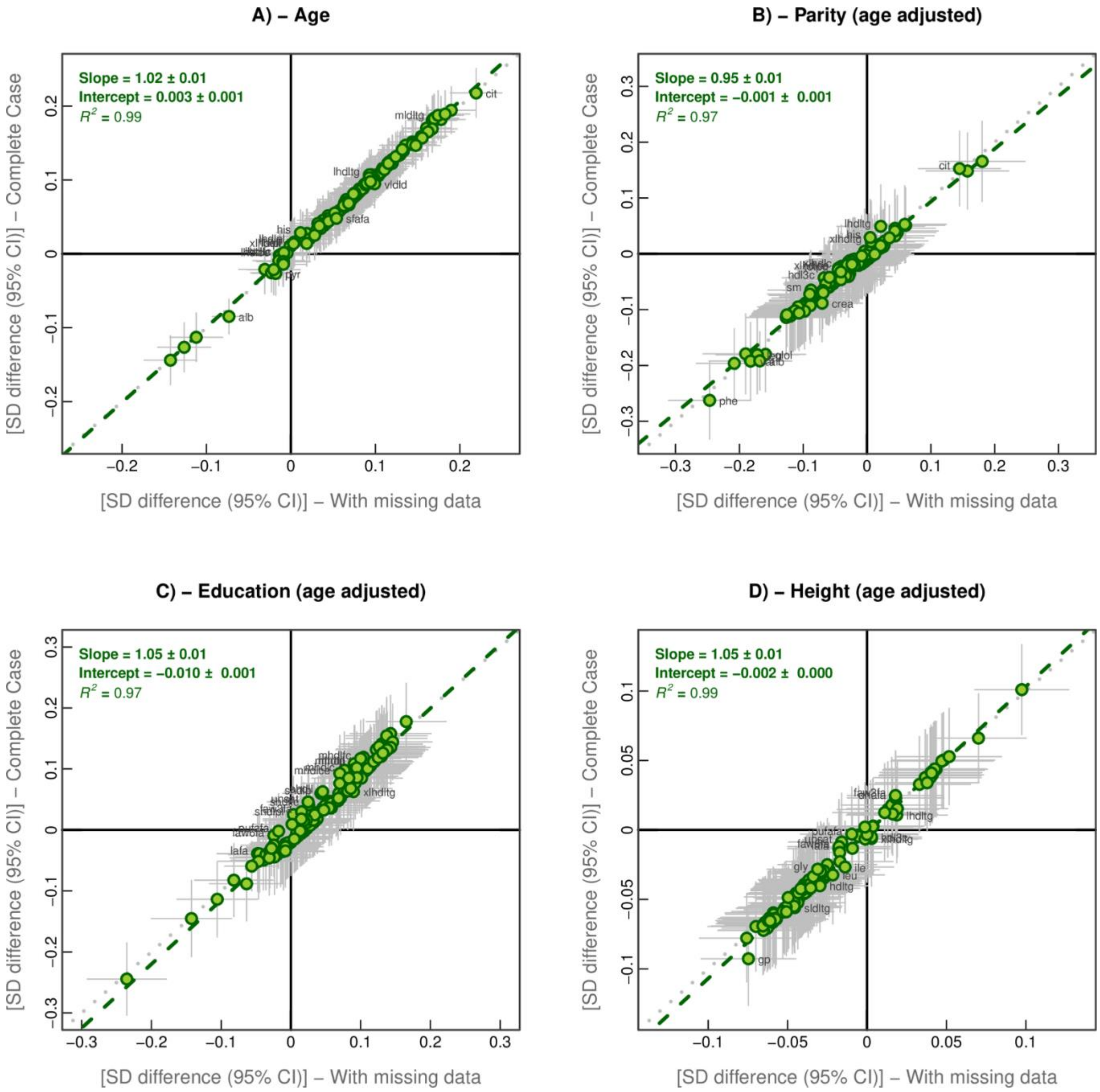

Figure S24. Sensitivity Analysis 1: Linear fit between missing data and complete case analyses for age-adjusted models (green dashed line): maternal age (a), parity (b), education (c), and height (d) in *South Asian* women. Each green dot represents a metabolic trait. The positions of the dots are determined by difference in mean maternal metabolite (in SD units) for each increase of 1-SD maternal exposure in the main analyses whereby data were not complete (x-axis) and difference in mean maternal metabolite (in SD units) for each increase in 1-SD maternal exposure in complete-case analyses (y-axis). For dichotomous exposures, the differences are in SD units between the exposures and non-exposed. Missing data is described in table 1 of the main text. The horizontal grey lines on each dot denote the confidence intervals (CI) for main analyses associations and the vertical grey lines indicate the CI for complete-case estimates. A linear fit of the overall concordance (the similarity in magnitude between complete case and non complete case associations) is shown by the green dashed line. A slope of 1 with an intercept of 0 (dashed grey line), with all green dots sitting on that line, would indicate that results had the same magnitude and direction. The proportion of variance in non complete case associations that is explained by complete case associations (R2) in these analyses provides a measure of goodness of linear fit, with higher values (closer to 1) indicating higher consistency between complete case.

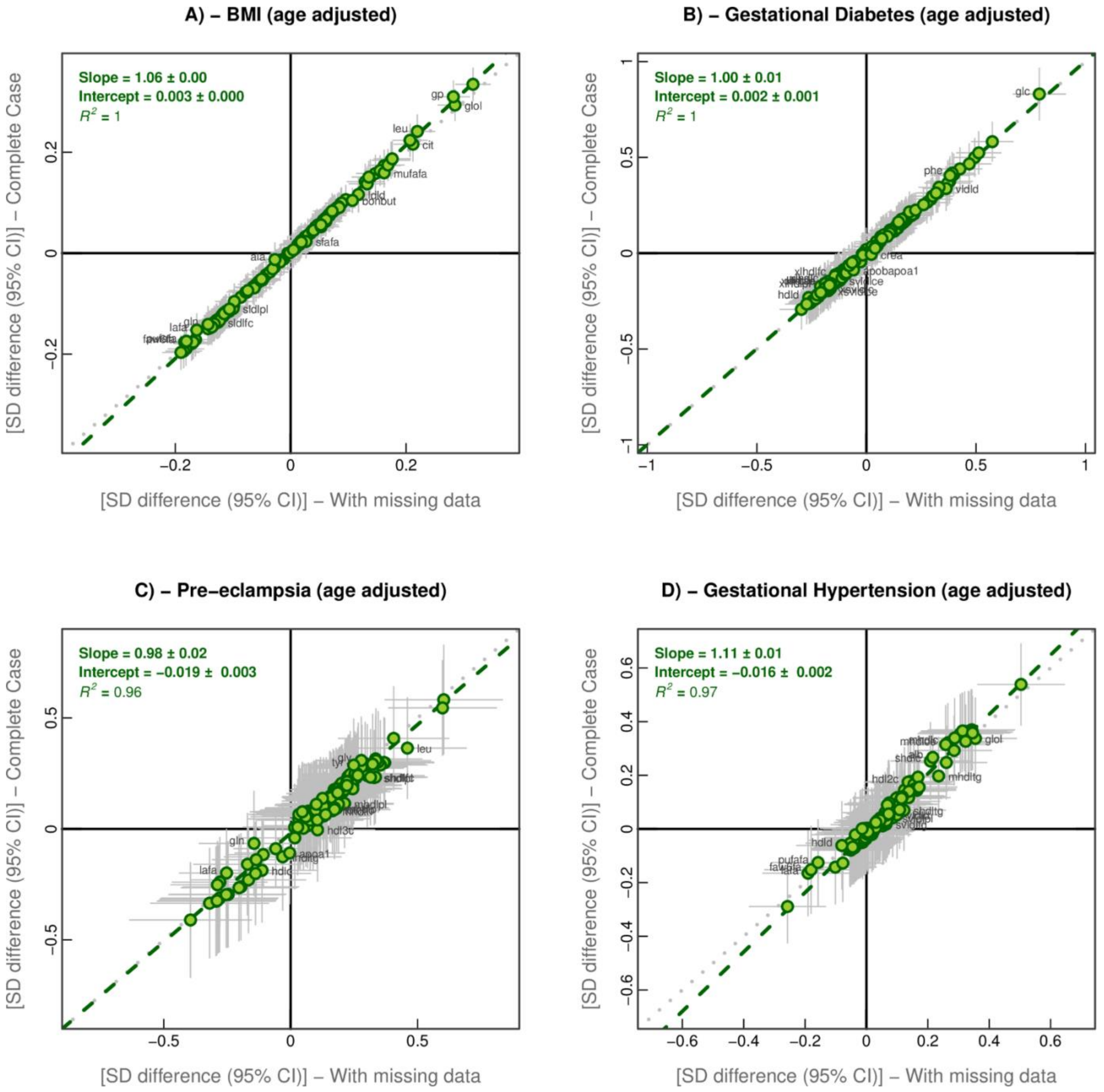

Figure S25. Sensitivity Analysis 1: Linear fit between missing data and complete case analyses for age-adjusted models (green dashed line): maternal BMI (a), gestational diabetes (b), pre-eclampsia (c) and gestational hypertension (d) in *South Asian* women. Each green dot represents a metabolic trait. The positions of the dots are determined by difference in mean maternal metabolite (in SD units) for each increase of 1-SD maternal exposure in the main analyses whereby data were not complete (x-axis) and difference in mean maternal metabolite (in SD units) for each increase in 1-SD maternal exposure in complete-case analyses (y-axis). For dichotomous exposures, the differences are in SD units between the exposures and non-exposed. Missing data is described in table 1 of the main text. The horizontal grey lines on each dot denote the confidence intervals (CI) for main analyses associations and the vertical grey lines indicate the CI for complete-case estimates. A linear fit of the overall concordance (the similarity in magnitude between complete case and non complete case associations) is shown by the green dashed line. A slope of 1 with an intercept of 0 (dashed grey line), with all green dots sitting on that line, would indicate that results had the same magnitude and direction. The proportion of variance in non complete case associations that is explained by complete case associations (R2) in these analyses provides a measure of goodness of linear fit, with higher values (closer to 1) indicating higher consistency between complete case.

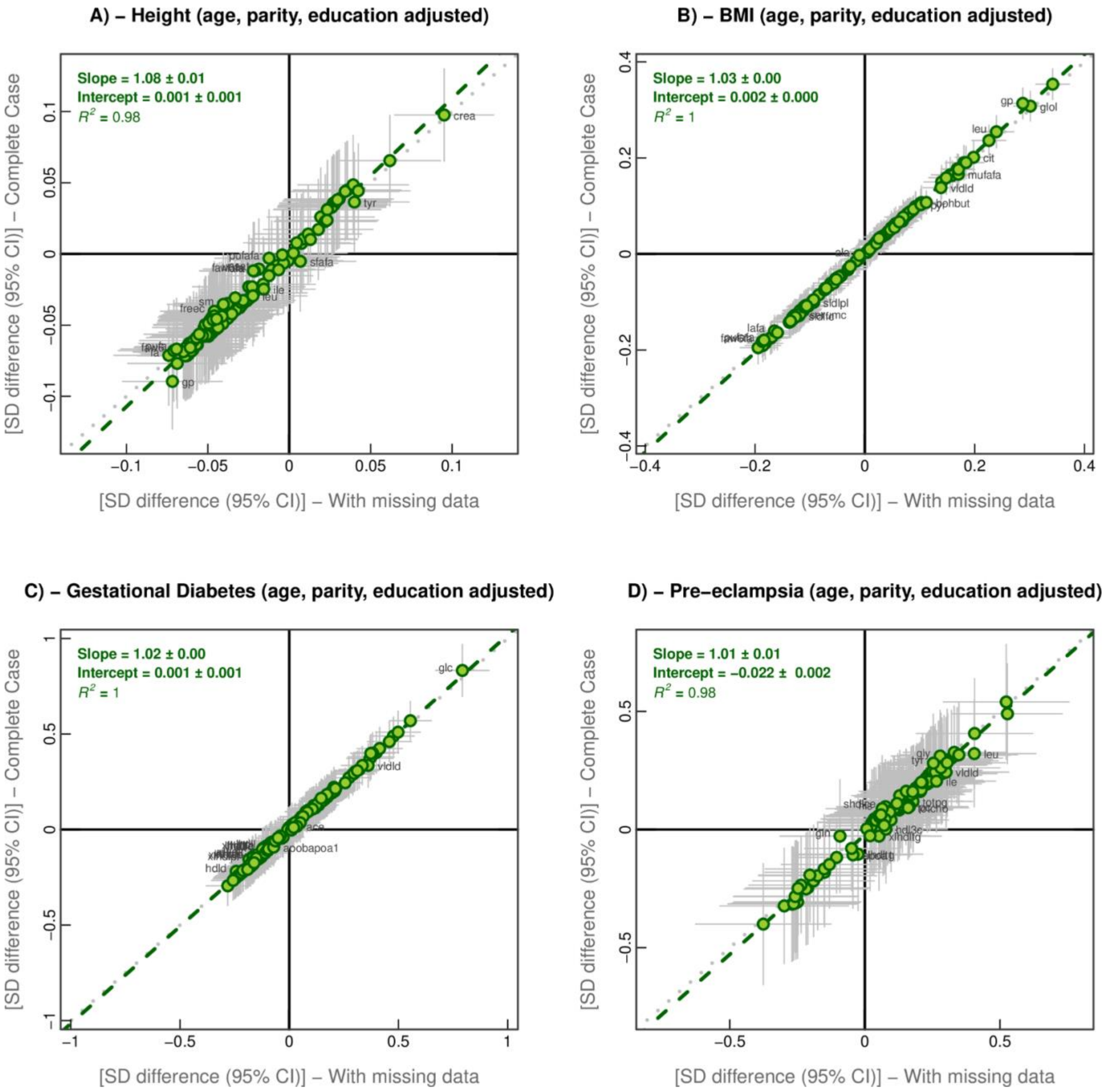

Figure S26. Sensitivity Analysis 1: Linear fit between missing data and complete case analyses for age, parity and education-adjusted models (green dashed line): maternal height (a), BMI (b) gestational diabetes (c) and pre-eclampsia (d) in *South Asian* women. Each green dot represents a metabolic trait. The positions of the dots are determined by difference in mean maternal metabolite (in SD units) for each increase of 1-SD maternal exposure in the main analyses whereby data were not complete (x-axis) and difference in mean maternal metabolite (in SD units) for each increase in 1-SD maternal exposure in complete-case analyses (y-axis). For dichotomous exposures, the differences are in SD units between the exposures and non-exposed. Missing data is described in table 1 of the main text. The horizontal grey lines on each dot denote the confidence intervals (CI) for main analyses associations and the vertical grey lines indicate the CI for complete-case estimates. A linear fit of the overall concordance (the similarity in magnitude between complete case and non complete case associations) is shown by the green dashed line. A slope of 1 with an intercept of 0 (dashed grey line), with all green dots sitting on that line, would indicate that results had the same magnitude and direction. The proportion of variance in non complete case associations that is explained by complete case associations (R2) in these analyses provides a measure of goodness of linear fit, with higher values (closer to 1) indicating higher consistency between complete case.

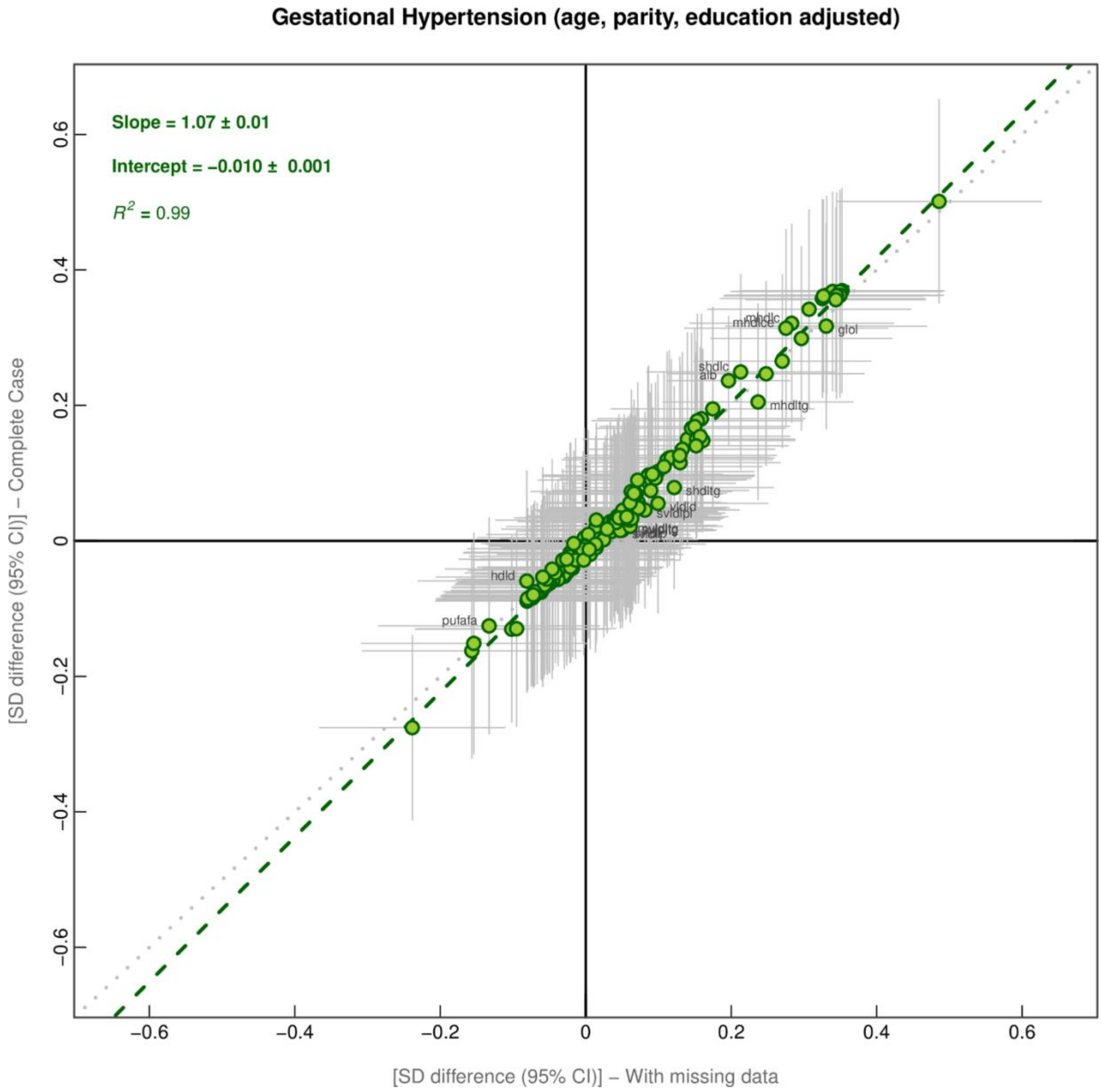

Figure S27. Sensitivity Analysis 1: Linear fit between missing data and complete case analyses for age, parity, education and BMI-adjusted models (green dashed line) for maternal gestational hypertension in *South Asian* women. Each green dot represents a metabolic trait. The positions of the dots are determined by difference in mean maternal metabolite (in SD units) for each increase of 1-SD maternal exposure in the main analyses whereby data were not complete (x-axis) and difference in mean maternal metabolite (in SD units) for each increase in 1-SD maternal exposure in complete-case analyses (y-axis). For dichotomous exposures, the differences are in SD units between the exposures and non-exposed. Missing data is described in table 1 of the main text. The horizontal grey lines on each dot denote the confidence intervals (CI) for main analyses associations and the vertical grey lines indicate the CI for complete-case estimates. A linear fit of the overall concordance (the similarity in magnitude between complete case and non complete case associations) is shown by the green dashed line. A slope of 1 with an intercept of 0 (dashed grey line), with all green dots sitting on that line, would indicate that results had the same magnitude and direction. The proportion of variance in non complete case associations that is explained by complete case associations (R2) in these analyses provides a measure of goodness of linear fit, with higher values (closer to 1) indicating higher consistency between complete case.

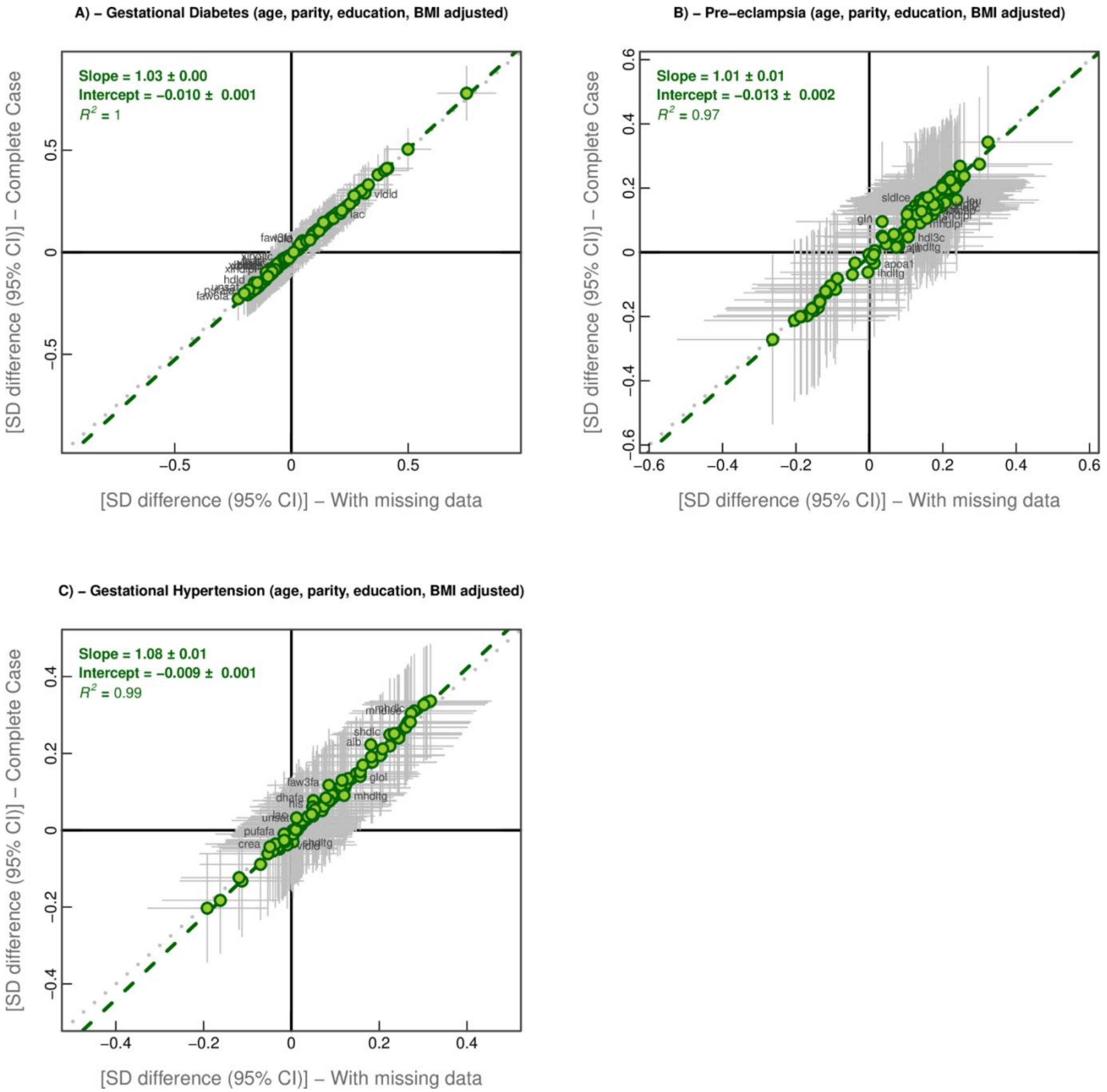

Figure S28. Sensitivity Analysis 1: Linear fit between missing data and complete case analyses for age, parity, education and BMI-adjusted models (green dashed line): gestational diabetes (a), pre-eclampsia (b) and gestational hypertension (c) in *South Asian* women. Each green dot represents a metabolic trait. The positions of the dots are determined by difference in mean maternal metabolite (in SD units) for each increase of 1-SD maternal exposure in the main analyses whereby data were not complete (x-axis) and difference in mean maternal metabolite (in SD units) for each increase in 1-SD maternal exposure in complete-case analyses (y-axis). For dichotomous exposures, the differences are in SD units between the exposures and non-exposed. Missing data is described in table 1 of the main text. The horizontal grey lines on each dot denote the confidence intervals (CI) for main analyses associations and the vertical grey lines indicate the CI for complete-case estimates. A linear fit of the overall concordance (the similarity in magnitude between complete case and non complete case associations) is shown by the green dashed line. A slope of 1 with an intercept of 0 (dashed grey line), with all green dots sitting on that line, would indicate that results had the same magnitude and direction. The proportion of variance in non complete case associations that is explained by complete case associations (R2) in these analyses provides a measure of goodness of linear fit, with higher values (closer to 1) indicating higher consistency between complete case.

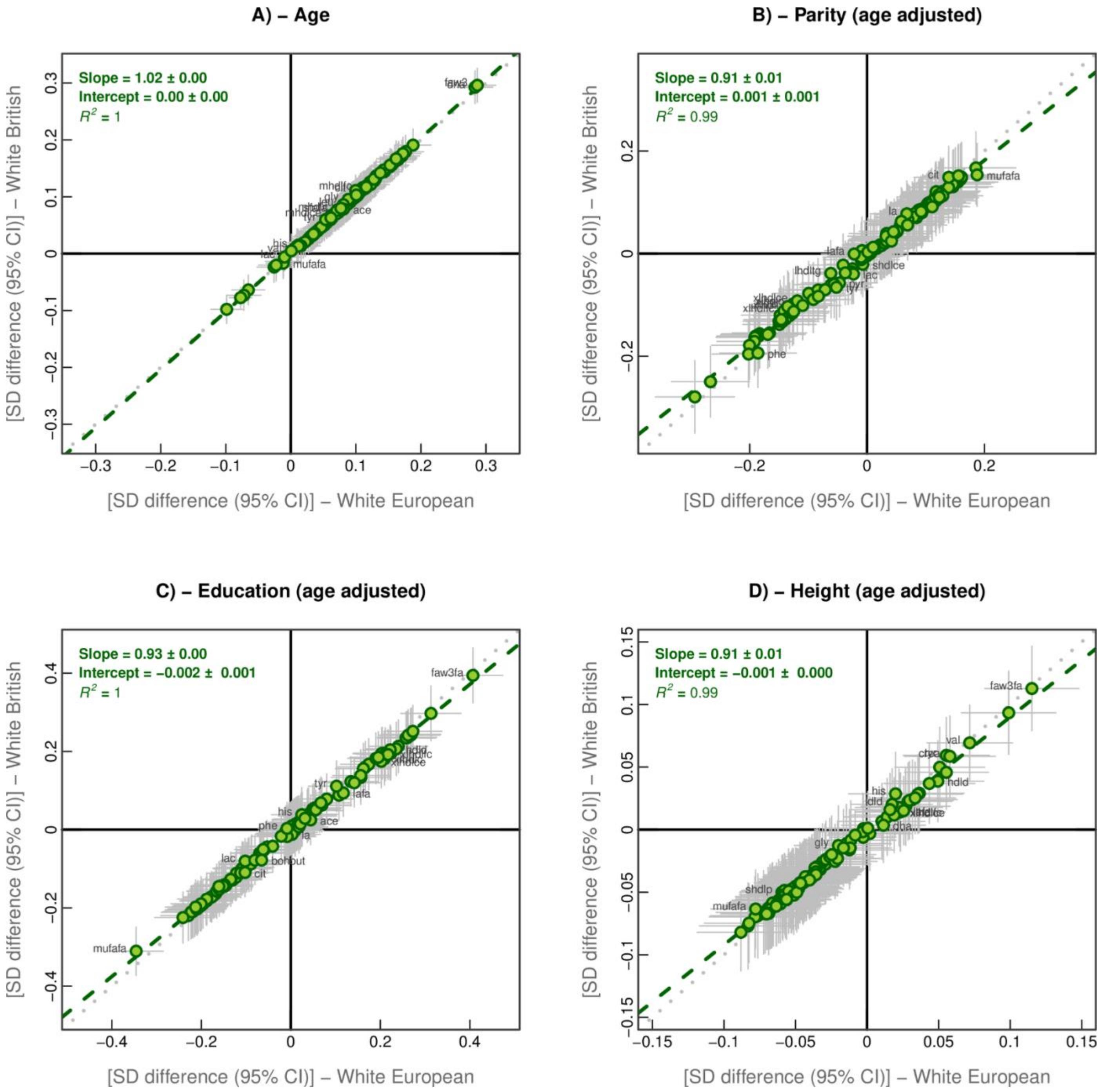

Figure S29. Sensitivity Analysis 2: Linear fit between *White European and White British* age-adjusted models (green dashed line) for maternal age (a), parity (b), education (c), and height (d). Each green dot represents a metabolic trait. The positions of the dots are determined by difference in mean maternal metabolite (in SD units) for each increase of 1-SD maternal exposure in the main analyses ethnic group (x-axis) and difference in mean maternal metabolite (in SD units) for each increase in 1-SD maternal exposure in the sensitivity analysis ethnic group (y-axis). For dichotomous exposures, the differences are in SD units between the exposures and non-exposed. The horizontal grey lines on each dot denote the confidence intervals (CI) for main analyses associations and the vertical grey lines indicate the CI for sensitivity analysis estimates. A linear fit of the overall concordance (the similarity in magnitude between the two associations) is shown by the green dashed line. A slope of 1 with an intercept of 0 (dashed grey line), with all green dots sitting on that line, would indicate that results had the same magnitude and direction. The proportion of variance in main analysis associations that is explained by sensitivity ethnic group associations (R2) in these analyses provides a measure of goodness of linear fit, with higher values (closer to 1) indicating higher consistency.

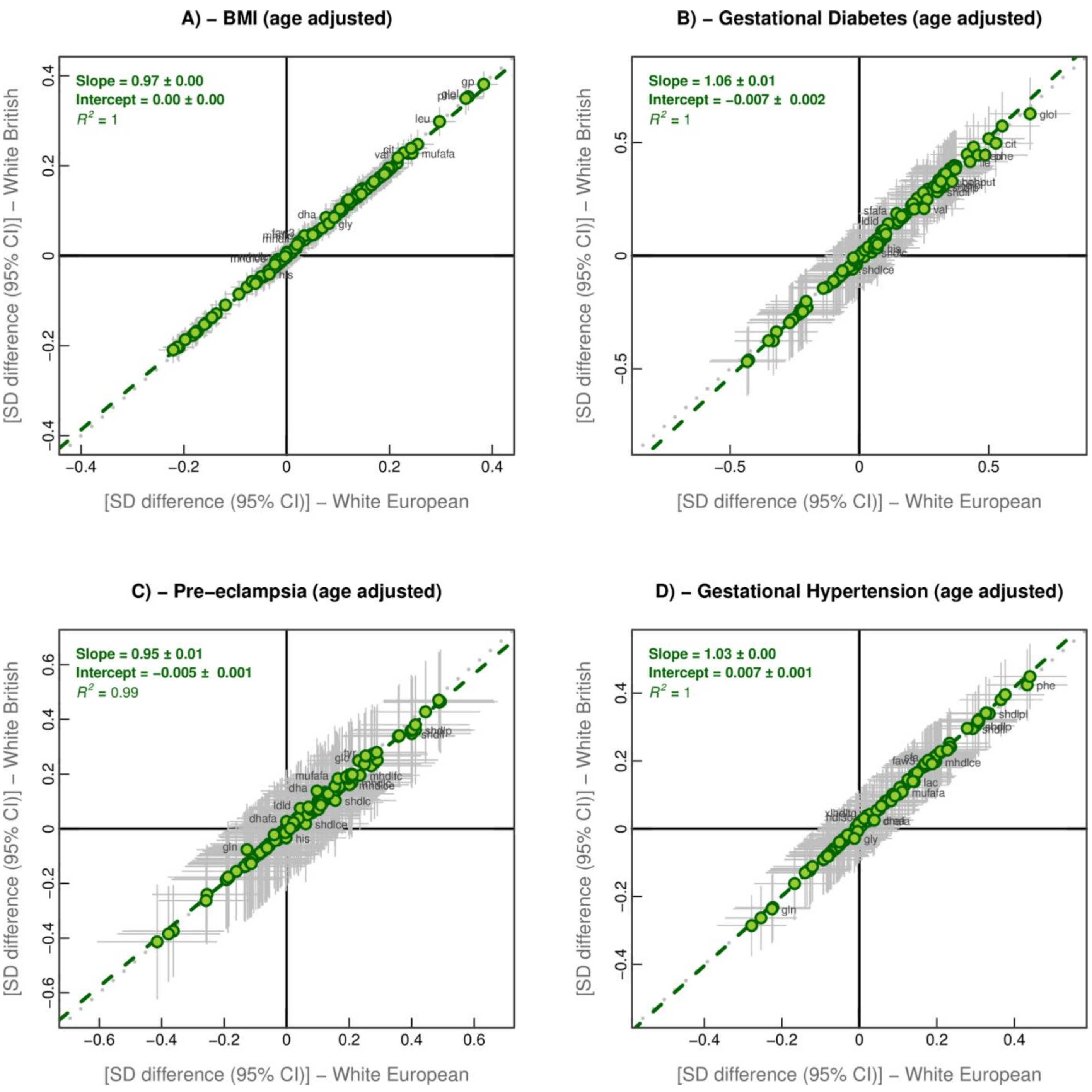

Figure S30. Sensitivity Analysis 2: Linear fit between *White European and White British* age-adjusted models (green dashed line) for maternal BMI (a), gestational diabetes (b), pre-eclampsia (c) and gestational hypertension (d). Each green dot represents a metabolic trait. The positions of the dots are determined by difference in mean maternal metabolite (in SD units) for each increase of 1-SD maternal exposure in the main analyses ethnic group (x-axis) and difference in mean maternal metabolite (in SD units) for each increase in 1-SD maternal exposure in the sensitivity analysis ethnic group (y-axis). For dichotomous exposures, the differences are in SD units between the exposures and non-exposed. The horizontal grey lines on each dot denote the confidence intervals (CI) for main analyses associations and the vertical grey lines indicate the CI for sensitivity analysis estimates. A linear fit of the overall concordance (the similarity in magnitude between the two associations) is shown by the green dashed line. A slope of 1 with an intercept of 0 (dashed grey line), with all green dots sitting on that line, would indicate that results had the same magnitude and direction. The proportion of variance in main analysis associations that is explained by sensitivity ethnic group associations (R2) in these analyses provides a measure of goodness of linear fit, with higher values (closer to 1) indicating higher consistency.

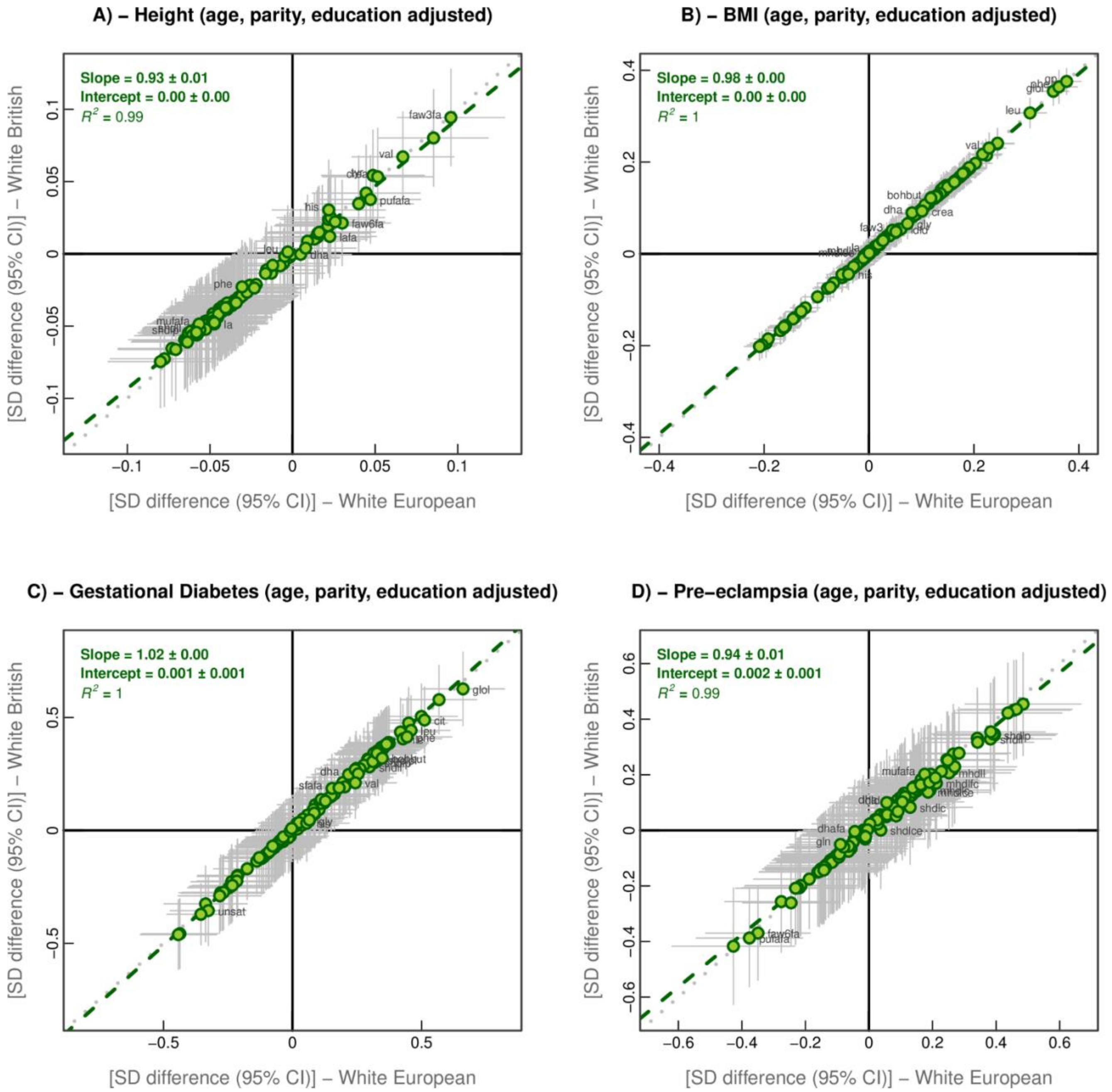

Figure S31. Sensitivity Analysis 2: Linear fit between *White European and White British* age, parity and education-adjusted models (green dashed line) for maternal height (a), BMI (b) gestational diabetes (c) and pre-eclampsia (d). Each green dot represents a metabolic trait. The positions of the dots are determined by difference in mean maternal metabolite (in SD units) for each increase of 1-SD maternal exposure in the main analyses ethnic group (x-axis) and difference in mean maternal metabolite (in SD units) for each increase in 1-SD maternal exposure in the sensitivity analysis ethnic group (y-axis). For dichotomous exposures, the differences are in SD units between the exposures and non-exposed. The horizontal grey lines on each dot denote the confidence intervals (CI) for main analyses associations and the vertical grey lines indicate the CI for sensitivity analysis estimates. A linear fit of the overall concordance (the similarity in magnitude between the two associations) is shown by the green dashed line. A slope of 1 with an intercept of 0 (dashed grey line), with all green dots sitting on that line, would indicate that results had the same magnitude and direction. The proportion of variance in main analysis associations that is explained by sensitivity ethnic group associations (R2) in these analyses provides a measure of goodness of linear fit, with higher values (closer to 1) indicating higher consistency.

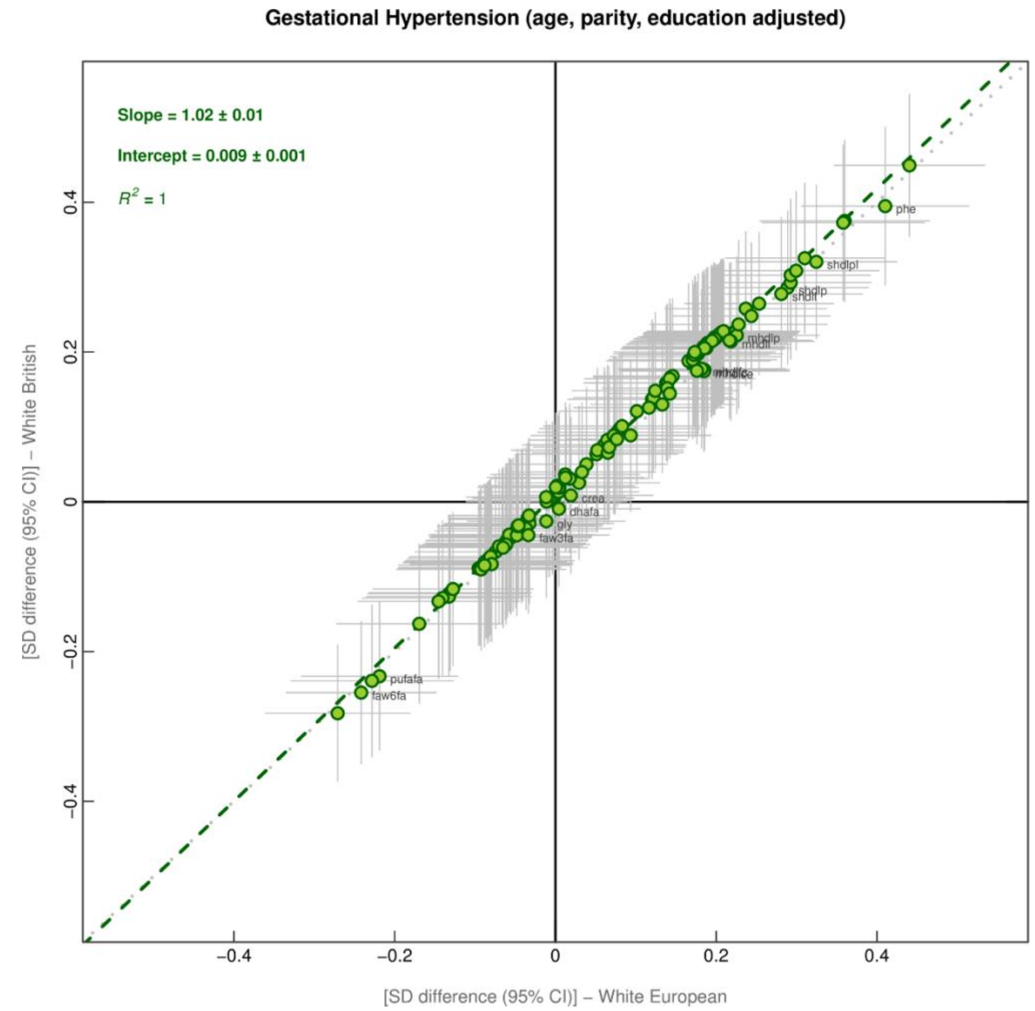

Figure S32. Sensitivity Analysis 2: Linear fit between ***White European and White British*** **age, parity, education-adjusted models (green dashed line) for maternal gestational hypertension.** Each green dot represents a metabolic trait. The positions of the dots are determined by difference in mean maternal metabolite (in SD units) for each increase of 1-SD maternal exposure in the main analyses ethnic group (x-axis) and difference in mean maternal metabolite (in SD units) for each increase in 1-SD maternal exposure in the sensitivity analysis ethnic group (y-axis). For dichotomous exposures, the differences are in SD units between the exposures and non-exposed. The horizontal grey lines on each dot denote the confidence intervals (CI) for main analyses associations and the vertical grey lines indicate the CI for sensitivity analysis estimates. A linear fit of the overall concordance (the similarity in magnitude between the two associations) is shown by the green dashed line. A slope of 1 with an intercept of 0 (dashed grey line), with all green dots sitting on that line, would indicate that results had the same magnitude and direction. The proportion of variance in main analysis associations that is explained by sensitivity ethnic group associations (R2) in these analyses provides a measure of goodness of linear fit, with higher values (closer to 1) indicating higher consistency.

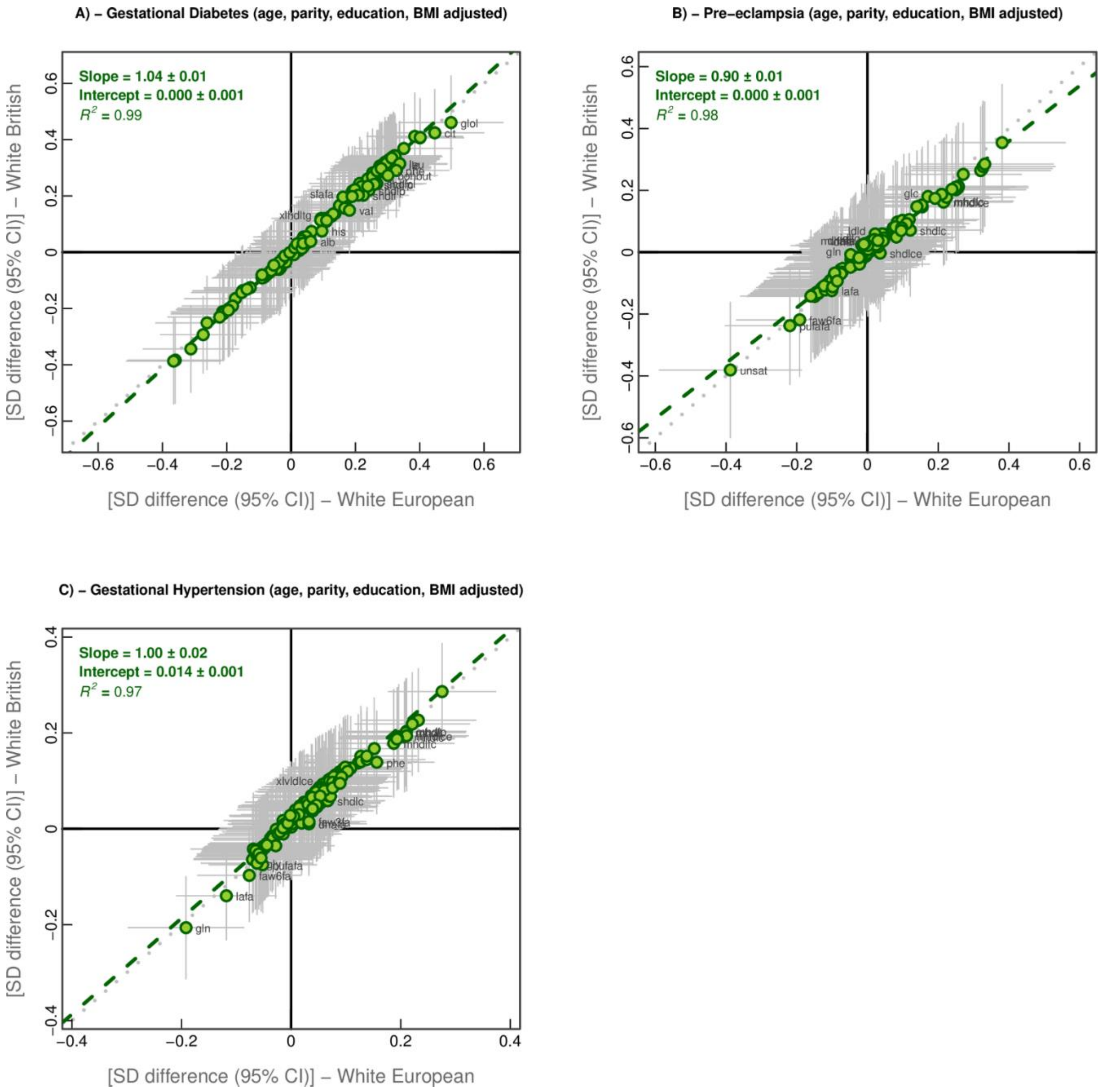

Figure S33. Sensitivity Analysis 2: Linear fit between *White European and White British* age, parity, education and BMI-adjusted models (green dashed line) for maternal gestational diabetes (a), pre-eclampsia (b) and gestational hypertension (c). Each green dot represents a metabolic trait. The positions of the dots are determined by difference in mean maternal metabolite (in SD units) for each increase of 1-SD maternal exposure in the main analyses ethnic group (x-axis) and difference in mean maternal metabolite (in SD units) for each increase in 1-SD maternal exposure in the sensitivity analysis ethnic group (y-axis). For dichotomous exposures, the differences are in SD units between the exposures and non-exposed. The horizontal grey lines on each dot denote the confidence intervals (CI) for main analyses associations and the vertical grey lines indicate the CI for sensitivity analysis estimates. A linear fit of the overall concordance (the similarity in magnitude between the two associations) is shown by the green dashed line. A slope of 1 with an intercept of 0 (dashed grey line), with all green dots sitting on that line, would indicate that results had the same magnitude and direction. The proportion of variance in main analysis associations that is explained by sensitivity ethnic group associations (R2) in these analyses provides a measure of goodness of linear fit, with higher values (closer to 1) indicating higher consistency.

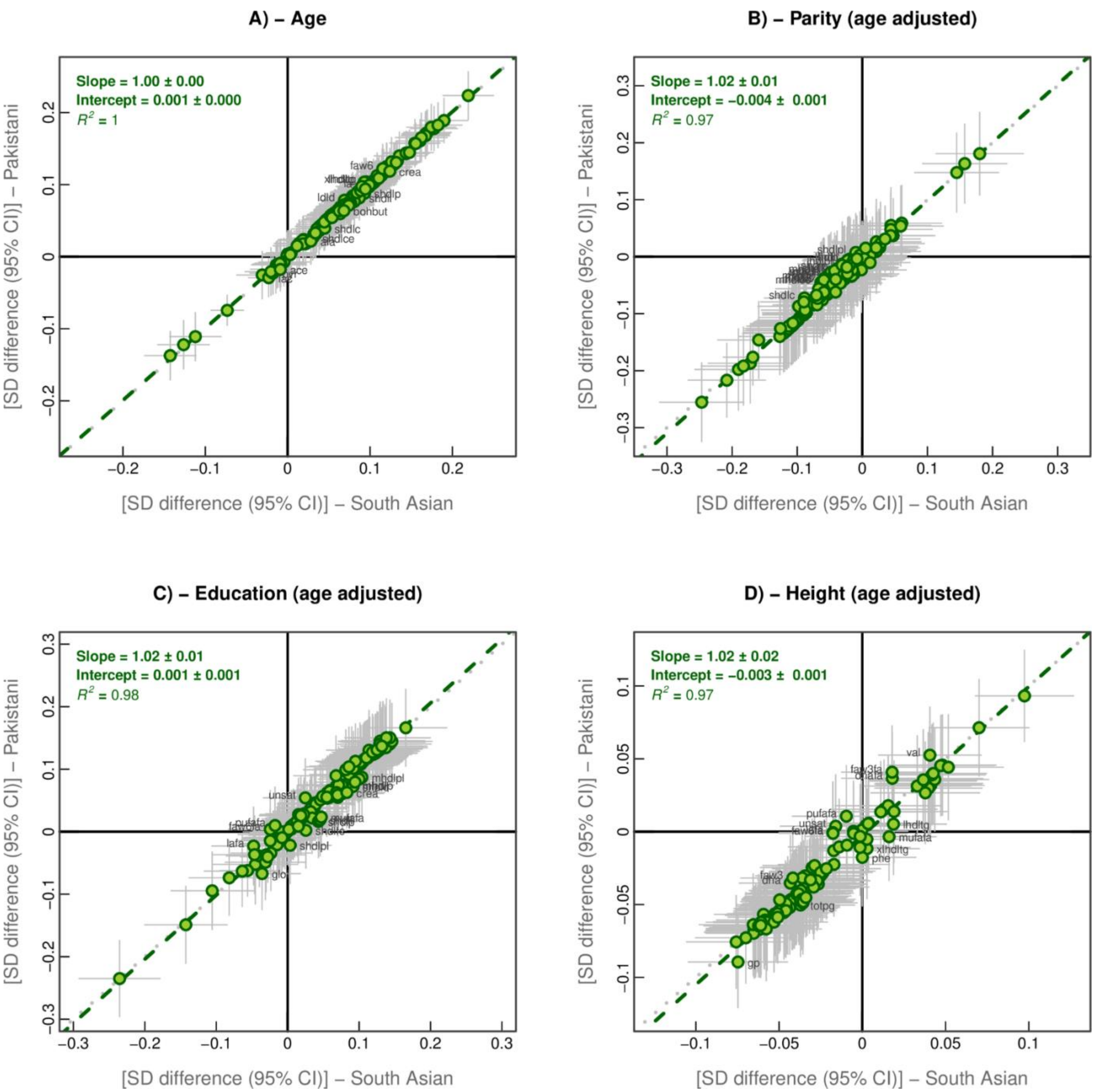

Figure S34. Sensitivity Analysis 2: Linear fit between *South Asian and Pakistani* age-adjusted models (green dashed line) for maternal age (a), parity (b), education (c), and height (d). Each green dot represents a metabolic trait. The positions of the dots are determined by difference in mean maternal metabolite (in SD units) for each increase of 1-SD maternal exposure in the main analyses ethnic group (x-axis) and difference in mean maternal metabolite (in SD units) for each increase in 1-SD maternal exposure in the sensitivity analysis ethnic group (y-axis). For dichotomous exposures, the differences are in SD units between the exposures and non-exposed. The horizontal grey lines on each dot denote the confidence intervals (CI) for main analyses associations and the vertical grey lines indicate the CI for sensitivity analysis estimates. A linear fit of the overall concordance (the similarity in magnitude between the two associations) is shown by the green dashed line. A slope of 1 with an intercept of 0 (dashed grey line), with all green dots sitting on that line, would indicate that results had the same magnitude and direction. The proportion of variance in main analysis associations that is explained by sensitivity ethnic group associations (R2) in these analyses provides a measure of goodness of linear fit, with higher values (closer to 1) indicating higher consistency.

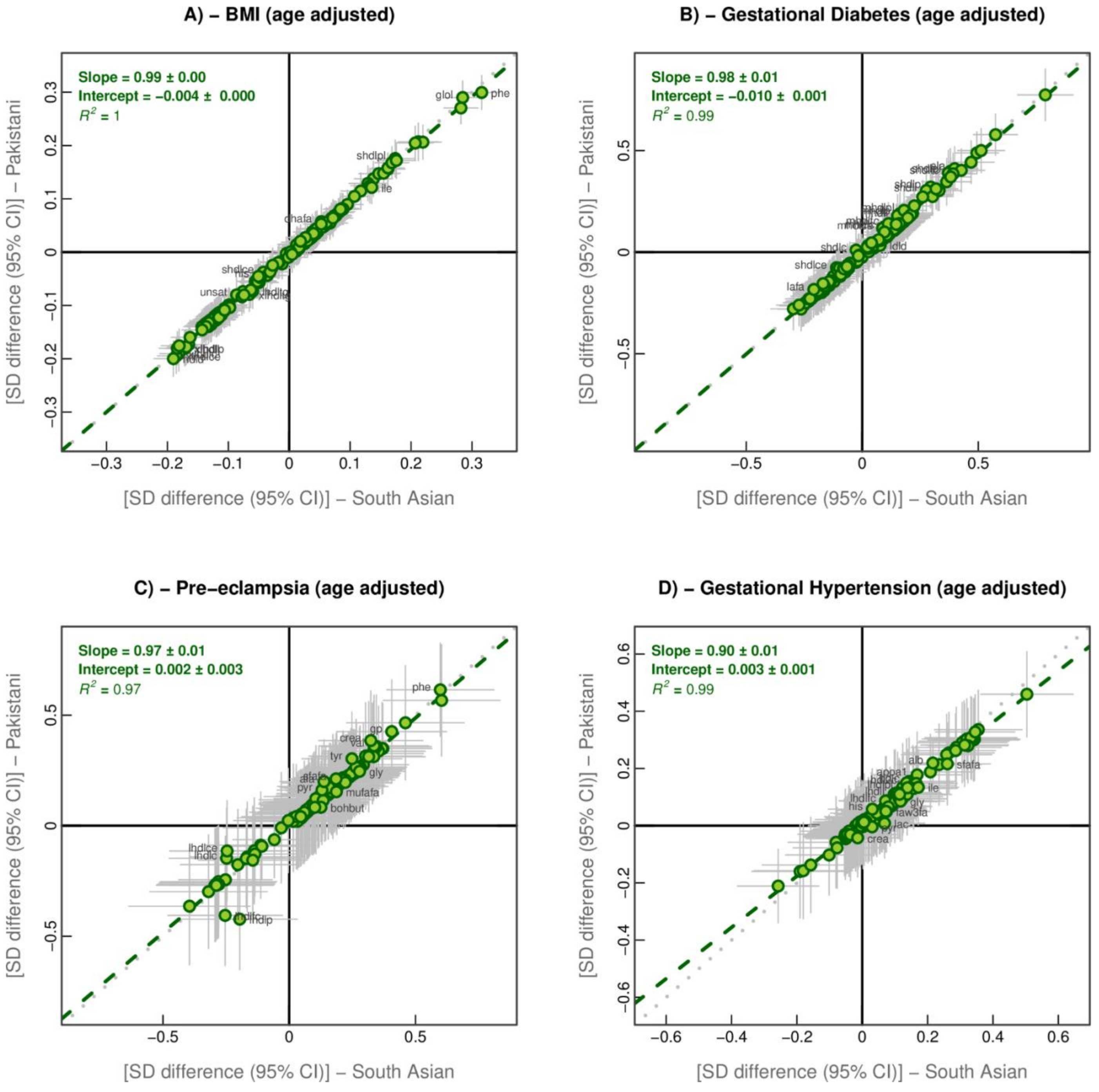

Figure S35. Sensitivity Analysis 2: Linear fit between *South Asian and Pakistani* age-adjusted models (green dashed line) for maternal BMI (a), gestational diabetes (b), pre-eclampsia (c) and gestational hypertension (d). Each green dot represents a metabolic trait. The positions of the dots are determined by difference in mean maternal metabolite (in SD units) for each increase of 1-SD maternal exposure in the main analyses ethnic group (x-axis) and difference in mean maternal metabolite (in SD units) for each increase in 1-SD maternal exposure in the sensitivity analysis ethnic group (y-axis). For dichotomous exposures, the differences are in SD units between the exposures and non-exposed. The horizontal grey lines on each dot denote the confidence intervals (CI) for main analyses associations and the vertical grey lines indicate the CI for sensitivity analysis estimates. A linear fit of the overall concordance (the similarity in magnitude between the two associations) is shown by the green dashed line. A slope of 1 with an intercept of 0 (dashed grey line), with all green dots sitting on that line, would indicate that results had the same magnitude and direction. The proportion of variance in main analysis associations that is explained by sensitivity ethnic group associations (R2) in these analyses provides a measure of goodness of linear fit, with higher values (closer to 1) indicating higher consistency.

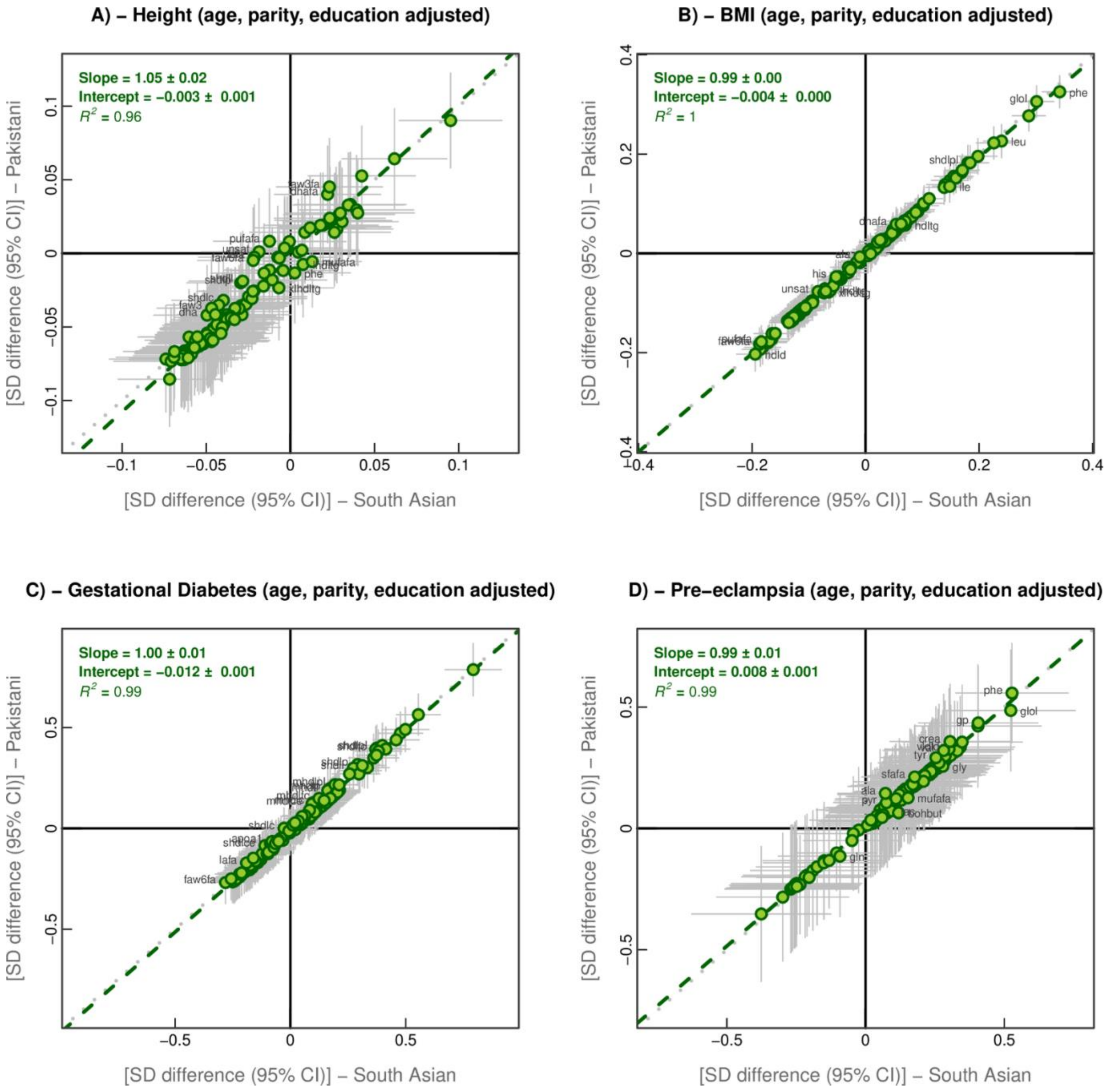

Figure S36. Sensitivity Analysis 2: Linear fit between *South Asian and Pakistani* age, parity and education-adjusted models (green dashed line) for maternal height (a), BMI (b) gestational diabetes (c) and pre-eclampsia (d). Each green dot represents a metabolic trait. The positions of the dots are determined by difference in mean maternal metabolite (in SD units) for each increase of 1-SD maternal exposure in the main analyses ethnic group (x-axis) and difference in mean maternal metabolite (in SD units) for each increase in 1-SD maternal exposure in the sensitivity analysis ethnic group (y-axis). For dichotomous exposures, the differences are in SD units between the exposures and non-exposed. The horizontal grey lines on each dot denote the confidence intervals (CI) for main analyses associations and the vertical grey lines indicate the CI for sensitivity analysis estimates. A linear fit of the overall concordance (the similarity in magnitude between the two associations) is shown by the green dashed line. A slope of 1 with an intercept of 0 (dashed grey line), with all green dots sitting on that line, would indicate that results had the same magnitude and direction. The proportion of variance in main analysis associations that is explained by sensitivity ethnic group associations (R2) in these analyses provides a measure of goodness of linear fit, with higher values (closer to 1) indicating higher consistency.

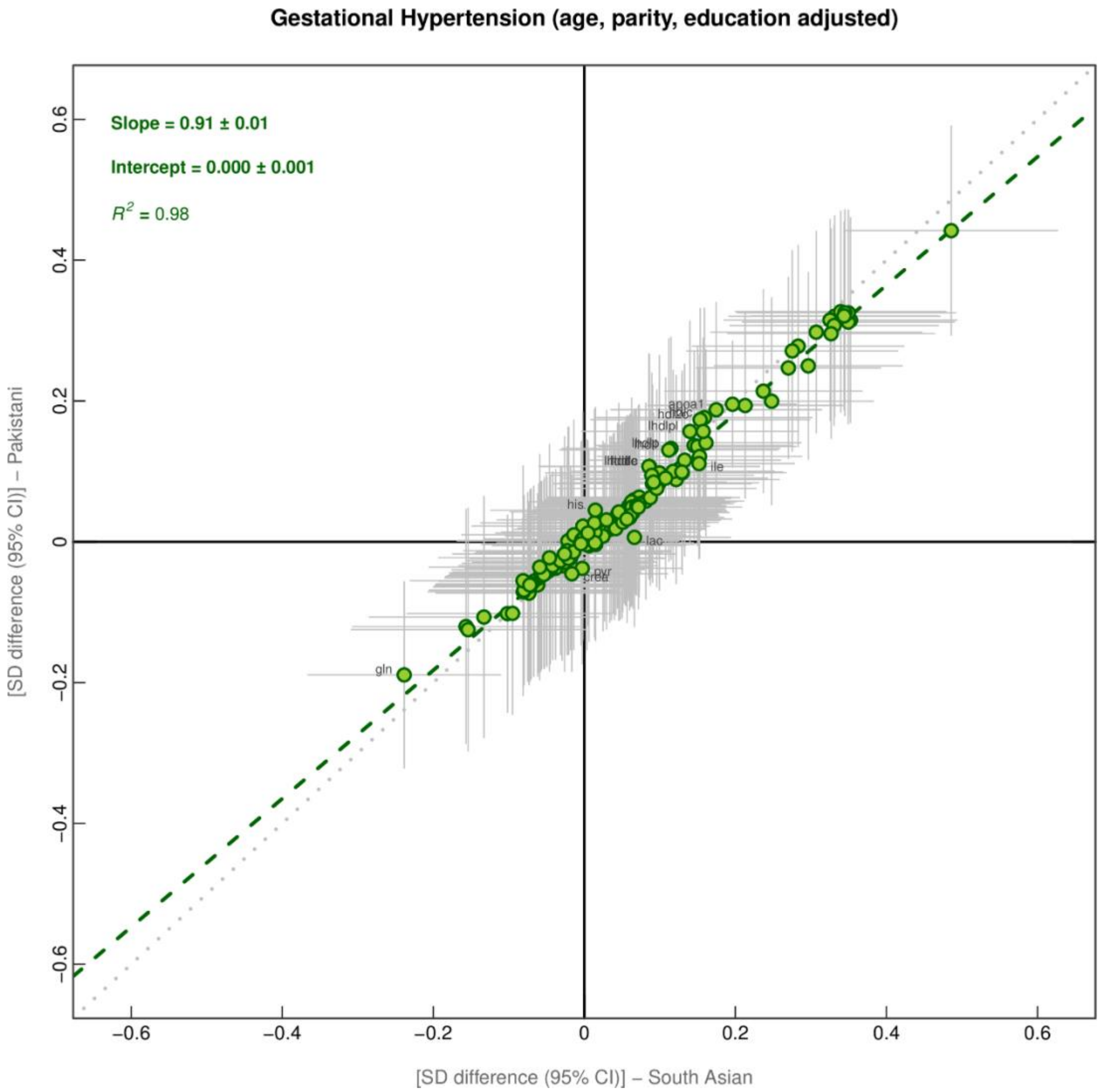

Figure S37. Sensitivity Analysis 2: Linear fit between *South Asian and Pakistani* age, parity, education-adjusted models (green dashed line) for maternal gestational hypertension. Each green dot represents a metabolic trait. The positions of the dots are determined by difference in mean maternal metabolite (in SD units) for each increase of 1-SD maternal exposure in the main analyses ethnic group (x-axis) and difference in mean maternal metabolite (in SD units) for each increase in 1-SD maternal exposure in the sensitivity analysis ethnic group (y-axis). For dichotomous exposures, the differences are in SD units between the exposures and non-exposed. The horizontal grey lines on each dot denote the confidence intervals (CI) for main analyses associations and the vertical grey lines indicate the CI for sensitivity analysis estimates. A linear fit of the overall concordance (the similarity in magnitude between the two associations) is shown by the green dashed line. A slope of 1 with an intercept of 0 (dashed grey line), with all green dots sitting on that line, would indicate that results had the same magnitude and direction. The proportion of variance in main analysis associations that is explained by sensitivity ethnic group associations (R2) in these analyses provides a measure of goodness of linear fit, with higher values (closer to 1) indicating higher consistency.

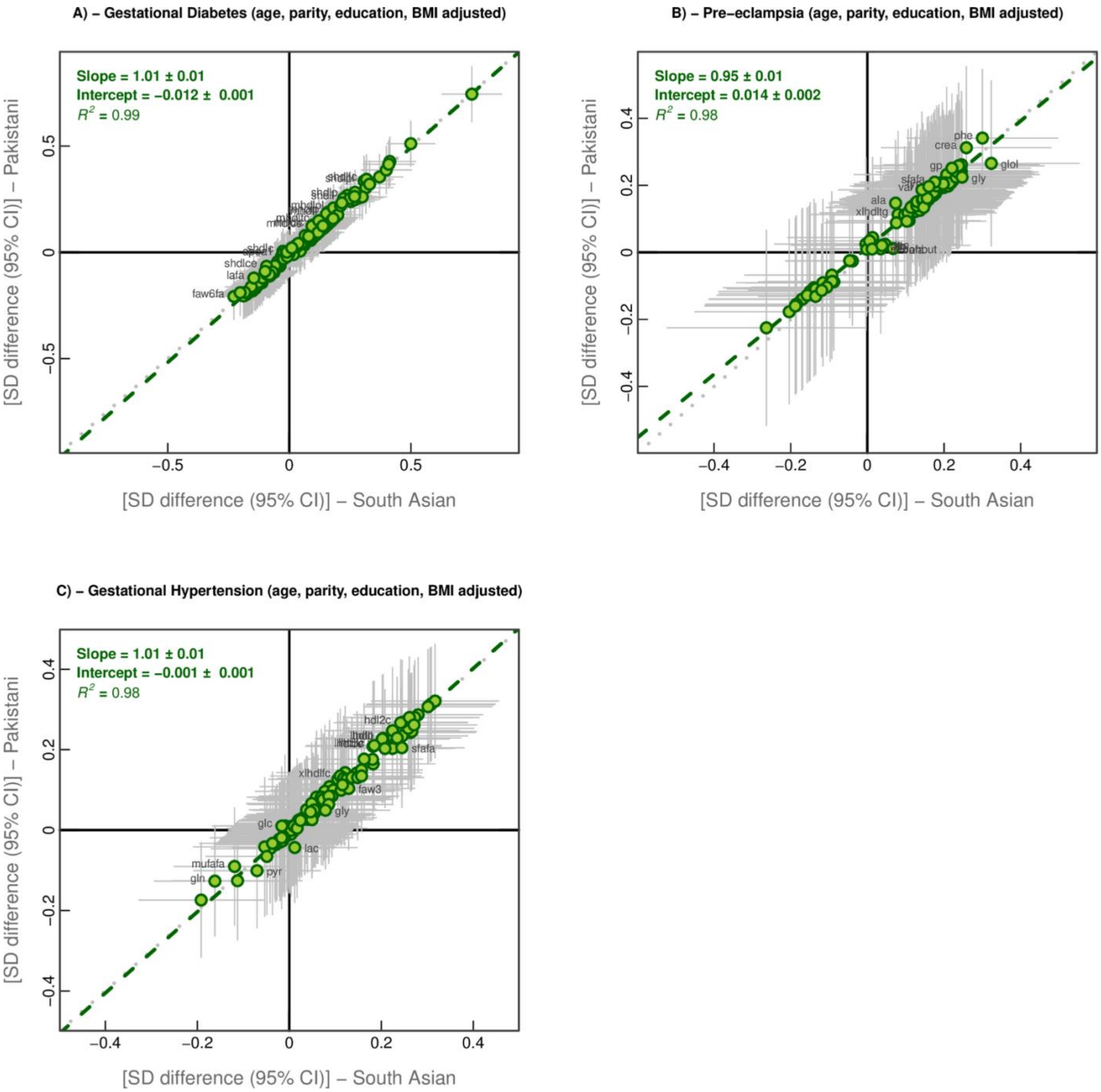

Figure S38. Sensitivity Analysis 2: Linear fit between *South Asian and Pakistani* age, parity, education and BMI-adjusted models (green dashed line) for maternal gestational diabetes (a), pre-eclampsia (b) and gestational hypertension (c). Each green dot represents a metabolic trait. The positions of the dots are determined by difference in mean maternal metabolite (in SD units) for each increase of 1-SD maternal exposure in the main analyses ethnic group (x-axis) and difference in mean maternal metabolite (in SD units) for each increase in 1-SD maternal exposure in the sensitivity analysis ethnic group (y-axis). For dichotomous exposures, the differences are in SD units between the exposures and non-exposed. The horizontal grey lines on each dot denote the confidence intervals (CI) for main analyses associations and the vertical grey lines indicate the CI for sensitivity analysis estimates. A linear fit of the overall concordance (the similarity in magnitude between the two associations) is shown by the green dashed line. A slope of 1 with an intercept of 0 (dashed grey line), with all green dots sitting on that line, would indicate that results had the same magnitude and direction. The proportion of variance in main analysis associations that is explained by sensitivity ethnic group associations (R2) in these analyses provides a measure of goodness of linear fit, with higher values (closer to 1) indicating higher consistency.
